# Ferroptosis-armed dendritic cell vaccines for glioma immunotherapy

**DOI:** 10.1038/s41467-026-72737-6

**Published:** 2026-05-07

**Authors:** Mariia Saviuk, Victoria D. Turubanova, Sara De Brée, Sandra Van Lint, Teresa Mendes Maia, Simon Devos, Iuliia Efimova, Julie Braet, Lore Van Oudenhove, Gitta Boons, Faye Naessens, Robin Demuynck, Ellen Saeys, Christian Vanhove, Lukas Bunse, Peter M. van Endert, Robrecht Raedt, Maria V. Vedunova, Olga Krysko, Roosmarijn E. Vandenbroucke, Karim Vermaelen, Tatiana A. Mishchenko, Elena Catanzaro, Dmitri V. Krysko

**Affiliations:** 1 Cell Death Investigation and Therapy (CDIT) Laboratory, Anatomy and Embryology Unit, Department of Human Structure and Repair, Faculty of Medicine and Health Sciences, Ghent University, Ghent, Belgium; 2 Cancer Research Institute Ghent, Ghent, Belgium; 3 Institute of Neurosciences, National Research Lobachevsky State University of Nizhny Novgorod, Nizhny, Russia; 4 Thoracic Tumor Immunology Laboratory (TTIL), Department of Internal Medicine and Pediatrics, Faculty of Medicine and Health Science, Ghent University, Ghent, Belgium; 5 VIB Proteomics Core, VIB, Ghent, Belgium; 6 VIB-UGent Center for Medical Biotechnology, VIB, Ghent, Belgium; 7 Department of Biomolecular Medicine, Ghent University, Ghent, Belgium; 8 myNEO Therapeutics, Ghent, Belgium; 9 IBiTech-MEDISIP-Infinity Laboratory, Department of Electronics and Information Systems, Faculty of Engineering and Architecture, Ghent University, Ghent, Belgium; 10 Clinical Cooperation Unit (CCU) Neuroimmunology and Brain Tumor Immunology, German Cancer Research Center (DKFZ), Heidelberg, Germany; 11 Neurology Clinic, Medical Faculty Mannheim, University Heidelberg, Mannheim, Germany; 12 Université Paris Cité, INSERM, CNRS, Institut Necker Enfants Malades, Paris, France; 13 Service Immunologie Biologique, AP-HP, Hôpital Universitaire Necker-Enfants Malades, Paris, France; 14 4Brain, Department of Head and Skin, Faculty of Medicine and Health Sciences, Ghent University, Ghent, Belgium; 15 Institute of Biology and Biomedicine, National Research Lobachevsky State University of Nizhny Novgorod, Nizhny, Russia; 16 VIB Center for Inflammation Research, Ghent, Belgium; 17 Department of Biomedical Molecular Biology, Faculty of Sciences, Ghent University, Ghent, Belgium

**Keywords:** Tumour vaccines, Necroptosis, Cell death and immune response, CNS cancer, Cancer immunotherapy

## Abstract

The type of cell death has proven to play a crucial role in cancer immunotherapy efficacy. Immunogenic cell death (ICD) enhances tumor adjuvanticity and antigenicity by releasing danger signals and altering the immune peptidome. The immunogenicity of ferroptosis, an iron-dependent form of cell death, remains uncertain. Here, we show that dendritic cell (DC) vaccines loaded with ferroptotic lysates protect mice against glioma growth, inducing IFN-γ production, and promoting robust CD8⁺ T cell infiltration, activation, and effector memory formation in the tumor microenvironment. The intrinsic immunogenicity of ferroptosis was independent of the glioma type and the ferroptosis inducer. Instead, it critically required the presence of the damage-associated molecular patterns calreticulin and ATP, rather than involving HMGB1–TLR4 signaling. However, supplementing these DAMPs into DC vaccines loaded with non-ICD lysates did not restore efficacy to the level of the ferroptosis-based DC vaccine, suggesting a more complex mechanism beyond a purely DAMP-mediated effect. These findings demonstrate that ferroptosis-loaded DC vaccines elicit a potent, tumor-specific immune response, capable of eradicating intracranial gliomas in mice, which highlights their potential in cancer immunotherapy.

## Abstract (web summary)

Ferroptosis is a form of programmed immunogenic cell death (ICD) that has gained increasing attention in cancer immunotherapy. In this study, we present dendritic cell vaccines loaded with ferroptotic tumor lysates to enhance antitumoral immune response in glioma models.

## Introduction

Gliomas are the most prevalent type of primary tumors in the central nervous system in adults and are associated with significant morbidity and mortality^1^. Originating from glial cells, they are classified by the WHO based on their histological and molecular genetic characteristics as glioblastomas, astrocytomas, and oligodendrogliomas^2^, with glioblastoma being the most aggressive and treatment-resistant subtype. Despite developments in complex, multidisciplinary targeted therapies, treating gliomas remains challenging due to their significant heterogeneity^3^. This heterogeneity is partly driven by the presence of tumor stem-like cells, which confer resistance to treatment. Furthermore, gliomas are often in an immunologically “cold state,” characterized by a lack of effector T-cell infiltration and inadequate immune activation^4^. Furthermore, the phenotypic and genetic diversity of tumor cells complicates treatment. Standard modalities, such as surgery, radiation therapy, and temozolomide chemotherapy, have limited success, with median overall survival ranging from 4.9 to 20.5 months^5–7^.

Recent findings highlight the immunogenicity of dying tumor cells as a critical factor influencing the therapeutic efficacy of anti-tumor treatments. Treatment success is more likely when cell death triggers an immune response specifically targeting tumor cells^8–10^. Inducing immunogenic cell death (ICD) offers two primary benefits: effective tumor cell elimination and the initiation of a targeted immune response. ICD is marked by the release or surface exposure of damage-associated molecular patterns (DAMPs), which act as adjuvants, activating innate immunity and triggering strong anti-tumor immune responses^8,11^. Additionally, ICD promotes antigenicity by releasing tumor-specific antigens that enhance the immune system’s ability to target the tumor through a combination of antigenicity and adjuvanticity. Various regulated cell death modalities, including apoptosis^12^ and necroptosis^13,14^, exhibit features of ICD.

Ferroptosis is a form of regulated cell death that is characterized by iron accumulation and the generation of reactive oxygen species (ROS) within cells. In addition, it exhibits distinct morphological, biochemical, and genetic features compared to other regulated cell death modalities^15–18^. The immunogenicity of ferroptosis remains under scientific investigation and is not yet fully understood. While the immunogenicity of ferroptotic tumor cells has been demonstrated^19,20^, cells within the tumor microenvironment undergoing ferroptosis can be immunosuppressive^21^. On the other hand, recent findings indicate that ferroptotic tumor cells are not immunogenic because they inhibit dendritic cell (DC) maturation, which impairs antigen cross-presentation^22^. This uncertainty underscores the necessity for further exploration of the mechanisms of ferroptosis immunogenicity and its role in potential applications for cancer immunotherapy.

Notably, DCs loaded with antigenic material derived from autologous tumors are widely used as DC vaccines in cancer immunotherapy, including for glioma^23^, and have been tested in several clinical trials^24,25^. However, despite the promising therapeutic potential of such DC vaccines loaded with tumor lysates, it remains unclear whether ferroptosis-derived tumor lysates are immunogenic and suitable for DC-based cancer immunotherapy.

Aiming to improve the efficacy of glioma DC immunotherapy, we investigated the immunogenicity of ferroptosis in combination with development of more effective DC vaccines for glioma. We demonstrated that DC vaccines primed with ferroptotic glioma lysates protected mice against viable glioma cell challenges in several orthotopic glioma models. These DC vaccines induced the production of interferon gamma (IFN-γ) in response to tumor antigen stimulation and were also effective in therapeutic applications. Importantly, ferroptosis-based DC vaccination enhanced CD8⁺ T cell infiltration, activation, and effector memory differentiation within the glioma microenvironment, supporting robust anti-tumor immune responses. The immunogenicity of ferroptotic lysates was independent of the glioma cell lines, the ferroptosis inducers employed, or the incorporation of additional bacterial adjuvants. This points to the strong intrinsic immunogenic potential of ferroptosis. Furthermore, we found that blocking key danger signals such as calreticulin (CRT) and ATP—but not the high mobility group box 1 (HMGB1)-Toll-like receptor 4 (TLR4) axis—significantly reduced the immunogenicity of ferroptotic glioma lysates, thereby negating the protective effect of DC vaccines in the orthotopic glioma model. Collectively, these findings suggest that DC armed vaccines with ferroptosis lysates elicit a potent and specific immune response and underscoring their potential for broader clinical application in developing DC-based cancer immunotherapies.

## Results

### Efficient induction of ferroptotic cell death in glioma cells

First, we examined the potential of inducing ferroptotic cell death in two murine glioma cell lines: GL261 and CT-2A. The glioma GL261 cell line shares many features with human glioblastomas, such as mutations in *p53* and *K-ras* and *PTEN* deficiency. It exhibits high expression levels of MHC I, indicating immunogenicity; however, it lacks co-stimulatory markers for T-cell activation, such as B7-1 and B7-2^26^. It also shares similarities with ependymoblastoma^27^. We also included the CT-2A glioma cell line which is categorized as *p53* wild type and *PTEN*-deficient, with a high mitotic index. Notably, CT-2A expresses the stem cell marker CD133 and replicates the histological features of high-grade gliomas^28^.

**Fig. 1:**
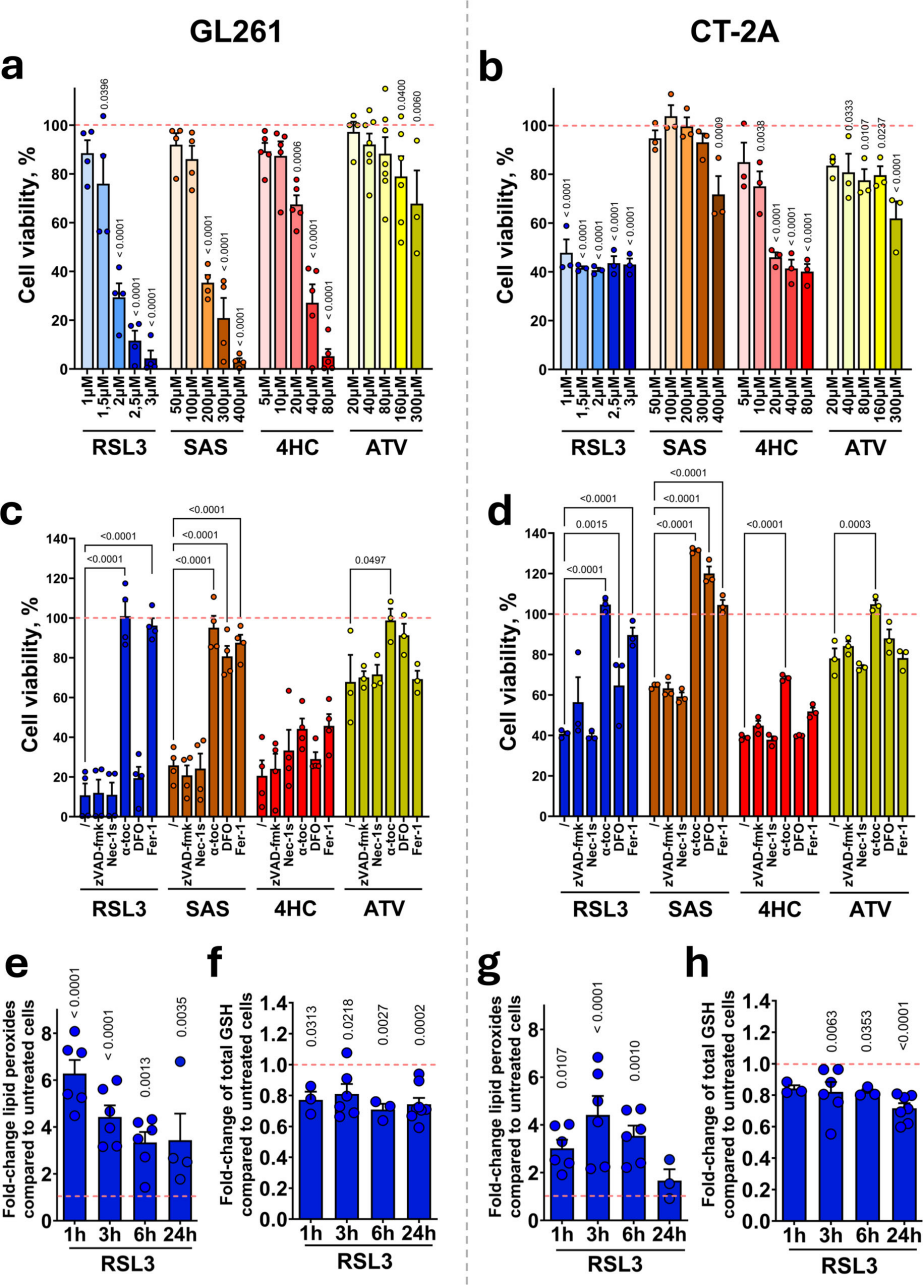
Induction of ferroptotic cell death in highly immunogenic murine glioma GL261 cells and weakly immunogenic CT-2A cells. **a–d** Cell death was induced by RSL3 (1–3 μM), SAS (50–400 μM), 4HC (5–80 μM) or ATV (20–300 μM) for 24 h and analyzed using MTS assay. RSL3, SAS, and 4HC efficiently induced cell death in GL261 cells (**a**), but in CT-2A cells, only RSL3 and 4HC were effective (**b**). The rate of cell death was significantly reduced in GL261 cells (**c**) and CT-2A cells (**d**) treated with 2.5 μM RSL3, 300 μM and 400 μM SAS (GL261 and CT-2A respectively), 40 μM and 80 μM 4HC (GL261 and CT-2A respectively) or 300 μM ATV by ferroptosis inhibitors (DFO, 10 µM; Fer-1, 1 µM; or α-toc, 50 µM) but not by inhibitors of apoptosis (zVAD-fmk 50 µM) and necroptosis (Nec-1s, 20 µM). **e**,**g** Generation of lipid ROS (lipid peroxides) during ferroptosis was assessed after treating glioma cells with RSL3 (2.5 μM) for 1, 3, 6 or 24 h and staining with BODIPY C11. Data are shown as fold change relative to untreated cells. RSL3 treatment of GL261 and CT-2A cells led to a significant increase in lipid peroxidation levels during the early stages of ferroptosis compared to untreated cells shown as dotted line. **f**,**h** Glutathione (GSH) levels were analyzed in lysates of GL261 and CT-2A cells treated for 24 h RSL3 (2.5 μM) for 1, 3, 6 or 24 h. RSL3 significantly reduced GSH levels in both glioma cell lines. Data are shown as fold change relative to untreated cells shown as dotted line. The values are expressed as means ± SEM, derived from 3 (1a – ATV 300 μM, 1b, 1c – ATV, 1 d, 1 f – RSL3 1 and 6 h, 1 g – RSL3 24 h, 1 h – RSL3 1 and 6 h), 4 (1a – RSL3, SAS, 1c RSL3, SAS, 4HC, 1e – RSL3 24 h), 5 (1a – 4HC), 6 (1a – ATV 160 μM, 1e – RSL3 1, 3 and 6 h, 1 f – RSL3 3 h, 1 g – control, RSL3 1, 3 and 6 h, 1 g – RSL3 3 h), 7 (1a – ATV 4 and 80 μM, 1e – control, 1 g – control and RSL3 24 h) or 8 (1 f – control, RSL3 24 h) independent biological experiments, each experiment contained 3 technical replicates. Statistical significance was determined using a one-way ANOVA followed by Dunnett multiple comparisons test or Kruskal-Wallis test with Dunn’s multiple comparisons correction. Source data are provided as a Source Data file.

To identify the most effective ferroptosis inducers for GL261 and CT-2A glioma cells, we screened several ferroptosis inducers with different mechanisms of action. These included RSL3 (GPX4 inactivation), 4-hydroperoxycyclophosphamide (4HC, which increases the labile iron pool), atorvastatin (ATV, which suppresses the intracellular antioxidant system), and sulfasalazine (SAS, which inhibits the cystine-glutamate transporter). The cells were treated with different concentrations of these compounds for 24 h (Fig. 1a, b) or 48 h (Supplementary Fig. 1a-b). RSL3 and 4HC rapidly induced cell death in both the highly immunogenic GL261 as well as the poorly immunogenic CT-2A murine glioma cell lines. SAS was only effective in GL261 cells, inducing death in over 90% of the cells at a concentration of 400 μM (Fig. 1a). In contrast, SAS had minimal effect on CT-2A cells, with only 28% of cells dead after 24 h at a concentration of 400 μM (Fig. 1b). On the other hand, ATV did not efficiently induce cell death in either cell line; while some cell death was observed, 60% of the cells remained viable after 24 h of treatment.

Next, to confirm the type of cell death, specific inhibitors of cell death were used to block apoptosis (zVAD-fmk), necroptosis (Nec-1s) or ferroptosis (Fer-1, DFO, α-toc). In GL261 cells, RSL3-induced cell death was significantly inhibited by the ferroptosis inhibitors Fer-1 and α-toc (Fig. 1c), whereas in CT-2A cells, RSL3-induced cell death was blocked by the ferroptosis inhibitors Fer-1, DFO, and α-toc (Fig. 1d). Additionally, cell death induced by SAS was significantly inhibited by all ferroptosis inhibitors in both glioma cell lines (Fig. 1c, d). Furthermore, α-toc significantly inhibited ATV-induced cell death in GL261 cells, and the cell death induced by both 4HC and ATV in CT-2A cells. However, 4HC and ATV did not efficiently induce cell death, and cell viability was not fully restored by ferroptosis inhibitors (Fig. 1c, d). Notably, the pan-caspase inhibitor zVAD-fmk and the RIPK-1 inhibitor Nec-1s were ineffective in blocking cell death, thereby excluding apoptosis and necroptosis, respectively. The addition of DMSO or cell death inhibitors in the absence of cell death inducers, did not result in significant changes in viability of cells (Supplementary Fig. 1c, d).

To further confirm induction of ferroptosis in GL261 and CT-2A glioma cell lines, lipid peroxidation and glutathione (GSH) levels were selected as primary markers of ferroptosis^29^. The oxidation of polyunsaturated fatty acids by lipoxygenases during ferroptosis results in the accumulation of peroxides, which then generate lipid peroxide breakdown products. After treatment with RSL3, both glioma GL261 (Fig. 1e) and CT-2A cells (Fig. 1g) showed a significant increase in lipid peroxidation levels compared to untreated cells in the early stages of ferroptosis. Notably, the peak of lipid peroxidation in GL261 cells occurred 1 h after cell death induction. In contrast, in CT-2A cells, the peak of lipid peroxidation was observed by 3 h, followed by a decrease by 6 h and the levels remained elevated. This suggests that the duration and intensity of lipid peroxidation during ferroptosis may differ between glioma cell lines.

Glutathione (GSH) functions as a reducing cofactor, enabling glutathione peroxidase to convert reactive phospholipid hydroperoxides into non-reactive and non-lethal phospholipid alcohols, thereby protecting cells from ferroptosis^30^. In glioma cells treated with RSL3 a significant decrease in GSH levels was observed after 1 h in GL261 cells and after 3 h in CT-2A cells (Fig. 1f, h). In addition, SAS induced a significant increase in lipid peroxidation levels in GL261 cells at 6 h after the treatment and decreased glutathione levels starting from 3 hours (Supplementary Fig. 1e, g). Of note, MTX does not significantly affect lipid peroxidation in either GL261 or CT-2A cells (Supplementary Fig. 1e–h).

Taken together, these data indicate that RSL3 and SAS efficiently induce ferroptosis in the highly immunogenic GL261, whereas in the weakly immunogenic^31,32^ CT2-A cells, only RSL3 is effective.

### Ferroptosis-based DC vaccines induce significant protective immunity against glioma

**Fig. 2:**
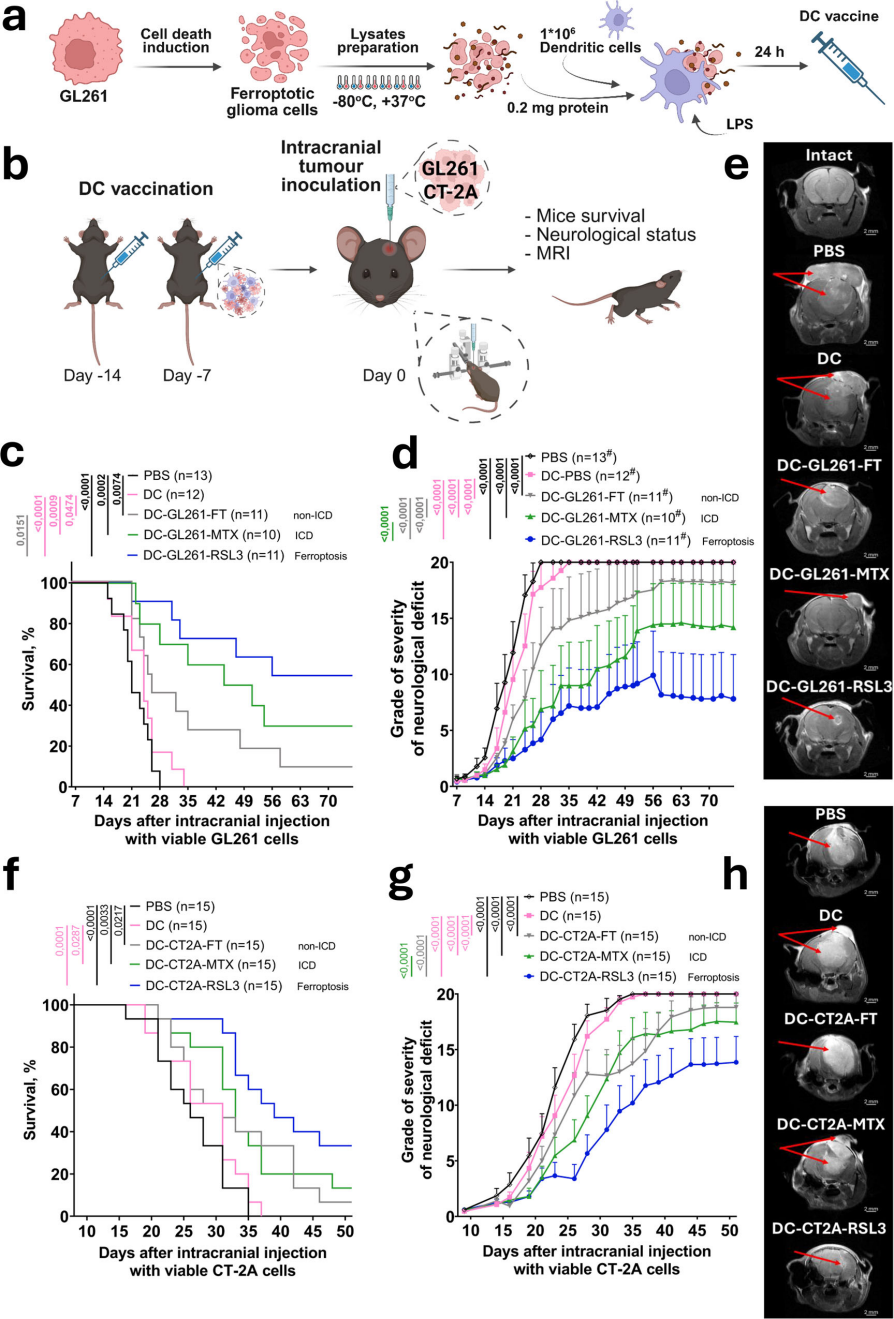
Ferroptosis-armed DC vaccines induce significant protective immunity against glioma. **a** Procedure for generating ferroptosis-armed DC vaccines from C57BL/6 J bone marrow-derived DCs. In glioma cells ferroptosis was induced by RSL3 (2.5 µM, 24 h). **b** Overview of the experimental setup to assess the effectiveness of DC vaccines in a prophylactic context using an orthotopic intracranial mouse model. Mice were administered 1*10^6^ DC vaccines’ cells on days −14 and −7, followed by challenge with an intracranial injection of viable glioma GL261 (2*10^4^ cells) or CT-2A (5*10^5^ cells) cells on day 0. Survival outcomes, neurological status and MRI scans were monitored thereafter. Created in BioRender. Krysko, D. (2026) https://BioRender.com/bqfgr9k. ***Prophylactic vaccination with DC-GL261-RSL3*** Mouse survival (**c**), neurological status (**d**) and MRI (**e**) were assessed after injection of the ferroptosis-armed DC vaccine, followed by intracranial challenge with viable glioma GL261 cells. Mouse survival and neurological status were assessed for up to 76 days. Mice were treated with DC vaccines loaded with glioma GL261 lysate treated with RSL3 (2.5 mM, DC-GL261-RSL3, *n* = 11). Control groups included mice injected with DC vaccines loaded with glioma GL261 lysate subjected to F/T cycles (negative control, non-ICD, DC-GL261-FT, *n* = 11) or treated with MTX (2.5 μM, positive control-ICD, DC-GL261-MTX, *n* = 10), and mice injected with unloaded DCs (negative control, DCs, *n* = 12) or with PBS (negative control, *n* = 13). ^#^On day 23, three mice per group underwent MRI scans. In some of these mice (2 in the PBS group and 2 in the DC group), the glioma size had already reached humane endpoints, and they were euthanized following the MRI. These mice were included in the analysis of survival and neurological status. However, other mice (1 in the PBS and DC groups and 3 in each of the DC-GL261-FT, DC-GL261-MTX, and DC-GL261-RSL3 groups) did not reach humane endpoints and were euthanized after the MRI; these mice were excluded from the analysis. (**e**) Representative T1-weighted MRI images of coronal brain sections taken on day 23, *n* = 3 mice per group. Arrows point to tumors. ***Prophylactic vaccination with DC-CT2A-RSL3*** Mouse survival (**f**), neurological status (**g**), and MRI (**h**) were evaluated after injection of the ferroptosis-armed DC vaccines and intracranial challenge with viable glioma CT2A cells. Mouse survival and neurological status were monitored for up to 51 days. Mice were treated with DC vaccines loaded with glioma CT2A lysate treated with RSL3 (2.5 mM, DC-CT2A-RSL3, *n* = 15). Control groups included mice injected with DC vaccines loaded with glioma CT-2A lysates subjected to F/T cycles (negative control, non-ICD, DC-CT2A-FT, *n* = 15) or treated with MTX (2.5 μM, positive control-ICD, DC-CT2A-MTX, *n* = 15), and mice injected with unloaded DCs (negative control, DCs, *n* = 15) or PBS (negative control, *n* = 15). **h** Representative T1-weighted MRI images of coronal brain sections taken on day 23, with the tumor indicated by arrows, *n* = 4 mice per group. Statistical analysis for mice survival (**c**,**f**) was determined by Mantel-Cox logarithmic test. Data of the neurological status of the mice (**d**,**g**) are shown as means ± SEM, statistical analysis was performed using a two-way ANOVA followed by Tukey’s multiple comparisons correction. Source data are provided as a Source Data file.

We examined whether glioma cells undergoing ferroptosis can induce anti-glioma protective immunity in a prophylactic setting using the orthotopic glioma mouse model. First, GL261 glioma cells were killed with RSL3, and ferroptotic lysates were prepared (Fig. 2a). The lysates were then loaded onto DCs for 24 h to generate the DC vaccines, which were tested for their ability to protect mice against an intracranial primary tumor challenge with viable glioma cells (Fig. 2b). This model is widely used to evaluate novel experimental anti-cancer treatments and assess the immunogenicity of dying glioma cells^31,33,34^. Immunocompetent syngeneic C57BL/6 mice were vaccinated twice intraperitoneally with DCs loaded ex vivo with ferroptotic glioma lysates (DC-GL261-RSL3). As a positive control, mice were vaccinated with DCs loaded ex vivo with mitoxantrone (MTX)-treated glioma GL261 cells (DC-GL261-MTX), as MTX is a well-known inducer of ICD^12^. However, the primary aim of this study was not to compare the efficacy between apoptotic and ferroptotic lysates, but rather to evaluate whether ferroptotic cell death is immunogenic and can trigger protective anti-tumor immunity. This question is particularly relevant in the context of tumor resistance to apoptosis, where ferroptosis could represent an alternative ICD modality. For the negative controls, we used PBS, unloaded DCs, or DCs loaded ex vivo with freeze-thawed (F/T, DC-GL261-FT) glioma GL261 cell. Notably, F/T does not induce ICD^13,19,35^. Subsequently, all mice were inoculated intracranially (intrathalamic) with 2*10⁴ live GL261 glioma cells and monitored for survival, the development of neurological deficit symptoms and pathological abnormalities by MRI (Fig. 2b).

Interestingly, mice vaccinated with DC-GL261-RSL3 before tumor challenge exhibited a significantly longer median survival compared to those injected with PBS (not reached *versus* 21 days, *p* = <0.0001), unloaded DC (not reached *versus* 24 days, *p* = <0.0001), or DCs loaded with DC-GL261-FT (not reached *versus* 26 days, *p* = 0.0151) (Fig. 2c). Importantly, 55% of the mice treated with DC-GL261-RSL3 remained tumor-free. Consistent with overall survival results, monitoring of glioma-induced neurological deficit grades revealed reduced severity of clinical symptoms and delayed progression of neurological deficits in mice vaccinated with DC-GL261-RSL3 (Fig. 2d). To further support these findings, we used MRI to non-invasively monitor the tumor growth in the same pool of mice (Fig. 2e). In the control groups, glioma formation subtly altered ventricular morphology and deformed the cerebral cortex. In contrast, most mice vaccinated with the DC-GL261-RSL3 vaccine showed minimal glioma masses at the inoculation site and better-preserved brain morphology. Notably, the efficacy of DC vaccines loaded with lysates prepared 3 hours after the induction of ferroptosis were not statistically different from those loaded with untreated lysates (DC-GL261-FT,. Supplementary Fig. 2a–c). At this time point, the ferroptotic process was not fully developed (Supplementary Fig. 2d). This finding further underscores the essential role of complete ferroptotic cell death in the protective effects of the DC vaccine.

Importantly, the GL261 glioma cell line is considered to be a highly immunogenic tumor model. To investigate further, we used the weakly immunogenic CT-2A glioma cell line^31,32^, a well-known immunotherapy-resistant model^26^, to generate ferroptotic lysates (Fig. 2a) and generate DC vaccines. These vaccines were used in a prophylactic vaccination protocol (Fig. 2b) similar to that used for DC vaccines loaded with ferroptotic GL261 lysates. Despite the difference in intrinsic immunogenicity between these glioma cell lines, mice prophylactically vaccinated with DC vaccines loaded with ferroptotic lysates of the weakly immunogenic CT-2A glioma (DC-CT2A-RSL3) demonstrated higher median survival than negative control PBS (39 days *versus* 26 days, p = <0.0001) or unloaded DC (39 days *versus* 31 days, *p* = 0.0001) (Fig. 2f). Furthermore, the use of DC-CT-2A-RSL3 vaccines prophylactically delayed the onset of neurological deficits (Fig. 2g) and inhibited glioma progression, as evidenced by MRI images (Fig. 2h). Altogether, these results indicate that DC vaccines based on ferroptotic lysates significantly enhance mice survival, whereas DC vaccines loaded with F/T-induced necrotic lysates do not. This demonstrates that the immunogenicity of ferroptotic lysates is independent of the glioma type.

### DC vaccines loaded with ferroptotic lysates induce curative anti-tumor immunity and prime cytotoxic T cells ex vivo

**Fig. 3:**
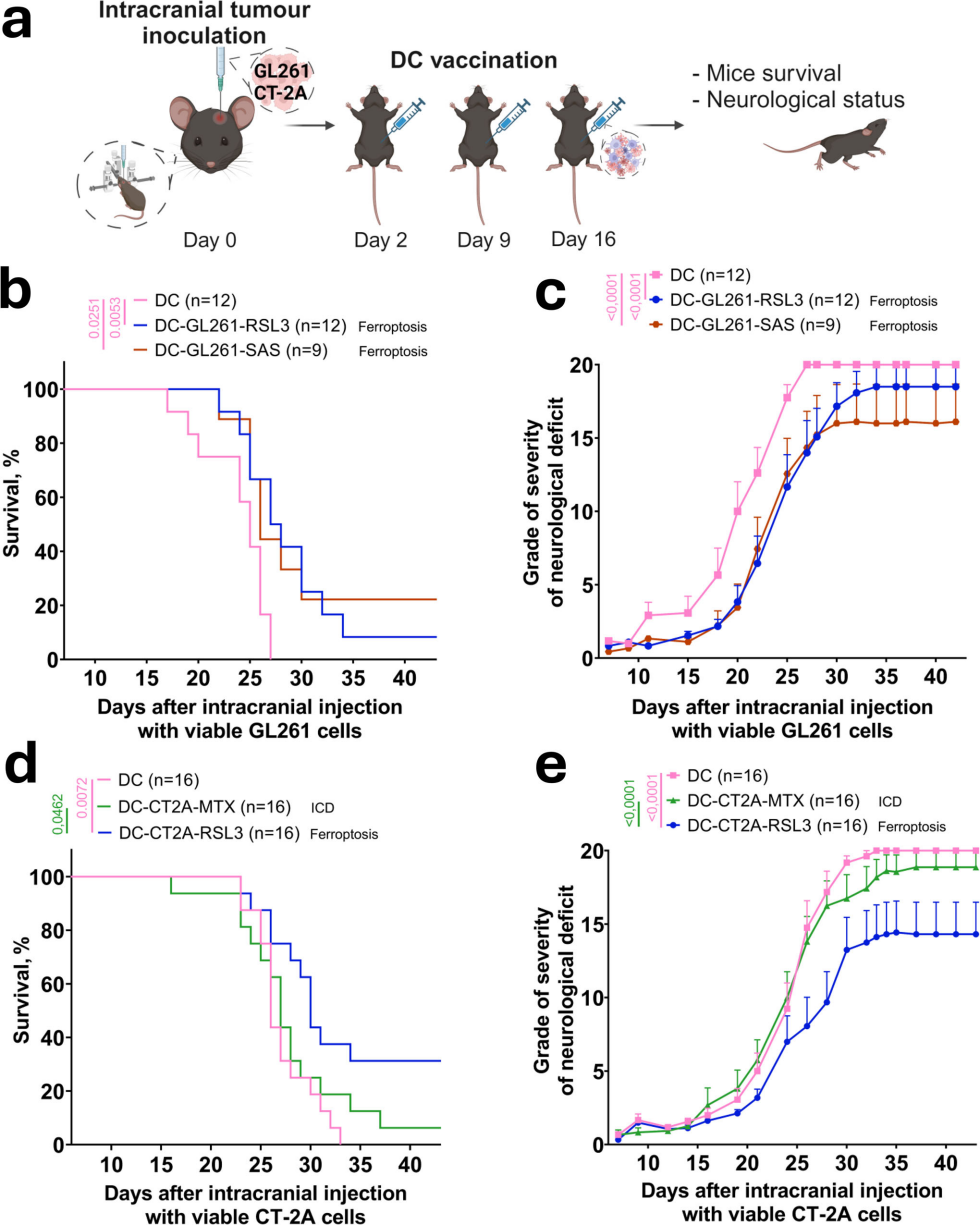
Therapeutic efficacy of ferroptosis-armed DC vaccines in orthotopic glioma models. **a** Procedure to assess the effectiveness of DC vaccines in a therapeutic setting using an orthotopic intracranial mouse model. On day 0, mice were injected intracranially with viable glioma GL261 or CT-2A cells, followed by administration of the corresponding DC vaccine on days 2, 9 and 16. Unloaded DCs were used as a control (negative control, DCs, *n* = 9). Created in BioRender. Krysko, D. (2026) https://BioRender.com/1z0ajr7. *Therapeutic vaccination with DC-GL261-RSL3* Mouse survival (b) and neurological status (c) after intracranial injection with viable glioma GL261 cells followed by therapy with ferroptosis-armed DC vaccines. Ferroptosis-armed DC vaccines were prepared using ferroptosis inducers with different mechanisms of action: RSL3 (2.5 μM, 24 h, DC-GL261-RSL3, *n* = 12) or SAS (300 μM, 24 h, DC-GL261-SAS, *n* = 9). Mouse survival and neurological status were monitored for up to 42 days. *Therapeutic vaccination with DC-CT2A-RSL3* Mouse survival (**d**) and neurological status (**e**) after intracranial injection with viable CT-2A cells followed by therapy with ferroptosis-armed DC vaccines. Mouse survival and neurological status were monitored for up to 55 days. Ferroptosis-armed DC vaccines were prepared with ferroptosis inducer RSL3 (2.5 μM, 24 h, DC-CT2A-RSL3, *n* = 16). As controls, we used mice injected with DC vaccines loaded with glioma GL261 lysates treated with MTX (2.5 μM, positive control-ICD, DC-CT2A-MTX, *n* = 16), or mice injected with unloaded DCs (negative control, DCs, *n* = 16). Statistical analysis for mice survival (**b**,**d**) was determined by Mantel-Cox logarithmic test. Data of the neurological status of the mice (**c**,**e**) are shown as means ± SEM. Statistical analysis was performed using a two-way ANOVA followed by Tukey’s multiple comparisons correction. Source data are provided as a Source Data file.

Although prophylactic vaccination is a valuable approach for investigating underlying molecular mechanisms and evaluating its ability to elicit an immune response, it is not feasible in a clinical setting, as patients typically require treatment of pre-existing tumors. Therefore, we assessed the efficacy of DC vaccines loaded with ferroptotic lysates (i.e., DC-GL261-RSL3) in a therapeutic orthotopic glioma mouse model (Fig. 3a). Unloaded DCs were included as a negative control. Three consecutive injections of the DC-GL261-RSL3 vaccine significantly extended survival in comparison to mice that received unloaded DCs (27.5 days for DC-GL261-RSL3 *versus* 25 days for unloaded DCs, *p* = 0.0251; Fig. 3b). Furthermore, the vaccine delayed the onset of neurological deficit symptoms (Fig. 3c) and provided protection against brain damage. Importantly, a DC vaccine based on glioma cell lysates killed by another ferroptosis inducer, SAS (DC-GL261-SAS), was also tested.

Importantly, two mice remained tumor-free for over 40 days following tumor inoculation. The vaccine demonstrated a comparable significant improvement in survival (Fig. 3b) and a reduction in neurological deficit progression (Fig. 3c) similar to the results observed with the DC-GL261-RSL3 vaccine.

Since GL261 is a highly immunogenic glioma cell line^36,37^, we also evaluated the DC vaccines therapeutically using the weakly immunogenic CT-2A glioma cells^26^. Again, mice therapeutically vaccinated with DC vaccines loaded with ferroptotic CT-2A lysates (DC-CT2A-RSL3) exhibited significantly longer median survival (30%) compared to negative controls, mice vaccinated with unloaded DCs (Fig. 3d, e). Interestingly, the survival of mice in the positive control group, which were vaccinated with DCs loaded ex vivo with lysates after MTX-treatment of glioma CT-2A cells (ICD inducer^12^, DC-CT2A-MTX), did not differ statistically from that of the control group vaccinated with unloaded DCs. This suggests that ferroptosis-armed DCs may have superior therapeutic potential. These findings demonstrate that the therapeutic effect of DC vaccines loaded with ferroptotic lysates is independent of the type of glioma or the specific ferroptosis inducer. It represents a general phenomenon of the ferroptotic cell death process and holds promise for application in clinically relevant glioma immunotherapy settings.

**Fig. 4:**
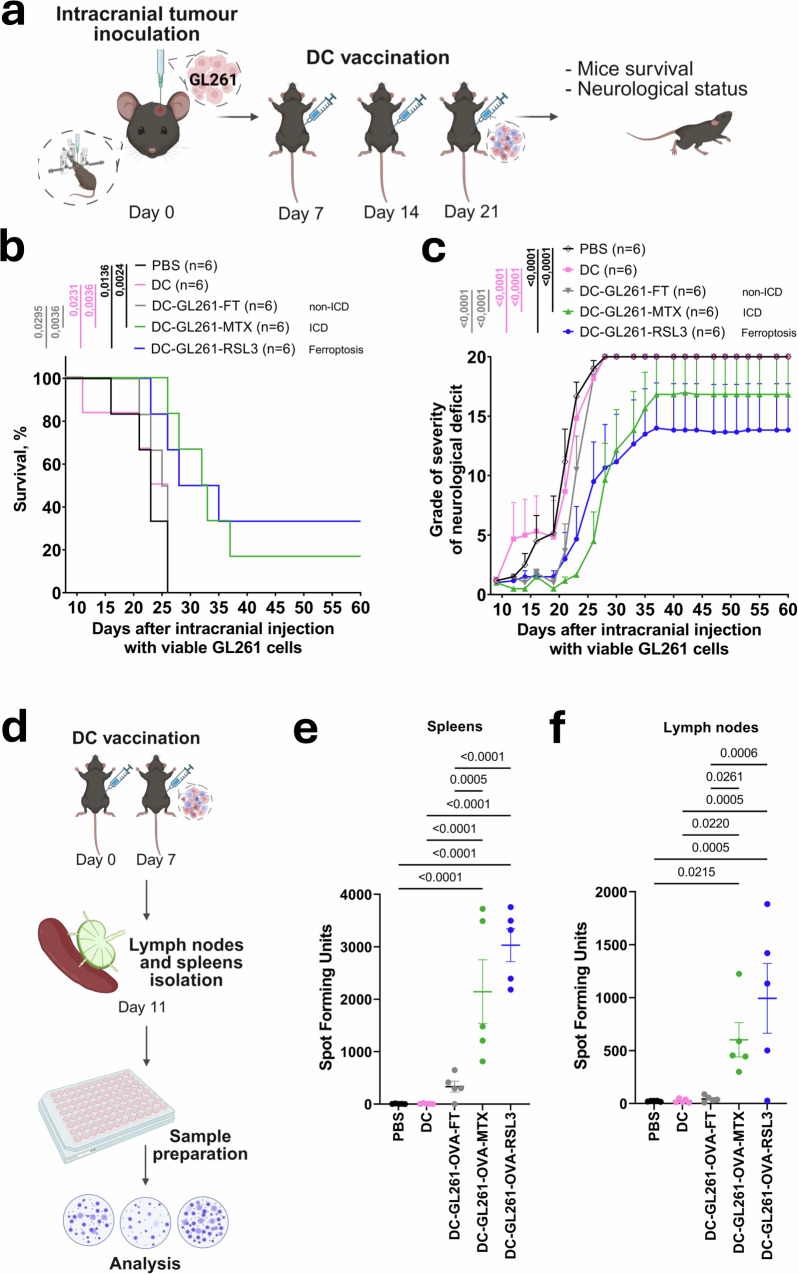
Ferroptosis-armed DC vaccines elicit therapeutic anti-tumor immunity and prime cytotoxic T cells ex vivo in a delayed treatment setting. **a** Procedure to assess the efficacy of DC vaccines in a more clinically relevant therapeutic setting using an orthotopic intracranial mouse model. Mice were injected intracranially with viable glioma GL261 cells on day 0, followed by administration of the corresponding DC vaccine on days 7, 14 and 21. Ferroptosis-armed DC vaccines were prepared with ferroptosis inducer RSL3 (2.5 μM, 24 h, DC-CT2A-RSL3, *n* = 6). Control groups included mice injected with DC vaccines loaded with glioma GL261 lysate subjected to F/T cycles (negative control: non-ICD, DC-GL261-FT, *n* = 6), DC vaccines loaded with MTX-treated lysates (2.5 μM, positive control: ICD, DC-GL261-MTX, *n* = 6), unloaded DCs (negative control, DCs, *n* = 6) or PBS (negative control, *n* = 6). Created in BioRender. Krysko, D. (2026) https://BioRender.com/lr8nwhi. Mouse survival (**b**) and neurological status (**c**) were assessed following intracranial injection with viable glioma GL261 cells and subsequent therapy with ferroptosis-armed DC vaccines. Survival and neurological status were monitored for up to 60 days. Mouse survival was analyzed using the Mantel–Cox log-rank test. Data of the neurological status of the mice are shown as means ± SEM, statistical analysis was performed using a two-way ANOVA followed by Tukey’s multiple comparisons correction. **d** Procedure to analyze the priming of cytotoxic T cells and IFN-γ production in mice immunized with DC vaccines on days 0 and 7. Four days after the final vaccination, the inguinal lymph nodes and splenocytes were isolated and re-stimulated ex vivo with the SIINFEKL peptide. Created in BioRender. Krysko, D. (2026) https://BioRender.com/lr8nwhi. **e**,**f** IFN-γ ELISpot assay of the spleens (**e**) and draining lymph nodes (**f**) from immunized mice. DC vaccines were loaded with ferroptotic GL261 lysates (DC-GL261-OVA-RSL3). Ferroptosis was induced by 2.5 μM RSL3. As controls, we used DC vaccines loaded with glioma GL261 cells subjected to F/T cycles (negative control: non-ICD, DC-GL261-OVA-FT), with MTX (2.5 μM, positive control: ICD, DC-GL261-OVA-MTX), unloaded DCs (negative control, DCs) or PBS (negative control). Each type of lysate was supplemented with 1 μM ovalbumin. The number of spot-forming units is presented as means ± SEM, *n* = 5. Statistical significance was determined using one-way ANOVA followed by Dunnett’s multiple comparisons test. Source data are provided as a Source Data file.

To further obtain clinically relevant data, we next performed a therapeutic vaccination experiment in which treatment was initiated 7 days after intracranial tumor inoculation, mimicking a more advanced disease setting (Fig. 4a). In this delayed treatment model, mice vaccinated with the ferroptosis-based DC vaccine (DC-GL261-RSL3) exhibited significantly prolonged survival compared to PBS-treated controls (31.5 days *versus* 23 days, *p* = 0.0136), unloaded DCs (31.5 days *versus* 24.5 days, *p* = 0.0231), and DCs loaded with freeze-thawed GL261 lysates (DC-GL261-FT) (31.5 days *versus* 25.5 days, *p* = 0.0295). Moreover, vaccination with DC-GL261-RSL3 delayed the onset and progression of neurological deficits in treated mice (Fig. 4b, c).

Importantly, despite the positive control DC-GL261-MTX-therapeutic vaccination was not as effective as the ferroptotic one, DC vaccines loaded with other two immunogenic apoptotic lysates (DC-GL261-DOX and DC-GL261-OXA) even if in a different extent, significantly improved mouse survival compared with vaccination with PBS or unloaded DCs, confirming the reliability of our data and demonstrating that the cell death inducers are the main driver of differential preclinical outcomes (Supplementary Fig. 3a–d).

To further examine the adaptive immune response elicited by intraperitoneal DC vaccines loaded with ferroptotic GL261 lysates, we measured cytokine release from cytotoxic T cells isolated from the draining inguinal lymph nodes and spleens of vaccinated mice (Fig. 4d). Vaccination with DC vaccines loaded with ferroptotic lysates (DC-GL261-OVA-RSL3) effectively primed immune cells for IFN-γ production in an ex vivo re-stimulation assay using the synthetic MHC-I-restricted OVA peptide (SIINFEKL). This response was observed in mice vaccinated with DCs loaded with ferroptotic lysates, similar to the response induced by immunogenic apoptotic lysates (DC-GL261-OVA-MTX), and was absent in control groups vaccinated with PBS, unloaded DCs, or DC vaccines based on non-ICD lysates (DC-GL261-OVA-FT) (Fig. 4e, f and Supplementary Fig. 2e).These findings support the conclusion that DC vaccines loaded with ferroptosis-induced lysates effectively prime cytotoxic T cells ex vivo and stimulate antigen-specific IFN-γ production.

### Ferroptotic lysates are intrinsically immunogenic and do not require bacterial adjuvants

**Fig. 5:**
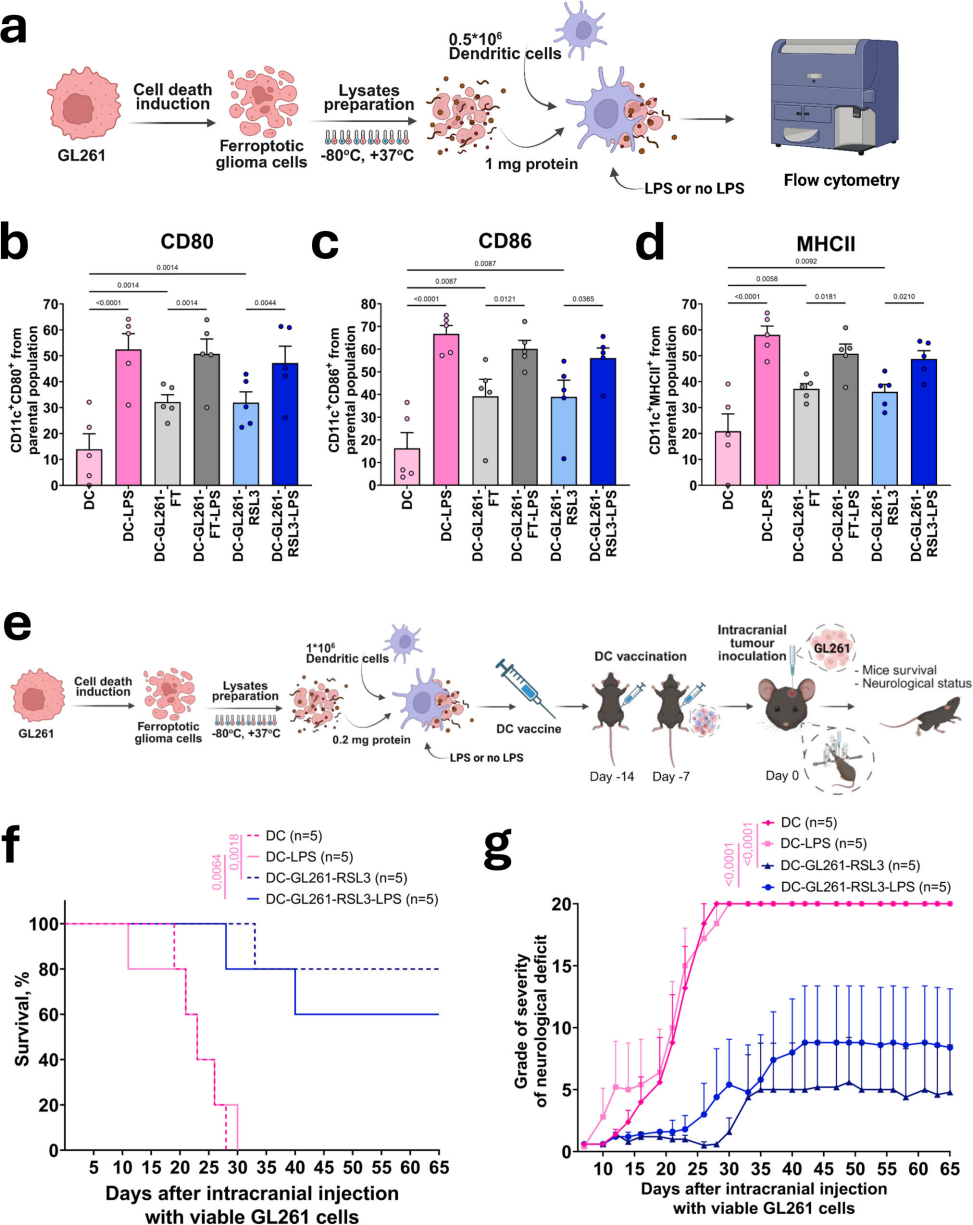
Ferroptosis-armed DC vaccine is intrinsically immunogenic and does not require bacterial adjuvants. **a** Procedure for studying DC maturation after co-culture with respective lysates. DCs were co-cultured with ferroptotic GL261 lysates (2.5 μM, 24 h, DC-GL261-RSL3) or F/T GL261 lysates (non-ICD, negative control, DC-GL261-FT) for 90 minutes, and then treated with lipopolysaccharide (LPS, 0.5 μg/ml) or left untreated for 24 hours. Unloaded DCs stimulated with LPS served as a positive control (DC-LPS), while untreated (DCs) served as a negative control. Created in BioRender. Krysko, D. (2026) https://BioRender.com/u1ngqru. **b–d** The percentages of viable CD11c^+^CD80^+^ (**b**), CD11c^+^CD86^+^ (**c**), and CD11c^+^MHCII^+^ (**d**) DCs are shown as mean values ± SEM from 5 independent experiments. Two-way ANOVA was used to assess the statistical significance of the results, followed by Hom-Sidac multiple comparisons test. **e** Procedure for generating ferroptosis-armed DC vaccines from wild-type (WT) mouse bone marrow-derived DCs (WT-DCs). WT-DCs were co-cultured for 90 min with ferroptotic GL261 lysates (RSL3, 2.5 μM, 24 h), and then further incubated with the same lysates in the presence (DC-GL261-RSL3-LPS) or absence (DC-GL261-RSL3) of 0.5 μg/mL LPS for 24 h. WT mice received the respective DC vaccines on days –14 and –7, followed by intracranial injection of viable GL261 glioma cells on day 0. As controls, mice were treated with unloaded DCs, either LPS-stimulated (DC-LPS) or unstimulated (DC). Created in BioRender. Krysko, D. (2026) https://BioRender.com/u1ngqru. **f**,**g** Mouse survival (**f**) and neurological status (**g**) were monitored up to day 65. Survival data were analyzed using the Mantel-Cox log-rank test. Neurological status was analyzed using two-way ANOVA followed by Tukey’s multiple comparisons test. Data are shown as means ± SEM, *n* = 5. Source data are provided as a Source Data file.

To further explore the mechanisms underlying the immunogenicity of ferroptotic lysates, we analyzed the activation markers on CD11c^+^ DCs following co-culture with glioma lysates (Fig. 5a–d and Supplementary Fig. 5). Co-culturing DCs with ferroptotic lysates (DC-GL261-RSL3) or with untreated lysates (DC-GL261-FT) induced the expression of CD80, CD86, and MHCII.

Interestingly, when lipopolysaccharide (LPS), a TLR4 ligand, was added to the co-cultures with ferroptotic lysates (DC-GL261-RSL3), the mean percentage of CD11c^+^ DCs expressing CD80, CD86, and MHCII significantly increased by 3.7-, 4.1-, and 2.8-folds, respectively (Fig. 5b–d). Similarly, LPS also significantly increased the expression of CD80, CD86, and MHCII in control DCs co-cultured with unstimulated lysates (DC-GL261-FT) compared to DC alone.

Importantly, in co-culture experiments, no differences were observed in activation markers between ferroptotic lysates (DC-GL261-RSL3) on one hand, and untreated lysates (DC-GL261-FT) in the presence of LPS on the other (Fig. 5a–d). As, however, prolonged survival was observed in mice following DC-GL261-RSL3 vaccination (Fig. 2), these findings suggests that the immunogenicity of glioma lysates is specifically influenced by the ferroptotic cell death process, which could not be identified in the co-culture assays with DCs.

Additional adjuvants are often incorporated in the tumor cell lysate-based DC vaccines to enhance DC activation, maturation, and overall vaccine efficacy^31,38–40^. LPS is frequently used in the preparation of such experimental vaccines^31,33^. To determine whether DC vaccine loaded with ferroptotic lysates are inherently immunogenic without the addition of the bacterial adjuvant LPS, we performed a vaccination experiment with DCs vaccines stimulated or not stimulated with LPS (Fig. 5e). The efficacy of DC vaccines loaded with ferroptotic lysates was not influenced by LPS supplementation, and unloaded DCs remained ineffective, irrespective of LPS addition (Fig. 5f, g).

Moreover, to minimize the potential agonistic contaminant confounders, we used DCs derived from TLR4 knockout mice, in an analogous experimental setup^41^. We generated DC-TLR4-KO vaccines with or without LPS (Supplementary Fig. 4a) and assessed their efficacy in an orthotopic intracranial mouse model (Supplementary Fig. 4b). Kaplan-Meier survival analysis of mice challenged intracranially with viable GL261 cells revealed that LPS did not impact the efficacy of the DC vaccine. Both groups of mice (with or without LPS) exhibited comparable survival rates (Supplementary Fig. 4c, e). DCs without ferroptotic lysates, regardless of LPS supplementation, were ineffective (Supplementary Fig. 4c, e). These findings suggest that ferroptosis-induced lysates are sufficient to drive DC-mediated anti-tumor immunity without the need for additional bacterial adjuvants

Furthermore, surviving mice (Supplementary Fig. 4c) were re-challenged 56 days after treatment with respective DC vaccines (with or without LPS) by intracranial injection of GL261 cell line (Supplementary Fig. 4b’). Both groups of mice (with or without LPS) exhibited comparable survival rates after the re-challenge with viable GL261 cells (Supplementary Fig. 4d, f). Non-invasive MRI monitoring (Supplementary Fig. 4g) revealed that mice re-challenged with viable GL261 cells remained macroscopically tumor-free. This indicates that the DC vaccines induced a robust and durable immune memory response, effectively protecting against glioma recurrence. These findings underscore the potential of ferroptotic lysate-loaded DC vaccines to provide long-term immunity independently of LPS, reinforcing their promise as a therapeutic strategy for glioma treatment and highlighting the intrinsic immunogenic potential of ferroptosis.

### Ferroptotic DC vaccines enhance CD8^+^ T cell infiltration and activation in the glioma microenvironment

**Fig. 6:**
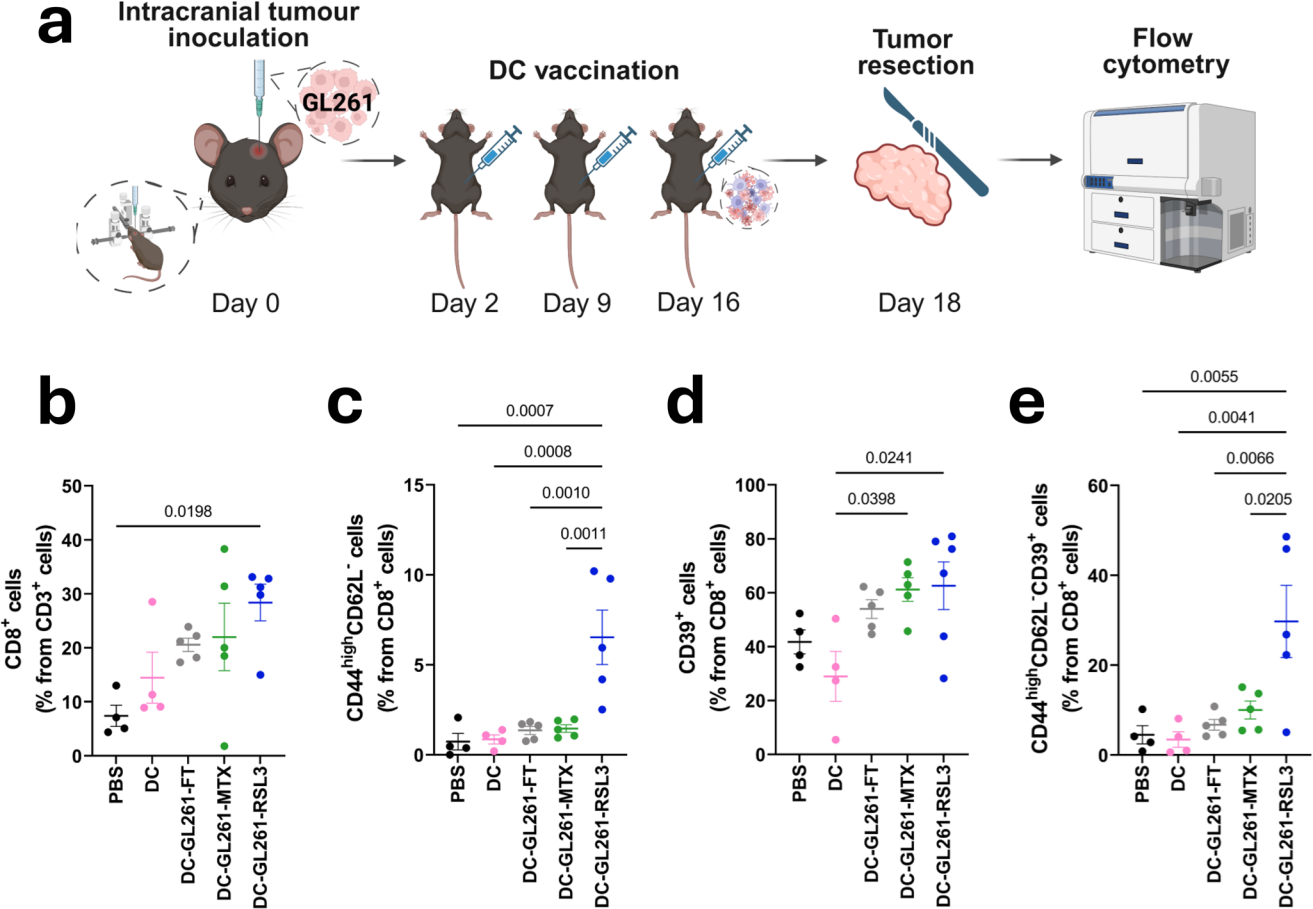
Ferroptotic DC vaccines enhance CD8^+^ T cell infiltration and activation in the glioma microenvironment. **a** Procedure to assess T-cell infiltration induced by DC vaccines in a therapeutic setting using an orthotopic intracranial mouse model. Mice were injected intracranially with viable glioma GL261 cells on day 0. Subsequently, they received the corresponding DC vaccine on days 2, 9 and 16. On day 18 after tumor inoculation, intracranial tumors were isolated, and immune cells were analyzed by multicolor flow cytometry. Control groups included mice treated with DC vaccines loaded with glioma GL261 lysate subjected to F/T cycles (negative control: non-ICD, DC-GL261-FT), DC vaccines loaded with MTX-treated lysates (2.5 μM, positive control: ICD, DC-GL261-MTX), unloaded DCs (negative control, DCs) or PBS (negative control). Created in BioRender. Krysko, D. (2026) https://BioRender.com/akwgjvw. **b–e** Percentage of CD8^+^ T cell infiltration. The ferroptosis-armed DC vaccine increased the infiltration of total CD8^+^ T cells (**b**), effector memory CD8^+^ T cells (CD44^+^CD62L^-^, **c** CD39^+^ exhausted CD8^+^ T cells (**d**) and CD39^+^ effector memory CD8^+^ T cells (**e**). The percentages of cells are shown as mean values ± SEM, *n* = 5. Statistical significance was determined using one-way ANOVA followed by Dunnett multiple comparisons test. Source data are provided as a Source Data file.

In order to analyze immune cell infiltration, the glioma microenvironment was investigated following DC vaccinations (Fig. 6a). Notably, the number of naïve (CD44^low^CD62L⁺), cytotoxic (GrB⁺), and central memory (CD44^high^CD62L⁺) CD8⁺ T-cells remained unchanged (Fig. 6b–e, Supplementary Fig. 6a–d and Fig S7). However, a significant increase in total CD8⁺ T-cells was observed in the mice vaccinated with DC loaded with ferroptotic lysates compared to those injected with PBS, unloaded DC, DC loaded with lysates from MTX-treated of glioma GL261 cells (positive control: ICD), or DC loaded with DC-GL261-FT (negative control: non-ICD) (Fig. 6b). In addition, a marked increase was detected in the number of CD44^high^CD62L^-^ effector memory CD8⁺ T cells and CD39⁺ exhausted CD8^+^ T-cells (Fig. 6d, e). Recent studies have shown that CD39⁺CD8⁺ T cells can play a beneficial role in the anti-tumor immune responses by contributing to tumor growth control and effectively killing autologous tumor cells^42,43^. Moreover, CD39⁺ T cells have been demonstrated to possess a tissue-resident memory T cell phenotype^44^.

Consequently, the co-expression of CD39⁺ T and memory markers CD44 and CD62L was examined, revealing a substantial increase in CD39⁺ effector memory CD8^+^T cells following administration of the ferroptosis-based DC vaccine (Fig. 6e). Furthermore, an increase in CD4⁺ T cells was observed after DC vaccination (Supplementary Fig. 6e), showing similar activation patterns (Supplementary Fig. 6f–j and Supplementary Fig. 7). No changes have been detected in tumor-infiltrating FoxP3⁺ regulatory T cells (Tregs, Supplementary Fig. 6k).

### Anti-tumor immunity induced by ferroptotic lysate-loaded DC vaccines depends on CRT and ATP signals, but not on HMGB1-TLR4

A defining feature of the immunogenicity of cancer cells undergoing ICD is their adjuvanticity, which is driven by the coordinated exposure or release of danger signals^10,45^. To ensure consistency with established mechanisms of ICD, we focused on the most canonical and mechanistically validated DAMPs (calreticulin (CRT), ATP, and HMGB1) which are widely accepted as critical mediators of adjuvanticity^10,11,46–48^.

**Fig. 7:**
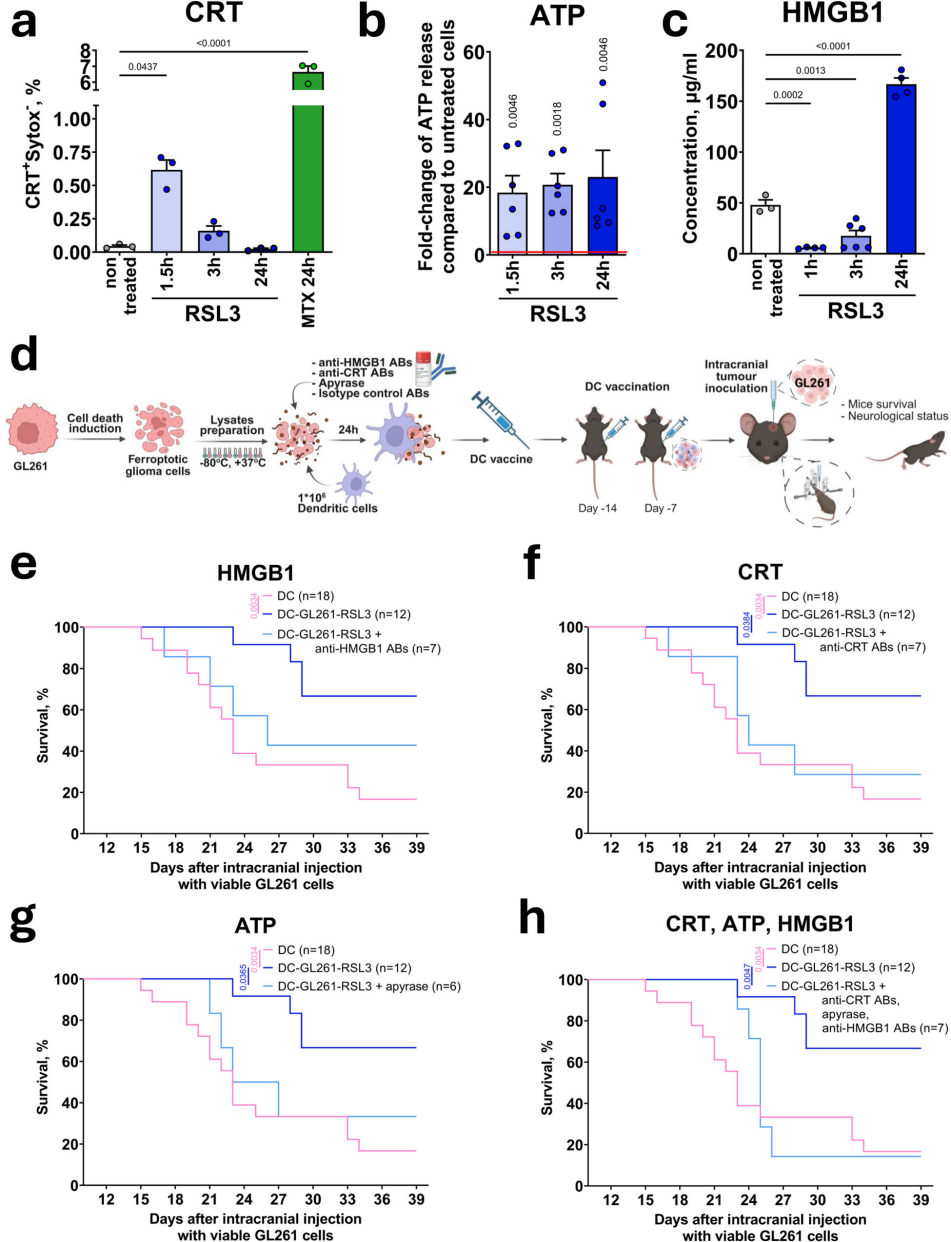
Anti-tumor immunity from ferroptosis-armed DC vaccines depends on CRT and ATP signals and is independent on HMGB1-TLR4. **a** Ferroptotic cell death is associated with CRT exposure on the cell surface. Flow cytometric analysis of CRT exposure on the cell surface of Sytox Green-negative GL261 cells treated with RSL3 (2.5 µM) for 1.5, 3, or 24 h or with MTX for 24 hours (2.5 µM, positive control-ICD). Time-dependent release of ATP (**b**) and HMGB1 (**c**) from ferroptotic glioma GL261 cells treated with RSL3 (2.5 µM) for 1.5, 3, and 24 h. Values are means ± SEM from 3 (a, c – non treated), 4 (c – RSL3 1,5 and 24 h) or 6 (b, c – RSL3 3 h) independent experiments. Statistical significance was calculated using one-way ANOVA followed by Dunnett multiple comparisons test. **d** Procedure for studying the efficacy of ferroptosis-armed DC vaccines in the presence of pharmacological inhibitors of DAMPs (HMGB1, CRT, and ATP) in an orthotopic intracranial mouse model. Ferroptosis was induced by RSL3 (2.5 µM, 24 h) in glioma GL261 cells. The ferroptotic glioma cells were then subjected to six freeze-thaw cycles to prepare ferroptotic lysates, which were incubated with anti-HMGB1 (DC-GL261-RSL3 + anti-HMGB1 Abs, *n* = 7), anti-CRT (DC-GL261-RSL3 + anti-CRT Abs, *n* = 7), apyrase (DC-GL261-RSL3 + apyrase, *n* = 6) or the combination of the three DAMPs (DC-GL261-RSL3 + anti-CRT Abs, apyrase, anti-HMGB1 Abs, *n* = 7) for 40 min. The respective DC vaccines were administered to mice on days −14 and −7. On day 0, the mice were challenged intracranially with viable GL261 glioma cells. Control groups included the ferroptotic DC vaccine without blockage (DC-CT2A-RSL3, *n* = 12) or unloaded DCs (DC, *n* = 18). Mouse survival and neurological status were monitored for up to 39 days. Created in BioRender. Krysko, D. (2026) https://BioRender.com/lmyfoee. **–** The curves depict the survival of mice over 39 days in the different groups, including pharmacological inhibition of HMGB1 (**e**), CRT (**f**), ATP (**g**), or the combination of the three DAMPs (**h**). As control, we used unloaded DCs (negative control, DCs) or ferroptotic lysates treated with an isotype control antibody (Supplementary Fig. 9a, b). Statistical significance was calculated using Mantel-Cox log-rank test. Source data are provided as a Source Data file.

We first examined CRT exposure during ferroptosis in GL261 cells treated with RSL3. CRT levels on the outer surface of the plasma membrane increased significantly at early time points (Fig. 7a and Supplementary Fig. 8). Interestingly, the patterns of ATP (Fig. 7b) and HMGB1 (Fig. 7c) release differed: ATP emission occurred early, while HMGB1 release peaked only after 24 h of stimulation with RSL3. The emission of these three key DAMPs may function as additional immunostimulatory adjuvants in DC vaccines.

To date, the impact of major ICD-associated DAMPs (CRT, ATP, and HMGB1) on the immunogenicity of ferroptotic tumor cells has not been comparatively tested. Therefore, we used well-established pharmacological strategies to block each DAMP^31^. GL261 glioma lysates were treated with anti-CRT^49^, anti-HMGB1 antibodies^50^, or with the ATP-hydrolyzing enzyme apyrase^13,51^ before generating DC vaccines for use in an orthotopic glioma model (Fig. 7d). Mice received two vaccine doses at one-week interval, and viable GL261 cells were injected intracranially one week after the second dose. We then analyzed the neurological status and mice survival.

Blocking HMGB1 (Fig. 7e and Supplementary Fig. 9c) in ferroptotic lysates slightly reduced the effectiveness of the DC vaccines, but this difference was not statistically significant (*p* = 0.256) compared to the DC vaccines loaded without HMGB1 blocking (DC-GL261-RSL3). Survival (Fig. 7e) was not significantly affected. Given that HMGB1 interacts with TLR4 on DCs to facilitate cross-priming of anti-tumor T lymphocytes and therefore is essential for ICD adjuvanticity^50,52^, we generated DCs from TLR4 knockout mice and loaded them with ferroptotic lysates (Fig. 4a, b). The absence of TLR4 had no measurable effect on mouse survival or neurological status (Fig. 4c, e). This excludes HMGB1 as a key danger signal in the immunogenicity of ferroptotic lysates. In contrast, blocking CRT (Fig. 7f) or ATP (Fig. 7g) significantly impaired the anti-tumor efficacy of the DC vaccine loaded with ferroptotic lysates and significantly increased neurological deficits (Supplementary Fig. 9d,e). Importantly, control isotype antibodies had no effect on survival or neurological status (Supplementary Fig. 9a, b). Combined inhibition of HMGB1, ATP, and CRT did not further diminish the immune response beyond the suppression observed with ATP or CRT blockade individually (Fig. 7h and Supplementary Fig. 9f), indicating that ATP and CRT are dominant DAMPs in mediating vaccine efficacy. Remarkably, despite this profound impairment, the simultaneous ablation of all three DAMPs still resulted in an improvement in median survival, with outcomes approaching those of the control group (Fig. 7h and Supplementary Fig. 9f). These findings suggest that the contribution of ferroptosis-induced adjuvanticity is primarily driven by ATP and CRT exposure, while HMGB1 plays a less dominant role in this context.

Interestingly, reconstitution of CRT and ATP into FT lysates did not significantly improve mouse survival but did delay the onset of neurological symptoms (Supplementary Fig. 10a–c), suggesting that this delay reflects a modest attenuation of tumor progression, indicative of a partial anti-tumor immune response.

Collectively, these results demonstrate that the immunogenicity of ferroptotic lysates and the efficacy of DC vaccines based on ferroptotic glioma cells can be influenced by blocking ATP and CRT, but not the HMGB1-TLR4 axis.

### Proteomic analysis and in silico immunopeptide prediction suggest unique antigenic determinants of ferroptosis

**Fig. 8:**
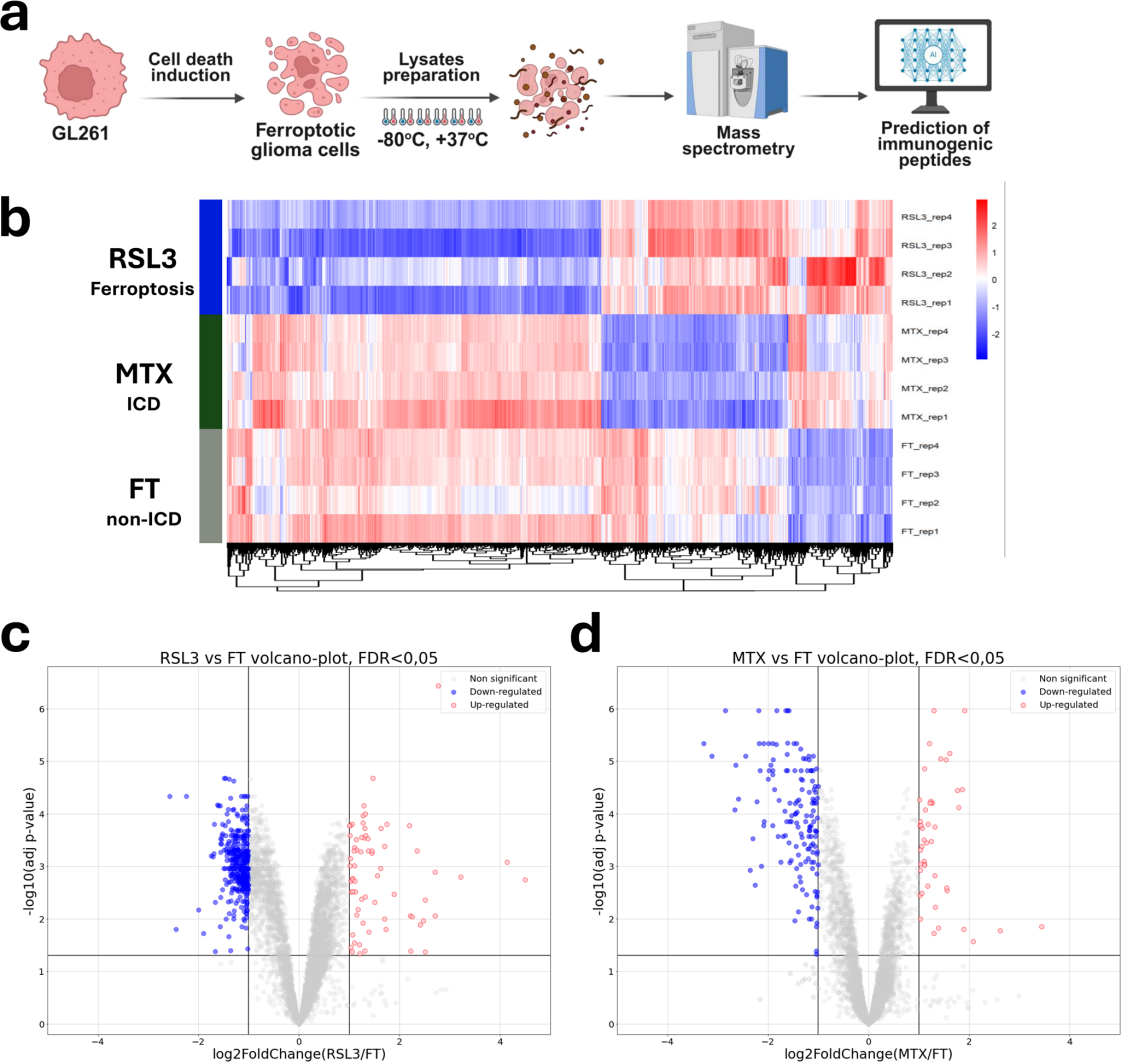
Proteomic profiling and in silico immunopeptide prediction suggest distinct tumor-associated antigens in ferroptotic lysates. **a** Ferroptosis and immunogenic apoptosis were induced in GL261 cells using RSL3 (2.5 μM, 24 h, *n* = 4) and MTX (2.5 μM, 24 h; ICD: positive control, *n* = 4), respectively. As a negative control, GL261 cells were subjected to F/T (non-ICD, *n* = 4). Proteomic analysis was then performed on the lysates used to load the DC vaccines. Created in BioRender. Krysko, D. (2026) https://BioRender.com/xok6a9n. **b** Proteins differentially abundant between groups (moderated t-test) were visualized in a heatmap following un-supervised hierarchical clustering of z-scored log_2_ reporter intensity values. **c**,**d** Differential protein expression analysis comparing RSL3 vs. F/T (**c**) and MTX *versus* F/T (**d**). Statistical analysis was performed using moderated t-test with statistical significance for differential regulation set at an FDR cut-off value of 0.05 and a |log2(fold change)| above 1. Source data are provided as a Source Data file.

A hallmark of ICD is antigenicity, driven by the release of tumor-specific antigens that facilitate the efficient and targeted induction of anti-tumor immunity through the interplay of antigenicity and adjuvanticity. Proteomic analysis was performed on untreated lysates (F/T, non-ICD), ferroptotic lysates (following RSL3 treatment), and lysates prepared after MTX-induced ICD (Fig. 8a). This analysis revealed significant alterations in protein expression profiles in all experimental groups, with ferroptotic lysates exhibiting a unique proteomic signature (Fig. 8b, Dataset S1, S2).

To assess differences in proteins intensities between ferroptotic lysates (RSL3) and FT lysates (negative control: non-ICD) (Fig. 8c), as well as between MTX-treated lysates (positive control: ICD, MTX) and FT lysates (Fig. 8d), statistical comparisons of group means were performed. We identified 55 up-regulated and 364 down-regulated proteins in ferroptotic lysates compared to the FT group (non-ICD), and 47 upregulated and 153 downregulated proteins in MTX-treated lysates (ICD) compared to FT lysates. Functional enrichment analysis revealed a downregulation of proteins involved in metabolic processes in ferroptotic lysates (Supplementary Fig. 11a) and an upregulation of proteins associated with mitotic processes in MTX-treated lysates (Supplementary Fig. 11b).

Next, we analyzed the peptides which can potentially be presented in the respective lysates derived from these proteins and predicted their immunogenicity in the context of murine MHC class I and II complexes. We identified 1,443 and 944 potentially MHC-I-presented peptides, as well as 981 and 583 potentially MHCII-presented peptides, in the ferroptotic and MTX-treated lysates, respectively. Notably, the ferroptotic and MTX-treated lysates shared 14 common upregulated proteins, giving rise to 289 potentially MHCI-presented peptides. Additionally, 13 commonly upregulated proteins between the two lysates contained 178 potentially MHCII-presented peptides (Supplementary Fig. 11c, Dataset S3, S4). This distinct profile in ferroptotic lysates likely introduces novel antigenic determinants^53^, fostering tumor-targeting immune responses and contributing to the observed immunogenic properties of ferroptotic lysates in the murine orthotopic glioma model.

## Discussion

We investigated the immunogenicity of ferroptotic cancer cells in DC-based glioma immunotherapy and demonstrated that DC vaccines primed with ferroptotic glioma lysates protect mice in a prophylactic setting against challenges with viable glioma cells in orthotopic glioma models, as well as in a therapeutic setting. Importantly, the immunogenicity of ferroptotic lysates was independent of type of the glioma cell line, the ferroptosis inducers, or the incorporation of additional bacterial adjuvants. We have also shown that blocking the key danger signals CRT and ATP but not the HMGB1-TLR4 axis significantly reduced the immunogenicity of ferroptotic glioma lysates and abolished the protective effect of DC vaccines in the orthotopic glioma model. In line with these findings, ferroptosis-based DC vaccination promoted substantial CD8⁺ T cell infiltration and effector memory differentiation within the glioma microenvironment, indicating the induction of a robust and functional anti-tumor immune response. This underscores the potential robustness of ferroptotic lysates in stimulating an effective immune response and highlights their potential broad applicability in developing DC-based cancer immunotherapies.

A promising approach in cancer immunotherapy focuses on the induction of ICD, which is defined by two critical features: (1) adjuvanticity, involving the release of DAMPs and cytokines/chemokines that activate anti-tumor immune responses, and (2) antigenicity, driven by the presence of tumor (neo)antigens in the dying tumor cells^8,54^. Both adjuvanticity and antigenicity are essential for eliciting robust anti-tumor immunity and establishing durable, life-long protection. Although ferroptosis is a recently identified regulated cell death modality distinguished from apoptosis and necroptosis by the iron-dependent accumulation of lipid peroxides^55^, its immunogenicity remains controversial^19,22^. Our results contribute to clarifying this debate by providing compelling evidence of the immunogenic potential of ferroptotic cancer cells, particularly in the context of DC-based glioma immunotherapy.

In our study, we first analyzed various ferroptosis inducers on two murine glioma cell lines (GL261 and CT-2A) and confirmed the occurrence of ferroptotic cell death. Among the inducers, RSL3 was selected for its widespread use in ferroptosis research and its demonstrated ability to induce both immunogenic^19^ and non-immunogenic^22^ ferroptotic cell death. Since ICD is associated with adjuvanticity, we further analyzed DAMPs release after ferroptosis induction with RSL3. Here, we showed that CRT exposure, a key feature of ICD^49^, occurs very early during the induction of ferroptosis (Fig. 7a). This finding contrasts with a previous report suggesting that CRT exposure in ferroptosis occurs shortly before cell membrane rupture^22^. However, the kinetic of CRT surface exposure is highly dependent on both cell type and the mode of cell death, underscoring the context-specific nature of ICD signaling. In most ICD settings, CRT exposure has been reported to precede phosphatidylserine externalization^56^. Accordingly, because ferroptosis induced by GPX4 inhibition, such as by RSL3, is characterized by rapid cell death kinetics, the early appearance of “eat-me” signals, including CRT and phosphatidylserine, is expected shortly after stimulation, as observed in our study. Nevertheless, differences in cell lines and death-inducing stimuli, including the type and concentration of the trigger, are likely to affect the timing of signal exposure, while not altering the overall execution of the process.

The emission of two other DAMPs, ATP and HMGB1 (Fig. 7b, c), aligns with prior findings in ferroptosis^19,22,57^ and also other regulated ICD modalities, such as apoptosis^49–51^ and necroptosis^13,14^. Altogether our results confirm that the emission of these DAMPs during ferroptosis underscores its immunogenic nature. In both prophylactic (Fig. 2) and therapeutic (Figs. 3, 4a–c) orthotopic mouse models, as well as in ex vivo re-stimulation assays (Fig. 4d–f), DC vaccines loaded with ferroptotic lysates demonstrated higher immunogenicity compared to those loaded with lysates after F/T (F/T-subjected cancer cells undergo accidental necrosis, a non-ICD form of unregulated cell death)^58^. This underscores the importance of the regulated nature of ferroptosis in its immunogenicity, consistent with previous reports indicating that regulated cell death modalities are more immunogenic than accidental necrosis^13,35,59,60^. Notably, several clinical studies have reported significant improvements in median overall survival of glioma patients using DC vaccines loaded with autologous tumor lysates prepared through non-ICD modalities^24,61^. In these studies, lysates were often generated using physicochemical methods such as freeze-thaw cycles, which induce accidental necrosis without activating regulated cell death pathways. However, there is no conclusive evidence that such lysates effectively prime antigen-specific T cell responses^13,35^.

To address this, we included a negative control group in our study in which viable glioma cells (GL261 or CT2A) were subjected to several freeze-thaw cycles, mimicking the preparation of lysates used in some clinical trials^62,63^. This allowed us to benchmark the immunogenicity of ferroptotic lysates (DC-GL261-RSL3) against lysates generated by accidental necrosis (DC-GL261-FT), providing a more clinically relevant comparison (Figs. 2, 4). It is intriguing to consider that DC vaccines loaded with lysates from ferroptotically killed glioma cells, which outperform lysates from glioma cells undergoing accidental necrosis in orthotopic mouse models, could offer a more effective immunotherapy.

It is known that distinct cell lines may exhibit different responses to cell death induction and therefore differ in their immunogenic potential^64^. Therefore, we used two different glioma models to generate DC vaccines: the highly immunogenic glioma GL261^65^ and the weakly immunogenic glioma, CT-2A^31,66^. DC vaccines based on ferroptotic lysates were protective in both prophylactic (Fig. 2) and therapeutic (Figs. 3, 4a-c) settings, regardless of the used glioma cell line or ferroptosis inducers. This highlights the inherent immunogenicity of drug-induced ferroptosis. These findings are consistent with a recently published report demonstrating ferroptosis immunogenicity after in situ photodynamic therapy^67^. These findings provide support for the concept that ferroptosis qualifies as an ICD modality^19,20^.

Recently, Wiernicki et al ^22^. reported that the immunogenicity of ferroptosis could be partially attributed to the presence of remaining viable cells within the ferroptosis-based vaccines, as observed in another tumor prophylactic vaccination model^19^. We addressed the issue of the presence of live cells by subjecting glioma cells to several cycles of F/T after ferroptosis induction (Fig. 2a). This effectively eliminated any remaining viable glioma cells from the DC vaccines to exclude their potential influence on the immunogenicity of ferroptosis in mice. The immunogenicity of ferroptotic lysates observed in this study strongly supports their potential for clinical translation, where such “devitalization” is required for safety and regulatory reasons and, in addition, the tumor tissue used in vaccine preparation must be preserved frozen^31,38^.

Incorporating bacterial components or cytokines/chemokines into immunotherapy protocols can enhance the efficacy of DC vaccines^40,68,69^. Notably, in an orthotopic intracranial prophylactic vaccination model, ferroptotic lysates demonstrated robust immunogenicity even in the absence of LPS (Fig. 5e–g and Fig S4). This effect was also seen when DC vaccines were generated from TLR4 knockout mice, underscoring the strong intrinsic immunogenicity of ferroptosis independent of potential agonistic contaminants^41^. Nevertheless, immune detection of cell death-associated danger involves multiple pattern recognition receptors and adapter pathways, including MyD88, TRIF, STING, and RIG-I. Therefore, while the absence of TLR4 dependence argues against LPS contamination specifically and excludes HMGB1 as a key mediator, it does not rule out the presence of other immunostimulatory molecules within the lysate that may contribute to DC activation and vaccine efficacy driven by ferroptotic lysates.

To further elucidate the mechanisms underlying ferroptosis immunogenicity and to analyze the role of key DAMPs (CRT, ATP, and HMGB1), we evaluated the effectiveness of DC vaccines in the orthotopic intracranial glioma mice model after pharmacologically blocking these molecules^13,31,50,51^ Our results revealed that CRT and ATP, rather than the HMGB1-TLR4 axis, are the primary ferroptosis-autonomous determinants driving the DC vaccine’s immunogenicity (Supplementary Fig. 4, Fig. 7e–h and Supplementary Fig. 9c–f). Importantly, simultaneous blockade of all three ICD-relevant danger signals was maximally suppressive to the in vivo immunogenicity of DC vaccine loaded with ferroptotic lysates (Fig. 7h and Supplementary Fig. 9f). These findings not only confirm the immunogenicity of ferroptosis but also expand the list of ferroptotic DAMPs responsible for its immunogenicity by adding CRT and ATP to the previously identified danger molecule decorin^20^ and various oxidized products, including oxygenated phosphatidylethanolamine^70^. However, we found that the reconstitution of CRT and ATP into FT lysates did not significantly improve mouse survival but did delay the onset of neurological symptoms (Supplementary Fig. 10a-c), suggesting that while individual DAMPs can partially enhance immune activation, solely they are insufficient to fully replicate the complex immunogenic profile induced by ferroptotic cell death^53^. The lack of a survival benefit implies that ferroptosis triggers additional immunogenic signals beyond CRT and ATP, which are essential for effective tumor clearance and durable immune protection. Indeed, although these pathways contribute to the overall immunogenicity of ferroptotic cells, future studies will be needed to clarify the contribution of additional DAMPs to ferroptosis-driven antitumor immunity, such as type I interferon and Annexin A1.

We demonstrated robust antigen presentation following vaccination with DC vaccines loaded with ferroptotic (RSL3) or apoptotic (MTX) tumor lysates (Fig. 4d, e), accompanied by increased tumor infiltration of CD8⁺ and CD4⁺ T cells (Fig. 6, Supplementary Fig. 6). Notably, ferroptosis-based vaccination was associated with a marked expansion of CD39⁺ effector memory T cells (Fig. 6e, Supplementary Fig. 6h). Elucidating the functional role and mechanistic contribution of this T cell subset in the context of ferroptosis driven DC vaccination is an intriguing direction for future investigation.

Our comparative proteomic and in silico immunopeptide prediction highlighted unique antigenic determinants of ferroptosis compared to both immunogenic apoptotic (MTX-treated) and non-immunogenic necrotic (FT) lysates (Fig. 8c, d). Interestingly, we identified a subset of proteins that were commonly upregulated in both ferroptotic and immunogenic apoptotic lysates, giving rise to MHC class I- and II- potentially presentable shared peptides. This overlap suggests the presence of a conserved ICD signature, potentially reflecting a shared mechanism of adaptive immune activation across different ICD modalities. At the same time, the broader divergence in antigenic profiles supports the notion that ferroptosis introduces a unique repertoire of tumor-associated antigens^53^. However, given the intrinsic limitations of current MHC-binding prediction algorithms, these analyses should be considered exploratory. The use of MHCnuggets, which combines a low false-positive rate with an estimated positive predictive value of ~60%, allows the identification of a conservative subset of putative binders. Validation of these predictions will require future immunopeptidomics-based approaches to more precisely define the antigenic landscape associated with ferroptosis. Collectively, these findings suggest that ferroptosis may partially recapitulate features of classical ICD while also generating a distinct repertoire of antigens. Such ferroptosis-associated antigens may represent a resource for the rational design of ferroptosis-based vaccination strategies, combinatorial immunotherapies, or biomarkers of effective ICD-associated immune responses.

In conclusion, though the immunogenicity of ferroptosis has been controversial, our study provides compelling evidence for the immunogenic properties of tumor cells undergoing ferroptosis and their protective and therapeutic potential in DC vaccines (Fig. graphical abstract). We demonstrate that DC vaccines based on lysates of ferroptotic glioma cells were protective in both prophylactic and therapeutic settings, regardless of the glioma cell line, ferroptosis inducer, or additional bacterial adjuvant. These findings underscore the strong intrinsic immunogenicity of ferroptotic lysates from drug-induced ferroptosis when used in combination with DCs. Furthermore, our study highlights the critical role of ferroptosis-associated DAMPs in driving the efficacy of potent DC vaccines based on ferroptotic lysates. Notably, although the tumor cells targeted by the immune system do not undergo ferroptosis themselves, the induction of ferroptosis prior to lysate generation may represent a key step for producing efficient immunogenic lysates and subsequent DC vaccines by altering antigenicity through lipid peroxidation-induced modifications of protein and lipid structures^53,71^, enhancing antigen uptake, promoting the exposure of cryptic epitopes, and facilitating more effective cross-priming of T cells against tumor-associated antigens present in the parental tumor proteome. In line with this, ferroptosis-armed DC vaccination markedly enhanced CD8⁺ T cell infiltration, activation, and effector memory differentiation within the glioma microenvironment, fostering robust tumor-targeting immune responses. Furthermore, our study highlights the critical role of ferroptosis-associated DAMPs in driving the efficacy of DC vaccines, reinforcing ferroptosis-based lysates as a promising platform for next-generation cancer immunotherapies.

## Methods

### Ethics statement

Female C57BL/6 J mice in C57BL/6 J background, aged 6-8 weeks, were housed in specific pathogen-free conditions. The TLR4 knock out mice carry the Tlr4^Lps-del^ spontaneous mutation corresponds to a 74,723 bp deletion that completely removes the *Tlr4 c*oding sequence, resulting in no mRNA or protein expression (B6.B10ScN-Tlr4 lps-del /JthJ, JAX strain #007227). This mutation leads to a lack of TLR4 expression on all cells.

The animals were housed in the IVC cages. Maximum 5 animals per cage were housed together. Food and water were offered ad libitum and nestlets were also provided. The cages were placed side by side in the same room and are manufactured in transparent plexiglass so that the animals still have some sensory contact with each other. The housing temperature was 20 °C–22 °C and the relative humidity 40–55%. The rooms had a controlled 12 h/12 h light/dark cycle.

The mouse experiments were conducted in accordance with the ethical guidelines of the local ethics committee of the Faculty of Medicine and Health Sciences of Ghent University (Belgium; ECD 22-12aan, ECD 23-73 and ECD 23-123) and the National Research Lobachevsky State University of Nizhny Novgorod (Russia).

Instead of tumor size, which cannot be measured in the current model, we used neurological status, physical behavior, and signs of discomfort as monitoring criteria, in accordance with the ethical protocol. These parameters were assessed regularly to minimize animal suffering and to determine humane endpoints. Mice were monitored daily for the behavioral signs of pain, including reluctance to move in the cage, abnormal posturing, social isolation, decreased grooming, aggression, delf-mutilation, piloerection, squinted eyes, pale eyes. If the abnormal behavioral changes are noted, mice were evaluated for the physiologic indicators of pain such as increased respiratory rate, changes in body temperature, and increased heart rate. If the signs of severe discomfort were noted mice will first received a subcutaneous injection of meloxicam (1 mg/kg). If no improvement was reached, humane killing was carried out to prevent or alleviate pain and/or distress. The level of neurological deficits in mice were measured after tumor inoculation. These tests include motor, sensory examination, assessment of coordination, reflexes and orienting activity. Furthermore, if the mice’s body temperature decreased to 34,5 °C or/and body weight loss more than 20% compared to pre-study or/and the level of neurological deficits (Tab. S1) was more than 15 and if the signs of severe discomfort are noted, mice will be immediately sacrificed by an i.p. injection of an overdose of sodium phenobarbital (Nembutal 150 mg/kg) with cervical dislocation.

Sample sizes for in vivo experiments were determined using G*Power 3.1.5 software. Everyone involved in the experiments and analyses of their outcomes was blinded.

### Cell lines

Murine glioma lines GL261, GL261-SIINFEKL, and CT-2A cells were cultured at 37 °C in a 5% CO₂ atmosphere in DMEM containing 4.5 g/L glucose (Cat. 11965092, Gibco, USA), supplemented with 2 mM glutamine (Cat. 25030081, Gibco, USA), 100 µM sodium pyruvate (Cat. 11360070, Gibco, USA), 100 units/ml penicillin/100 µg/L streptomycin (Cat. 15140122, Gibco, USA), and 10% fetal bovine serum (Cat. A5670701), all purchased from Gibco, USA. The GL261 cells were generously provided by Prof. P. Agostinis from the Laboratory of Cell Death Research & Therapy, Department of Cellular and Molecular Medicine, KU Leuven, Leuven, Belgium. The CT-2A cells (cat. SCC194) were procured from MERK KGaA (Germany). Cell lines were tested monthly for mycoplasma using the MycoAlert Mycoplasma Detection Kit (Cat. LT07-318, Lonza, USA). All samples were randomly allocated for the treatment. The investigators involved in the experiment were blinded.

### Cell death induction and analysis of cell death types

Cell death was induced using RAS-selective lethal (RSL3, CAS 1219810-16-8, Sigma-Aldrich, Germany), sulfasalazine (SAS, CAS 599-79-1, Sigma-Aldrich, Germany), 4-hydroperoxy cyclophosphamide (4HC, CAS 39800-16-3, Santa Cruz Biotechnology, USA), atorvastatin (ATV, CAS 344423-98-9, Sigma-Aldrich, Germany), mitoxantrone (MTX, CAS 70476-82-3, Sigma Aldrich, Germany), doxorubicin (DOX, CAS 23214-92-8, Selleckchem, USA) or Oxaliplatin (OXA, CAS 61825-94-3, MedChemExpress, USA).

Glioma cells were treated with RSL3 (1–3 μM), SAS (50–400 μM), 4HC (5–80 μM) and ATV (20–300 μM) and cultured for 24 or 48 h or with DOX (0,1–10 μM) or OXA (10–500 μM) and cultured for 24 hours. For MTX-induced cell death, cells were cultured with 2.5 µM MTX for 24 hours. Control cells were cultured under the same conditions without the addition of these agents.

To differentiate between various cell death modalities, the following cell death inhibitors were used: the pan-caspase inhibitor carbobenzoxy-valyl-alanyl-aspartyl-[O-methyl]-fluoromethylketone (zVAD-fmk, 50 μM, CAS 187389-52-2, Sigma-Aldrich, Germany), the RIPK1 inhibitor necrostatin-1s (Nec-1s, 20 μM, CAS 4311-88-0, Abcam, USA), the lipid peroxidation inhibitor α-Tocopherol (α-toc, 50 μM, CAS 59-02-9, Sigma-Aldrich, Germany), the inhibitor of ROS and lipid peroxidation, ferropstatin-1 (Fer-1, 1 μM, CAS 347174-05-4, Sigma-Aldrich, Germany), and the iron chelator deferoxamine (DFO, 10 μM, CAS 138-14-7, Sigma-Aldrich, Germany). These inhibitors were added 1 h before and simultaneously with the corresponding cell death-inducing reagent, and cells were incubated for 24 h. All cell death inducers and cell death inhibitors were dissolved in DMSO.

Cell viability was assessed using the MTS assay with the CellTiter 96 AQ_ueous_ One Solution Cell Proliferation Assay (Cat. G3580, Promega, USA) according to the manufacturer’s instructions. Optical density was measured at 490 nm using a Tecan Spark 20 M multimode microplate reader (Tecan Group, Switzerland). Flow cytometry analysis was conducted by staining the cells with Sytox Green (0.7 µM, Cat. S7020, ThermoFisher Scientific, USA) and Annexin V (5 µL/sample, Cat. A13199, ThermoFisher Scientific, USA) and analyzing them on an LSRFortessa (BD, USA) flow cytometer.

### Analysis of lipid ROS

To quantify lipid ROS, the fluorescent probe BODIPY 581/591 C11 (Cat. D3861, ThermoFisher Scientific, USA) was used. BODIPY 581/591 C11 exhibits intrinsic red fluorescence that shifts to the green channel upon interaction with peroxyl radicals due to a change in its chemical structure. Glioma GL261 or CT-2A cells were treated with RSL3 (2.5 µM), SAS (300 µM) or MTX (2.5 µM) for 1, 3, 6 or 24 hours to monitor lipid oxidative stress. After treatment, the cells were washed and incubated for 30 minutes at 37 °C in FBS-free medium containing 0.5 µM BODIPY 581/591 C11. Following incubation, cells were washed again, stained with Sytox Blue (3.3 µM, Cat. S34857, ThermoFisher Scientific, USA), and analyzed using an LSRFortessa flow cytometer (BD, USA).

Lipid peroxidase levels were assessed by calculating the ratio of the median fluorescence intensity in the green channel (FITC, oxidized lipids) to that in the red channel (PE, non-oxidized lipids). Only non-permeabilized cells were included in the analysis.

### GSH analysis

Glioma GL261 and CT-2A cells were seeded and treated with 2.5 µM RSL3, 300 µM SAS or 2.5 µM MTX for 1, 3, 6 or 24 h, after which they were collected, lysed using CelLytic™ MT Cell Lysis Reagent (Cat. C3228, Sigma-Aldrich, Germany), and centrifuged at 15,000 g for 15 min at 4 °C. Protein concentration in the supernatant was measured using the BCA Protein Assay Kit (Cat. 23227, ThermoFisher Scientific, USA), according to the manufacturer’s instructions.

For further analysis, 40 µL of the supernatant, containing 20–30 µg of protein, was mixed with 20 µL of water and 5 µL of 50% trichloroacetic acid (TCA, CAS 76-03-9, Sigma-Aldrich, Germany). The mixture was incubated on ice for 5 min, and centrifuged at 15,000 g for 5 min at 4 °C. In a 96-well plate, 100 µL of TRIS EDTA (pH 8.9; 0.2 M), 50 µL of the prepared solution, and 2.5 µL of 0.01 M 2,2′-dinitro-5,5′-dithiodibenzoic acid (CAS 69-78-3, Sigma-Aldrich, Germany) were combined. Absorbance was measured at 405 nm using a Tecan Spark 20 M multimode microplate reader (Tecan Group, Switzerland).

### Quantification of DAMPs emission

#### ATP release

Glioma GL261 cells were seeded, treated with 2.5 µM RSL3 as described previously, and incubated for 1.5, 3, 18, and 24 hours in medium containing 2% FBS. At each time point, supernatants were collected and centrifuged at 15,000 g for 3 minutes at 4 °C. The resulting supernatants were either stored at −80 °C or used immediately for ATP measurement. ATP levels were measured using the CellTiter-Glo Luminescent Cell Viability Assay Kit (Cat. G7570, Promega, USA) according to the manufacturer’s instructions. Luminescence was recorded on a Tecan Spark 20 M multimode microplate reader (Tecan Group, Switzerland).

#### HMGB1 release

Glioma GL261 cells were treated with 2.5 µM RSL3 for 1, 3, or 24 h. After treatment, supernatants were collected, cleared by centrifugation, and stored at −80 °C for subsequent HMGB1 analysis. HMGB1 levels were determined with an ELISA kit (Cat. 30164033, Tecan Group, Switzerland) following the manufacturers’ instructions. Measurements were made on a Tecan Spark 20 M multimode microplate reader (Tecan Group, Switzerland) and data analysis utilized a four-parameter logistic curve fit.

#### Calreticulin exposure on the outer surface of the plasma membrane

Glioma GL261 cells were seeded and treated with 2.5 µM RSL3 for 1.5, 3, 5, 18, and 25 hours. After treatment, cells were detached and stained with anti-calreticulin (CRT) antibodies (Cat. ab210431, Abcam, USA) or isotype control antibodies (Cat. ab172730, Abcam, USA), along with Sytox Green (0.7 µM, Cat. S7020, ThermoFisher Scientific, USA) for flow cytometry analysis (LSRFortessa, BD, USA), data analysis was conducted using FlowJo software (V.10.0.8) following the method described by Mishchenko et. al^34^. The gating strategy is shown in Supplementary Fig. 8.

### Generation of glioma lysates and proteomics analysis

Glioma GL261 or CT-2A cells were treated as described previously and incubated for 24 h. Following treatment with RSL3, SAS, MTX, or no treatment, cells underwent six cycles of freezing (at –80 °C) and thawing (at +37 °C). The total protein content in the glioma cell lysates was quantified using a BCA Protein Assay Kit (Cat. 23227, ThermoFisher Scientific, USA) and measured on a Tecan Spark 20 M multimode microplate reader (Tecan Group, Switzerland).

The proteome analysis was conducted at the VIB Ghent Proteomics Core. Sodium dodecyl sulfate (SDS) and triethylammonium bicarbonate (TEAB) was added to the sample to a final concentration of 5% SDS, 50 mM TEAB, pH 8.5. Then, the sample was transferred to a 96-well PIXUL plate and sonicated with a PIXUL Multisample sonicator (Active Motif) for 10 minutes with default settings (Pulse 50 cycles, PRF 1 kHz, Burst Rate 20 Hz). After centrifugation of the samples for 15 min at maximum speed at room temperature (RT) to remove insoluble components, from each sample 100 µg of protein was isolated to continue the protocol. Proteins were reduced and alkylated by addition of 10 mM Tris(2-carboxyethyl)phosphine hydrochloride and 40 mM chloroacetamide and incubation for 10 min at 95 °C in the dark. Phosphoric acid was added to a final concentration of 1.2% and subsequently samples were diluted 7-fold with binding buffer containing 90% methanol in 100 mM TEAB, pH 7.55. The samples were loaded on the 96-well S-Trap™ plate (Protifi), placed on top of a deepwell plate, and centrifuged for 2 min at 1,500 x g at RT. After protein binding, the S-trap™ plate was washed three times by adding 200 µl binding buffer and centrifugation for 2 min at 1,500 x g at RT. A new deepwell receiver plate was placed below the 96-well S-Trap™ plate and 50 mM TEAB containing 1 µg trypsin (1/100, w/w) was added for digestion overnight at 37 °C. Using centrifugation for 2 min at 1500 x g, peptides were eluted in three times, first with 80 µl 50 mM TEAB, then with 80 µl 0.2% formic acid (FA) in water and finally with 80 µl 0.2% FA in water/acetonitrile (ACN) (50/50, v/v). Eluted peptides were dried completely by vacuum centrifugation.

TMTpro^TM^ 16-plex labels (0.5 mg, Thermo Fisher Scientific) were equilibrated to RT immediately before use and dissolved in 20 µl anhydrous ACN. The dried peptides were re-suspended in 100 mM TEAB (pH 8.5), peptide concentration was determined on a Lunatic spectrophotometer (Unchained Labs)^72^ and peptide amount was adjusted to 40 µg in 30 µl for each sample. Peptides were labeled for 1 hour at RT using 0.5 mg of TMTPro^TM^ label (labels used: 126, 128 N, 131 C & 133 N: RSL3 replicates; 127 N, 130 C, 132 N & 133 C: MTX replicates; 127 C, 131 N, 132 C, 134 N: F/T replicates). The reaction was quenched for 15 min at RT by addition of 2 µl 5% hydroxylamine. The 12 labeled samples were combined, aliquoted in 100 µg aliquots, dried by vacuum centrifugation, re-dissolved in 100 µl loading solvent A (0.1% TFA in water/ACN (98:2, v/v)) and desalted on a reversed phase (RP) C18 OMIX tip (Agilent). The tip was first washed 3 times with 100 µl pre-wash buffer (0.1% TFA in water/ ACN (20:80, v/v)) and pre-equilibrated 5 times with 100 µl of wash buffer (0.1% TFA in water) before the sample was loaded on the tip. After peptide binding, the tip was washed 3 times with 100 µl of wash buffer and peptides were eluted twice with 100 µl elution buffer (0.1% TFA in water/ACN (40:60, v/v)). The combined elutions were dried in a vacuum concentrator.

Peptides were re-dissolved in 100 µl loading solvent A and 95 µl (+/− 100 µg) was injected for fractionation by RP-HPLC (Agilent series 1200) connected to a Probot fractionator (LC Packings). Peptides were first loaded in loading solvent A on a 4 cm pre-column (made in-house, 250 µm internal diameter (ID), 5 µm C18 beads, Dr. Maisch) for 10 min at 25 µl/min and then separated on a 15 cm analytical column (made in-house, 250 µm ID, 3 µm C18 beads, Dr Maisch). Elution was done using a linear gradient from 100% RP-HPLC solvent A (10 mM ammonium acetate (pH 5.5) in water/ACN (98:2, v/v)) to 100% RP-HPLC solvent B (70% ACN, 10 mM ammonium acetate (pH 5.5)) in 100 min at a constant flow rate of 3 µL/min. Fractions were collected every minute between 20 and 92 min and pooled every 24 minutes to generate a total of 24 samples for LC-MS/MS analysis. All 24 fractions were dried under vacuum in HPLC inserts and stored at −20 °C until further use.

#### LC-MS/MS analysis

Purified peptides from all fractions were re-dissolved in 20 µl solvent A and 15 µl of each fraction was injected for LC-MS/MS analysis on an Ultimate 3000 RSLCnano system in-line connected to an Orbitrap Fusion Lumos mass spectrometer (Thermo Fisher Scientific) equipped with a pneu-Nimbus dual ion source (Phoenix S&T). Trapping was performed at 20 μl/min for 2 min in solvent A on a 5 mm PepMap™ Neo trapping column (300 μm ID, 5 μm beads, C18, Thermo Fisher Scientific, p/n 174500) and the peptides were separated on a 250 mm Aurora Ultimate, 1.7 µm C18, 75 µm inner diameter (Ionopticks, AUR3-25075C18) kept at a constant temperature of 45 °C. Peptides were eluted by a non-linear increase from 0.5 to 26.4% MS solvent B (0.1% FA in water/ACN (2:8, v/v)) over 52,5 min, then to 44% in 66.5 minutes followed by a 5-minutes wash at 70 minutes reaching 56% MS solvent B and re-equilibration with MS solvent A (0.1% FA in water) all at a constant flowrate of 250 nl/min.

The mass spectrometer was operated in data-dependent mode with a top speed of three seconds. Full-scan MS spectra (375-1500 m/z) were acquired at a resolution of 120,000 in the Orbitrap analyzer after accumulation to a target AGC value of 400,000 with a maximum injection time of 50 ms. The precursor ions were filtered for charge states (2-7 required), dynamic exclusion (60 s; +/− 10 ppm window) and intensity (minimal intensity of 5E4). The precursor ions were selected in the quadrupole with an isolation window of 0.7 Da and accumulated to an AGC target of 1E4 or a maximum injection time of 50 ms and activated using CID fragmentation (35% NCE). The fragments were analyzed in the Ion Trap Analyzer at turbo scan rate. 10 most intense MS2 fragments were selected in the quadrupole using MS3 multi-notch isolation windows of 2 m/z. An orbitrap resolution of 60,000 was used with an AGC target of 1E5 or a maximum injection time of 118 ms and activated using HCD fragmentation (65% NCE). The polydimethylcyclosiloxane background ion at 445.120028 Da was used for internal calibration (lock mass). QCloud was used to control instrument longitudinal performance during the project^73^. Raw data were deposed in PRIDE^74^.

#### Data analysis

LC-MS/MS runs for all samples were processed together using the MaxQuant algorithm (version 2.2.0.0) with default search settings, including a false discovery rate of 1% at both the precursor and protein levels. Spectra were matched against mouse protein sequences from the Swiss-Prot database (release version 2023_01), containing 17,137 sequences (www.uniprot.org).

Further statistical analysis was performed in R (version 4.1.1) using an in-house script. The proteinGroups output table was processed as follows. First, decoy proteins and proteins only identified by site were filtered out, leading to a list of 5165 protein identifications. Only proteins with at least three quantification values in one experimental condition were kept, leading to a list of 5162 proteins reliably quantified proteins. Next, reporter intensity-corrected values were log2 transformed and median-normalized. Missing values were imputed by random sampling from a normal distribution centered around the noise level (package DEP)^75^. Pairwise comparison testing (moderated t-test) between groups was then carried out using the limma package^76^, with statistical significance for differential regulation set at an FDR cut-off value of 0.05 and a |log2(fold change)| above 1. Differentially abundant proteins detected over all comparisons were visualized in a heatmap following non-supervised hierarchical clustering of z-scored Log2 reporter intensity-corrected values.

### In silico prediction of peptides immunogenicity

To evaluate the immunogenic potential of expressed proteins, we performed in silico MHC presentation and immunogenicity predictions. Protein sequences were first fragmented into overlapping 9–11-mer and 15-mer peptides, corresponding to canonical MHC class I and class II epitope lengths, respectively. Peptide binding affinity to MHC molecules was predicted using MHCnuggets^77^, a long short-term memory deep neural network trained on in vitro binding affinity datasets and immunopeptidomics data. Predictions were generated for the mouse MHC class I alleles H2-Db and H2-Kb, and the class II allele H2-Iab. Based on the predicted binding affinities, each peptide was ranked relative to a reference set of 100,000 peptides, yielding a percentile-based MHCrank score. Peptides with an MHCrank ≤ 2 were considered likely to be presented. Predicted MHC class I binders were further assessed for immunogenicity using neoIM, a random forest classifier trained to distinguish immunogenic from non-immunogenic MHCI peptides based on their physicochemical properties. Peptides with a neoIM^78^ score ≥ 0.64 were classified as strongly immunogenic, indicating a high likelihood of eliciting a CD8⁺ T cell response. The predicted ferroptosis-associated MHC-I and MHC-II peptide repertoires highlight potential alterations in the tumor antigenic landscape but do not provide direct evidence of peptide immunogenicity, and should therefore be considered a hypothesis-generating resource.

### Generation of bone-marrow-derived dendritic cells (DCs)

DCs were isolated as described previously^34^. Bone marrow was collected from the tibias and femurs of mice in RPMI medium (Cat. 21875034, Gibco, USA) supplemented with 10% heat-inactivated HyClone™ FetalClone™ I serum (Cat. SH30080.03HI, Cytiva, USA), 20 ng/ml murine GM-CSF (UGent-IRC-VIB Protein Core Facility), 1% L-glutamine (Cat. 25030081, Gibco, USA), 100 µM sodium pyruvate (Cat. 11360070, Gibco, USA), 100 units/ml penicillin/100 µg/L streptomycin (Cat. 15140122, Gibco, USA) and 50 μM 2-mercapthoethanol (Cat. 21985023, Gibco, USA).

The bone marrow was aspirated with a 25-gauge needle (0.5 ×25 mm), resuspended, and filtered through a 70-μm cell strainer (Corning, USA) to remove coarse debris. Erythrocytes were lysed using a lysing solution (Beckman Colter, USA). The cells were cultured for up to eight days, with fresh culture medium added on day three and a medium refresh on day six.

### Analysis of DCs maturation

On the eighth day of DCs cultivation, the cells were harvested for subsequent co-cultivation. Lysates from untreated or RSL3-treated GL261 glioma cells were prepared as previously described. A total of 1 mg of protein was added to a suspension of 500,000 DCs and incubated for 90 minutes. The DCs were either treated with lipopolysaccharide (0.5 μg/ml) for 24 h to activate them (positive control) or left untreated. After the 24-hours, all cells were collected, centrifuged (300 g for 5 min at 4 °C), and washed once in PBS (Gibco, USA). The maturation of DCs was analyzed by immunostaining with the following antibodies:

- APC-eFluor 780-anti-CD11c (ThermoFisher Scientific, Cat. 47-0114-82);
- SB600-MHC class II (Biolegend, Cat. 107639);
- AF700-anti-CD86 (ThermoFisher Scientific, Cat. 62-0862-82);
- eFluor 450-anti-CD80 (ThermoFisher Scientific, Cat. 48-0801-82);
- mouse Fc block (eBioscience, Cat. 16016185);
- Sytox Red (ThermoFisher Scientific, Cat. S34859).

The manufacturer’s recommended dilutions were used.

Mature DCs were identified as a percentage of viable (Sytox Red^-^) CD11c^+^MHCII^+^, CD11c^+^CD80^+^, and CD11c^+^CD86^+^ cells. Samples were acquired with a BD LSRFortessa flow cytometer (BD, USA), and data analysis was conducted using FlowJo software (V.10.0.8). The gating strategy is shown in Supplementary Fig. 5.

### DC vaccination in an orthotopic glioma mouse model

Orthotopic tumor inoculation was performed as previously described^33,34^. Briefly, mice were anesthetized with isoflurane (5% induction, 1.5–2% maintenance) (CAS 26675-46-7, Laboratorios Karizoo, Spain). After making an incision in the scalp and exposing the skull, either 20,000 GL261 glioma cells in 2 μl of PBS or 500,000 CT-2A cells in 3 μl of PBS were injected into the brains at the following coordinates: AP: –2.0 mm, ML: –2.0 mm, DV: –3.3 mm (relative to bregma). Cells were injected at a rate of 0.3 µl/min using a Hamilton syringe mounted on a motorized stereotactic injector (World Precision Instruments, USA). After injection, the needle was left in place for 5 min before withdrawing it. The scalp was sutured, treated with neobactracine (Cat. 2132926, Erfa, Belgium) to prevent infection, and Metacam analgesia was administered (0.002 mg/mouse, CNK 2458032, Boehringer Ingelheim, Germany). DC vaccine was injected intraperitoneally (i.p.) at 1*10^6^ DCs per mouse. In prophylactic experiments mice received DC vaccine 14 and 7 days before the intracranial inoculation of viable tumor cells. In therapeutic experiments, mice received DC vaccine 2, 9 and 16 or 7, 14 and 21 days after the intracranial injection. For analysis of the requirements of HMGB1 and TLR4 agonizts, a DC vaccine was prepared from TLR4 knock out mice and administered at 14 and 7 days before the intracranial challenge with GL261 cells. TLR4^-/-^ mice still alive on day 56 after the first challenge with viable GL261 cells were re-challenged intracranially with 2*10⁴ viable glioma GL261 cells. An MRI scan was performed on all mice surviving on day 116 after the initial glioma injection.

### Neurological status assessment

Following intracranial inoculation with glioma GL261 or CT-2A cells, mice were monitored weekly, and the functional state of the central nervous system was assessed on a modified scale (Tab. S1) to evaluate the severity of neurological deficit in mice^34^. This scale includes assessments of motor activity, coordination, reflexes, muscle tone, ptosis, and exophthalmos. Each test was scored as follows: 2 points for no reaction, 0 points for normal reaction, and 1 point for partial disturbances. The scores were aggregated and interpreted as follows: 19–20 points indicated severe damage or death of the central nervous system; 15–18 points indicated high damage; 8–14 points indicated moderate damage; 2–7 points indicated light damage; and 0-1 points suggested no damage. An investigator, blinded to the identity of each assessment group, conducted the neurological evaluations.

### Magnetic resonance imaging

To assess the dynamics of intracranial tumor growth in the prophylactic model, magnetic resonance imaging (MRI) was conducted using a 7 Tesla MRI scanner (BioSpin PharmaScan 70/16, Bruker, Ettlingen, Germany) equipped with a mouse body volume coil. Ten minutes before scanning, a gadolinium-based contrast agent (CNK 1663822, Dotarem, Guerbet, France; 2 mmol/kg) was administered intraperitoneally. The animals were anesthetized (isoflurane: 5% induction, 1.5–2% maintenance) in a fixed position within the magnet tunnel for approximately 20 minutes. A whole brain anatomical T1-weighted scan was acquired with the following settings: repetition time (TR) of 1453 ms; echo time (TE) of 9.1 ms; four averages; echo train length of 4; field of view of 3 × 2.4 cm; matrix of 200 × 200; and 30 contiguous slices with a thickness of 600 µm, strategically selected to capture the tumor area. Data were analyzed using MicroDicom DICOM Viewer 2022.3.

### Ex vivo restimulation of lymphocytes and IFN-γ ELISpot assay

Three DC vaccines were administered at seven-day intervals to the following mouse groups: PBS (vehicle), DC alone (DCs without co-culture with glioma cells), DC-GL261-OVA-FT (DCs co-cultured with lysates of GL261 glioma cells and 1 µM OVA peptide), DC-GL261-OVA-MTX (DCs co-cultured with immunogenic apoptotic lysates of GL261 cells and 1 µM OVA peptide), or DC-GL261-OVA-RSL3 (DCs co-cultured with ferroptotic lysates of GL261 cells and 1 µM OVA peptide).

Four days after the final vaccination, the mice were anesthetized and euthanized by cervical dislocation. Inguinal lymph nodes were isolated, minced, and centrifuged (600 g, 7 minutes). The cells were resuspended in RPMI medium containing 2 mM glutamine, 100 units/ml penicillin, 100 µg/L streptomycin, and 10% fetal bovine serum. Cells were counted using a Countess 3 automated cell counter (ThermoFisher Scientific, USA) and diluted to 100,000 viable cells per 100 µl. The cells were placed in pre-coated ELISpot plates (Cat. 3321-4APT-2, Mabtech, Sweden) and restimulated with OVA257-264 (SIINFEKL) peptide (Cat. S7951-1MG, Sigma-Aldrich, Germany). After incubation for 24 h, the plates were washed according to the manufacturer’s instructions, read on a Mabtech IRIS 2 reader (software version 1.1.59.128, Mabtech, Sweden), and the number of Spot Forming Units was analyzed.

### Tumor isolation and characterization of the glioma microenvironment

Three DC vaccines were administered on days 2, 9 and 16 after intracranial inoculation of glioma GL261 cells to the following mouse groups: PBS (vehicle), DC alone (DCs without co-culture with glioma cells), DC-GL261-FT (DCs, co-cultured with the FT lysates of glioma GL261 cells), DC-GL261-MTX (DCs co-cultured with immunogenic apoptotic lysates of GL261 cells), or DC-GL261-RSL3 (DCs co-cultured with ferroptotic lysates of GL261 cells).

Two days after the final vaccination, the mice were anesthetized and euthanized by cervical dislocation. Intracranial tumors were isolated, placed at RPMI and cut into small pieces. An enzyme mix stock solution was added to achieve final concentrations of 90 U/mL DNase I (Thermofisher Scientific, DNAse I, RNAse-free, Cat. EN0521), 30 U/mL collagenase type I (Thermofisher Scientific, Collagenase Type I, Cat. 17018029) and 1200 U/mL collagenase type IV (Thermofisher Scientific, Collagenase Type IV, Cat. 17104019). Samples were incubated for 20 min at 37 °C.

Following enzymatic digestion, samples were homogenized in 5 ml of RPMI, filtered twice through a a 100 µm cell strainer, and centrifuged at 515 g for 7 minutes at 4 °C. Cell pellets were resuspended in red blood cell lysis buffer, incubated for 3 min on ice, and washed with RMPI. After centrifugation, 5*10^6^ cells were transferred to FACS tube, washed again with RPMI, and stained with the following antibodies:

- Anti-CD8a-BUV 737 (clone 53-6.7, Invitrogen, Cat. 367-0081)
- Anti-CD4-Vio Blue (clone REA604, Miltenyi Biotech, Cat. 130-118-696)
- Anti-CD137-PE-Vio 615 (clone REA936, Miltenyi Biotech, Cat. 130-115-572)
- Anti-Granzyme B-PE-FITC (clone REA226, Miltenyi Biotech, Cat. 130-118-480)
- Anti-CD44-APC-Vio779 (clone REA664, Miltenyi Biotech, Cat. 130-118-695)
- Anti-CD62L-PerCP-Vio700 (clone REA828, Miltenyi Biotech, Cat. 130-112-840)
- Anti-CD39-APC (clone REA870, Miltenyi Biotech, Cat. 130-114-359)
- Anti-FoxP3-PE (clone REA788, Miltenyi Biotech, Cat. 130-111-678)
- Zombie-UV (Biolegend, Cat. 423107).

The manufacturer’s recommended dilutions were used.

For intracellular FoxP3 staining, cells were fixed and permeabilized using the FOXP3 buffer set (Miltenyi Biotech, Cat. 130-093-142) according to the manufacturer’s instructions. Samples were acquired using a BD Symphony A5 flow cytometer (BD, USA), and data were analyzed with FlowJo software (V.10.0.8). The gating strategy is shown in Supplementary Fig. 7.

### Blocking of DAMPs: CRT, HMGB1, and ATP

GL261 glioma cells were treated with RSL3, and lysates were prepared as previously described. The lysates were co-cultured for 40 minutes with antibodies against CRT (6 µg/10⁶ cells, Cat. Ab22683, Abcam, USA), HMGB1 (10 µg/10⁶ cells, Cat. Ab79823, Abcam, USA) or apyrase (13 units/10⁶ cells, CAS 9000-95-7, Sigma-Aldrich, Germany). The lysates were then used to prepare DC vaccines following the previously described protocol. Mice were vaccinated 14 and 7 days before intracranial inoculation of viable glioma GL261 cells. After inoculation, the neurological status and survival of mice were monitored. An additional control experiment was conducted using isotype control antibodies to CRT (Cat. Ab 170190, Abcam, USA) and HMGB1 (Cat. Ab172730, Abcam, USA).

### Reconstitution of DAMPs in FT lysates: CRT and ATP

GL261 glioma cells were subjected to freeze-thaw (FT) cycles as described above. Recombinant cell-expressed human calreticulin (CRT, 3 µg/10⁶ cells, kindly provided by Dr. Peter van Endert, Université de Paris, France)^49^ or ATP (1 µM, A7699, Sigma-Aldrich, Germany) was added to the lysates^79^, which were then used to prepare DC vaccines following the previously described protocol. Mice were vaccinated on days −14 and −7 prior to intracranial inoculation of viable glioma GL261 cells.

### Statistical analysis

Data were analyzed in GraphPad Prism (V.9.2). The normality of the data distribution was assessed with the Shapiro–Wilk test. One-way ANOVA with Dunnett’s multiple comparisons correction, Kruskal-Wallis test with Dunn’s multiple comparisons correction, Two-way ANOVA with Tukey’s multiple comparisons test, were used for statistical analysis. Kaplan-Meier survival curves were analyzed using the log-rank Mantel-Cox test. A p-value of <0.05 was considered statistically significant. All experiments were performed with at least three independent biological replicates. All in vitro cell death experiments contained 3 technical replicates. For all in vivo experiments, each mouse was considered an independent biological replicate. The number “n” in the figure legends means the number of biological replicates.

### Reporting summary

Further information on research design is available in the Nature Portfolio Reporting Summary linked to this article.

## Supplementary information

Supplementary Information

Description of Additional Supplementary Files

Supplementary Dataset 1

Supplementary Dataset 2

Supplementary Dataset 3

Supplementary Dataset 4

Reporting Summary

Transparent Peer Review file

## Source data

Source Data

## Data Availability

The Proteomics data generated in this study have been deposited in PRIDE database under accession code PXD060116^74^. All data reported in this manuscript are available in the main text and supplementary information. Source data are provided with this paper.
