# Supplementary material for: Ferroptosis-armed dendritic cell vaccines for glioma immunotherapy
Source: Nat Commun. 2026 May 7;17:6158. doi: 10.1038/s41467-026-72737-6 (PMC13365579; doi:10.1038/s41467-026-72737-6)
Supplement: Supplementary file 1 — Supplementary Information [file 41467_2026_72737_MOESM1_ESM.pdf]

Supplementary Information

Ferroptosis-armed dendritic cell vaccines for glioma immunotherapy

Mariia Saviuk[1,2], Victoria D. Turubanova[1,3], Sara De Brée[1,2], Sandra Van Lint[2,4], Teresa Mendes Maia[5,6,7], Simon Devos[5,6,7], Iuliia Efimova[1,2], Julie Braet[2,4], Lore Van Oudenhove[8], Gitta Boons[8], Faye Naessens[1,2], Robin Demuynck[1,2], Ellen Saeys[1,2], Christian Vanhove[9], Lukas Bunse[10,11], Peter M. van Endert[12,13], Robrecht Raedt[14], Maria V. Vedunova[15], Olga Krysko[1], Roosmarijn E. Vandenbroucke[16,17], Karim Vermaelen[2,4], Tatiana A. Mishchenko[15], Elena Catanzaro[1,2,18,*], Dmitri V. Krysko[1,2,18]

[1]Cell Death Investigation and Therapy (CDIT) Laboratory, Anatomy and Embryology Unit, Department of Human Structure and Repair, Faculty of Medicine and Health Sciences, Ghent University, Ghent, Belgium
[2]Cancer Research Institute Ghent, Ghent, Belgium
[3]Institute of Neurosciences, National Research Lobachevsky State University of Nizhny Novgorod, Nizhny Novgorod, Russia
[4]Thoracic Tumor Immunology Laboratory (TTIL), Department of Internal Medicine and Pediatrics, Faculty of Medicine and Health Science, Ghent University, Ghent, Belgium
[5]VIB Proteomics Core, VIB, Ghent, Belgium
[6]VIB-UGent Center for Medical Biotechnology, VIB, Ghent, Belgium
[7]Department of Biomolecular Medicine, Ghent University, Ghent, Belgium
[8]myNEO Therapeutics, Ghent, Belgium
[9]IBiTech-MEDISIP-Infinity Laboratory, Department of Electronics and Information Systems, Faculty of Engineering and Architecture, Ghent University, Ghent, Belgium
[10]Clinical Cooperation Unit (CCU) Neuroimmunology and Brain Tumor Immunology, German Cancer Research Center (DKFZ), Heidelberg, Germany
[11]Neurology Clinic, Medical Faculty Mannheim, University Heidelberg, Mannheim, Germany
[12]Université Paris Cité, INSERM, CNRS, Institut Necker Enfants Malades, Paris, France
[13]Service Immunologie Biologique, AP-HP, Hôpital Universitaire Necker-Enfants Malades, Paris, France
[14]4Brain, Department of Head and Skin, Faculty of Medicine and Health Sciences, Ghent University, Ghent, Belgium
[15]Institute of Biology and Biomedicine, National Research Lobachevsky State University of Nizhny Novgorod, Nizhny Novgorod, Russia
[16]VIB Center for Inflammation Research, Ghent Belgium
[17]Department of Biomedical Molecular Biology, Faculty of Sciences, Ghent University, Ghent, Belgium
[18]These authors shared the last authorship

*Correspondence:
Dr. Elena Catanzaro
Elana.Catanzaro@UGent.be

Table of Contents

Supplementary Figures 3
Supplementary Figure 1 4
Supplementary Figure 2 5
Supplementary Figure 3 7
Supplementary Figure 4 9
Supplementary Figure 5 11
Supplementary Figure 6 12
Supplementary Figure 7 14
Supplementary Figure 8 16
Supplementary Figure 9 17
Supplementary Figure 10 19
Supplementary Figure 11 20
Supplementary Tables 21
Supplementary Table 1 21

Supplementary Figures

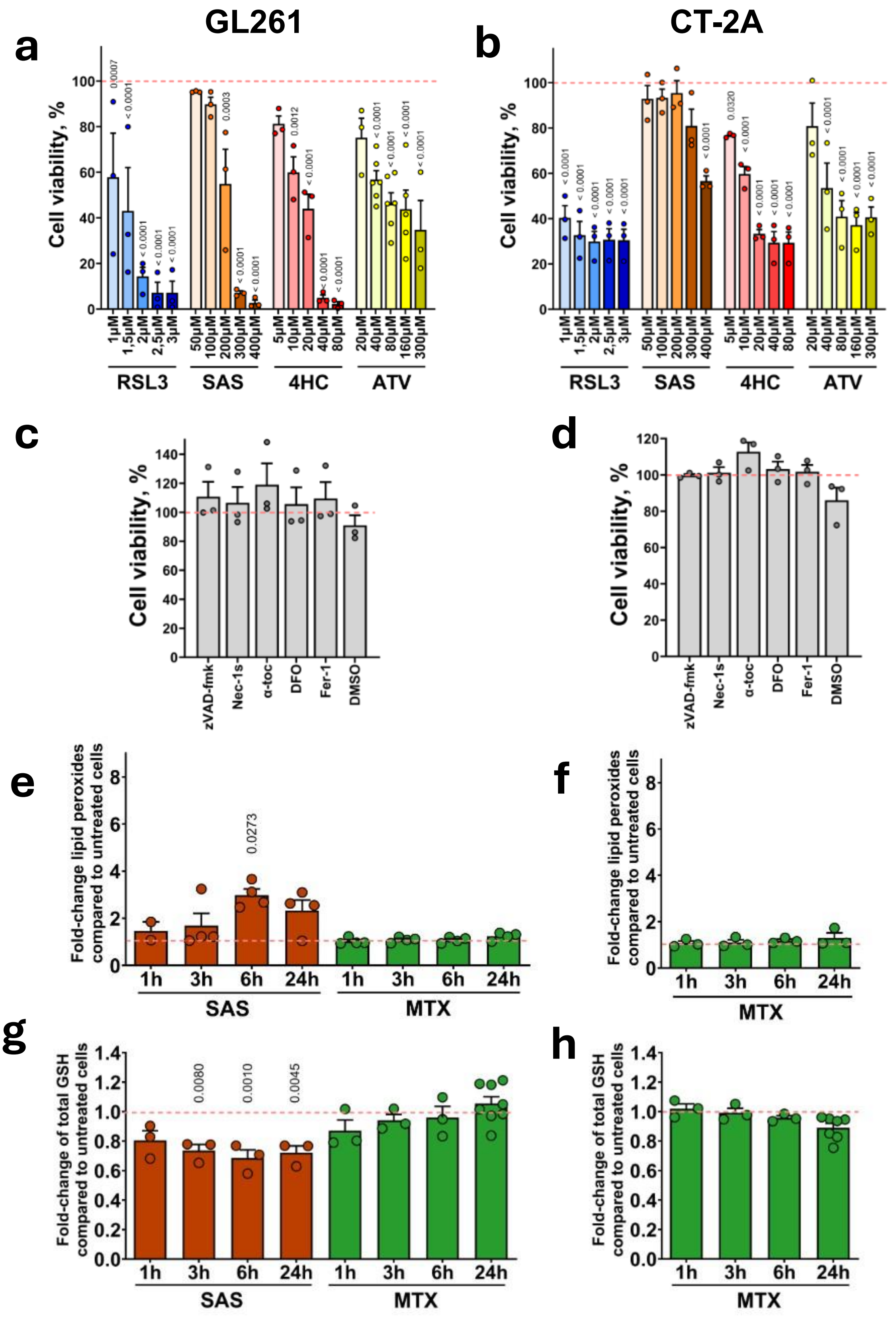

Supplementary Figure 1. Characterization of cell death induction, lipid ROS generation, and GSH depletion in glioma cells treated with ferroptosis inducers

(a, b) Cell death was analyzed using MTS assay in glioma GL261 **(a)** and CT-2A cells **(b)**. Cell death was induced by RSL3 (1 µM – 3 µM), SAS (50 µM – 400 µM), 4HC (5 µM – 80 µM) or ATV (20 µM – 300 µM) for 48 hours.
(c, d) Cell death was analyzed using the MTS assay in glioma GL261 (**c**) and CT-2A (**d**) cells. The toxicity of cell death inhibitors or vehicle (DMSO) was assessed. Data are presented as mean ± SEM from three independent experiments. Statistical significance was determined using one-way ANOVA followed by Dunnett's multiple comparisons test.
(e, g) Lipid ROS (lipid peroxide) generation during cell death was assessed by treating GL261 cells with SAS (300 µM) or MTX (2.5 µM) for 1, 3, 6, or 24 hours (**e**), and CT-2A cells with MTX (2.5 µM) for the same timepoints (**g**), followed by BODIPY C11 staining. Data are presented as fold change relative to untreated cells.
(f, h) Glutathione (GSH) levels during cell death were assessed after treating GL261 cells with SAS (300 µM) or MTX (2.5 µM) for 1, 3, 6, or 24 hours (**f**), and CT-2A cells with MTX (2.5 µM) for the same timepoints (**h**).
The values are expressed as means ± SEM, derived from 3 (1a – RSL3, SAS, 4HC, ATV 20 µM and 300 µM, 1b, 1c, 1d, 1f SAS and MTX 1, 3, 6h, 1g, 1h – MTX 1, 3, 6h), 4 (1e), 5 (1a – ATV 160 µM), 6 (1a – ATV 40 µM and 80 µM) 7 (1h – MTX 24h), 8 (1e – MTX 24h) independent biological experiments, each experiment contained 3 technical replicates. Statistical significance was determined using one-way ANOVA followed by Dunnett's multiple comparisons test or the Kruskal-Wallis test with Dunn's multiple comparisons correction. Source data are provided as a Source Data file.

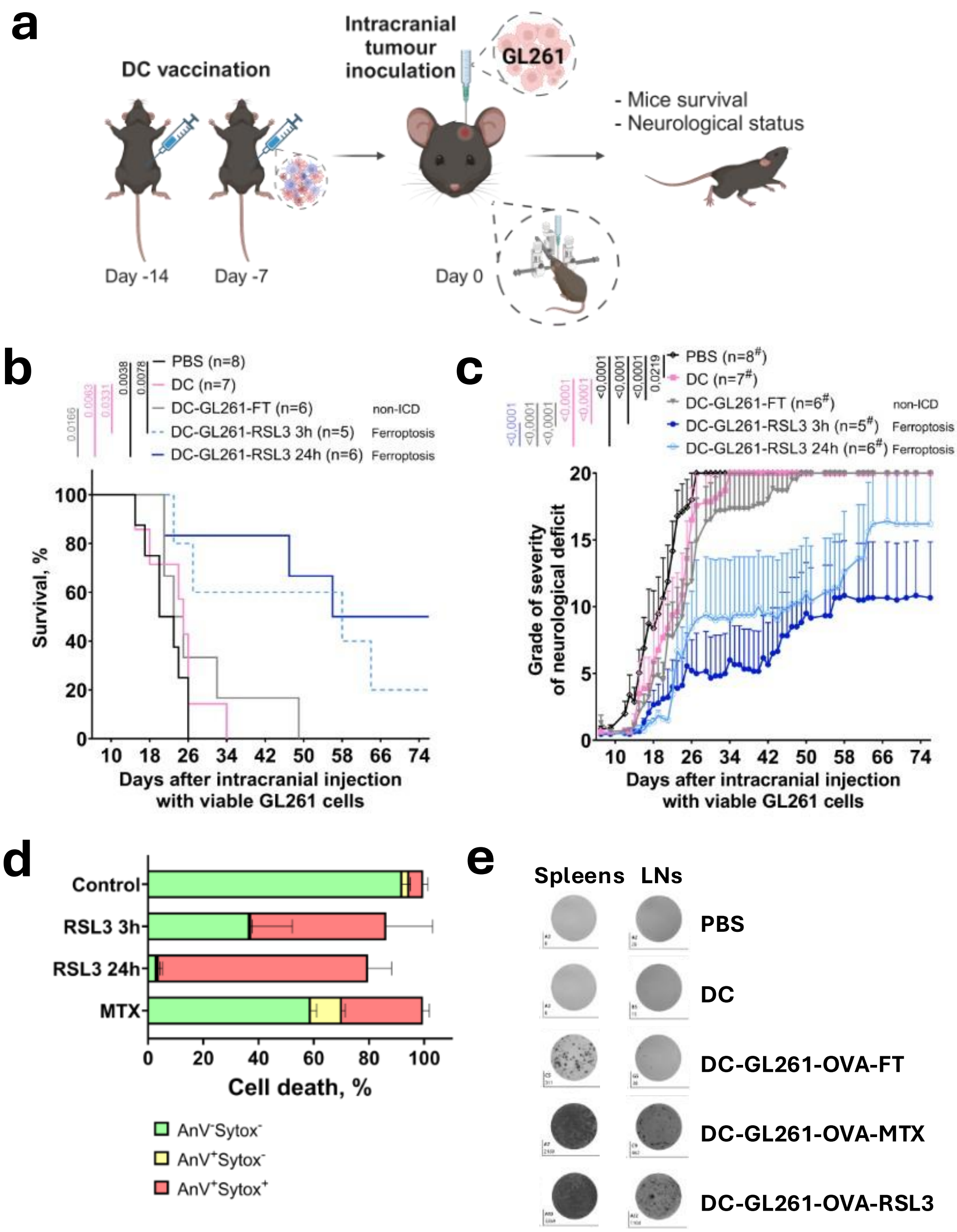

Supplementary Figure 2. Fully executed ferroptosis: a key to effective DC vaccines against glioma

(a) Procedure to evaluate the effectiveness of DC vaccines in a prophylactic setting using an orthotopic intracranial mouse model. Mice received DC vaccines on days -14 and -7, followed by an intracranial injection with viable glioma GL261 cells on day 0. Mouse survival and neurological status were monitored. Created in BioRender. Krysko, D. (2026) https://BioRender.com/382aqv7.

Mouse survival **(b)** and neurological status **(c)** were monitored after injection of ferroptosis-armed DC vaccines and subsequently challenged intracranially with viable GL261 cells. Ferroptotic lysates were prepared by inducing ferroptosis in GL261 cells with RSL3 for 3 h (2.5 μM, DC-GL261-RSL3 3h, n=5) or 24 h (2.5 μM, DC-GL261-RSL3 24 h, n=6). Controls included DC vaccines loaded with glioma GL261 lysates subjected to F/T cycles (negative control, non-ICD, DC-GL261-FT, n=6), unloaded DCs (negative control, DCs, n=7) or PBS (negative control, n=8). Survival and neurological

status were monitored for up to 76 days. Statistical analysis for mice survival was determined by Mantel-Cox logarithmic test. Data of the neurological status of the mice are shown as means ± SEM, statistical analysis was performed using a two-way ANOVA followed by Tukey's multiple comparisons correction. Source data are provided as a Source Data file.

(d) Quantification of ferroptotic cell death in GL261 cells by flow cytometry. GL261 cells were treated with 2.5 μM RSL3 for 3 or 24 hours, or with 2.5 μM MTX for 24 hours. Cell death was assessed using Sytox Blue and Annexin V-FITC staining. Data are presented as mean ± SEM from 3 (RSL3 3h and 24h) or 4 (Control, MTX) independent experiments.

(e) IFN-γ ELISpot assay of spleens and draining lymph nodes from immunized mice (performed as described **in Fig. 4d-f**). Source data are provided as a Source Data file.

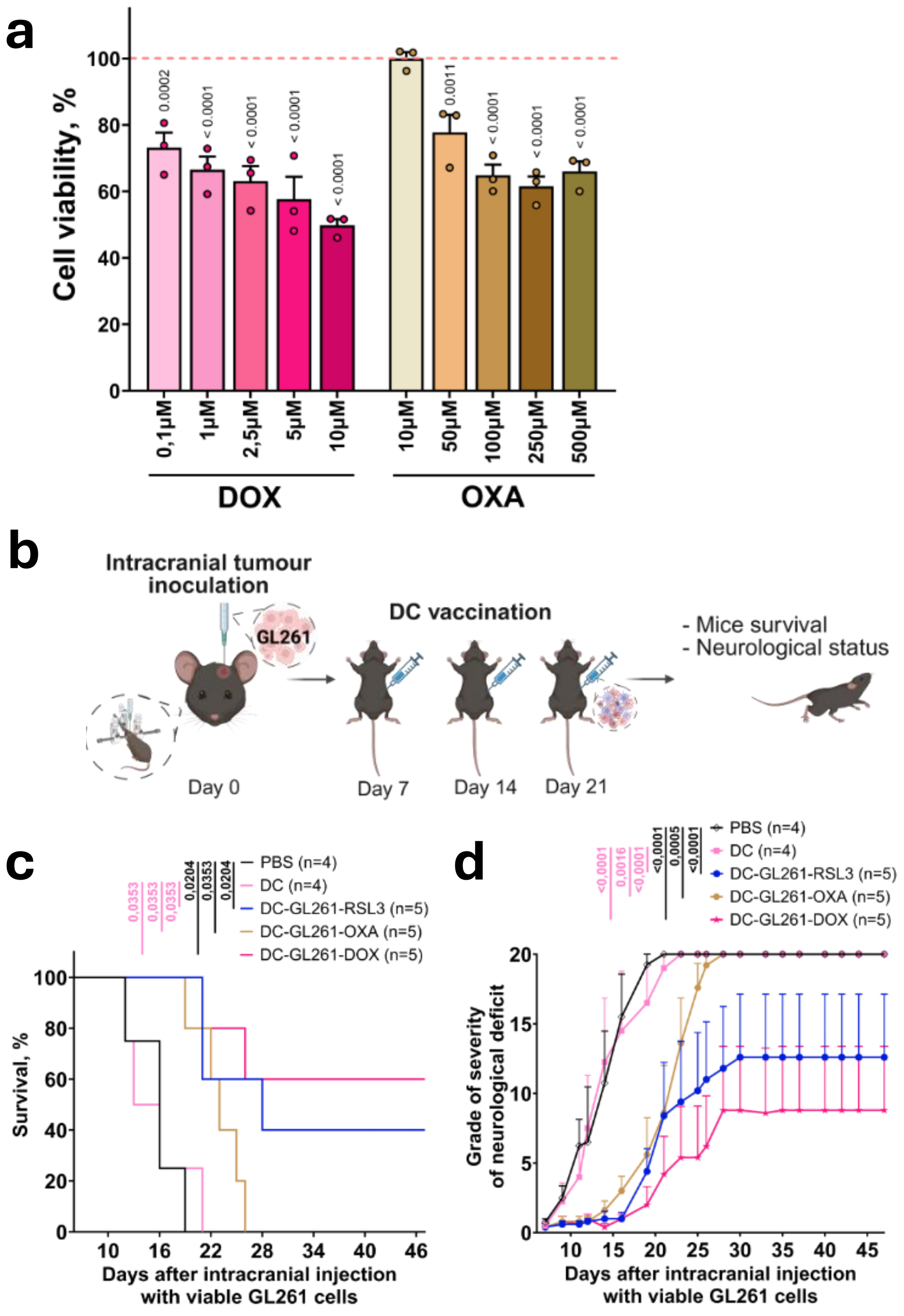

Supplementary Figure 3. Therapeutic vaccination with DC vaccine loaded with apoptotic lysates

(a) Cell death was induced by DOX (0,1 µM – 10 µM) or OXA (10 µM – 500 µM) on glioma GL261 cell line. Statistical significance was determined using a one-way ANOVA followed by Dunnett multiple comparisons test. Data are presented as mean ± SEM from 3 independent biological experiments, each experiment contained 3 technical replicates.

(b) Procedure to assess the efficacy of DC vaccines in a more clinically relevant therapeutic setting using an orthotopic intracranial mouse model. Mice were injected intracranially with viable glioma GL261 cells on day 0, followed by administration of the corresponding DC vaccine on days 7, 14 and 21. Created in BioRender. Krysko, D. (2026) https://BioRender.com/lr8nwhi. Mouse survival **(c)** and neurological status **(d)** were assessed following intracranial injection with viable glioma GL261

cells and subsequent therapy with ferroptosis-armed DC vaccines. The lysates were prepared by inducing ferroptosis in GL261 cells with RSL3 (2.5 μM, DC-GL261-RSL3, n=5) or by inducing apoptosis in GL261 cells with OXA (100 μM, DC-GL261-OXA, n=5) or DOX (5 μM, DC-GL261-DOX, n=5). Control groups included mice injected with unloaded DCs (negative control, DCs, n=4) or PBS (negative control, n=4). Survival and neurological status were monitored for up to 47 days. Mouse survival was analyzed using the Mantel-Cox log-rank test. Data of the neurological status of the mice are shown as means ± SEM, statistical analysis was performed using a two-way ANOVA followed by Tukey's multiple comparisons correction. Source data are provided as a Source Data file.

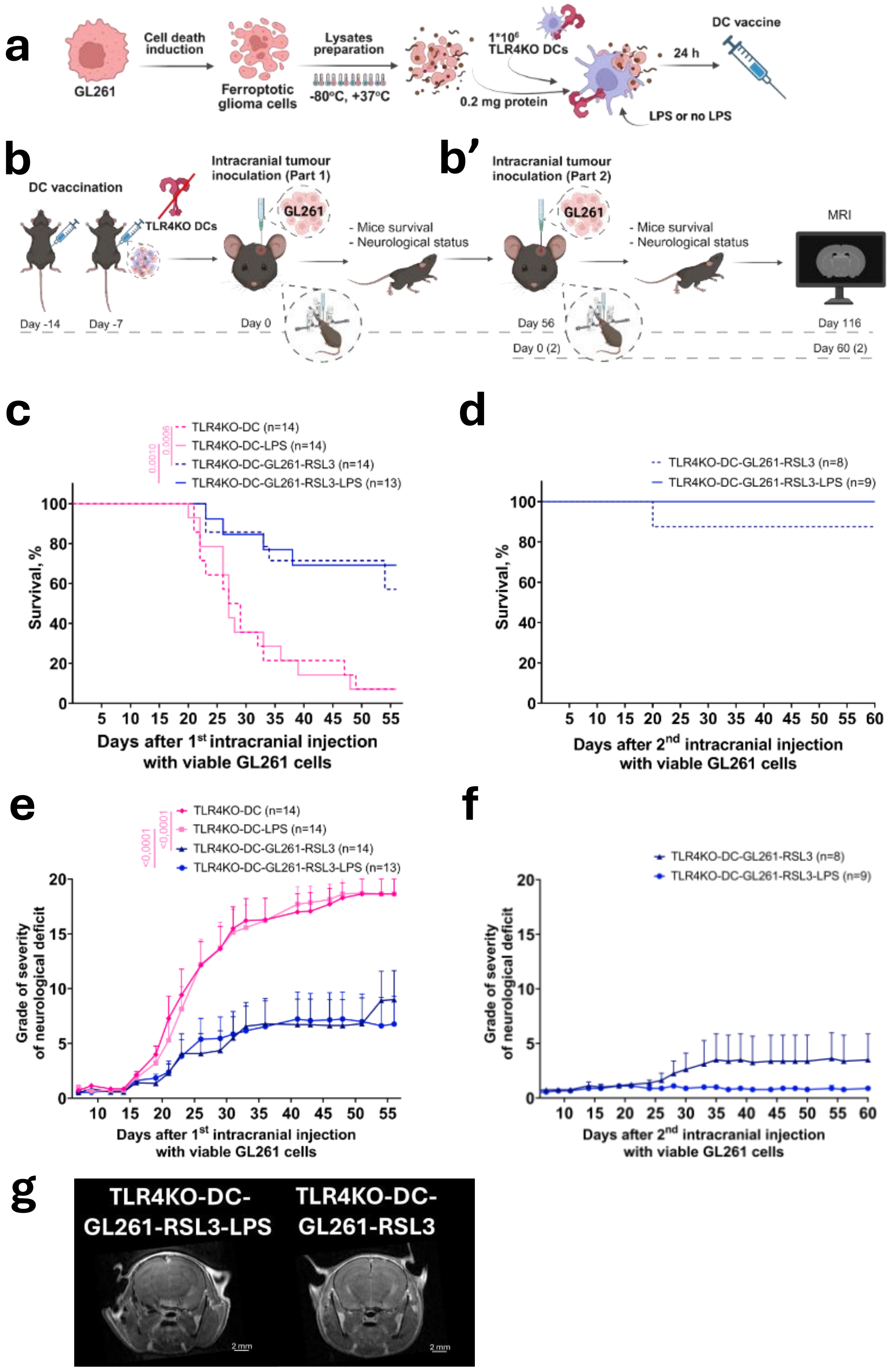

Supplementary Figure 4. Ferroptosis-armed DC vaccine is intrinsically immunogenic and does not require bacterial adjuvants

(a) Procedure for generating ferroptosis-armed DC vaccines from mouse bone marrow DCs of TLR4 knock out mice (TLR4KO-DCs). TLR4KO-DCs were co-cultured for 90 minutes with ferroptotic GL261 lysates (RSL3, 2.5 μM, 24 h) and then co-cultured with ferroptotic lysates in the presence or

absence of 0.5 µg/ml LPS for 24 hours. Created in BioRender. Krysko, D. (2026) https://BioRender.com/a4ljss9.

Part 1:

(b) Procedure for studying the effectiveness of TLR4KO-DC vaccines generated in the presence or absence of LPS (as explained in **A**) in wild type C57BL/6J mice (*i.e.,* **part 1**).

Wild type mice received ferroptosis-armed TLR4KO-DC vaccines (TLR4KO-DC-GL261-RSL3, n=14 or TLR4KO-DC-GL261-RSL3-LPS, n=13) or control vaccines (TLR4KO-DC, n=14 or TLR4KO-DC - LPS, n=14) on days -14 and -7, followed by intracranial injection of viable glioma GL261 cells on day 0 **(part 1)**. In part 1, mouse survival and neurological status were monitored up to day 56.

Part 2:

(b') Procedure **(part 2)** for assessing the development of immunological memory in immunocompetent C57BL/6J mice that had rejected viable GL261 cells due to therapy with ferroptosis-armed TLR4KO-DCs in **(b, part 1)**. **In part 2**, the surviving mice were re-challenged on day 0 of the second intracranial glioma injection (56 days after the first challenge) with a GL261 glioma cell dose that normally induces glioma growth in naive mice (*e.g.,* see **Fig. 2c,f**; the negative control group injected with PBS). Mouse survival and neurological status were monitored for up to 116 days after the first injection (*i.e.,* 60 after the second injection). The MRI scans were performed on day 116. Created in BioRender. Krysko, D. (2026) https://BioRender.com/a4ljss9.

(c: part 1; d: part 2) Mouse survival after therapy with ferroptosis-armed TLR4KO-DC vaccines (TLR4KO-DC-GL261-RSL3, n=14 or TLR4KO-DC-GL261-RSL3-LPS, n=13) and subsequent intracranial rechallenge with viable GL261 cells. Mouse survival was monitored for up to 60 days after the rechallenge. As controls, we used unloaded TLR4KO-DC treated with LPS (TLR4KO-DC-LPS, n=14) or left untreated (TLR4KO-DC, n=14).

(e: part 1; f: part 2) Neurological status after therapy with ferroptosis-armed TLR4KO-DC vaccines (TLR4KO-DC-GL261-RSL3, n=14 or TLR4KO-DC-GL261-RSL3-LPS, n=13) or unloaded TLR4KO-DC treated with LPS (TLR4KO-DC-LPS, n=14) or left untreated (TLR4KO-DC, n=14) was monitored up to day 56 **(e)** after the first challenge and up to day 60 after the rechallenge of survived mice (TLR4KO-DC-GL261-RSL3, n=8 or TLR4KO-DC-GL261-RSL3-LPS, n=9).

(g) Representative T1-weighted MRI images of coronal brain sections taken on day 60 after the second challenge (*i.e.,* day 116 after the first challenge; see part 2). Note the absence of tumors.

Statistical analysis for mice survival **(c, e)** was determined by Mantel-Cox logarithmic test. Data of the neurological status **(d, f)** of the mice are shown as means ± SEM. Statistical analysis was performed using a two-way ANOVA followed by Tukey's multiple comparisons correction. Source data are provided as a Source Data file.

Maturation gating strategy

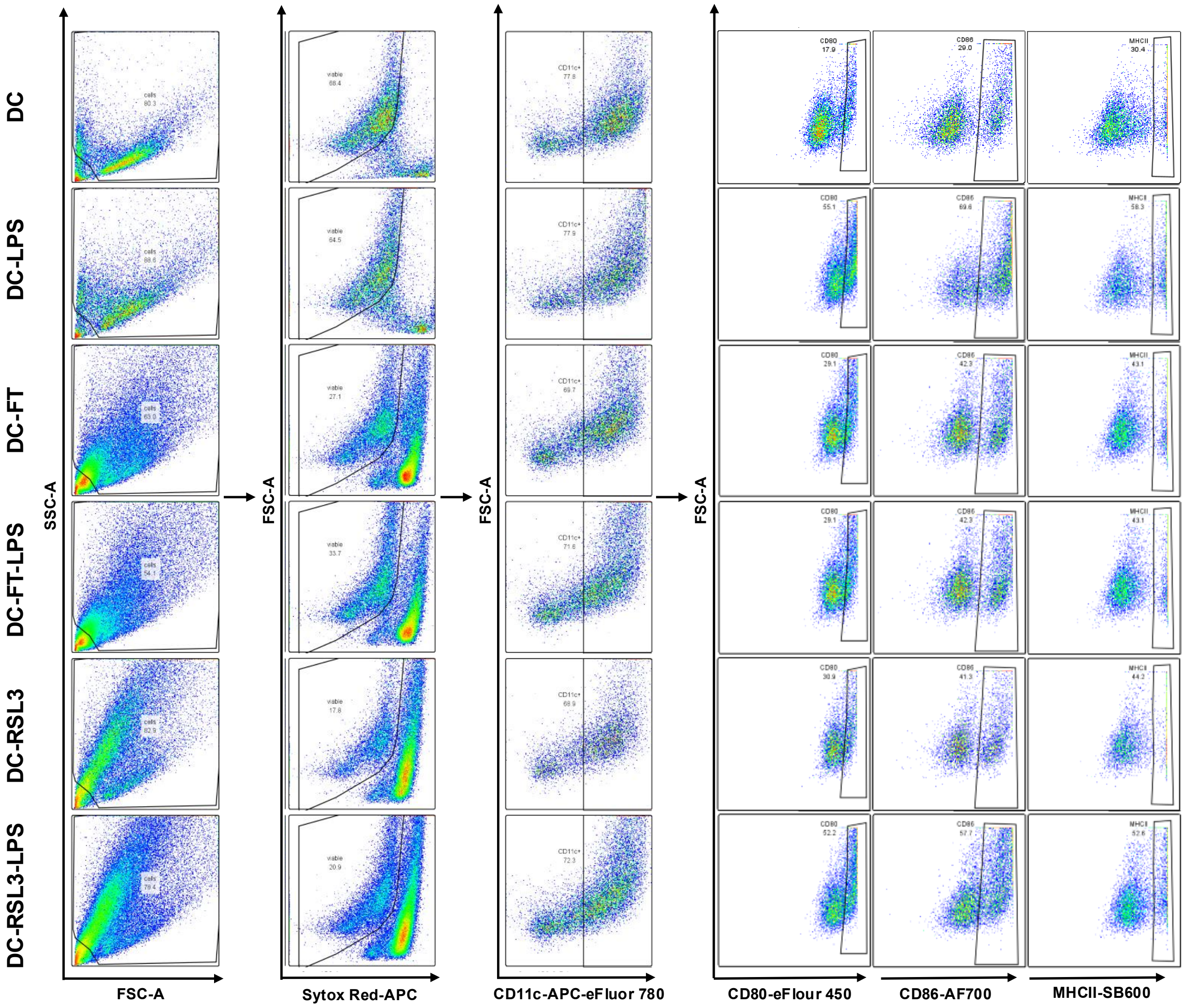

Supplementary Figure 5. The gaiting strategy for DC maturation analysis

Representative dot plots (quantified in **Fig. 7a-c**) showing the gating and percentage of CD11c$^+$CD80$^+$, CD11c$^+$CD86$^+$, and CD11c$^+$MHC-II$^+$ DCs in co-culture with either ferroptotic GL261 lysates (2.5 μM, 24 h, DC-GL261-RSL3) or freeze-thawed (F/T) GL261 (non-ICD, negative control, DC-GL261-FT) for 90 minutes and subsequently treated or not treated with lipopolysaccharide (LPS, 0.5 μg/ml) for 24 hours. Unloaded DCs stimulated with LPS served as a positive control (DC-LPS) and untreated cells (DC) served as negative control.

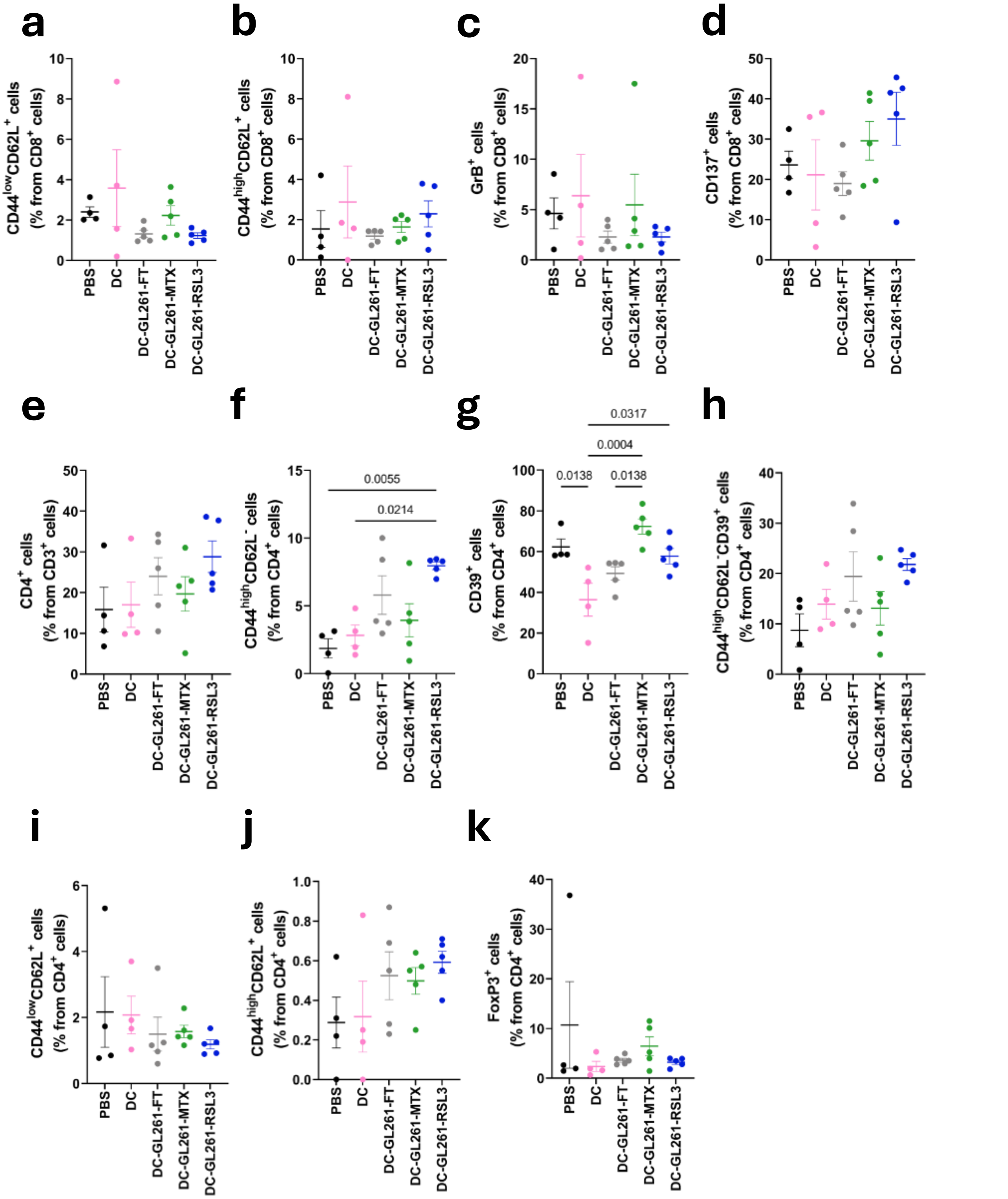

Supplementary Figure 6. Characterization of T cell infiltration in the tumor microenvironment following ferroptosis-armed DC vaccination

(a-d) Percentage of $CD8^+$ T cell infiltration. The ferroptosis-armed DC vaccine did not alter the infiltration of naïve ($CD44^{low}CD62L^+$) **(a)**, central memory ($CD44^{high}CD62L^+$) **(b)**, activated (GrB^+) **(c)**, or tumor-specific ($CD137^+$) **(d)** $CD8^+$ T cells.

(e-h) Percentage of $CD4^+$ T cell infiltration **(e)**, effector memory $CD4^+$ T cells ($CD44^{low}CD62L^-$) **(f)**, $CD39^+$ $CD4^+$ T cells **(g)**, and $CD39^+$ effector memory $CD4^+$ T cells **(h)**.

(i-k) The ferroptosis-armed DC vaccine did not alter the infiltration of naïve ($CD44^{low}CD62L^{+}$) **(i)**, central memory ($CD44^{high}CD62L^{+}$) **(j)**, or regulatory ($FoxP3^{+}$) **(k)** $CD4^{+}$ T cells. Statistical significance was determined using one-way ANOVA followed by Dunnett's multiple comparisons test. The percentages of cells are shown as mean values ± SEM from 5 biological replicates. Source data are provided as a Source Data file.

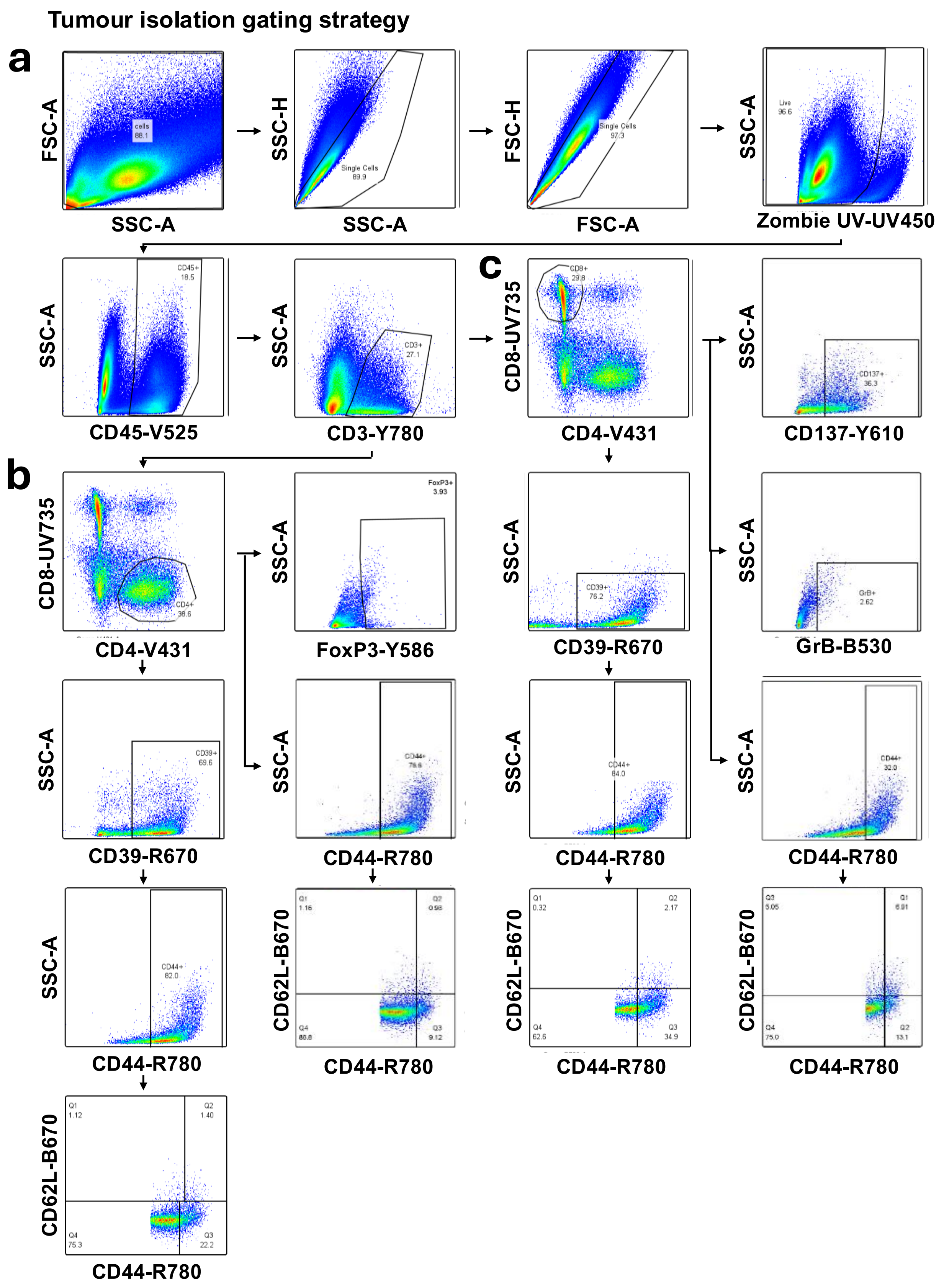

Supplementary Figure 7. Flow cytometry gating strategy for identification and characterization of T cell subsets in the glioma tumor microenvironment

Initially, single cells were extracted. Subsequently, viable cells (Zombie UV$^-$), CD45$^+$ (to exclude non-immune cells), and CD3$^+$ (to select T cells) were gated **(a)**. CD4$^+$ **(b)** and CD8$^+$ **(c)** T cell populations were then identified. Within the CD4$^+$ population **(b)**, further gating was performed to

identify FoxP3$^+$ regulatory T cells (Tregs), CD44$^+$, and CD39$^+$ T cells. In this study, three distinct subsets of T cells were analyzed: CD44$^+$ cells, naïve T cells (CD44^{low}CD62L$^+$), central memory T cells (CD44^{high}CD62L$^+$), and effector memory T cells (CD44^{high}CD62L$^-$). The same gating strategy was applied to identify naïve, central memory, and effector memory T cells within the CD4$^+$CD39$^+$ population. In the CD8$^+$ population **(c)**, tumor-specific T cells (CD137$^+$), activated T cells (Granzyme B$^+$), CD39$^+$, and CD44$^+$ T cells were identified. The same gating strategy was used to analyze naïve, central memory, and effector memory T cell subsets within the CD8$^+$ population. Source data are provided as a Source Data file.

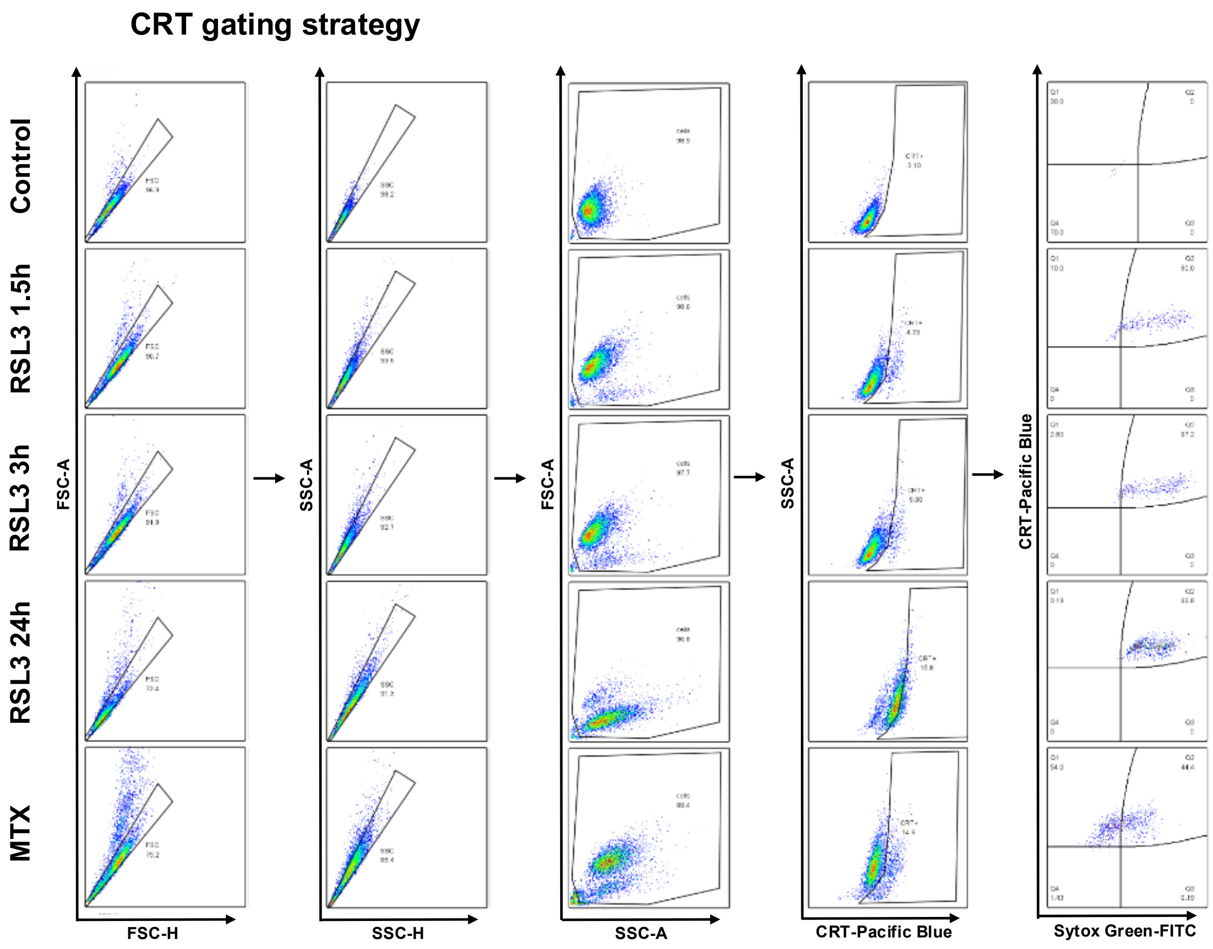

Supplementary Figure 8. Calreticulin^{+} cells gating strategy showing the identification and percentage of CRT^{+} Sytox^{-} GL261 cells which is quantified in **Fig. 7a**.

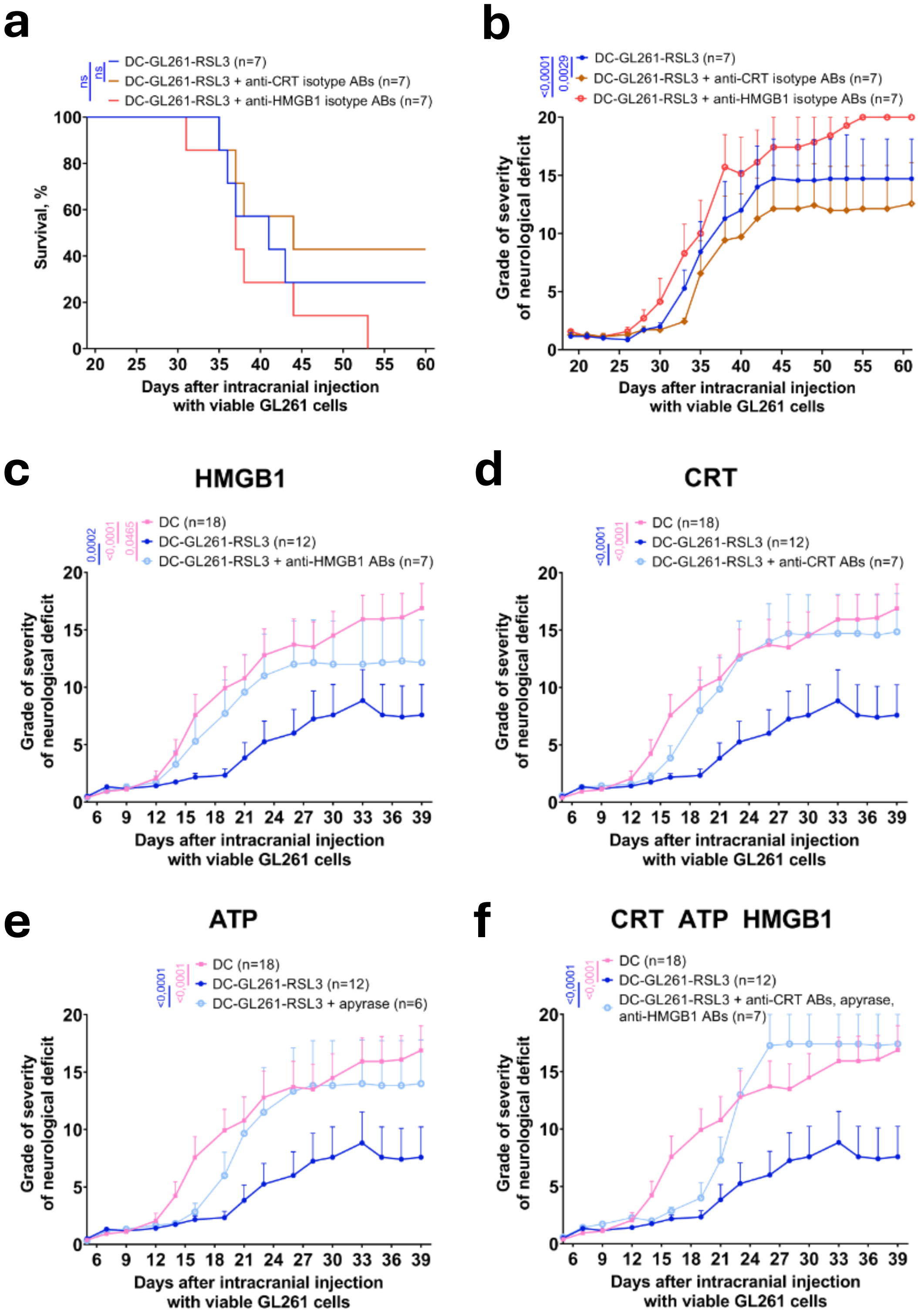

Supplementary Figure 9. Anti-tumor immunity from ferroptosis-armed DC vaccines depends on CRT and ATP signals and is independent on HMGB1-TLR4

(a, b) Analysis of survival **(a)** and neurological status **(b)** in an orthotopic intracranial mouse model. The ferroptotic glioma cells were subjected to six freeze-thaw cycles to prepare lysates, which were incubated with isotype control anti-HMGB1 antibodies (DC-GL261-RSL3-anti-HMGB1 isotype Abs, n=5) and isotype anti-CRT antibodies (DC-GL261-RSL3-anti-CRT isotype Abs, n=5) for 40 minutes or used to prepare the control vaccine (DC-GL261-RSL3, n=7). The DC vaccines were administered

to mice on days -14 and -7. On day 0, the mice were intracranially challenged with viable GL261 glioma cells. Mouse survival and neurological status were monitored over 60 days.
(c-f) Analysis of neurological status in an orthotopic intracranial mouse model (as described in **Fig. 7d-h**) following treatment with ferroptosis-armed DC vaccines under pharmacological inhibition of the DAMPs HMGB1 (DC-GL261-RSL3 + anti-HMGB1 Abs, n=7), CRT (DC-GL261-RSL3 + anti-CRT Abs, n=7), and ATP DC-GL261-RSL3 + apyrase, n=6) individually **(c-e)** or collectively DAMPs (DC-GL261-RSL3 + anti-CRT Abs, apyrase, anti-HMGB1 Abs, n=7) **(f)**. Ferroptosis armed DC vaccine (DC-GL261-RSL3, n=12) or unloaded DCs (DC, 18) were used as the controls. Statistical analysis for mice survival **(a)** was determined by Mantel-Cox logarithmic test. Data of the neurological status **(b-f)** of the mice are shown as means ± SEM, statistical analysis was performed using a two-way ANOVA followed by Tukey's multiple comparisons correction. Source data are provided as a Source Data file.

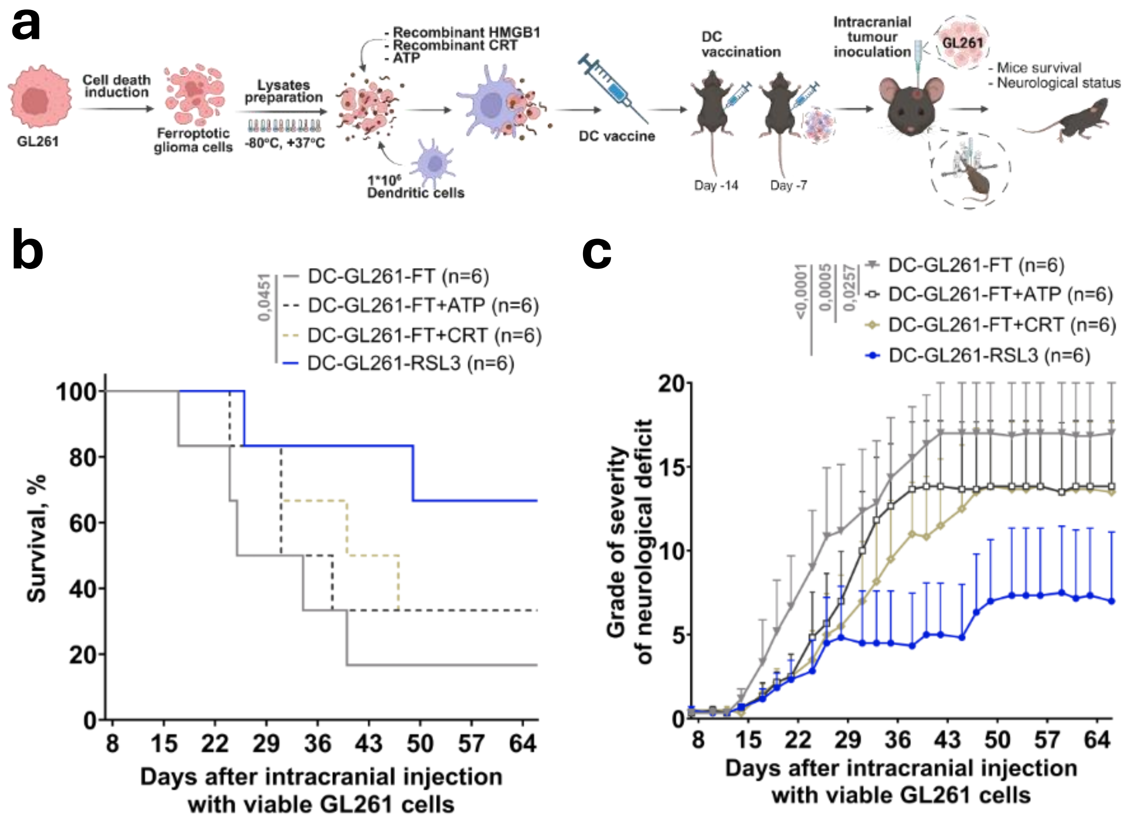

Supplementary Figure 10. Pharmacological reconstitution of DAMPs in non-immunogenic (FT) lysates (a) Procedure to evaluate the efficacy of pharmacological reconstitution of DAMPs (CRT and ATP) in an orthotopic intracranial mouse model. Mice received DC vaccines loaded with GL261 lysates subjected to freeze-thawing alone (non-ICD; DC-GL261-FT) or reconstituted with recombinant CRT (3 µg/mL; GL261-FT+CRT) or ATP (1 µM; GL261-FT+ATP). The corresponding DC vaccines were administered to mice on days -14 and -7. On day 0, mice were challenged intracranially with viable GL261 glioma cells. Created in BioRender. Krysko, D. (2026) https://BioRender.com/7avyt2w. Mouse survival **(b),** neurological status **(c)** were monitored. Mouse survival was analyzed using the Mantel–Cox log-rank test. Data of the neurological status **(b-f)** of the mice are shown as means ± SEM, in each group 6 mice were included, statistical analysis was performed using two-way ANOVA followed by Tukey's multiple comparisons test. Source data are provided as a Source Data file.

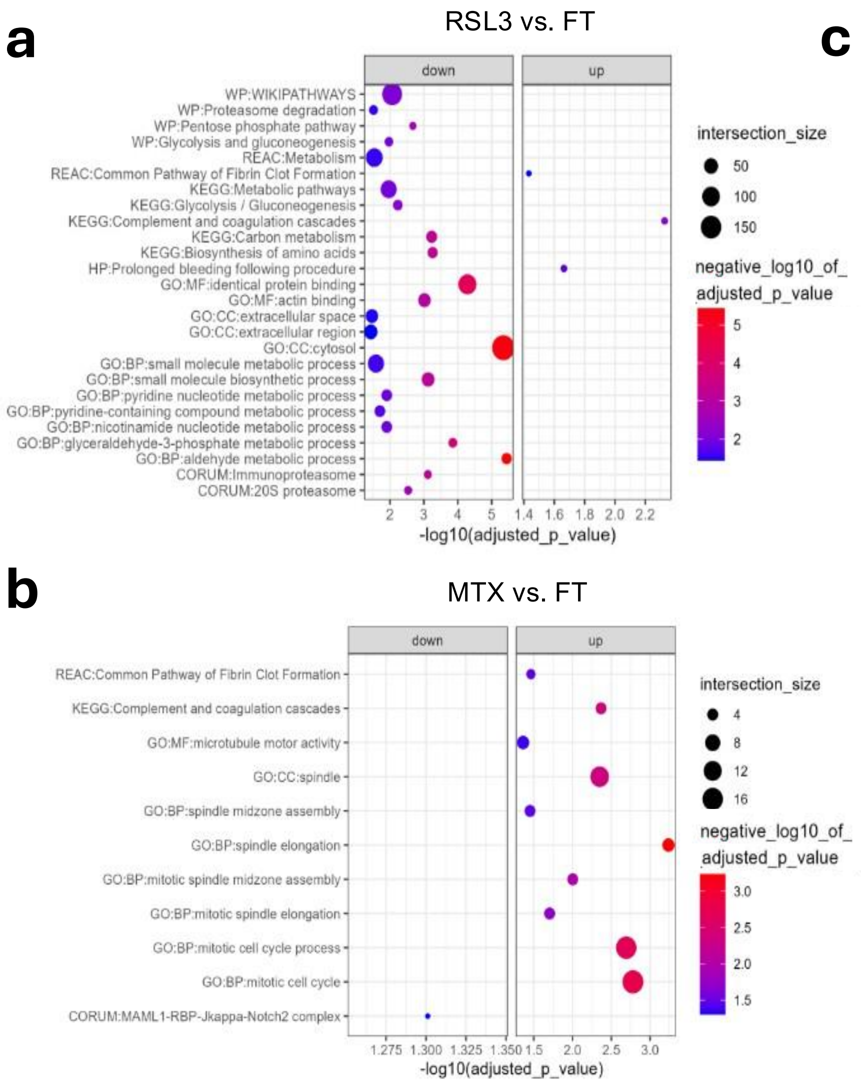

c

Up-regulated proteins which are common for RSL3 and MTX	
**MHCI**	**MHCII**
NAGPA	NAGPA
FIBG	FIBG
KIF23	KIF23
CENPE	CENPE
MMTA2	MMTA2
ANT3	ANT3
TROAP	TROAP
PRR11	PRR11
KI20A	KI20A
APOM	APOM
F13A	F13A
AURKA	AURKA
RETST	RETST
UPAR	

Peptides from these proteins which can be presented on MHCI or MHCII

289 peptides	178 peptides

Supplementary Figure 11. GO enrichment analysis of differentially expressed proteins

(a, b) Gene Ontology (GO) enrichment analysis of differentially expressed genes (DEGs) in RSL3 versus F/T lysates (**a**) and MTX versus F/T lysates **(b)**. Circle color represents the significance level of enrichment, and circle size indicates the number of enriched genes. Enrichment results with $p< 0.05$ were considered statistically significant. **(c)** Commonly upregulated proteins which contain peptides shared between RSL3- and MTX-treated lysates. Source data are provided as a Source Data file.

Supplementary Tables

Supplementary Table 1. Scale for assessing the severity of neurological deficits in mice.

Test	Parameter / Condition	Score	Description
Eyes	Protrusion of the eyeballs (exophthalmos)	0	Normal
		1	One eye with exophthalmos
		2	Both eyes with exophthalmos
Visual recognition	Placed on a 30–40 cm platform, wants to reach the table (stretching the legs to the edge of the table when lowering from a height to its surface)	0	Reaches the table - Normal surface recognition
		1	Reaches but does not touch, gropes, pulls up
		2	Does not reach, no searching movements
Reflex with irritation of the limb	Response to stimulus	0	Response present
		1	No response
Balance on plank/rod	Assessment of vestibular function (The mouse should to beable to hold on to a round rod with a diameter of 1 cm for 10 s)	0	Holds easily, can move
		1	Grasps but loses balance
		2	Cannot hold and does not grasp
Negative geotaxis	Ability to turn the body in the opposite direction on an inclined surface (45 degrees) when positioned upside down	0	Turns head upward, climbs upward
		1	Does not restore head-up position
Weight loss	Loss of body mass	0	Mouse has normal appearance
		1	Mouse is severely underweight
Whisker response	Response to whiskers. Light touch of whiskers causes the mouse to turn its head toward the stimulus. If the test is performed while holding the mouse from the back, the corresponding forelimb should move	0	Mouse responds to whisker touch (at least 1 out of 5 times)
		1	Mouse does not respond to whisker touch
Forward travel (ability to maintain directional movement	Directional movement of more than 30 cm	0	Moves straight
		1	Does not move or cannot move straight
Search behavior	Assessment of exploratory activity. The mouse is placed in the center of a circle with a diameter of 30 cm	0	Exits the circle and shows interest
		1	Does not exit immediately, hides in corners/dark areas
		2	Does not move, no exploratory activity
Grasp reflex	Ability to grab the grid with all limbs	0.5	One paw grips weaker
		2	All four paws do not grip (0.5 p. for each paw)
Body tilt	Convulsive body curvature	0	No curvature
		1	Body tilted; mouse can move, or tilt visible only when the mouse is freely suspended by the tail

		2	Pronounced body tilt (angle > 60° between forelimb and hindlimb axes); mouse cannot move
Spinal lordosis	Opposite curvature of the spine	0	No lordosis
		1	Lordosis visible, mouse can still straighten
		2	Pronounced lordosis, mouse cannot straighten
Cylinder test	Mouse in a cage or cylinder shows exploratory activity and, upon reaching an obstacle or incline, raises its forelimbs onto the wall.	0	Raises forelimbs onto the wall
		1	Shows no exploratory activity
