# Supplementary material for: Ferroptosis-armed dendritic cell vaccines for glioma immunotherapy
Source: Nat Commun. 2026 May 7;17:6158. doi: 10.1038/s41467-026-72737-6 (PMC13365579; doi:10.1038/s41467-026-72737-6)
Supplement: Supplementary file 2 — Description of Additional Supplementary Files [file 41467_2026_72737_MOESM2_ESM.pdf]

Description of Additional Supplementary Files

Supplementary Dataset 1: List of identified protein groups and corresponding quantification information per sample (n=5,165). Columns from left to right contain protein and gene IDs, protein intensities ('Reported intensity corrected' from MaxQuant output table), protein identification confidence values (i.e. Score, number of detected peptides, number of MS/MS counts).

Supplementary Dataset 2: List of quantified protein groups (n=5,162) and corresponding differential abundance analysis results for RSL3vsFT, RSL3vsMTX and MTXvsFT pairwise comparisons. Columns from left to right contain protein and gene IDs, statistical significance label (i.e. '+' for FDR < 0.05 and |log2FC|> 1), adj.p-values and log2 fold-changes per protein group and comparison. Statistical analysis was performed using moderated t-test with statistical significance for differential regulation set at an FDR cut-off value of 0.05 and a |log2(fold change)| above 1.

Supplementary Dataset 3: The following is a list of peptides that have the potential to be highly immunogenic for MHCI, along with the relevant information for each peptide (n=18,869). The columns from left to right comprises the following elements: a description, protein IDs, protein sequence, the start and stop position of the peptide, the epitope, and information regarding potential immunogenicity (including the presentation of MHCI, alleles, and immunogenicity score), and the length of the epitope.

Supplementary Dataset 4: The following is a list of peptides that have the potential to be highly immunogenic for MHCII, along with the relevant information for each peptide (n=14,573). The columns from left to right comprises the following elements: a description, protein IDs, protein sequence, the start and stop position of the peptide, the epitope, and information regarding potential immunogenicity (the presentation of MHCII), and the length of the epitope.
