# Supplementary material for: Ferroptosis-armed dendritic cell vaccines for glioma immunotherapy
Source: Nat Commun. 2026 May 7;17:6158. doi: 10.1038/s41467-026-72737-6 (PMC13365579; doi:10.1038/s41467-026-72737-6)
Supplement: Supplementary file 7 — Reporting Summary [file 41467_2026_72737_MOESM7_ESM.pdf]

nature portfolio

Corresponding author(s): Elena Catanzaro

Last updated by author(s): Jun 2, 2026

Reporting Summary

Nature Portfolio wishes to improve the reproducibility of the work that we publish. This form provides structure for consistency and transparency in reporting. For further information on Nature Portfolio policies, see our Editorial Policies and the Editorial Policy Checklist.

Statistics

For all statistical analyses, confirm that the following items are present in the figure legend, table legend, main text, or Methods section.

n/a	Confirmed	
☐	☒	The exact sample size (*n*) for each experimental group/condition, given as a discrete number and unit of measurement
☐	☒	A statement on whether measurements were taken from distinct samples or whether the same sample was measured repeatedly
☐	☒	The statistical test(s) used AND whether they are one- or two-sided<br>*Only common tests should be described solely by name; describe more complex techniques in the Methods section.*
☐	☒	A description of all covariates tested
☐	☒	A description of any assumptions or corrections, such as tests of normality and adjustment for multiple comparisons
☐	☒	A full description of the statistical parameters including central tendency (e.g. means) or other basic estimates (e.g. regression coefficient) AND variation (e.g. standard deviation) or associated estimates of uncertainty (e.g. confidence intervals)
☐	☒	For null hypothesis testing, the test statistic (e.g. *F*, *t*, *r*) with confidence intervals, effect sizes, degrees of freedom and *P* value noted<br>*Give P values as exact values whenever suitable.*
☒	☐	For Bayesian analysis, information on the choice of priors and Markov chain Monte Carlo settings
☒	☐	For hierarchical and complex designs, identification of the appropriate level for tests and full reporting of outcomes
☒	☐	Estimates of effect sizes (e.g. Cohen's *d*, Pearson's *r*), indicating how they were calculated

Our web collection on statistics for biologists contains articles on many of the points above.

Software and code

Policy information about availability of computer code

Data collection	BD FACSDiva Software for flow cytometry data collection, Orbitrap™ Tribrid™ MS Series: Instrument Control Software for proteomics data collection, ParaVision 5.1 for MRI data collection, Mabtech Apex 1.1.59.128 for ELISpot data collection, SparkControl for TECAN data collection
Data analysis	FlowJo-v10.10.0 for flow cytometry data analysis, MicroDicom DICOM Viewer 2022.3 for MRI data analysis, Mabtech Apex 1.1.59.128 for ELISpot data analysis, GraphPad Prism (version 8, 9, 10 and 11) for creating graphs and statistical analysis, Microsoft Excel for calculations, LC-MS/MS runs for all samples were processed together using the MaxQuant algorithm (version 2.2.0.0), Proteomics statistical analysis was performed in R (version 4.1.1)

For manuscripts utilizing custom algorithms or software that are central to the research but not yet described in published literature, software must be made available to editors and reviewers. We strongly encourage code deposition in a community repository (e.g. GitHub). See the Nature Portfolio guidelines for submitting code & software for further information.

Data

Policy information about availability of data

All manuscripts must include a data availability statement. This statement should provide the following information, where applicable:

- Accession codes, unique identifiers, or web links for publicly available datasets
- A description of any restrictions on data availability
- For clinical datasets or third party data, please ensure that the statement adheres to our policy

The Proteomics data generated in this study have been deposited in PRIDE database under accession code PXD060116 (https://proteomecentral.proteomexchange.org/ui?pxid=PXD060116). All data reported in this manuscript are available in the main text and supplementary information Source data are provided with this paper.

Research involving human participants, their data, or biological material

Policy information about studies with human participants or human data. See also policy information about sex, gender (identity/presentation), and sexual orientation and race, ethnicity and racism.

Reporting on sex and gender	No human participants were involved in this study.
Reporting on race, ethnicity, or other socially relevant groupings	No human participants were involved in this study.
Population characteristics	No human participants were involved in this study.
Recruitment	No human participants were involved in this study.
Ethics oversight	No human participants were involved in this study.

Note that full information on the approval of the study protocol must also be provided in the manuscript.

Field-specific reporting

Please select the one below that is the best fit for your research. If you are not sure, read the appropriate sections before making your selection.

☒ Life sciences ☐ Behavioural & social sciences ☐ Ecological, evolutionary & environmental sciences

For a reference copy of the document with all sections, see nature.com/documents/nr-reporting-summary-flat.pdf

Life sciences study design

All studies must disclose on these points even when the disclosure is negative.

Sample size	Sample size was calculated based on the previously published dataset from (Victoria D Turubanova et al, 2019, J Immunother Cancer, Immunogenic cell death induced by a new photodynamic therapy based on photosens and photodithazine and Maria V Vedunova et al, 2022, Cell Death & Disease, DC vaccines loaded with glioma cells killed by photodynamic therapy induce Th17 anti-tumor immunity and provide a four-gene signature for glioma prognosis). The required sample size was calculated based on a given α =0,05, power =0,95 using G*Power 3.1 software.
Data exclusions	No data were excluded.
Replication	We performed at least 3 biological replicates which were all reproducible.
Randomization	Mice were randomly allocated to experimental groups.
Blinding	The investigators were blinded during data collection.

Reporting for specific materials, systems and methods

We require information from authors about some types of materials, experimental systems and methods used in many studies. Here, indicate whether each material, system or method listed is relevant to your study. If you are not sure if a list item applies to your research, read the appropriate section before selecting a response.

Materials & experimental systems

n/a	Involved in the study
	☒ Antibodies
	☒ Eukaryotic cell lines
☒	☐ Palaeontology and archaeology
	☒ Animals and other organisms
☒	☐ Clinical data
☒	☐ Dual use research of concern
☒	☐ Plants

Methods

n/a	Involved in the study
☒	☐ ChIP-seq
	☒ Flow cytometry
	☒ MRI-based neuroimaging

Antibodies

Antibodies used	Annexin-V, FITC (ThermoFisher Scientific, A13199)<br>Anti-CRT-Alexa Fluor 405 (clone EPR3924, Abcam, ab210431)<br>Anti-CRT (clone FMC 75, Abcam, ab22683)<br>Anti-HMGB1 (clone EPR3507, Abcam, ab79823)<br>IgG isotype control-Alexa Fluor 405 (clone EPR25A, Abcam, ab208150)<br>IgG isotype control (clone EPR25A, Abcam, ab172730)<br>IgG1 isotype control (clone 15-6E10A7, Abcam, ab170190)<br>Anti-CD11c-APC-eFluor 780 (clone N418, ThermoFisher Scientific, 47-0114-82)<br>Anti-CD86-AF700 (clone GL1, ThermoFisher Scientific, 62-0862-82)<br>Anti-CD80-eFluor 450 (clone 16-10A1, ThermoFisher Scientific, 48-0801-82)<br>Anti-MHC class II-SB600 (clone M5/114.15.2, Biolegend, 107639)<br>Anti-CD16/CD32 (clone 93, ThermoFisher Scientific, 14-0161-82)<br>Anti-CD8a-BUV 737 (clone 53-6.7, Invitrogen, 367-0081)<br>Anti-CD4-Vio Blue (clone REA604, Miltenyi Biotech, 130-118-696)<br>Anti-CD137-PE-Vio 615 (clone REA936, Miltenyi Biotech, 130-115-572)<br>Anti-Granzyme B-PE-FITC (clone REA226, Miltenyi Biotech, 130-118-480)<br>Anti-CD44-APC-Vio779 (clone REA664, Miltenyi Biotech, 130-118-695)<br>Anti-CD62L-PerCP-Vio700 (clone REA828, Miltenyi Biotech, 130-112-840)<br>Anti-CD39-APC (clone REA870, Miltenyi Biotech, 130-114-359)<br>Anti-FoxP3-PE (clone REA788, Miltenyi Biotech, 130-111-678)
Validation	The manufacturer's recommended dilutions were used and optimized for murine cells for flow cytometry.

Eukaryotic cell lines

Policy information about cell lines and Sex and Gender in Research

Cell line source(s)	All cell lines were derived from C57Bl6 mice. GL261 murine glioblastoma cells were kindly gifted by Prof. P. Agostinis. CT-2A murine glioma cells were procured from MERK KGaA (cat. SCC194).
Authentication	None of the cell lines used were authenticated, they were provided by other labs.
Mycoplasma contamination	All cell lines tested negative for mycoplasma.
Commonly misidentified lines (See ICLAC register)	No commonly misidentified cell lines were used.

Animals and other research organisms

Policy information about studies involving animals; ARRIVE guidelines recommended for reporting animal research, and Sex and Gender in Research

Laboratory animals	Mus Musculus, C57Bl6/J, female, aged 6 weeks were used in this study
Wild animals	No wild animals were used in this study.
Reporting on sex	Sex was not considered in this study. Only female mice were used for practical reasons.
Field-collected samples	This study did not include samples collected from the field.
Ethics oversight	All in vivo experiments were conducted according to the guidelines of the local Ethics Committee of Ghent University.

Note that full information on the approval of the study protocol must also be provided in the manuscript.

Plants

Seed stocks	No plants were used in this study.
Novel plant genotypes	No plants were used in this study.
Authentication	No plants were used in this study.

Flow Cytometry

Plots

Confirm that:

☒ The axis labels state the marker and fluorochrome used (e.g. CD4-FITC).

☒ The axis scales are clearly visible. Include numbers along axes only for bottom left plot of group (a 'group' is an analysis of identical markers).

☒ All plots are contour plots with outliers or pseudocolor plots.

☒ A numerical value for number of cells or percentage (with statistics) is provided.

Methodology

Sample preparation	Cultured cells (See part Eukaryotic cell lines, cell line source(s)) were collected, incubated with Fc-blocker (anti-CD16/32) and stained with antibodies.
Instrument	BD LSRFortessa and BD Symphony A5 were used.
Software	FlowJo-v10.10.0 was used for flow cytometry data analysis.
Cell population abundance	Cell sorting was not performed. 10 000 events were recorded from the population of interest.
Gating strategy	Cell debris, identified as events with low SSC-A and FSC-A values in the main population of events on the SSC-A vs FSC-A dot plots, are discarded. Single cells are identified on FSC-H vs FSC-A dot plot as the main population proportional to the FSC-H vs. FSC-A. Viable cells were selected by the Sytox Red (APC) or Sytox Green (FITC) or Sytox Blue (Bacific Blue) or Zombie UV (UV450) vs SSC-A plot as negative population based on the histogram. All gating strategies are provided in the Supplementary information.

☒ Tick this box to confirm that a figure exemplifying the gating strategy is provided in the Supplementary Information.

Magnetic resonance imaging

Experimental design

Design type	MRI was performed once to determine the presence of tumor after inoculation.
Design specifications	At least 3 mice from each group had MRI. A representative scan was selected for the figure.
Behavioral performance measures	MRI was done to visualize the presence/absence of an intracranial tumor. Statistical criteria are not applicable.

Acquisition

Imaging type(s)	Structural T1-weighted scan with contrast
Field strength	7 Tesla
Sequence & imaging parameters	Repetition time (TR) of 1453 ms; echo time (TE) of 9.1 ms; four averages; echo train length of 4; field of view of 3 × 2.4 cm; matrix of 200 × 200; and 30 contiguous slices with a thickness of 600 µm.
Area of acquisition	Whole brain scans, which captures the tumour area

Diffusion MRI ☐ Used ☒ Not used

Preprocessing

Preprocessing software	MRI was done to visualize the presence/absence of an intracranial tumor. Preprocessing is not applicable.
Normalization	MRI was done to visualize the presence/absence of an intracranial tumor. Normalization is not applicable.
Normalization template	MRI was done to visualize the presence/absence of an intracranial tumor. Normalization is not applicable.
Noise and artifact removal	MRI was done to visualize the presence/absence of an intracranial tumor. No noise or artifact removal were done.
Volume censoring	MRI was done to visualize the presence/absence of an intracranial tumor. The volume was not analysed.

Statistical modeling & inference

Model type and settings	MRI was done to visualize the presence/absence of an intracranial tumor. No models were used.
Effect(s) tested	MRI was done to visualize the presence/absence of an intracranial tumor. No models were used.

Specify type of analysis: ☒ Whole brain ☐ ROI-based ☐ Both

Statistic type for inference (See Eklund et al. 2016)	MRI was done to visualize the presence/absence of an intracranial tumor. Statistical criteria are not applicable.
Correction	MRI was done to visualize the presence/absence of an intracranial tumor. Correlation criteria are not applicable.

Models & analysis

n/a	Involved in the study
☒	☐ Functional and/or effective connectivity
☒	☐ Graph analysis
☒	☐ Multivariate modeling or predictive analysis
