# Supplementary material for: Ferroptosis-armed dendritic cell vaccines for glioma immunotherapy
Source: Nat Commun. 2026 May 7;17:6158. doi: 10.1038/s41467-026-72737-6 (PMC13365579; doi:10.1038/s41467-026-72737-6)
Supplement: Supplementary file 8 — Transparent Peer Review file [file 41467_2026_72737_MOESM8_ESM.pdf]

nature portfolio

Peer Review File

Ferroptosis-armed dendritic cell vaccines for glioma immunotherapy

Corresponding Author: Dr Elena Catanzaro

This file contains all reviewer reports in order by version, followed by all author rebuttals in order by version.

Version 0:

Reviewer comments:

Reviewer #1

(Remarks to the Author)
Saviuk et al. demonstrated that dendritic cell (DC) loaded with ferroptotic lysates effectively protect mice against tumor growth in orthotopic glioma models, inducing the production of IFN-γ in response to tumor antigen stimulation, and exhibiting significant therapeutic efficacy. The intrinsic immunogenicity of ferroptosis was independent on the type of glioma or the ferroptosis inducers. The effectiveness of DC vaccines and ferroptosis immunogenicity depended on the presence of calreticulin and ATP but not on HMGB1-TLR4 signaling.

This is a first report demonstrating that tumor cell lysate from tumor cells undergoing ferroptosis can generate immune response. The study reports several interesting facts: weak activation DCs by the lysate from tumor cells killed by ferroptosis, different transcriptional profile in DC induced by different lysates, clear preventive effect of DCs loaded with ferroptotic lysates, calreticulin and ATP involvement in ICD. These are all interesting facts. Authors conclusion that ferroptotic cell death can generate antitumor immune response is justified. The possibility that it causes ICD is suggestive, although it was not clearly demonstrated due to lack of data documenting generation of tumor-specific T cell responses. However, some question/issues need to be addressed.

The major concern with this study is whether ferroptosis mediated killing of tumor cells generates special antigens that provide for potent protection. It is entirely possible that it is indeed the case. However, it also equally possible that antitumor response is the just a result of loading DCs with tumor cell derived proteins regardless of the nature of cell death. And connected to that, whether ferroptosis mediated cell death is superior to other types of cell death in generating antitumor immune response. This question can be answered by various ways. The most obvious one is by comparing antitumor activity of DCs loaded with lysates of tumor cells killed by different ways. Authors demonstrated different transcriptional profile by comparing the effect of lysates from ferroptotic, apoptotic, and necrotic tumor cells (Fig. 4H). However, when they evaluated side by side lysates from ferroptosis and apoptosis induced cell death no differences were seen. This raises question about conclusions and interpretation of the study.

Comparison with necrotic cell death lysate was not entirely accurate since this type of cell death does not lead to activation of DCs. In the multiple previous studies, potent antitumor effect of tumor cell lysates loaded DCs was achieved when DCs were activated with various TLR agonists or CD40 that would lead to up-regulation of MHC class I and co-stimulatory molecules. This was not done in this study. In the absence of this treatment, DCs were simply not activated and comparison with ferroptosis induced lysates was not convincing since ferroptosis as authors showed caused modest up-regulation of DCs. To follow on the previous point, RSL3 induced less activation of DCs than LPS. Why don’t authors try to compare the effect of different vaccines (RSL3, MTX, necrosis) in LPS activated DCs. Maybe it would allow them to observe more differences in antitumor activity between apoptosis and ferroptosis.

Analysis of T cell response was very limited. No comparison with MTX induced cell death was performed and no difference between FT and RSL3 induced cell death was seen.

The effect of vaccination in a weakly immunogenic CT-2A tumor was very small raising the question of biological impact of this source of antigens. The same concern is with Figure 3. Effects are too small for strong conclusions made by the authors.

One of the most exciting questions is the reason for potential specificity of RSL3 induced cell death associated proteins. If

authors could assess proteins in some fashion (for instance proteomics) this would enhance the impact of the study.

Reviewer #2

(Remarks to the Author)
Saviuk et al. described the study of DC vaccine development via activation with ferroptotic glioma cells. I focused my review on the proteomic aspect of the research. Proteomic analyses compared glioma cells treated with RSL3 (ferroptosis inducer) or MTX and compared to control cells. The authors show the results of this comparison in Figure 4H. Overall, the description of the results and the methodology are too minimalistic to understand the contribution of these data to the manuscript. I list below the specific comments:
1. The manuscript lacks a description of the proteins that significantly change between treatment groups. The heatmap (Fig. 4H) hardly gives any information, as it is impossible to see the protein names. The authors should expand the description to show the changing proteins and biological processes. In addition, they should add the list of significantly changing proteins as supplementary material.
2. Following the previous comment, the authors should discuss these results also in the Discussion section.
3. Supplementary material should include an Excel file with all identified proteins (MaxQuant output table).
4. The methods section should provide more details about the sample preparation, data acquisition, and data analysis. Missing information includes TMT, peptide pre-fractionation, length of runs, MS acquisition parameters, and the details of the maxquant parameters. Default settings might change from one version to the other and should be explicitly detailed. All data processing steps should also be added, such as normalization, imputation, etc.

Reviewer #3

(Remarks to the Author)
Summary
Here, Saviuk et al., reports the use of ferroptotic cell death to generate dendritic cell-based vaccines with increased anti-tumor activity compared to other form of cell death. The authors are the first to investigate the role of this type of cell death in vaccine development. They demonstrated the anti-tumor activity using a mouse model for intracranial glioma. Mechanistically, the authors reveal that the increased anti-tumor effect is dependent on three DAMPs: ATP, CRT and HMGB1. Additionally, the authors also showed long term protection of the anti-tumor effect and no added-value of the vaccine adjuvant LPS. The results are well presented and clearly described. The data is relevant and solid. The claims of this manuscript are well supported by the data. However, few concerns should be addressed before publication. Mainly, the authors should investigate further the mechanisms underlying the increased anti-tumor activity and fully report the proteomics data. The proteomics data might be a valuable resource to investigate further the mechanisms at play.

Major comments:
Major comment 1
The proteomics should be fully analyzed and presented. The method section is completely absent from the manuscript. Thus is is impossible to evaluate the quality of the data. The protein table is also not shared. The data should be deposited to a public proteomics repository. Additionally analysis should be performed: gene ontology, protein network analysis. The authors shows the differential regulation with a heatmap (what type or comparison? Pair-wise? What is the overlap between the conditions? The expression level of CRT and HMGB1 should be shown.

Major comment 2
Overall, this is very nice work that significantly contributes to the state of the art of DC vaccine development. However, I do feel that the molecular mechanisms driving the effects of the ferroptotic lysates could have been studied more in-depth, for instance by exploring the proteomics dataset in more detail. (FT) Could the effect of ferroptotic lysate be mimicked by adding ATP, recombinant HMGB1 and recombinant CRT in the lysate with cell death induced by freeze and thaw (FT-DC) or with MTX-induced lysate? Alternatively, other experimental design could be envisionsed. For instance, the authors could use ferroptotic cell death in mouse cells and mix this lysate with the glioma cell line GL261-FT (after freeze and thaw). One would expect that the induced DAMPs are sufficient for the increased stimulation of DC while the GL261 lysate will bring the (potentially) required antigens. Mechanistically, the authors should comment why they investigated only a subset of the ICD-associated DAMPs (CRT, ATP and HMGB1)? The authors should investigate for more or provide a rational for the selection of these DAMPs and the exclusion of the others DAMPs? Is this selection motivated by the proteomics data?

Major comment 3
The mechanisms associated for increased anti-tumor effects have been partially elucidated. However, the authors only focus on the adjuvant effect with LPS and few DAMPs. What about the antigenicity? Which tumor-associated antigens (TAA) or tumor-specific antigen (TSA) are driving the immune response against the tumor? The authors initiated this investigation with their proteomics data. However, they could further explore major histocompatibility presentation of the tumor antigens. This could be performed in targeted manner using known TAA/TSA or rather untargeted using mass spectrometry-based

immunopeptidomics. Both MHC-II and MHC-I (cross-presentation) seems relevant in this model. Additionally, ferroptosis-induced cell death might reveal relevant antigens compared to other form of cell death.

Minor comments

What could be the advantage of the use of ferroptotic lysates compared to the DC vaccines that are currently being used in the clinic? Which types of lysates are currently being used? Some more context about this could be added to the introduction and/or the first paragraphs of the discussion.

Figure 1: Based on the M&M sections, no solvent controls appeared to be used when using cell death-inducing or -inhibiting agents. Additionally, it was not mentioned in which solvent(s) the chemical compounds were dissolved. Solvents such as DMSO can already exert significant effects on cells, and can induce cell stress and even cell death when used at high final concentrations, acting as a confounder for the effect of the actual compounds. Ideally, these experiments should be repeated with proper solvent controls. Alternatively, the effect of the solvent alone should be tested.

In Fig1: the authors tested the induction of ferroptotic cell death in two glioma cell lines. Why those two cell lines have a different response. Although this was briefly eluded in the text, it remains unclear. Could the authors comment whether it would be relevant to rescue the low anti-tumor effect in CT2A cells? May be, these cells have a reduced expression of DAMPs such as CRT or HMGB1? If yes, another proteomics investigation could be envisioned to reveal this. Alternatively, the reduced anti-tumor effect might be driven by low expression of MHC I in CT-2A cells, could the authors comment whether rescuing low MHC-I expression with transduced overexpression be relevant?

Figure 1B: The effect RSL3 on CT-2A cells appears to be dose-independent. Is there a potential explanation for this?

Figure 1E-H: What is the effect of SAS on GSH and lipid peroxidase levels in the two cell lines?

Figure 2: for me, as a non-expert in in vivo studies, it is unclear why reaching the humane endpoints (or not) determines whether or not the mice were included in the analysis. As a side note, it would also be interesting to know whether there's statistically significant differences between the RSL3, FT and MTX groups.

Fig. 2-3: The statistical evaluation of neurological deficits should not be done via multiple unpaired t-tests, as these do not correct for multiple comparisons. All groups should be compared in one statistical test, such as one-way ANOVA. Were the Mantel-Cox tests done on all groups at once, or was each experimental group also separately compared to the PBS here?

The authors state the following regarding the data in Fig. 3G: "This response was observed in the ferroptotic lysate group and was not observed in control groups vaccinated with PBS, DC alone, or DC vaccines based on non-ICD lysates". The data do not fully support this statement, as there is no significant difference between the empty DC, DC-GL261-SIINFEKL-FT and DC-GL261-SIINFEKL-RSL3. Similar claims are being made in the discussion section.

Did the authors investigate whether the different lysate (e.g. freeze thaw) have a lower amount of ATP, CRT, HMGB1?

To appreciate better the added value of DC-GL261-RSL3, DC-GL261-MTX should be consistently included in the whole manuscript. It is unclear why this was included in some experiments but not in other.

Fig. 4A-C: these data should be analyzed via two-way ANOVA.

The Figure 4E-G shows two key data. (1) LPS has no added value in vivo for anti-tumor activity (Fig 4F) and (2) vaccinated mice surviving the initial tumor challenge show a prolonged immune response after second tumor challenge at day 56. This is nice data but this would have been more appreciated on wild-type DC vaccine instead of TLR4- KO DC vaccines.

In figure 4F, as mentioned above, the proteomics data should be deposited in a proteomics repository such as PRIDE. This data deserves more investigation. The authors only looked at the proteins exclusively regulated. What is the overlap between the conditions? Gene ontology? If relevant (Figure 5?), the authors could show the expression level of HMGB1 and CRT with a bare plot, a s-curve showing log2 IBAQ intensity and the rank protein according to abundance. Additionally, a schematic to describe the experimental design could be included.

The authors mentioned TSA. Is this related to specific mutations? Or non-canonical protein? What are the known TSA in this glioma model?

The authors should consider combining immunopeptidomics with the proteomics. It will be strongly appreciated if the authors attempt to reveal the cellular immunopeptidome presented on MHC class II and MHC class I (cross-presentation) by the DC vaccines.

Suggestion: May be figure 4H should be move to a separate figure as it is not related to the investigation of LPS. Of note, figure 4H is a bit related to figure 5 where the authors investigate the antigenicity of they ferroptotic DC vaccines.

In Fig. 5, the authors observe that CRT expression is actually much more increased with MTX than with RSL3. A similar comparison between RSL3 and MTX should be done for ATP and HMGB1. Furthermore, more detailed explanation/exploration is missing about how the authors link the stronger increase of CRT with MTX to the lower effectiveness of MTX lysates in vivo. Likely, CRT is only one factor driving the efficacy of the ferroptotic DC vaccines in vivo. Further analysis of the proteomics data might be helpful to reveal additional mechanisms/factors.

Fig 5A what is the control? The authors should repeat the experiment but including the freeze-thaw control “FT” condition (non-ICD).

Fig 5B shows the FACS related ot figure fig 5A. to be complete, the authors should show all the same groups for fig 5A and fig 5B.

In Figure 5, the authors hypothesize that the superior protection of the ferroptotic lysates is among others driven by ATP and CRT. However, CRT upregulation is much more pronounced in MTX-treated lysates, yet these MTX-treated lysates are less effective in vivo, when loaded onto DCs. How could these observations be explained?

Figure 5C and 5D should also include the condition with MTX to be consistent. It would be interesting to also test FT to evaluate the effect of non-ICD.

The experiments in figure 5E-I are very impressive with very nice data. This data is already very convincing and suggest that all components are required for the increased anti-tumor effect. Did the authors consider reconstituting the increased anti-tumor effect using the DC-GL261-FT supplemented with HMGB1, CRT and ATP? The data suggests that removing one of these three components is sufficient to block the increased anti-tumor effect. However, one could speculate that other DAMPs required for this effect? Thus, it seems worthwhile to investigate whether adding those three DAMPs could be sufficient for the increased anti-tumor effect

Line 309: “The emission of these three key DAMPs may function as additional immunostimulatory adjuvants in DC vaccines.” The experiments in figure 5E-I are very impressive. This data suggest that the three components are required for the increased anti-tumor effect. Did the authors consider reconstituting the increased anti-tumor effect using the DC-GL261-FT supplemented with, CRT and ATP? but also HMGB1? The data suggests that removing one components is sufficient to block the increased anti-tumor effect. Additionally, one could speculate that other DAMPs required for this effect? Thus, it seems worthwhile to investigate whether these DAMPs are sufficient for the increased anti-tumor effect. Adding these DAMPs could be sufficient to restore the increased anti-tumor effect. If not, there might be other DAMPs required for the increased anti-tumor effect. Those unknown DAMPs might be hidden in the available proteomics data.

Did the authors consider investigating whether CD8 or CD4 T cells are expected to be most relevant for ferroptotic DC vaccines? What happens if CD8 and CD4 t cells are functionally blocked with antibody.

Reviewer #4

(Remarks to the Author)
I co-reviewed this manuscript with one of the reviewers who provided the listed reports. This is part of the Nature Communications initiative to facilitate training in peer review and to provide appropriate recognition for Early Career Researchers who co-review manuscripts.

Reviewer #5

(Remarks to the Author)
This is an interesting manuscript that could have implications for dendritic cell vaccine design. The authors are subjecting the experimental glioma cell lines, GL261 and CT-2A to various death inducing agents. This results in these gliomas undergoing cell death by apoptosis, necroptosis, and ferroptosis. The resulting apoptotic gliomas are then co-cultured with dendritic cells (DCs) before transfer as DC based vaccines. It is determined that ferroptosis DC vaccination resulted in enhanced survival prophylactically and when administered immediately after tumor inoculation. Additional strategies to demonstrate the independence from LPS adjuvant but reliance on calreticulin, ATP, and potentially HMGB1 DAMPs is put forward. These are the strengths of the work.

The weaknesses of the study include:
1. The underpowered study in Figure 2C which does not determine if DC vaccine with RSL3 (ferroptosis) is advantageous over DC vaccine with MTX (immune cell death). This is important to know, as this is of importance for future DC vaccine based design studies.
2. In Figure 2E and 2H, the MRIs shown have animals in which the gliomas have grown outside the skull. It is unclear if the glioma presents this way there would be an effect on immunotherapy efficacy.
3. Figure 3A - The administration of immunotherapy at Day 2 post glioma inoculation is VERY early. This is well before there is an MRI detectable glioma in these animals.
4. The studies in Figure 3 end at day 40. This is an early endpoint, as tumor recurrence is common in these models. Are these animals cured long term?
5. In Figure 3F and G, there is considerable variability in the ELISpot analysis. It would make sense to track SIINFEKL antigen specific CD8 T cells with peptide:MHC tetramers in the lymph nodes. Additional analysis with increased brain infiltration of CD8 T cells targeting this model antigen would be informative too.
6. In Figure 4 , the analysis of LPS is informative negative data. However, it appears that Figure 5F is lacking wild type mouse derived DC controls in addition to the TLR4 KO mouse DC groups. Figure 4G needs a control for glioma induced mortality.
7. The data in Figure 5F is interpreted as HMGB1 not being required for ferroptosis DC vaccine efficacy. However, the survival curve implies this conclusion is based on an underpowered experiment.
8. The graphical abstract in Figure 6 is more confusing than useful. The concept of ferroptosis DC vaccine is more clearly written than illustrated.

Version 1:

Reviewer comments:

Reviewer #1

(Remarks to the Author)
Authors addressed all my concerns and questions. I have no additional comments and think this is a strong report.

Reviewer #2

(Remarks to the Author)
The authors addressed my comments and added all the necessary information regarding the proteomics data analysis. However, their revision raised some additional questions. The authors added a prediction of the MHC-presented peptides. They used the MHCnuggets tool to predict the presented peptides on MHCI and MHCII. In the supplementary Tables the authors share 18K and 14K peptides that are predicted to be presented and elicit immune response. Given the known high error rates of many of these algorithms, it seems unlikely that these predictions are correct and it is not convincing that the list of potential MHC peptides is reliable. It is also uncommon to use a single predictive tool and gain any insights. In addition, as the authors themselves indicate in their response to reviewers, the neoantigens that are elevated in the ferroptotic cells may harbor ferroptosis/redox-related modifications. Altogether, the logic of these analyses is not clear- The prediction tools are not expected to unravel the modified peptides, and these modifications are likely to be specific to ferroptosis and may markedly differ from the apoptosis-induced neoantigens. Thus the suggested similarity between these pathways is not convincing.

Reviewer #3

(Remarks to the Author)
Reviewer 3:

I thank the authors for the answers to my comments. I appreciate the format of the rebuttal letter very much. The data presented here is of good quality and the claims and conclusions are supported by the data. However, the link between the proteomics data and the mechanistic study is elusive. Indeed, despite the authors identified potential determinants mediating the increased immunogenic response (figure 7), they decided to focus on the canonical DAMPs (CRT, ATP and HMGB1). Investigating this DAMPs in ICD immunogenicity is very relevant, although the link with the proteomics data is not strong. Preliminary or negative data testing the newly identified proteins (or antigens) will be of great interest. Namely, the proteins

indicated in figure 7E could have been tested. They could envision to test these candidates with immunoprecipitation to deplete the ICD lysate and prepare new DC vaccines. Alternatively, The authors might consider including the "Additional figure 1" in their manuscript. This immunopeptidomics data might deserve to be published in part or in full. Overall, this manuscript is of good quality and deserves to be published.

Comment 1: Figure 7: proteomics
The authors shows a heatmap in figure 7B. What statistical test was performed? Is this a one-way ANOVA? This should be indicated in legend and method section.
Figure 7C-E the number of peptides presented on MHC I and MHC II is indicated in the protein volcano plots. Mixing experimental protein-level data with predicted (immuno-)peptides data is confusing. Overall, the take home message is not clear from the figure. Does this author want to highlight the high number of presented peptides in one of the conditions? The authors could consider separating the protein-level and peptide level data.
Comment 2: Figure 7: proteomics
The results section nicely describes the data on the prediction of the MHC-I or -II presented peptides. However, the figure is not clear. The author could also consider to include the prediction of presented peptides in another figure.
The authors should consider re-phrasing “immunogenic peptides” to peptides presented on MHC-I or -II molecules. Indeed, most MHC-presented peptides are not immunogenic (i.e. triggering T cell activation).

Comment 3: Sup Figure 10: reconstitution of DAMPs in FT lysates
The data is impressive and add value to this manuscript by dissecting the mechanism at play. Figure 8 clearly shows the importance of those canonical DAMPs. Thus, answering a key question in the field of ICD and DC vaccination. Additionally, Sup. Figure 10 shows that individual reconstitution of the DAMPs is not sufficient to induce the immunogenic response from RSL3 lysate. Together, this data shows that while these DAMPs are essential, they are not sufficient for the immunogenic response mediated by ICD.
The authors should consider adding this concept to the abstract.
The authors could consider switching figure 7 and 8 since the figure 7 test the hypothesis that ATP, CRT and HMGB1 are important for the immunogenicity of ICD while the figure 8 shows data suggesting novel molecular determinants mediating the immunogenicity of ICD.

Comment 4:
I thank the authors for clarifying the aim of this study. I agree with data and results description. Figure 8E, the authors indicated a different -p-value between the text (line 394) and the figure 8E. Could the authors comment or adjust which p-value is correct.

Comment 5:
I thank the authors for generating the new data. They used in silico prediction to identify the potentially presented peptides on MHC-I and -II. This data is interesting but the presentation of this data in figure 7 is unclear. They authors should consider splitting the experimental protein-level data from the predicted (immuno-) peptide data.
Additionally, the authors should consider using the “additional figure 1” to validate the proteomics data in figure 7. Although not perfect, this data seems relevant and could shed light on which antigens have a role in the ICD immunogenicity. Adding this data to the manuscript will also improve the value of this paper by further suggesting which antigens might be relevant for ICD immunogenicity.

Reviewer #4

(Remarks to the Author)
I co-reviewed this manuscript with one of the reviewers who provided the listed reports. This is part of the Nature Communications initiative to facilitate training in peer review and to provide appropriate recognition for Early Career Researchers who co-review manuscripts.

Reviewer #5

(Remarks to the Author)
The authors have made a concerted effort to address my prior critique. I have no further comments.

Version 2:

Reviewer comments:

Reviewer #2

(Remarks to the Author)
The authors address my comment regarding the prediction of antigen presentation. While I still think this is a rather weak part of the manuscript, I also acknowledge that addressing this appropriately is beyond the scope of this work.

Reviewer #3

(Remarks to the Author)

The authors addressed all my concerns. I have no additional comments.

Reviewer #4

(Remarks to the Author)
I co-reviewed this manuscript with one of the reviewers who provided the listed reports. This is part of the Nature Communications initiative to facilitate training in peer review and to provide appropriate recognition for Early Career Researchers who co-review manuscripts.

Reviewer #6

(Remarks to the Author)
In the revised manuscript "Ferroptosis-armed dendritic cell vaccines for glioma immunotherapy" (#NCOMMS-25-07518B), Mariia Saviuk and co-workers provide experimental evidence for the immunogenicity, in terms of both adjuvanticity and antigenicity, of ferroptosis and set the route for taking advantage of it in the design of DC-based vaccines to treat gliomas. The message of the article is important, novel and within scope for Nature Communication. The manuscript is well written, precise and easy to follow. Findings are abundant, technically diverse and well presented. However, some evidence from the series of in vitro and in vivo (mouse) experimentation is not yet compelling. Hence we (my co-reviewer and I) are listing a number of concerns and making suggestions that should enhance the manuscript. Given the great future importance of these studies, every possible artifact and misinterpretation must be ruled out. In summary, we believe the article by Mariia Saviuk and colleagues represent a strong candidate for publication in Nature Communication upon revision.

Major point 1: Figure 2C. These reviewers are surprised to see MTX-induced ICD protecting only the 30-35% of animals. Also, in Figure 4 and 6, MTX does not seem to be so immunogenic (low vaccination potential and low % of effector and memory T cells). This can be due to intrinsic or acquired resistance of gliomas to MTX. Verification of the induction of ICD with other ICD-inducers such as doxorubicin or oxaliplatin would be very useful and reassuring.

Major point 2. Figures 3B and 4B. According to the author model, therapeutic use of DC-GL261-RSL3 provide benefit better than any other therapeutic vaccine tested. However, this strategy seems to work better when tumors are already in progression (7 days after inoculation) than when tumors are in early development (2 days after inoculation) with survival rates of 40% at 60 days after tumor inoculation in the first case and 10% at 40 days after tumor inoculation in the second case. How do the authorsexplain this discrepancy?

Major point 3. Figure 5G,H. By using TLR4-/- DCs the authors are intended to outline that even if LPS can work as an adjuvant in DC maturation, it does not affect DC ability to mount a protective immune response in DC-GL261-RSL3 vaccines. However, treatment of TLR4-/- DCs with LPS is a redundant control and add less. More interesting and convincing is the experiment showed in Supplementary Figure 3E-G. In the opinion of these reviewers, it is surely better to show data in Supplementary Figure 3E-G as main data and data in Figure 5F-H as supplementary data.

Major point 4. Figure 6E. Additional experiments, such as blocking of CD39, are required to verify the features of CD44highCD62L-CD39+ cells as a newly discovered tissue-resident memory T cell population.

Major point 5: Figure 7. What is the rationale to investigate these selective alarmins? Why have the authors excluded the role of other DAMPs released during ICD such as IFNs-I (PMID: 25344738) and Ann-I (PMID: 26516201), the last being involved in DC-dying cancer cell juxtaposition?

Major point 6: Figure 7A and discussion. The authors see CRT surface exposure at a very early time point (1.5 hours)and, as they discuss, this finding is at odds with previous literature. How do they interpret these data? Could they be more accurate in the discussion?

Major point 7: Figure 8. The authors provide data on the role of ferroptosis in molding the peptidome of cancer cells. However, as the authors admit, this is a chapter of another story. Since there is no functional data showing the immunogenicity of ferroptotic cancer cell peptides, can the authors either provide at least a list of tumor-specific and/or tumor-associated antigens among the 289-MHC-I-restricted peptides to have an idea of non-self molecules that can be able to mount an anti-tumor immune response and even better to synthetize a couple of them and test through DC-mediated T cell cross-priming (but these authors understand that antigen synthesis and testing may be out of the scope) or remove it and write a second story?

Major point 8: material and methods. Why do the authors exclude IL-4 to differentiate DCs from bone marrow cells?

Minor point 1: Figure 1A-D. Please add control group to each plot (also a dotted line as in panels E-H).

Minor point 2: Supplementary Figure 4. Cell populations in the plots showing surface expression of CD80, CD86 and MHC-II are flattened on the right side. Please check the sample analysis and correct the plots moving the populations on the center to increase in value data.

Minor point 3: Supplementary Figure 8D. Please show graphs with isotypes before and graphs with specific Abs after.

Minor point 4: line 270. Please correct “the synthetic the MHC” with “the synthetic MHC”.

Minor point 5: line 333. Please correct “CD44lowCD62L- effector memory cells” with “CD44highCD62L- effector memory cells”.

Minor point 6: line 368. Please add “E” to “Fig.7”.

Minor point 7: line 450. Please correct “DAMPs release” with “DAMP release”.

Minor point 8: line 460. Please add “)” after “unregulated cell death59”.

Minor point 9: line 552, authors use reference 46 to support the concept of lipid peroxidation-induced modifications of proteins and lipids. Reference 46 is a review and hence does not support the author sentence. Please add the reference of an original article showing that.

Minor point 10: line 614. Please add “)” after “non-oxidized lipids”.

Minor point 11: line 875. Please remove the space between IFN- and γ.

Minor point 12: line 1102. Please correct “DCs maturation” with “DC maturation”.

Minor point 13: legend graphical abstract. Please insert panels 1, 2 and 3. Only sub-panels a, b, c of panel 2 are described.

Reviewer #7

(Remarks to the Author)
I co-reviewed this manuscript with one of the reviewers who provided the listed reports. This is part of the Nature Communications initiative to facilitate training in peer review and to provide appropriate recognition for Early Career Researchers who co-review manuscripts.

Version 3:

Reviewer comments:

Reviewer #6

(Remarks to the Author)
The authors addessed all my concerns. I have no additional comments and I recommend for pubblication.

Reviewer #7

(Remarks to the Author)
I co-reviewed this manuscript with one of the reviewers who provided the listed reports. This is part of the Nature Communications initiative to facilitate training in peer review and to provide appropriate recognition for Early Career Researchers who co-review manuscripts.

Open Access This Peer Review File is licensed under a Creative Commons Attribution 4.0 International License, which permits use, sharing, adaptation, distribution and reproduction in any medium or format, as long as you give appropriate credit to the original author(s) and the source, provide a link to the Creative Commons license, and indicate if changes were made.
In cases where reviewers are anonymous, credit should be given to 'Anonymous Referee' and the source.
The images or other third party material in this Peer Review File are included in the article's Creative Commons license, unless indicated otherwise in a credit line to the material. If material is not included in the article's Creative Commons license and your intended use is not permitted by statutory regulation or exceeds the permitted use, you will need to obtain permission directly from the copyright holder.
To view a copy of this license, visit https://creativecommons.org/licenses/by/4.0/

Response to the Reviewers: Point-by-point reply

Reviewer #1

"Saviuk et al. demonstrated that dendritic cell (DC) loaded with ferroptotic lysates effectively protect mice against tumor growth in orthotopic glioma models, inducing the production of IFN-γ in response to tumor antigen stimulation, and exhibiting significant therapeutic efficacy. The intrinsic immunogenicity of ferroptosis was independent on the type of glioma or the ferroptosis inducers. The effectiveness of DC vaccines and ferroptosis immunogenicity depended on the presence of calreticulin and ATP but not on HMGB1-TLR4 signaling.
This is a first report demonstrating that tumor cell lysate from tumor cells undergoing ferroptosis can generate immune response. The study reports several interesting facts: weak activation DCs by the lysate from tumor cells killed by ferroptosis, different transcriptional profile in DC induced by different lysates, clear preventive effect of DCs loaded with ferroptotic lysates, calreticulin and ATP involvement in ICD. These are all interesting facts. Authors conclusion that ferroptotic cell death can generate antitumor immune response is justified. The possibility that it causes ICD is suggestive, although it was not clearly demonstrated due to lack of data documenting generation of tumor-specific T cell responses. However, some question/issues need to be addressed."

We sincerely thank the Reviewer #1 for the positive assessment of our work. We appreciate the suggestion to include additional experiments to further support our conclusions. We have performed the suggested experiments.

Comment 1. *"The major concern with this study is whether ferroptosis mediated killing of tumor cells generates special antigens that provide for potent protection. It is entirely possible that it is indeed the case. However, it also equally possible that antitumor response is the just a result of loading DCs with tumor cell derived proteins regardless of the nature of cell death. And connected to that, whether ferroptosis mediated cell death is superior to other types of cell death in generating antitumor immune response. This question can be answered by various ways. The most obvious one is by comparing antitumor activity of DCs loaded with lysates of tumor cells killed by different ways. Authors demonstrated different transcriptional profile by comparing the effect of lysates from ferroptotic, apoptotic, and necrotic tumor cells (Fig. 4H). However, when they evaluated side by side lysates from ferroptosis and apoptosis induced cell death no differences were seen. This raises question about conclusions and interpretation of the study."*

Answer 1. It is important to note that apoptosis is a well-established form of immunogenic cell death (ICD) (Kroemer et al., 2022). Vaccines based on apoptotic lysates or apoptotic cells have been extensively tested *in vivo* (Apetoh et al., 2007; Casares et al., 2005; Goldszmid et al., 2003; Kokhaei et al., 2004; Obeid et al., 2007). Therefore, we used apoptotic lysates derived from immunogenic apoptotic cells as a positive control in our study.
In addition, in our study we also included a negative control group in which the viable glioma cells (*e.g.,* GL261 or CT2A were subjected to several cycles of freeze-thawing, leading to accidental necrosis - *i.e.,* cell death without the induction of a specific regulated cell death pathway). It has been demonstrated that tumor cells undergoing such freeze-thaw cycles are not immunogenic (Aaes et al., 2016; Efimova et al., 2020; Krysko et al., 2017; Tirapu et al., 2008; Turubanova et al., 2019).
Importantly, a similar physical disruption of glioma cells was used in a recent clinical trial involving DC vaccines for immunotherapy of glioblastoma (Crespo-Diaz et al., 2011; Liau et al., 2023; Parney et al., 2022). Therefore, we believe it is essential to include this negative control group (DC-GL261-FT) in our experiments, to benchmark it against DCs loaded with ferroptotic lysates. Thus, the DC-GL261-FT group was included in multiple experiments to enable this comparison.

In this way, the distinction between ferroptosis and accidental necrosis was demonstrated in the context of prophylactic vaccination **(Fig. 2)**, therapeutic vaccination **(Fig. 4B,C)** and the priming of cytotoxic T cell response **(Fig. 4E,F)**, as well as at the level of the immune cell infiltration **(Fig. 6, Suppl. Fig. 5)**.

In summary, by incorporating both immunogenic apoptotic lysates as a positive control (DC-GL261-MTX) and non-immunogenic necrotic lysates (DC-GL261-FT) as a negative control, we established a robust experimental framework to evaluate the specific immunogenic potential of ferroptotic lysates. Our data consistently demonstrate that ferroptosis-induced cell death elicits superior antitumor immune responses compared to accidental necrosis, as evidenced by enhanced survival, T cell priming, and immune infiltration. While ferroptotic and apoptotic lysates showed comparable immunogenicity in some settings, ferroptosis provides a distinct and relevant modality of ICD, especially considering its unique molecular pathways and translational relevance. We believe that our comprehensive experimental approach addresses the reviewer's concern and reinforces the validity of our conclusions.

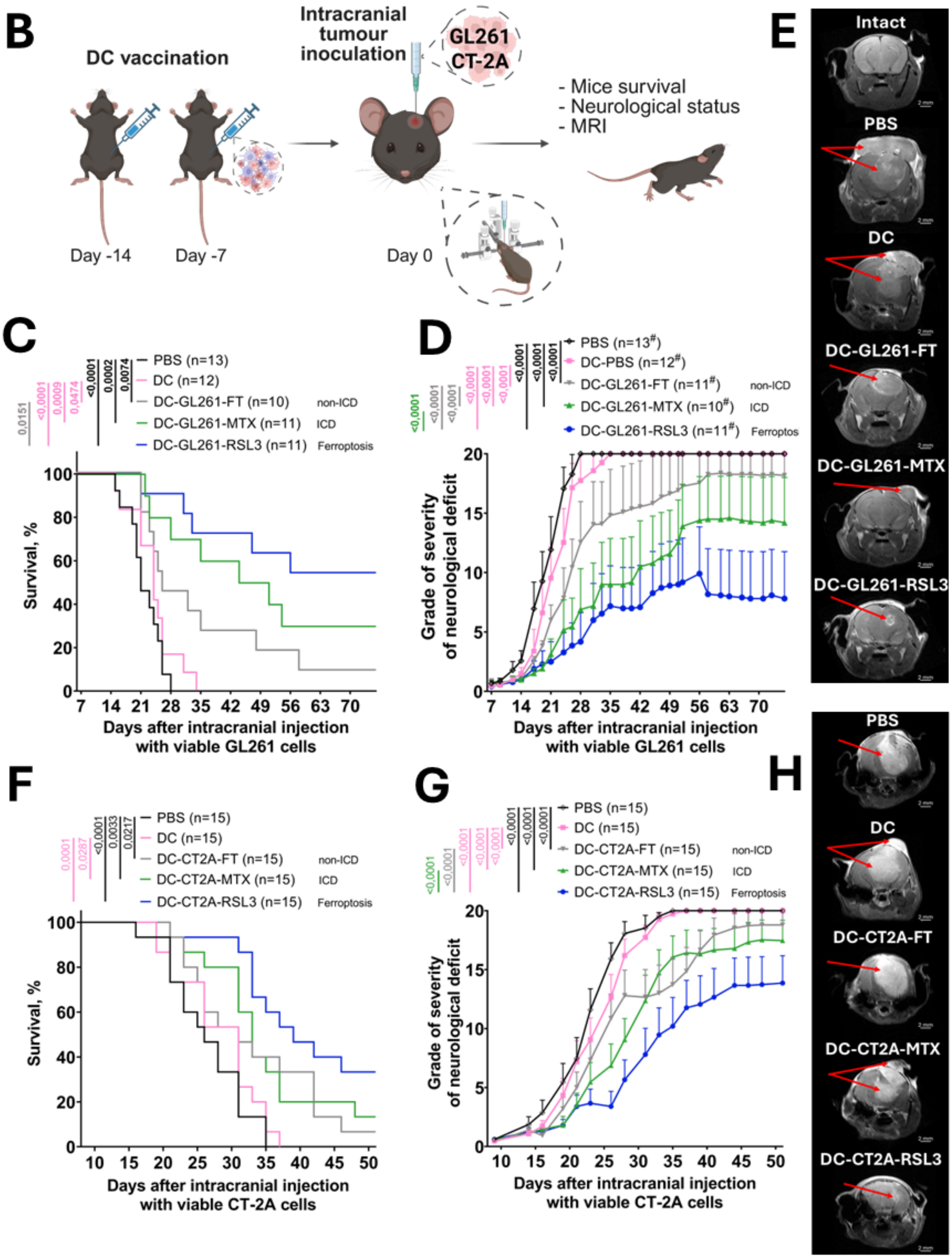

Figure 2. **(B)** Overview of the experimental setup to assess the effectiveness of DC vaccines in a prophylactic context using an orthotopic intracranial mouse model. Mice were administered $1*10^6$ DC vaccines' cells on days -14 and -7, followed by challenge with an intracranial injection of viable glioma GL261 ($2*10^4$ cells) or CT-2A ($5*10^5$ cells) cells on day 0. Survival outcomes, neurological status and MRI scans were monitored thereafter.

Prophylactic vaccination with DC-GL261-RSL3

Mouse survival **(C)**, neurological status **(D)** and MRI **(E)** were assessed after injection of the ferroptosis-armed DC vaccine, followed by intracranial challenge with viable glioma GL261 cells. Mouse survival and neurological status were assessed for up to 76 days. Control groups included mice injected with DC vaccines loaded with glioma GL261 lysate subjected to F/T cycles (negative control, non-ICD, DC-GL261-FT) or treated with MTX (2.5 μM, positive control-ICD, DC-GL261-MTX), and mice injected with unloaded DCs (negative control, DCs) or with PBS (negative control). #On day 23, three mice per group underwent MRI scans. In some of these mice (2 in the PBS group and 2 in the DC group), the glioma size had already reached humane endpoints, and they were euthanized following the MRI. These mice were included in the analysis of survival and neurological status. However, other mice (1 in the PBS and DC groups and 3 in each of the DC-GL261-FT, DC-GL261-MTX, and DC-GL261-RSL3 groups) did not reach humane endpoints and were euthanized after the MRI; these mice were excluded from the analysis. **(E)** Representative T1-weighted MRI images of coronal brain sections taken on day 23, n=3 mice per group. Arrows point to tumors. Statistical analysis

for mice survival was performed using Mantel-Cox logarithmic test. Statistical analysis of the neurological status of the mice was performed using a two-way ANOVA followed by Tukey's multiple comparisons correction.

Prophylactic vaccination with DC-CT2A-RSL3

Mouse survival **(F)**, neurological status **(G),** and MRI **(H)** were evaluated after injection of the ferroptosis-armed DC vaccines and intracranial challenge with viable glioma CT2A cells. Mouse survival and neurological status were monitored for up to 51 days. Control groups included mice injected with DC vaccines loaded with glioma CT-2A lysates subjected to F/T cycles (negative control, non-ICD, DC-CT2A-FT) or treated with MTX (2.5 μM, positive control-ICD, DC-CT2A-MTX), and mice injected with unloaded DCs (negative control, DCs) or PBS (negative control). **(H)** Representative T1-weighted MRI images of coronal brain sections taken on day 23, with the tumor indicated by arrows, n=4 mice per group. Statistical analysis for mice survival was determined by Mantel-Cox logarithmic test. Statistical analysis of the neurological status of the mice was performed using a two-way ANOVA followed by Tukey's multiple comparisons correction.

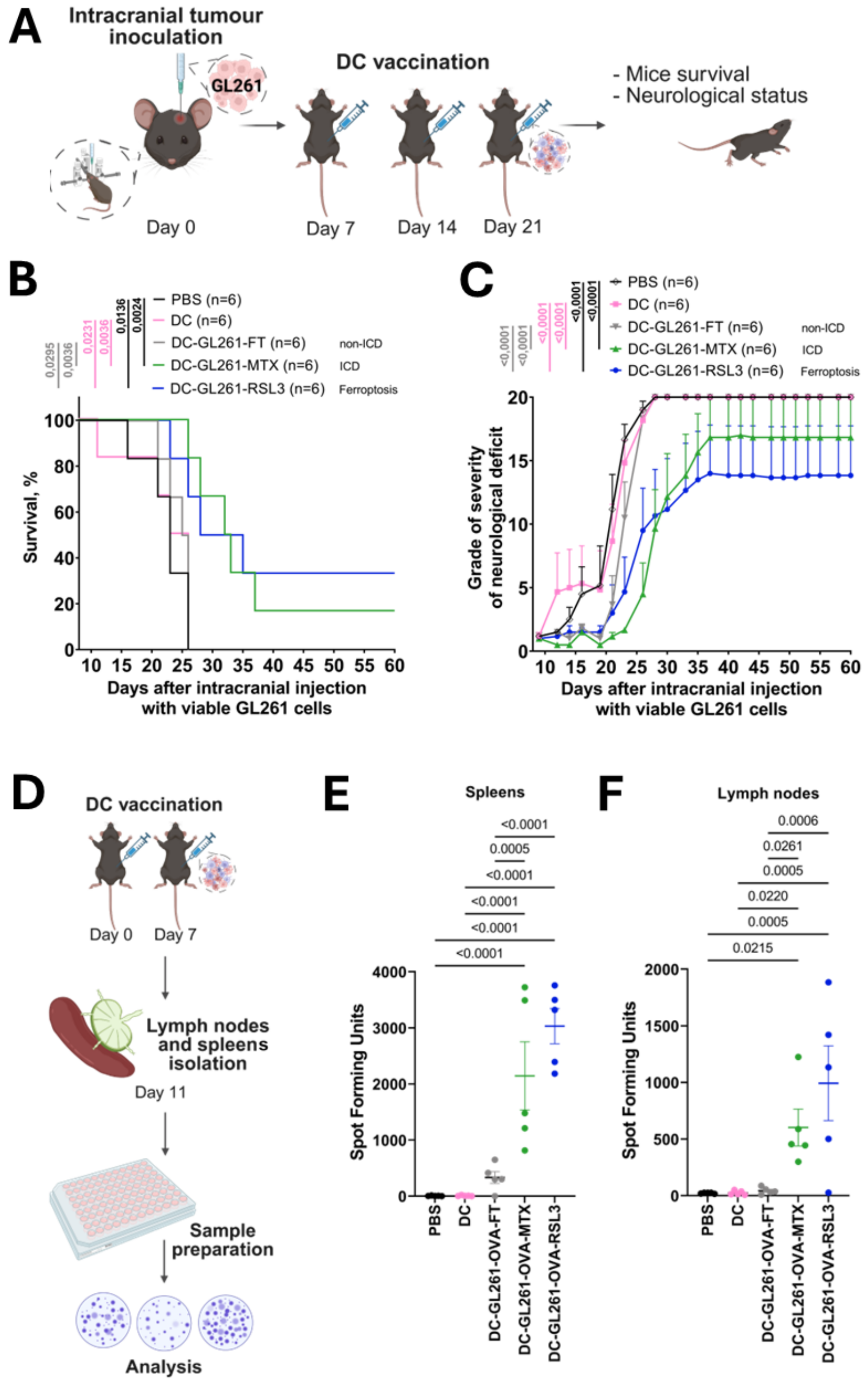

Figure 4. **(A)** Procedure to assess the efficacy of DC vaccines in a more clinically relevant therapeutic setting using an orthotopic intracranial mouse model. Mice were injected intracranially with viable glioma GL261 cells on day 0, followed by administration of the corresponding DC vaccine on days 7, 16 and 25. Control groups included mice injected with DC vaccines loaded with glioma GL261 lysate subjected to F/T cycles (negative control: non-ICD, DC-GL261-FT), DC vaccines loaded with MTX-treated

lysates (2.5 µM, positive control: ICD, DC-GL261-MTX), unloaded DCs (negative control, DCs) or PBS (negative control). Mouse survival **(B)** and neurological status **(C)** were assessed following intracranial injection with viable glioma GL261 cells and subsequent therapy with ferroptosis-armed DC vaccines. Survival and neurological status were monitored for up to 60 days. Mouse survival was analyzed using the Mantel–Cox log-rank test. Statistical analysis of the neurological status of the mice was performed using a two-way ANOVA followed by Tukey's multiple comparisons correction.

(D) Procedure to analyze the priming of cytotoxic T cells and IFN-γ production in mice immunized with DC vaccines on days 0 and 7. Four days after the final vaccination, the inguinal lymph nodes and splenocytes were isolated and re-stimulated *ex vivo* with the SIINFEKL peptide. **(E, F)** IFN-γ ELISpot assay of the spleens **(E)** and draining lymph nodes **(F)** from immunized mice. DC vaccines were loaded with ferroptotic GL261 lysates (DC-GL261-OVA-RSL3). Ferroptosis was induced by 2.5 µM RSL3. As controls, we used DC vaccines loaded with glioma GL261 cells subjected to F/T cycles (negative control: non-ICD, DC-GL261-OVA-FT), with MTX (2.5 µM, positive control: ICD, DC-GL261-OVA-MTX), unloaded DCs (negative control, DCs) or PBS (negative control). Each type of lysate was supplemented with 1 µM ovalbumin. The number of spot-forming units is presented as means ± SEM from data obtained from 5 mice. Statistical significance was determined using one-way ANOVA followed by Dunnett's multiple comparisons test.

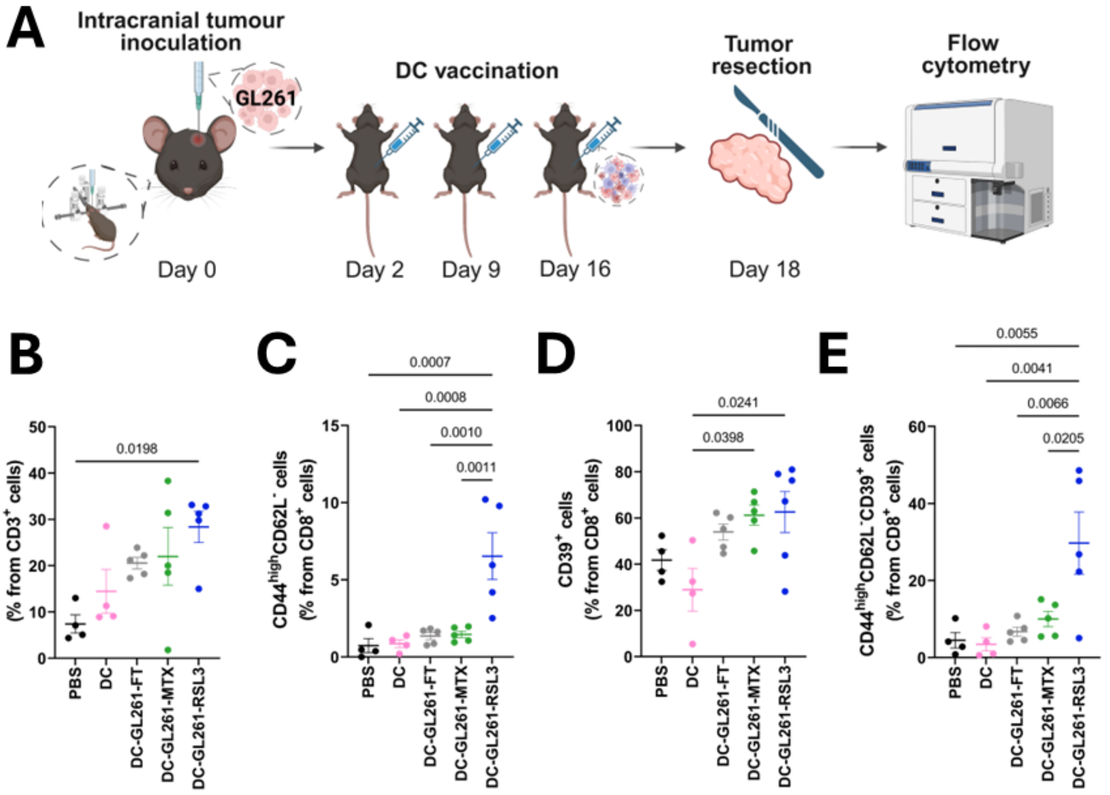

Figure 6. **(A)** Procedure to assess T-cell infiltration induced by DC vaccines in a therapeutic setting using an orthotopic intracranial mouse model. Mice were injected intracranially with viable glioma GL261 cells on day 0. Subsequently, they received the corresponding DC vaccine on days 2, 9 and 16. On day 18 after tumor inoculation, intracranial tumors were isolated, and immune cells were analyzed by multicolor flow cytometry. Control groups included mice treated with DC vaccines loaded with glioma GL261 lysate subjected to F/T cycles (negative control: non-ICD, DC-GL261-FT), DC vaccines loaded with MTX-treated lysates (2.5 µM, positive control: ICD, DC-GL261-MTX), unloaded DCs (negative control, DCs) or PBS (negative control). **(B-E)** Percentage of CD8$^+$ T cell infiltration. The ferroptosis-armed DC vaccine increased the infiltration of total CD8$^+$ T cells **(B)**, effector memory CD8$^+$ T cells (CD44$^+$CD62L$^-$ **C)**, CD39$^+$ exhausted CD8$^+$ T cells **(D)** and CD39$^+$ effector memory CD8$^+$ T cells **(E)**. Statistical significance was determined using one-way ANOVA followed by Dunnett multiple comparisons test.

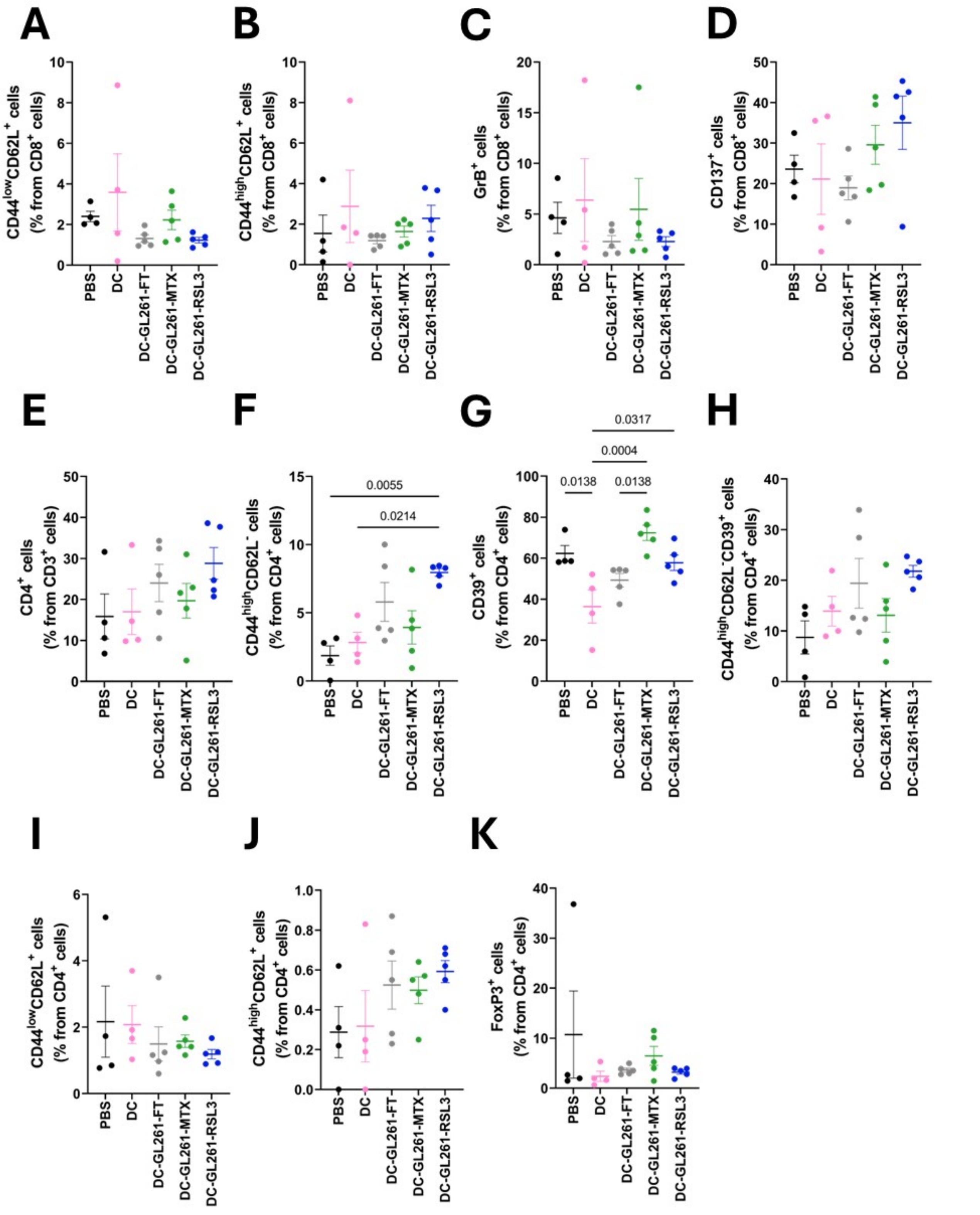

Suppl. Figure 5. **(A-D)** Percentage of $CD8^+$ T cell infiltration. The ferroptosis-armed DC vaccine did not alter the infiltration of naïve ($CD44^{low}CD62L^+$) **(A)**, central memory ($CD44^{high}CD62L^+$) **(B)**, activated (GrB^+) **(C)**, or tumor-specific ($CD137^+$) **(D)** $CD8^+$ T cells.

(E-H) Percentage of $CD4^+$ T cell infiltration **(E)**, effector memory $CD4^+$ T cells ($CD44^{low}CD62L^-$) **(F)**, $CD39^+$ $CD4^+$ T cells **(G)**, and $CD39^+$ effector memory $CD4^+$ T cells **(H)**.

(I–K) The ferroptosis-armed DC vaccine did not alter the infiltration of naïve ($CD44^{low}CD62L^+$) **(I)**, central memory ($CD44^{high}CD62L^+$) **(J)**, or regulatory ($FoxP3^+$) **(K)** $CD4^+$ T cells. Statistical significance was determined using one-way ANOVA followed by Dunnett's multiple comparisons test.

Comment 2. *"Comparison with necrotic cell death lysate was not entirely accurate since this type of cell death does not lead to activation of DCs. In the multiple previous studies, potent antitumor effect of tumor cell lysates loaded DCs was achieved when DCs were activated with various TLR agonists or CD40 that would lead to up-regulation of MHC class I and co-stimulatory molecules. This was not done in this study. In the absence of this treatment, DCs were simply not activated and comparison with ferroptosis induced lysates was not convincing since ferroptosis as authors showed caused modest up-regulation of DCs. To follow on the previous point, RSL3 induced less activation of DCs than LPS. Why don't authors try to compare the effect of different vaccines (RSL3, MTX, necrosis) in LPS activated DCs. Maybe it would allow them to observe more differences in antitumor activity between apoptosis and ferroptosis."*

Answer 2. Indeed, analyzing the effect of LPS, a TLR4 agonist, is an important aspect of DC vaccine characterization and may also enhance their efficacy. In our study, during the co-cultivation experiments of DC with lysates, a specific type of lysates was added first for 90 minutes, followed by the additional (or not) of LPS. This approach allowed us to assess the effect of LPS on DC maturation and to compare both treated and untreated cells **(Fig. 5B-D)**.

To clarify the experiment setup, we have added an additional figure **(Fig. 5A)**.

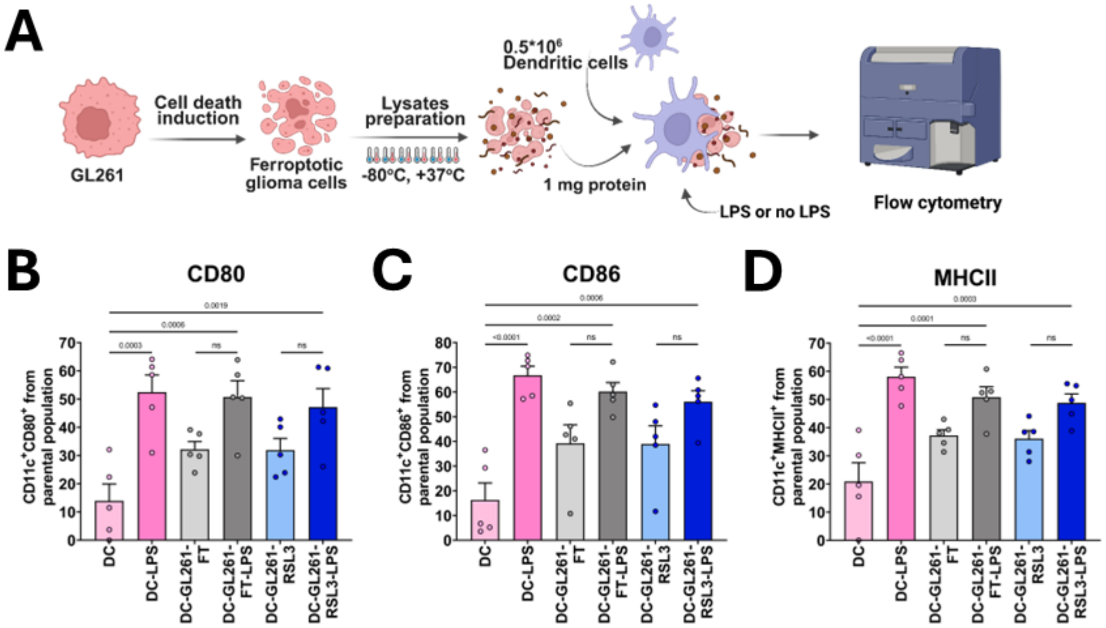

Figure 5. **(A)** Procedure for studying DCs maturation after co-culture with respective lysates. DCs were co-cultured with ferroptotic GL261 lysates (2.5 μM, 24h, DC-GL261-RSL3) or F/T GL261 lysates (non-ICD, negative control, DC-GL261-FT) for 90 minutes, and then treated with lipopolysaccharide (LPS, 0.5 μg/ml) or left untreated for 24 hours. Unloaded DCs stimulated with LPS served as a positive control (DC-LPS), while untreated (DCs) served as a negative control.

(B, C, D) The percentages of viable $CD11c^{+}CD80^{+}$ **(B)**, $CD11c^{+}CD86^{+}$ **(C)**, and $CD11c^{+}MHCII^{+}$ **(D)** DCs are shown as mean values ± SEM from 5 independent experiments. Two-way ANOVA was used to assess the statistical significance of the results, followed by Hom-Sidac multiple comparisons test.

In clinical practice and in *in vivo* experiments, immature DCs are typically pulsed with tumor lysates, followed by a maturation step (Palucka & Banchereau, 2013). In the context of *in vivo* studies, LPS is frequently used as a maturation or activating signal in various cancer models, including the orthotopic intracranial glioma model (Basirjafar et al., 2024; Vandenberk et al., 2016).

To analyze the effect of LPS not only on DC maturation but also on the therapeutic efficacy of DC vaccine loaded with ferroptotic lysates, a series of experiments were conducted in which mice were treated with DC vaccines either supplemented with LPS or not. The DC vaccines were generated using TLR4 knockout (TLR4KO) DCs **(Fig. 5 E-F')**. TLR4 is the primary receptor for LPS;

thus, using TLR4KO DCs allowed us to specifically assess the role of LPS in modulating the therapeutic efficacy of ferroptotic-lysates-loaded DC vaccines.

Our results showed that the addition of LPS did not significantly influence the efficacy of the DC vaccine. Importantly, the ferroptotic-armed TLR4KO DC vaccine significantly prolonged mouse survival compared to unloaded DCs in both conditions -with and without LPS **(Fig. 5G, H)**.

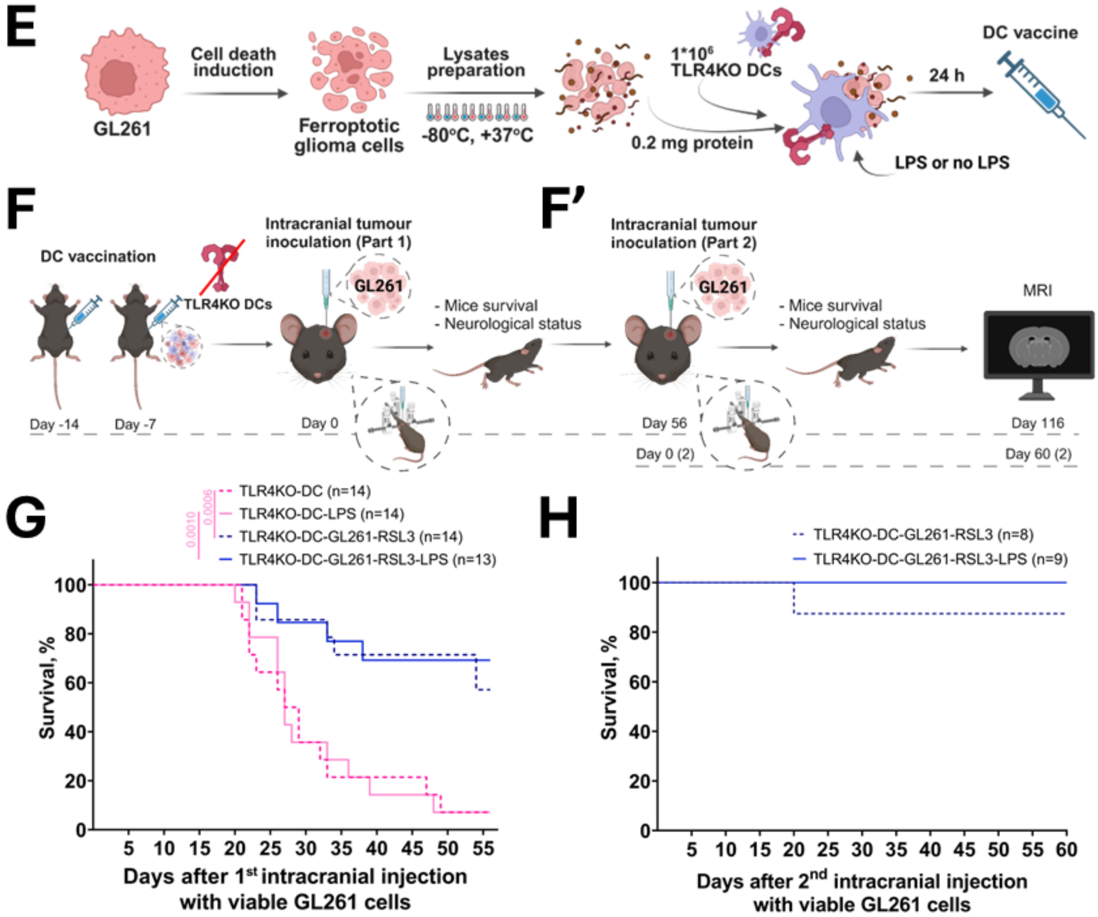

Figure 5. **(E)** Procedure for generating ferroptosis-armed DC vaccines from mouse bone marrow DCs of TLR4 knock out mice (TLR4KO-DCs). TLR4KO-DCs were co-cultured for 90 minutes with ferroptotic GL261 lysates (RSL3, 2.5 µM, 24 h) and then co-cultured with ferroptotic lysates in the presence or absence of 0.5 µg/ml LPS for 24 hours.

Part 1:

(F) Procedure for studying the effectiveness of TLR4KO-DC vaccines generated in the presence or absence of LPS (as explained in **E**) in wild type C57BL/6J mice (*i.e.*, **part 1**).

Wild type mice received ferroptosis-armed TLR4KO-DC vaccines (TLR4KO-DC-GL261-RSL3 or TLR4KO-DC-GL261-RSL3-LPS) on days -14 and -7, followed by intracranial injection of viable glioma GL261 cells on day 0 **(part 1)**. In part 1, mouse survival and neurological status were monitored up to day 56.

In part 1 **(F)**, mouse survival after injection of ferroptosis-armed TLR4KO DC vaccines (2.5 µM, 24 h, TLR4KO-DC-GL261-RSL3) followed by intracranial challenged with viable GL261 cells. Mouse survival was monitored for up to 56 days **(part 1)**. As controls, we used unloaded TLR4KO DCs, which were either treated with LPS (TLR4KO-DC-LPS) or untreated (TLR4KO-DC). Statistical analysis was performed using the Mantel-Cox logarithmic test.

Part 2:

(F') Procedure **(part 2)** for assessing the development of immunological memory in immunocompetent C57BL/6J mice that had rejected viable GL261 cells due to therapy with ferroptosis-armed TLR4KO-DCs in **(F, part 1)**. **In part 2**, the surviving mice were re-challenged on day 0 of the second intracranial glioma injection (56 days after the first challenge) with a GL261 glioma cell dose that normally induces glioma growth in naive mice (*e.g.*, see **Fig. 2C, F**; the negative control group injected with PBS). Mouse survival and neurological status were monitored for up to 116 days after the first injection (*i.e.*, 60 after the second injection). The MRI scans were performed on day 116.

(G: part 1; H: part 2) Mouse survival after therapy with ferroptosis-armed TLR4KO-DC vaccines (TLR4KO-DC-GL261-RSL3) and subsequent intracranial rechallenge with viable GL261 cells. Mouse survival was monitored for up to 60 days after the rechallenge. As controls, we used unloaded TLR4KO-DC treated with LPS (TLR4KO-DC-LPS) or left untreated (TLR4KO-DC). Results were analyzed with Mantel-Cox logarithmic rank test.

In response to Reviewer #1's comment, we conducted an additional experiment in which DC vaccines were generated from wild-type mice, and the effect of LPS treatment versus no LPS treatment was analyzed. The ferroptotic-armed DC vaccine significantly improved mouse survival compared to unloaded DCs, regardless of the presence or absence of LPS **(Fig. 5E-G)**.

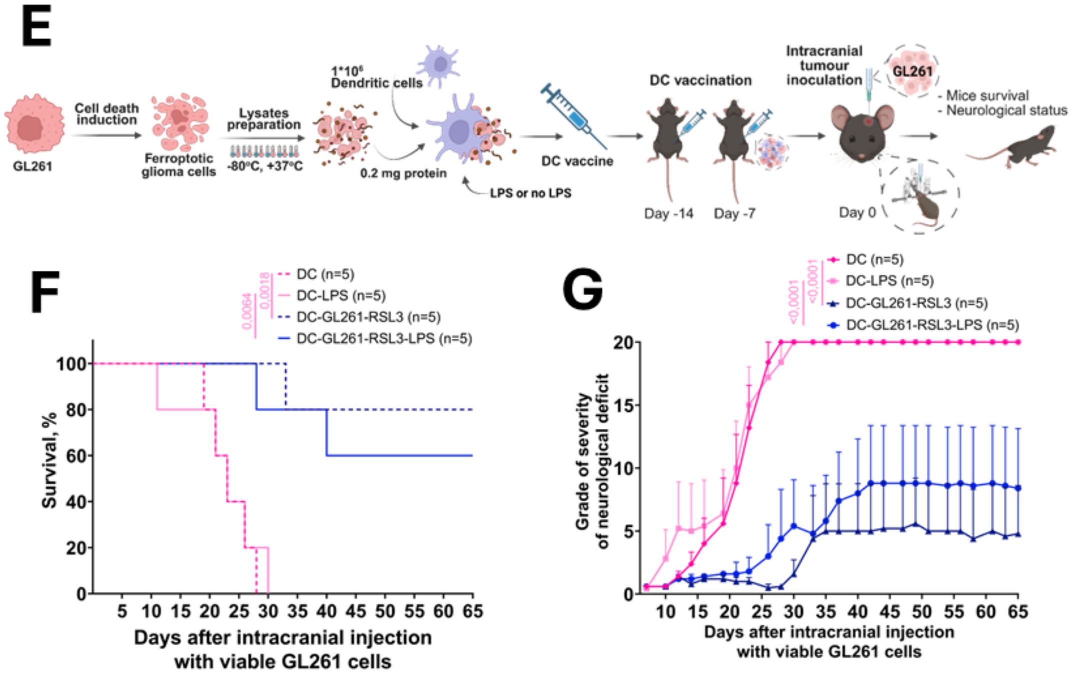

Suppl. Figure 3. (E) Procedure for generating ferroptosis-armed DC vaccines from wild-type (WT) mouse bone marrow-derived DCs (WT-DCs). WT-DCs were co-cultured for 90 minutes with ferroptotic GL261 lysates (RSL3, 2.5 μM, 24 h), and then further incubated with the same lysates in the presence (DC-GL261-RSL3-LPS) or absence (DC-GL261-RSL3) of 0.5 μg/mL LPS for 24 hours. WT mice received the respective DC vaccines on days –14 and –7, followed by intracranial injection of viable GL261 glioma cells on day 0. As controls, mice were treated with unloaded DCs, either LPS-stimulated (DC-LPS) or unstimulated (DC).
(F, G) Mouse survival **(F)** and neurological status **(G)** were monitored up to day 65. Survival data were analyzed using the Mantel-Cox log-rank test. Neurological status was analyzed using two-way ANOVA followed by Tukey's multiple comparisons test.

Comment 3. *"Analysis of T cell response was very limited. No comparison with MTX induced cell death was performed and no difference between FT and RSL3 induced cell death was seen."*

Answer 3. Following the Reviewer #1 comment, we have performed new experiments (**Fig. 4D-F** and please see above the Answer 1).

The experimental design included the use of DC vaccines loaded with glioma GL261 glioma cells subjected to F/T cycles (negative control: non-ICD, DC-GL261-OVA-FT), treated with MTX (2.5 μM, positive control: ICD, DC-GL261-OVA-MTX), as well as unloaded DCs and PBS (both negative controls). A significant increase in T cell responses was observed in the DC-GL261-OVA-RSL3 (ferroptosis) and DC-GL261-OVA-MTX (ICD, positive control) groups, but not in the negative control groups DC-GL261-OVA-FT, DC and PBS. These new data have been added in the revised version of the manuscript.

Comment 4. *"The effect of vaccination in a weakly immunogenic CT-2A tumor was very small raising the question of biological impact of this source of antigens. The same concern is with Figure 3. Effects are too small for strong conclusions made by the authors."*

Answer 4. We appreciate the reviewer's comment and the opportunity to clarify. We have performed additional experiments in which we increased the number of mice in order to draw valid conclusions. We performed prophylactic vaccination lysates derived from with GL261 and CT-2A cells (**Fig. 2C–H** and please see above the Answer 1).

We have also conducted a new experiment involving therapeutic vaccination in the GL261 glioma model. In this experiment, vaccination was started 7 days after the intracranial inoculation of tumour cells to achieve a more clinically relevant setting (**Fig. 4B, C** and please see above the Answer 1).

While the CT-2A model is indeed considered weakly immunogenic, our data show that ferroptosis-armed DC vaccination conferred a clear survival benefit even in this challenging context. In the prophylactic setting, 33% (5/15) of CT-2A-bearing mice survived following vaccination, compared to 0% in the PBS and unloaded DC control groups (**Fig. 2F-H** and please see above the Answer 1). For comparison, in the more immunogenic GL261 model, survival reached 54% (6/11), reinforcing the reproducibility and biological relevance of the approach across tumor types (**Fig. 2C-E** and please see above the Answer 1).

In the therapeutic setting, as expected, overall efficacy was reduced compared to prophylactic vaccination, yet the ferroptotic DC vaccine still improved survival relative to controls—8% in GL261 and 25% in CT-2A (**Fig. 3**). These findings demonstrate that ferroptotic lysates can promote antigen presentation and T cell priming even in low-immunogenic tumor models. While the magnitude of the effect is modest, it is both statistically significant and biologically meaningful, especially considering the inherent resistance of the CT-2A model to immunotherapy.

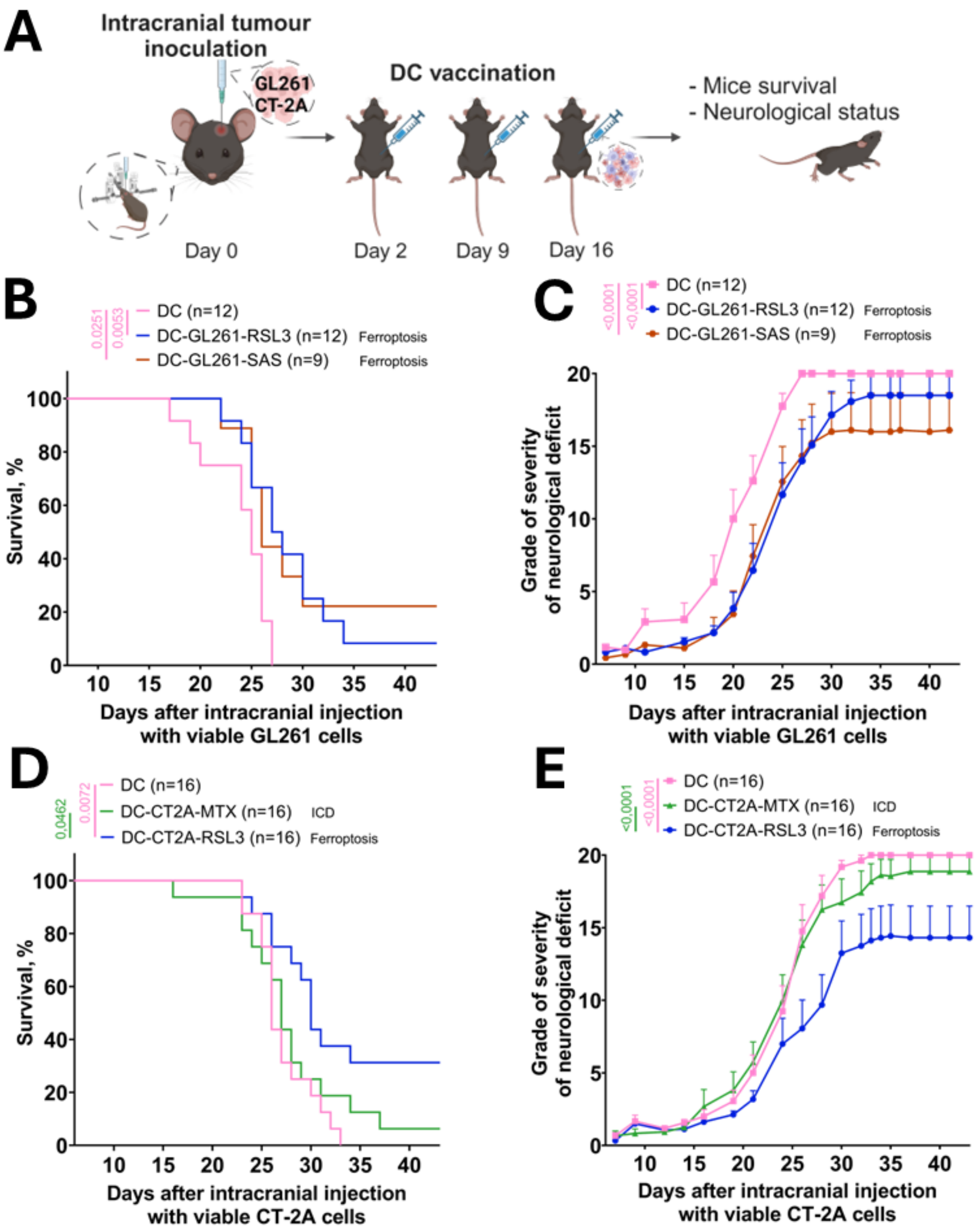

Figure 3. **(A)** Procedure to assess the effectiveness of DC vaccines in a therapeutic setting using an orthotopic intracranial mouse model. On day 0, mice were injected intracranially with viable glioma GL261 or CT-2A cells, followed by administration of the corresponding DC vaccine on days 2, 9 and 16. Ferroptosis-armed DC vaccines were prepared using ferroptosis inducers with different mechanisms of action: RSL3 (2.5 μM, 24 h, DC-GL261-RSL3) or SAS (300 μM, 24 h, DC-GL261-SAS). Unloaded DCs were used as a control (negative control, DCs).

Therapeutic vaccination with DC-GL261-RSL3

Mouse survival **(B)** and neurological status **(C)** after intracranial injection with viable glioma GL261 cells followed by therapy with ferroptosis-armed DC vaccines. Mouse survival and neurological status were monitored for up to 42 days. Statistical analysis for mice survival was determined by Mantel-Cox logarithmic test. Statistical analysis of the neurological status of the mice was performed using a two-way ANOVA followed by Tukey's multiple comparisons correction.

Therapeutic vaccination with DC-CT2A-RSL3

Mouse survival **(D)** and neurological status **(E)** after intracranial injection with viable CT-2A cells followed by therapy with ferroptosis-armed DC vaccines. Mouse survival and neurological status were monitored for up to 55 days. Ferroptosis-armed DC vaccines were prepared with ferroptosis inducer RSL3 (2.5 μM, 24 h, DC-CT2A-RSL3). As controls, we used mice injected with DC vaccines loaded with glioma GL261 lysates treated with MTX (2.5 μM, positive control-ICD, DC-CT2A-MTX), or mice injected with unloaded DCs (negative control, DCs). Statistical analysis for mice survival was determined by Mantel-Cox logarithmic test. Statistical analysis of the neurological status of the mice was performed using a two-way ANOVA followed by Tukey's multiple comparisons correction.

Comment 5. *"One of the most exciting questions is the reason for potential specificity of RSL3 induced cell death associated proteins. If authors could assess proteins in some fashion (for instance proteomics) this would enhance the impact of the study."*

Answer 5. We fully agree with the reviewer that identifying proteins specifically associated with RSL3-induced cell death would significantly enhance the mechanistic insight and impact of the study. To address this, we re-analyzed the proteomic data of the lysates (**Fig. 7, Suppl. Fig. 7**) and integrated this with additional analyses focusing on the prediction of immunogenic MHC-I and MHC-II peptides (**Fig. 7E and Suppl. Table 3,4**). These new data are now included in the revised manuscript, along with corresponding figures. For further details, please refer to our response to Reviewer 2 and the newly added results and figures in the revised version.

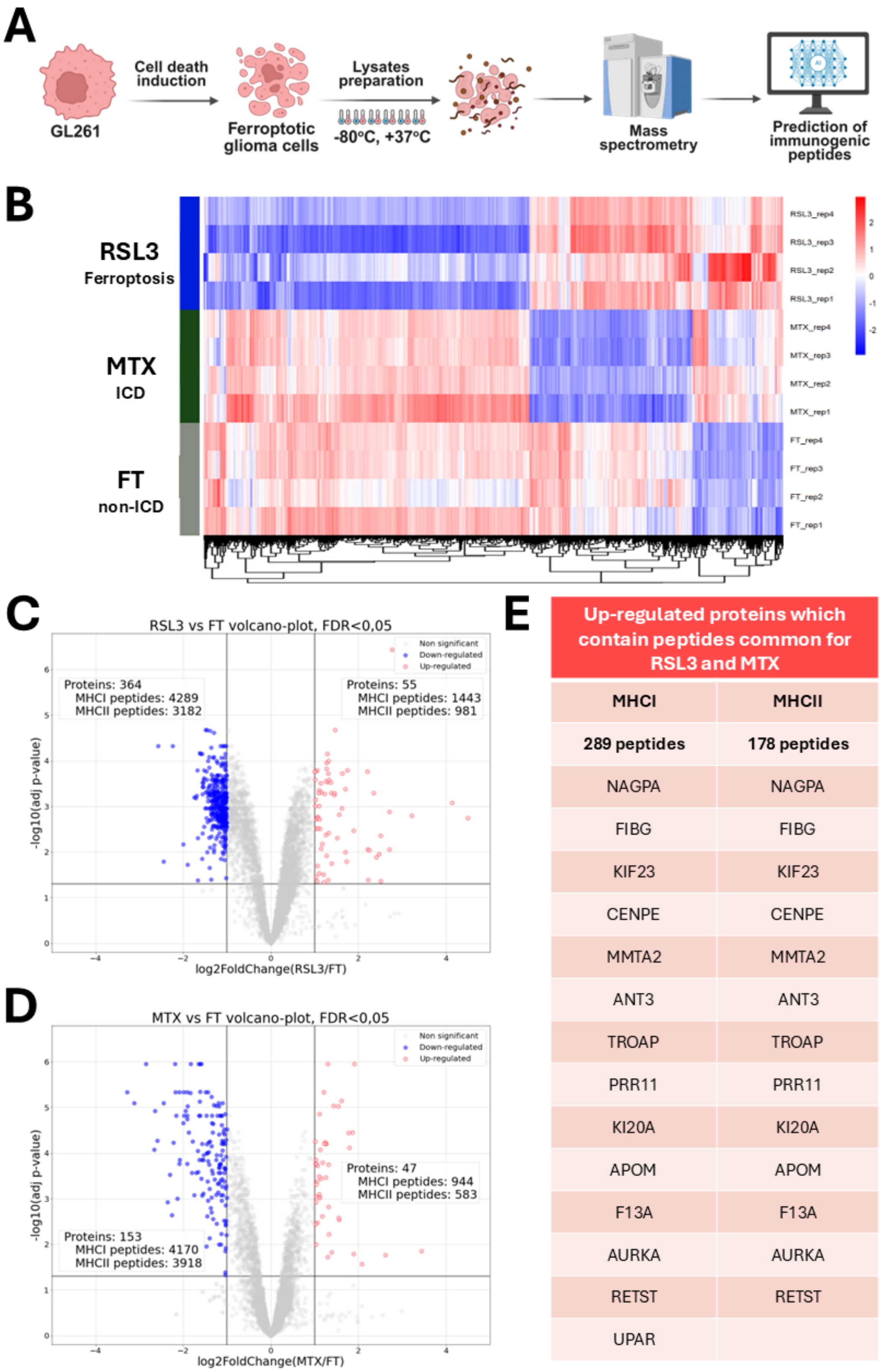

E

Up-regulated proteins which contain peptides common for RSL3 and MTX	
**MHCI**	**MHCII**
**289 peptides**	**178 peptides**
NAGPA	NAGPA
FIBG	FIBG
KIF23	KIF23
CENPE	CENPE
MMTA2	MMTA2
ANT3	ANT3
TROAP	TROAP
PRR11	PRR11
KI20A	KI20A
APOM	APOM
F13A	F13A
AURKA	AURKA
RETST	RETST
UPAR	

Figure 7. **(A)** Ferroptosis and immunogenic apoptosis were induced in GL261 cells using RSL3 (2.5 μM, 24 h) and MTX (2.5 μM, 24 h; ICD: positive control), respectively. As a negative control, GL261 cells were subjected to F/T (non-ICD). Proteomic analysis was then performed on the lysates used to load the DC vaccines. **(B)** Proteins differentially abundant between groups were visualized in a heatmap following un-supervised hierarchical clustering of z-scored $\log_2$ reporter intensity values. **(C, D)** Differential protein expression analysis comparing RSL3 vs. F/T **(C)** and MTX *versus* F/T **(D)**. **(E)** Commonly upregulated proteins which contain peptides shared between RSL3- and MTX-treated lysates.

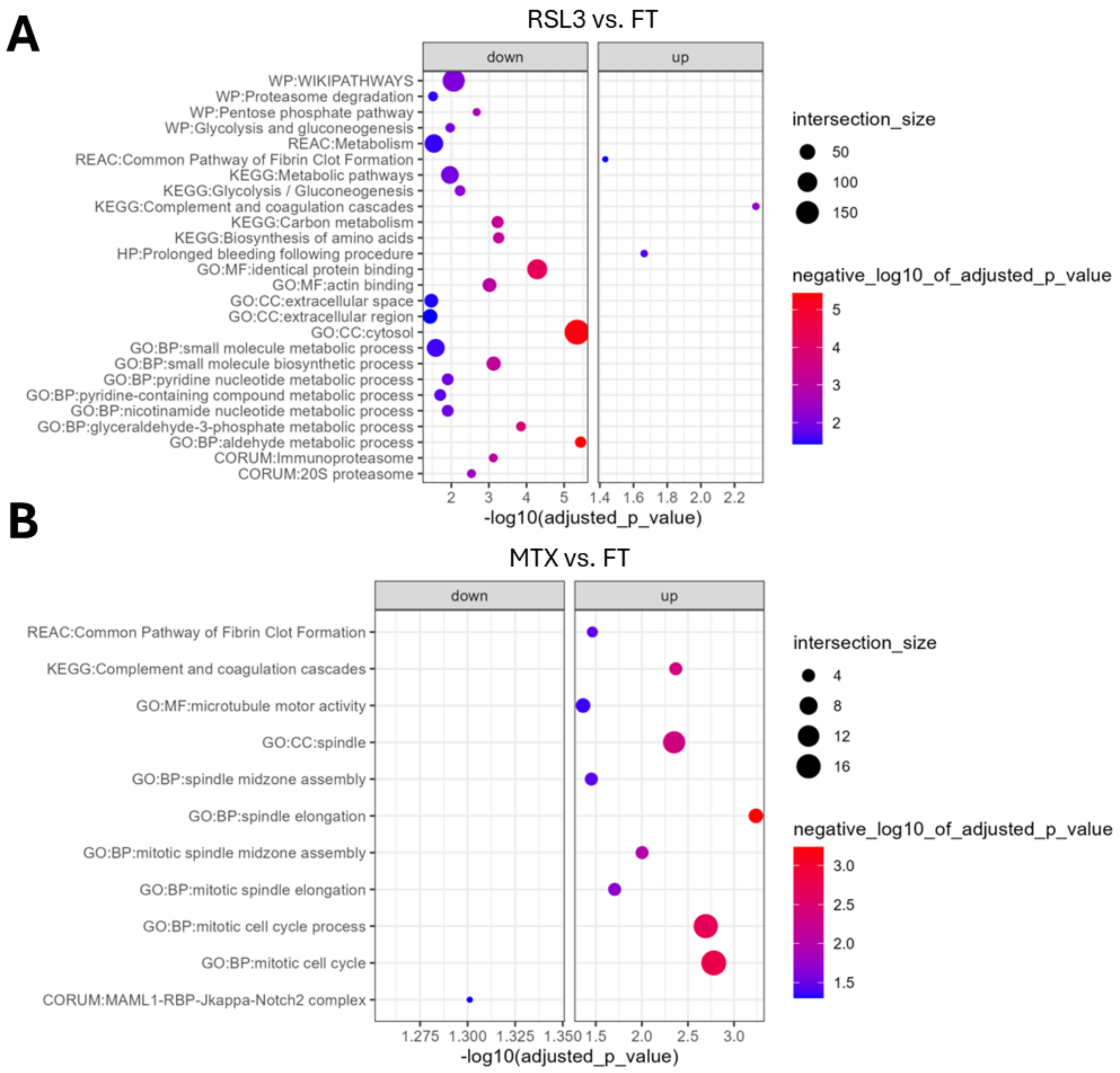

Suppl. Figure 7. **(A, B)** Gene Ontology (GO) enrichment analysis of differentially expressed genes (DEGs) in RSL3 *versus* F/T lysates (**A**) and MTX *versus* F/T lysates (**B**). Circle color represents the significance level of enrichment, and circle size indicates the number of enriched genes. Enrichment results with $p < 0.05$ were considered statistically significant.

Reviewer #2

"Saviuk et al. described the study of DC vaccine development via activation with ferroptotic glioma cells. I focused my review on the proteomic aspect of the research. Proteomic analyses compared glioma cells treated with RSL3 (ferroptosis inducer) or MTX and compared to control cells. The authors show the results of this comparison in Figure 4H. Overall, the description of the results and the methodology are too minimalistic to understand the contribution of these data to the manuscript. I list below the specific comments."

We sincerely thank the Reviewer #2 for the insightful comments and for taking the time to evaluate our manuscript. We greatly appreciate the reviewer's focus on the proteomic aspect of our study and the constructive feedback regarding **Fig. 4H**. We agree that the description of the results and methodology was too minimal, and we have revised the corresponding sections to provide more detailed information and to clarify the contribution of these data to the manuscript. Please find our detailed responses to the specific comments below.

Comment 1. *"The manuscript lacks a description of the proteins that significantly change between treatment groups. The heatmap (Fig. 4H) hardly gives any information, as it is impossible to see the protein names. The authors should expand the description to show the changing proteins and biological processes. In addition, they should add the list of significantly changing proteins as supplementary material."*

Answer 1. We performed a more comprehensive analysis of the proteomics data. Specifically, we analyzed the up- and downregulation of proteins in ferroptotic (RSL3), apoptotic (MTX) and accidental necrotic (FT) lysates. We then performed an enrichment analysis to determine the functional activity of these peptides. The list of significantly altered proteins has been included as **Suppl Table 2**.

In addition, we analyzed the peptides derived from these proteins to predict their potential immunogenicity. The *Results* and *Materials and Methods* sections of the manuscript have been updated accordingly. Furthermore, we have included additional main and supplementary figures: **Fig. 7**, **Suppl. Fig. 7**.

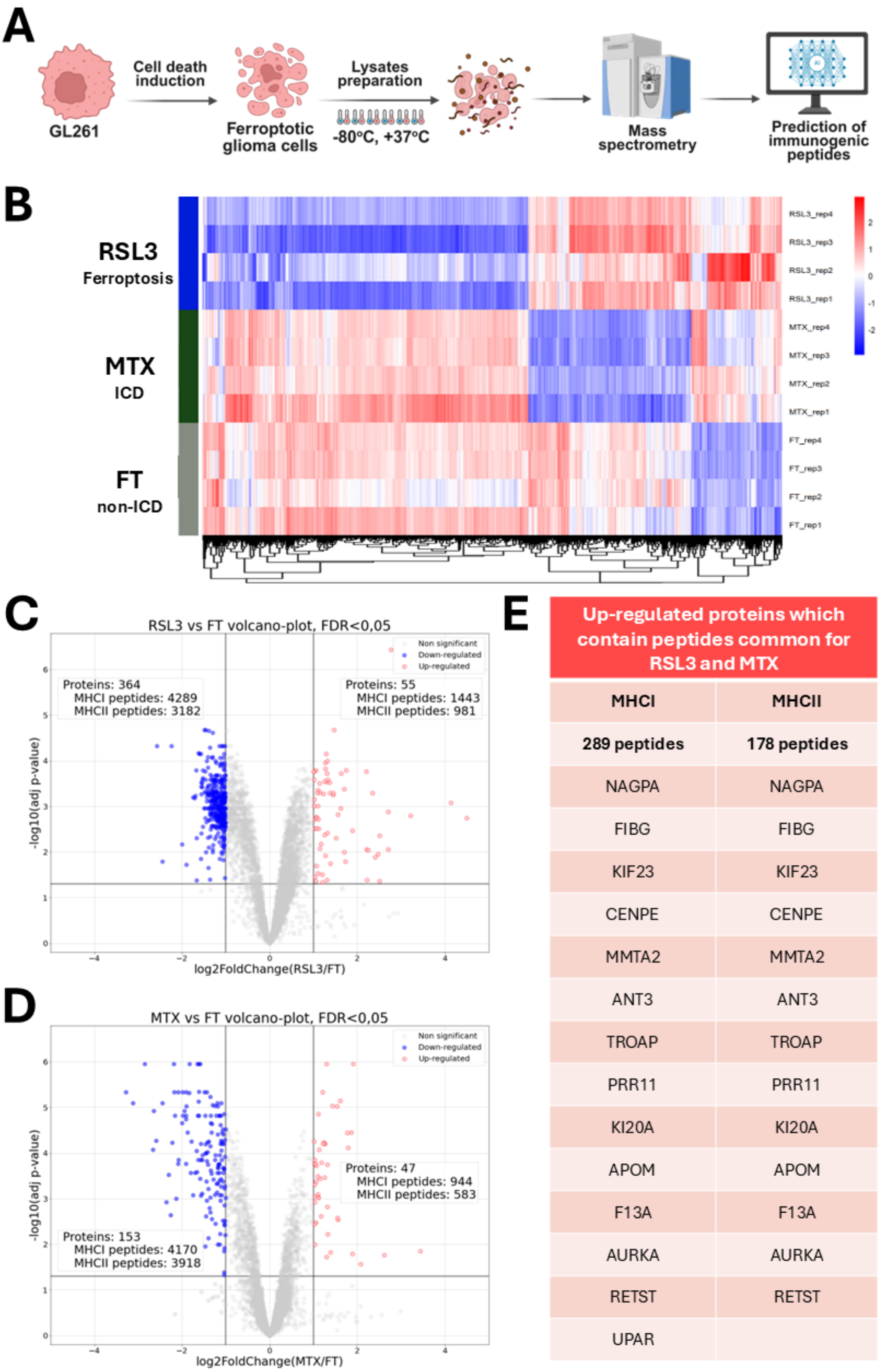

Up-regulated proteins which contain peptides common for RSL3 and MTX	
**MHCI**	**MHCII**
**289 peptides**	**178 peptides**
NAGPA	NAGPA
FIBG	FIBG
KIF23	KIF23
CENPE	CENPE
MMTA2	MMTA2
ANT3	ANT3
TROAP	TROAP
PRR11	PRR11
KI20A	KI20A
APOM	APOM
F13A	F13A
AURKA	AURKA
RETST	RETST
UPAR	

Figure 7. **(A)** Ferroptosis and immunogenic apoptosis were induced in GL261 cells using RSL3 (2.5 µM, 24 h) and MTX (2.5 µM, 24 h; ICD: positive control), respectively. As a negative control, GL261 cells were subjected to F/T (non-ICD). Proteomic analysis was then performed on the lysates used to load the DC vaccines. **(B)** Proteins differentially abundant between groups were visualized in a heatmap following un-supervised hierarchical clustering of z-scored log_2 reporter intensity values. **(C, D)** Differential protein expression analysis comparing RSL3 vs. F/T **(C)** and MTX *versus* F/T **(D)**. **(E)** Commonly upregulated proteins which contain peptides shared between RSL3- and MTX-treated lysates.

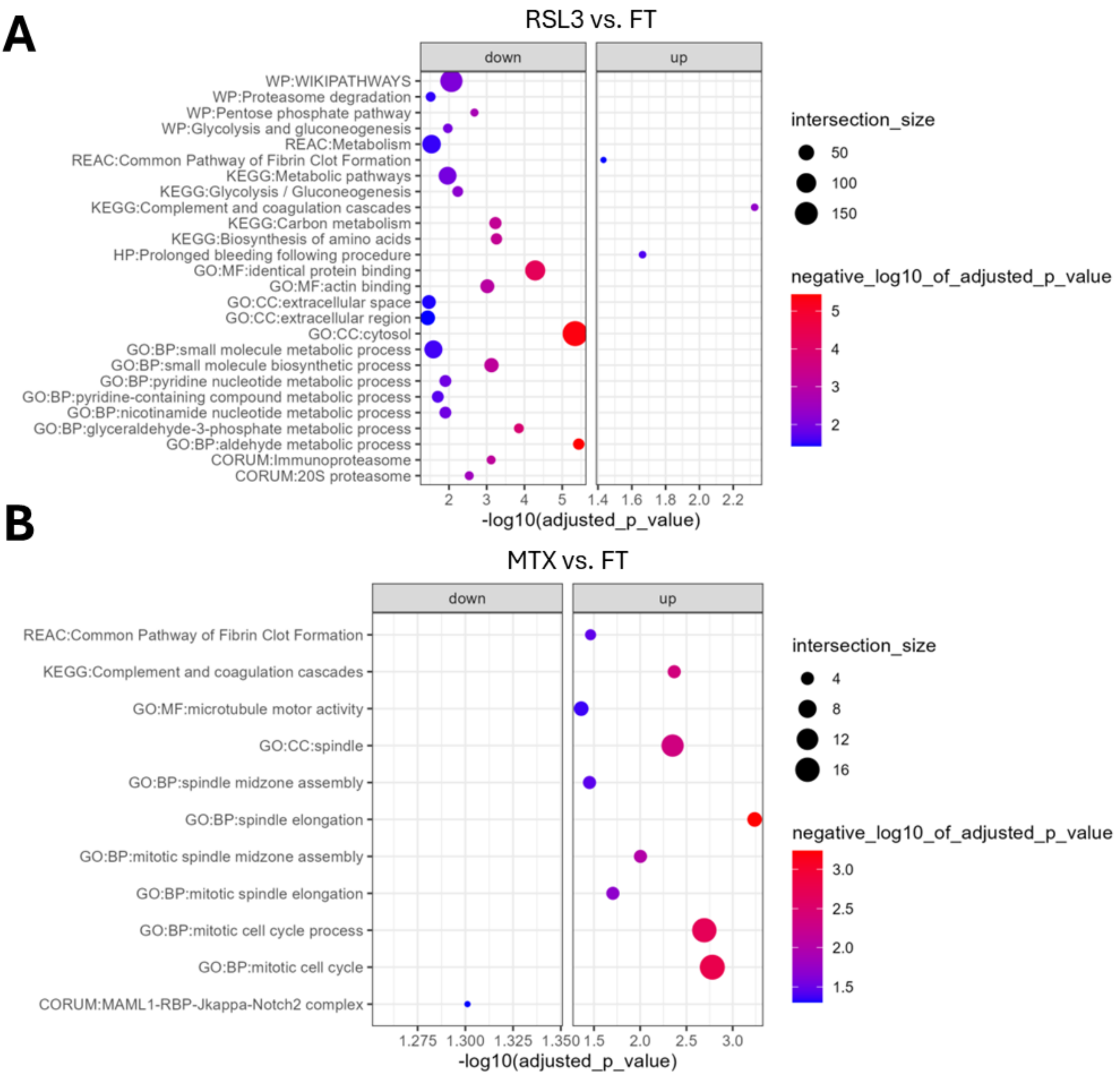

Suppl. Figure 7. **(A, B)** Gene Ontology (GO) enrichment analysis of differentially expressed genes (DEGs) in RSL3 *versus* F/T lysates (**A**) and MTX *versus* F/T lysates (**B**). Circle color represents the significance level of enrichment, and circle size indicates the number of enriched genes. Enrichment results with $p < 0.05$ were considered statistically significant.

Results section Page 8:

"To assess differences in proteins intensities between ferroptotic lysates (RSL3) and FT lysates (negative control: non-ICD) (**Fig. 7C**), as well as between MTX-treated lysates (positive control: ICD, MTX) and FT lysates (**Fig. 7D**), statistical comparisons of group means were performed. We identified 55 up-regulated and 364 down-regulated proteins in ferroptotic lysates compared to the FT group (non-ICD), and 47 upregulated and 153 downregulated proteins in MTX-treated lysates (ICD) compared to FT lysates. Functional enrichment analysis revealed a downregulation of proteins involved in metabolic processes in ferroptotic lysates (**Suppl. Fig. 7A**) and an upregulation of proteins associated with mitotic processes in MTX-treated lysates (**Suppl. Fig. 7B**).

Next, we analyzed the peptides which can potentially be presented in the respective lysates derived from these proteins and predicted their immunogenicity in the context of murine MHC class I and II complexes. We identified 1,443 and 944 potentially MHC-I-presented immunogenic

peptides, as well as 981 and 583 potentially MHCII-presented immunogenic peptides, in the ferroptotic and MTX-treated lysates, respectively. Notably, the ferroptotic and MTX-treated lysates shared 14 common upregulated proteins, giving rise to 289 potentially MHCI-presented immunogenic peptides. Additionally, 13 commonly upregulated proteins between the two lysates contained 178 potentially MHCII-presented immunogenic peptides (**Fig. 7E, Suppl. Tables 3, 4**). This distinct profile in ferroptotic lysates likely introduces novel antigenic determinants (Catanzaro et al., 2024), fostering tumor-targeting immune responses and contributing to the observed immunogenic properties of ferroptotic lysates in the murine orthotopic glioma model."

Comment 2. *"Following the previous comment, the authors should discuss these results also in the Discussion section."*

Answer 2. We have added the following paragraph to the Discussion section on page 12.

"Our comparative proteomic and in silico immunopeptide prediction revealed unique antigenic determinants of ferroptosis compared to both immunogenic apoptotic (MTX-treated) and non-immunogenic necrotic (FT) lysates (**Fig. 7B and Fig. 7E**). Interestingly, we identified a subset of proteins that were commonly upregulated in both ferroptotic and immunogenic apoptotic lysates, giving rise to MHC class I- and II- potentially presentable shared peptides. This overlap suggests the presence of a conserved ICD signature, potentially reflecting a shared mechanism of adaptive immune activation across different ICD modalities. At the same time, the broader divergence in antigenic profiles supports the notion that ferroptosis introduces a unique repertoire of tumor-associated antigens (Catanzaro et al., 2024). These findings imply that ferroptosis may not only partially mimic classical ICD pathways but also contribute novel neoantigens, which could be used for the development of ferroptosis-based cancer vaccines or combination immunotherapies. Such neoantigens may serve as promising candidates for designing broad-spectrum therapeutic cancer vaccines or for identifying biomarkers of effective ICD-based immunotherapy."

Comment 3.*"Supplementary material should include an Excel file with all identified proteins (MaxQuant output table)."*

Answer 3. An Excel file containing all identified proteins and associated information **(Suppl. Table 1)**, as well as differential expression analysis results **(Suppl. Table 2)**, has been included in the supplementary materials.

Comment 4. *"The methods section should provide more details about the sample preparation, data acquisition, and data analysis. Missing information includes TMT, peptide pre-fractionation, length of runs, MS acquisition parameters, and the details of the maxquant parameters. Default settings might change from one version to the other and should be explicitly detailed. All data processing steps should also be added, such as normalization, imputation, etc."*

Answer 4. The *Materials and Methods* section have been substantially expanded to include detailed information on sample preparation, TMT labeling, peptide pre-fractionation, LC-MS/MS run length, acquisition parameters, and MaxQuant settings. Additionally, all data processing steps including normalization, imputation, and statistical analysis are now described explicitly. Please see section "Generation of glioma lysates and proteomics analysis" pages 15 - 17.

"The proteome analysis was conducted at the VIB Ghent Proteomics Core. Sodium dodecyl sulfate (SDS) and triethylammonium bicarbonate (TEAB) was added to the sample to a final

concentration of 5% SDS, 50 mM TEAB, pH 8.5. Then, the sample was transferred to a 96-well PIXUL plate and sonicated with a PIXUL Multisample sonicator (Active Motif) for 10 minutes with default settings (Pulse 50 cycles, PRF 1 kHz, Burst Rate 20 Hz). After centrifugation of the samples for 15 minutes at maximum speed at room temperature (RT) to remove insoluble components, from each sample 100 µg of protein was isolated to continue the protocol. Proteins were reduced and alkylated by addition of 10 mM Tris(2-carboxyethyl)phosphine hydrochloride and 40 mM chloroacetamide and incubation for 10 minutes at 95°C in the dark. Phosphoric acid was added to a final concentration of 1.2% and subsequently samples were diluted 7-fold with binding buffer containing 90% methanol in 100 mM TEAB, pH 7.55. The samples were loaded on the 96-well S-Trap™ plate (Protifi), placed on top of a deepwell plate, and centrifuged for 2 min at 1,500 x g at RT. After protein binding, the S-trap™ plate was washed three times by adding 200 µl binding buffer and centrifugation for 2 min at 1,500 x g at RT. A new deepwell receiver plate was placed below the 96-well S-Trap™ plate and 50 mM TEAB containing 1 µg trypsin (1/100, w/w) was added for digestion overnight at 37°C. Using centrifugation for 2 min at 1,500 x g, peptides were eluted in three times, first with 80 µl 50 mM TEAB, then with 80 µl 0.2% formic acid (FA) in water and finally with 80 µl 0.2% FA in water/acetonitrile (ACN) (50/50, v/v). Eluted peptides were dried completely by vacuum centrifugation.

TMTpro™ 16-plex labels (0.5 mg, Thermo Fisher Scientific) were equilibrated to RT immediately before use and dissolved in 20 µl anhydrous ACN. The dried peptides were re-suspended in 100 mM TEAB (pH 8.5), peptide concentration was determined on a Lunatic spectrophotometer (Unchained Labs) (Maia et al., 2020) and peptide amount was adjusted to 40 µg in 30 µl for each sample. Peptides were labeled for 1 hour at RT using 0.5 mg of TMTPro™ label (labels used: 126, 128N, 131C & 133N: RSL3 replicates; 127N, 130C, 132N & 133C: MTX replicates; 127C, 131N, 132C, 134N: F/T replicates). The reaction was quenched for 15 min at RT by addition of 2 µl 5% hydroxylamine. The 12 labeled samples were combined, aliquoted in 100 µg aliquots, dried by vacuum centrifugation, re-dissolved in 100 µl loading solvent A (0.1% TFA in water/ACN (98:2, v/v)) and desalted on a reversed phase (RP) C18 OMIX tip (Agilent). The tip was first washed 3 times with 100 µl pre-wash buffer (0.1% TFA in water/ ACN (20:80, v/v)) and pre-equilibrated 5 times with 100 µl of wash buffer (0.1% TFA in water) before the sample was loaded on the tip. After peptide binding, the tip was washed 3 times with 100 µl of wash buffer and peptides were eluted twice with 100 µl elution buffer (0.1% TFA in water/ACN (40:60, v/v)). The combined elutions were dried in a vacuum concentrator.

Peptides were re-dissolved in 100 µl loading solvent A and 95 µl (+/- 100 µg) was injected for fractionation by RP-HPLC (Agilent series 1200) connected to a Probot fractionator (LC Packings). Peptides were first loaded in loading solvent A on a 4 cm pre-column (made in-house, 250 µm internal diameter (ID), 5 µm C18 beads, Dr. Maisch) for 10 min at 25 µl/min and then separated on a 15 cm analytical column (made in-house, 250 µm ID, 3 µm C18 beads, Dr Maisch). Elution was done using a linear gradient from 100% RP-HPLC solvent A (10 mM ammonium acetate (pH 5.5) in water/ACN (98:2, v/v)) to 100% RP-HPLC solvent B (70% ACN, 10 mM ammonium acetate (pH 5.5)) in 100 min at a constant flow rate of 3 µL/min. Fractions were collected every minute between 20 and 92 minutes and pooled every 24 minutes to generate a total of 24 samples for LC-MS/MS analysis. All 24 fractions were dried under vacuum in HPLC inserts and stored at −20°C until further use.

LC-MS/MS analysis

Purified peptides from all fractions were re-dissolved in 20 µl solvent A and 15 µl of each fraction was injected for LC-MS/MS analysis on an Ultimate 3000 RSLCnano system in-line connected to an Orbitrap Fusion Lumos mass spectrometer (Thermo Fisher Scientific) equipped with a pneu-Nimbus dual ion source (Phoenix S&T). Trapping was performed at 20 µl/min for 2 min in solvent A on a 5 mm PepMap™ Neo trapping column (300 µm ID, 5 µm beads, C18, Thermo Fisher Scientific, p/n 174500) and the peptides were separated on a 250 mm Aurora Ultimate, 1.7µm C18, 75 µm inner diameter (Ionopticks, AUR3-25075C18) kept at a constant temperature of 45°C. Peptides were eluted by a non-linear increase from 0.5 to 26.4% MS solvent B (0.1% FA in water/ACN (2:8, v/v)) over 52,5 minutes, then to 44% in 66.5 minutes followed by a 5-minutes wash at 70 minutes reaching 56% MS solvent B and re-equilibration with MS solvent A (0.1% FA in water) all at a constant flowrate of 250 nl/min.
The mass spectrometer was operated in data-dependent mode with a top speed of three seconds. Full-scan MS spectra (375-1500 m/z) were acquired at a resolution of 120,000 in the Orbitrap analyzer after accumulation to a target AGC value of 400,000 with a maximum injection time of 50 ms. The precursor ions were filtered for charge states (2-7 required), dynamic exclusion (60 s; +/- 10 ppm window) and intensity (minimal intensity of 5E4). The precursor ions were selected in the quadrupole with an isolation window of 0.7 Da and accumulated to an AGC target of 1E4 or a maximum injection time of 50 ms and activated using CID fragmentation (35% NCE). The fragments were analyzed in the Ion Trap Analyzer at turbo scan rate. 10 most intense MS2 fragments were selected in the quadrupole using MS3 multi-notch isolation windows of 2 m/z. An orbitrap resolution of 60,000 was used with an AGC target of 1E5 or a maximum injection time of 118 ms and activated using HCD fragmentation (65% NCE). The polydimethylcyclosiloxane background ion at 445.120028 Da was used for internal calibration (lock mass). QCloud was used to control instrument longitudinal performance during the project (Chiva et al., 2018).

Data analysis

LC-MS/MS runs for all samples were processed together using the MaxQuant algorithm (version 2.2.0.0) with default search settings, including a false discovery rate of 1% at both the precursor and protein levels. Spectra were matched against mouse protein sequences from the Swiss-Prot database (release version 2023_01), containing 17,137 sequences (www.uniprot.org).
Further statistical analysis was performed in R (version 4.1.1) using an in-house script. The proteinGroups output table was processed as follows. First, decoy proteins and proteins only identified by site were filtered out, leading to a list of 5,165 protein identitications. Only proteins with at least three quantification values in one experimental condition were kept, leading to a list of 5,162 proteins reliably quantified proteins. Next, reporter intensity-corrected values were log2 transformed and median-normalized. Missing values were imputed by random sampling from a normal distribution centered around the noise level (package DEP) (Zhang et al., 2018). Pairwise comparison testing between groups was then carried out using the limma package (Ritchie et al., 2015), with statistical significance for differential regulation set at an FDR cut-off value of 0.05 and a |log2(fold change)| above 1. Differentially abundant proteins detected over all comparisons were visualized in a heatmap following non-supervised hierarchical clustering of z-scored Log2 reporter intensity-corrected values."

Prediction of immunogenicity using the MyNeo platform has also been included. Please see Page 17 in the *Materials and Met*hods section.

"***In silico* prediction of immunoigenicity of immunopeptides**

To evaluate the immunogenic potential of expressed proteins, we performed in silico MHC presentation and immunogenicity predictions. Protein sequences were first fragmented into overlapping 9–11-mer and 15-mer peptides, corresponding to canonical MHC class I and class II epitope lengths, respectively. Peptide binding affinity to MHC molecules was predicted using MHCnuggets (Shao et al., 2020), a long short-term memory (LSTM) deep neural network trained on in vitro binding affinity datasets and immunopeptidomics data. Predictions were generated for the mouse MHC class I alleles H2-Db and H2-Kb, and the class II allele H2-Iab. Based on the predicted binding affinities, each peptide was ranked relative to a reference set of 100,000 peptides, yielding a percentile-based MHCrank score. Peptides with an MHC rank ≤ 2 were considered likely to be presented. Predicted MHC class I binders were further assessed for immunogenicity using neoIM, a random forest classifier trained to distinguish immunogenic from non-immunogenic MHCI peptides based on their physicochemical properties. Peptides with a neoIM (Pfitzer et al., 2022) score ≥ 0.64 were classified as strongly immunogenic, indicating a high likelihood of eliciting a $CD8^+$ T cell response."

Reviewer #3

"Here, Saviuk et al., reports the use of ferroptotic cell death to generate dendritic cell-based vaccines with increased anti-tumor activity compared to other form of cell death. The authors are the first to investigate the role of this type of cell death in vaccine development. They demonstrated the anti-tumor activity using a mouse model for intracranial glioma. Mechanistically, the authors reveal that the increased anti-tumor effect is dependent on three DAMPs: ATP, CRT and HMGB1. Additionally, the authors also showed long term protection of the anti-tumor effect and no added-value of the vaccine adjuvant LPS. The results are well presented and clearly described. The data is relevant and solid. The claims of this manuscript are well supported by the data. However, few concerns should be addressed before publication. Mainly, the authors should investigate further the mechanisms underlying the increased anti-tumor activity and fully report the proteomics data. The proteomics data might be a valuable resource to investigate further the mechanisms at play."

We sincerely thank Reviewer #3 for the thoughtful and positive evaluation of our work. We are grateful for the recognition of the novelty and relevance of our study, as well as the clarity and strength of the data presented. We also appreciate the reviewer's constructive suggestions to further investigate the mechanisms underlying the increased anti-tumor activity and to more fully report the proteomics data.

In response, we have expanded our *Results, Discussion and Materials and Methods sections* related to the proteomic analysis and have provided additional data supporting immunogenicity of ferroptosis. In additional, we have performed immunogenicity prediction of peptides which could be potentially presented by MHC-I and MHC-II molecules. We hope that these new data adequately address the reviewer's concerns. Please see the new figures (**Fig. 7, Suppl. Fig. 7** and **Suppl. Tables 1,2,3,4**) and the detailed responses regarding the proteomics data addressed to Reviewer #2.

Comment 1. *"The proteomics should be fully analyzed and presented. The method section is completely absent from the manuscript. Thus is is impossible to evaluate the quality of the data. The protein table is also not shared. The data should be deposited to a public proteomics repository. Additionally analysis should be performed: gene ontology, protein network analysis. The authors shows the differential regulation with a heatmap (what type or comparison? Pair-wise? What is the overlap between the conditions? The expression level of CRT and HMGB1 should be shown."*

Answer 1: We performed a more comprehensive analysis of the proteomics data. Specifically, we analyzed the up- and downregulation of proteins in ferroptotic (RSL3), apoptotic (MTX) and accidental necrotic (FT) lysates. We then performed an enrichment analysis to determine the functional activity of these peptides. The list of significantly altered proteins has been included as **Suppl Table 2**.

In addition, we analyzed the peptides derived from these proteins to predict their potential immunogenicity. The *Results* and *Materials and Methods* sections of the manuscript have been updated accordingly. Furthermore, we have included additional main and supplementary figures: **Fig. 7**, **Suppl. Fig. 7**.

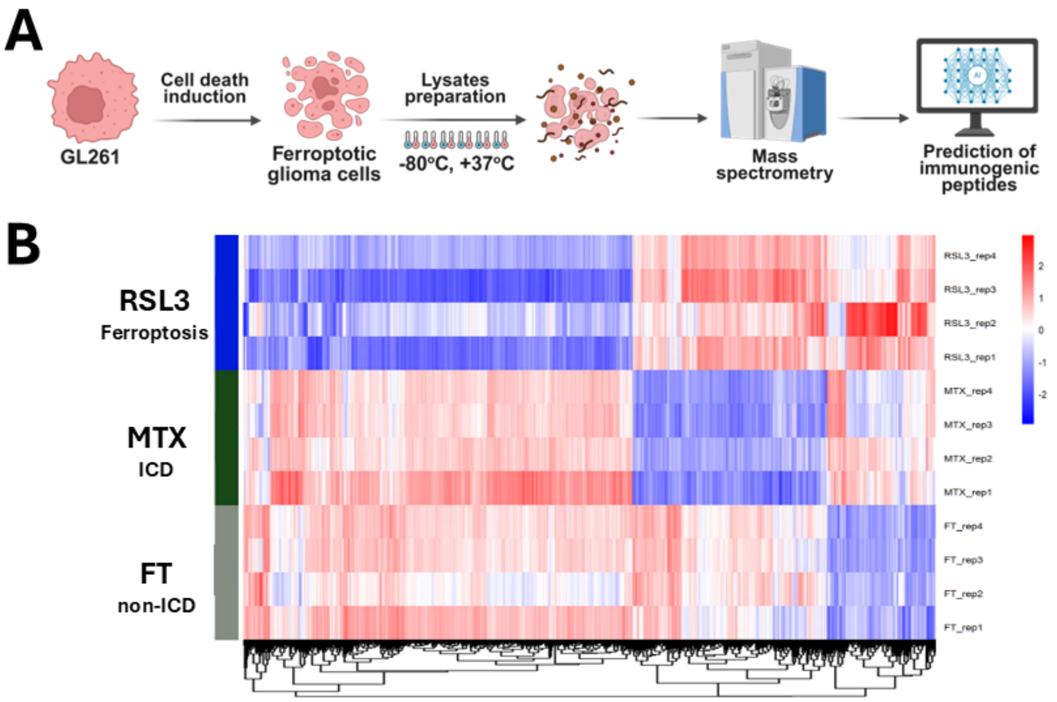

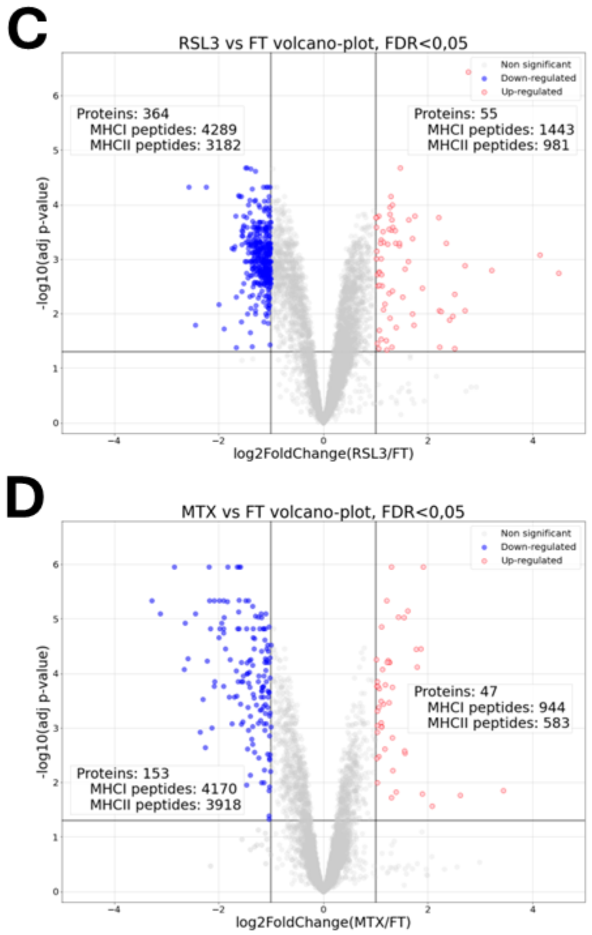

E

Up-regulated proteins which contain peptides common for RSL3 and MTX	
**MHCI**	**MHCII**
**289 peptides**	**178 peptides**
NAGPA	NAGPA
FIBG	FIBG
KIF23	KIF23
CENPE	CENPE
MMTA2	MMTA2
ANT3	ANT3
TROAP	TROAP
PRR11	PRR11
KI20A	KI20A
APOM	APOM
F13A	F13A
AURKA	AURKA
RETST	RETST
UPAR	

Figure 7. **(A)** Ferroptosis and immunogenic apoptosis were induced in GL261 cells using RSL3 (2.5 μM, 24 h) and MTX (2.5 μM, 24 h; ICD: positive control), respectively. As a negative control, GL261 cells were subjected to F/T (non-ICD). Proteomic analysis was then performed on the lysates used to load the DC vaccines. **(B)** Proteins differentially abundant between groups were visualized in a heatmap following un-supervised hierarchical clustering of z-scored $\log_2$ reporter intensity values. **(C, D)** Differential protein expression analysis comparing RSL3 vs. F/T **(C)** and MTX *versus* F/T **(D)**. **(E)** Commonly upregulated proteins which contain peptides shared between RSL3- and MTX-treated lysates.

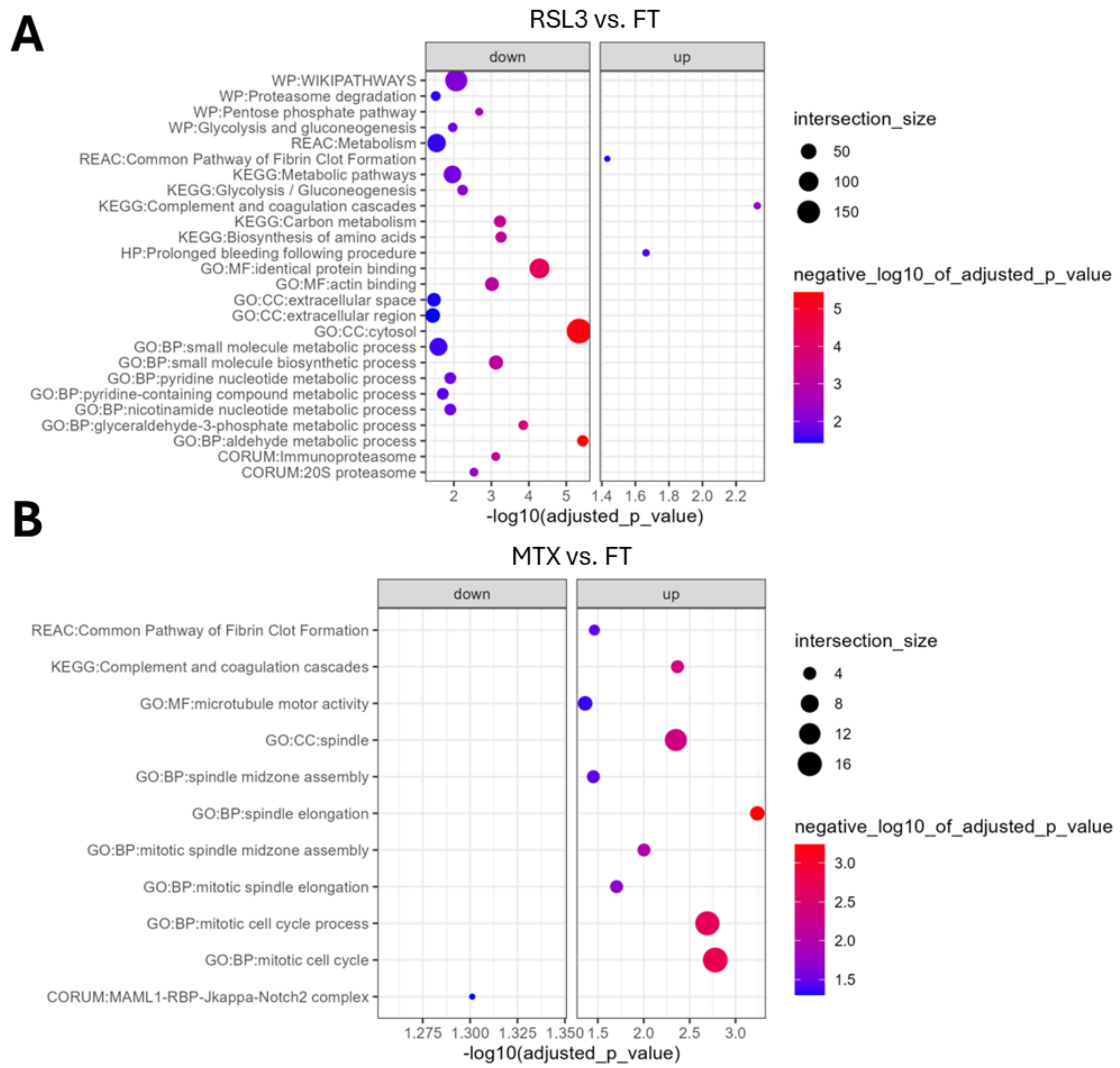

Suppl. Figure 7. **(A, B)** Gene Ontology (GO) enrichment analysis of differentially expressed genes (DEGs) in RSL3 *versus* F/T lysates (**A**) and MTX *versus* F/T lysates (**B**). Circle color represents the significance level of enrichment, and circle size indicates the number of enriched genes. Enrichment results with $p < 0.05$ were considered statistically significant.

Results section Page 8:

"To assess differences in proteins intensities between ferroptotic lysates (RSL3) and FT lysates (negative control: non-ICD) (**Fig. 7C**), as well as between MTX-treated lysates (positive control: ICD, MTX) and FT lysates (**Fig. 7D**), statistical comparisons of group means were performed. We identified 55 up-regulated and 364 down-regulated proteins in ferroptotic lysates compared to the FT group (non-ICD), and 47 upregulated and 153 downregulated proteins in MTX-treated lysates (ICD) compared to FT lysates. Functional enrichment analysis revealed a downregulation of proteins involved in metabolic processes in ferroptotic lysates (**Suppl. Fig. 7A**) and an upregulation of proteins associated with mitotic processes in MTX-treated lysates (**Suppl. Fig. 7B**)."

Comment 2. *"Overall, this is very nice work that significantly contributes to the state of the art of DC vaccine development. However, I do feel that the molecular mechanisms driving the effects of the ferroptotic lysates could have been studied more in-depth, for instance by exploring the proteomics dataset in more detail.*

Answer 2. In response, we have expanded our Results, Discussion and Materials and Methods sections related to the proteomic analysis (**Fig. 7 and Suppl. Fig. 7** and please see above the Answer 1) and have provided additional data supporting immunogenicity of ferroptosis.

In additional, we have performed immunogenicity prediction of peptides which could be potentially presented by MHC-I and MHC-II molecules (**Fig. 7E and Suppl. Table 3,4** and please see above the Answer 1). We have added the following text in the *Result section* on page 9:

"Next, we analyzed the peptides which can potentially be presented in the respective lysates derived from these proteins and predicted their immunogenicity in the context of murine MHC class I and II complexes. We identified 1,443 and 944 potentially MHC-I-presented immunogenic peptides, as well as 981 and 583 potentially MHCII-presented immunogenic peptides, in the ferroptotic and MTX-treated lysates, respectively. Notably, the ferroptotic and MTX-treated lysates shared 14 common upregulated proteins, giving rise to 289 potentially MHCI-presented immunogenic peptides. Additionally, three commonly upregulated proteins between the two lysates contained 178 potentially MHCII-presented immunogenic peptides (**Fig. 7E, Suppl. Tables 3, 4**). This distinct profile in ferroptotic lysates likely introduces novel antigenic determinants (Catanzaro et al., 2024), fostering tumor-targeting immune responses and contributing to the observed immunogenic properties of ferroptotic lysates in the murine orthotopic glioma model."

Comment 3. *"(FT) Could the effect of ferroptotic lysate be mimicked by adding ATP, recombinant HMGB1 and recombinant CRT in the lysate with cell death induced by freeze and thaw (FT-DC) or with MTX-induced lysate? Alternatively, other experimental design could be envisionsed. For instance, the authors could use ferroptotic cell death in mouse cells and mix this lysate with the glioma cell line GL261-FT (after freeze and thaw). One would expect that the induced DAMPs are sufficient for the increased stimulation of DC while the GL261 lysate will bring the (potentially) required antigens."*

Answer 3. As we have shown (**Fig. 8E, Suppl. Fig. 9A**), blocking HMGB1 does not affect the efficacy of ferroptosis-based DC vaccines; therefore, recombinant HMGB1 was not used to supplement FT lysates.

However, in response to this comment, we conducted experiments to restore DAMPs in FT lysates. Specifically, FT lysates were supplemented with recombinant CRT (3 µg/10^6 cells or ATP (1 µM) (Tesniere et al., 2010). Mice were vaccinated twice with these reconstituted FT lysates (non-ICD) derived from GL261 cells, followed by intracranial inoculation of viable GL261 cells 7 days after the second immunization.

We found that the reconstitution of CRT and ATP into FT lysates did not significantly improve mouse survival but did delay the onset of neurological symptoms (**Suppl. Fig. 10A-D**), suggesting that this delay reflects a modest attenuating of tumor progression, indicative of a partial anti-tumor immune response.

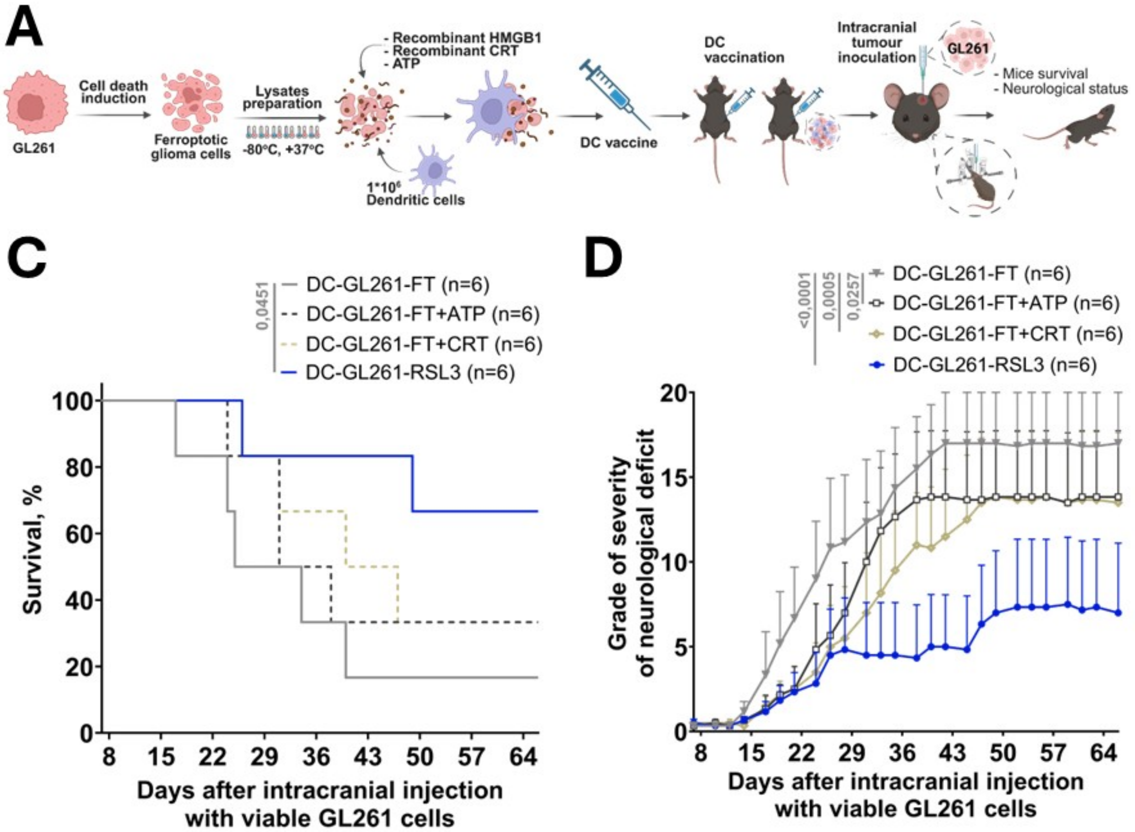

Suppl. Figure 10. **(A)** Procedure to evaluate the efficacy of pharmacological reconstitution of DAMPs (CRT and ATP) in an orthotopic intracranial mouse model. Mice received DC vaccines loaded with GL261 lysates subjected to freeze-thawing alone (non-ICD; DC-GL261-FT) or reconstituted with recombinant CRT (3 µg/mL; GL261-FT+CRT) or ATP (1 µM; GL261-FT+ATP). The corresponding DC vaccines were administered to mice on days -14 and -7. On day 0, mice were challenged intracranially with viable GL261 glioma cells. Mouse survival **(C),** neurological status **(D)** were monitored. Mouse survival was analyzed using the Mantel–Cox log-rank test. Neurological status was analyzed using two-way ANOVA followed by Tukey's multiple comparisons test.

The following text was added in the *Materials and Methods, Results and Discussion sections*:

Results on page 10:

"Interestingly, reconstitution of CRT and ATP into FT lysates did not significantly improve mouse survival but did delay the onset of neurological symptoms (**Suppl. Fig. 10A-D**), suggesting that this delay reflects a modest attenuation of tumor progression, indicative of a partial anti-tumor immune response."

Materials and Methods on page 20:

"Reconstitution of DAMPs in FT lysates: CRT and ATP

GL261 glioma cells were subjected to freeze-thaw cycles as described above. Recombinant cell-expressed human calreticulin CRT (3 µg/10^6 cells, kindly provided by Dr. Peter van Endert, Université de Paris, France) or ATP (1 µM, A7699, Sigma-Aldrich, Germany) was added to the lysates (Tesniere et al., 2010), which were then used to prepare DC vaccines following the previously described protocol. Mice were vaccinated on days -14 and -7 prior to intracranial inoculation of viable glioma GL261 cells."

Discussion on page 12:

"However, we found that the reconstitution of CRT and ATP into FT lysates did not significantly improve mouse survival but did delay the onset of neurological symptoms (**Suppl. Fig. 10A-D**), suggesting that while individual DAMPs can partially enhance immune activation, they are insufficient to fully replicate the complex immunogenic profile induced by ferroptotic cell death (Catanzaro et al., 2024). The lack of a survival benefit implies that ferroptosis triggers additional immunogenic signals beyond CRT and ATP, which are essential for effective tumor clearance and durable immune protection."

Comment 4. *"Mechanistically, the authors should comment why they investigated only a subset of the ICD-associated DAMPs (CRT, ATP and HMGB1)? The authors should investigate for more or provide a rational for the selection of these DAMPs and the exclusion of the others DAMPs? Is this selection motivated by the proteomics data?"*

Answer 4. One of the main objectives of this study was to evaluate the immunogenicity of ferroptosis, a topic that remains highly debated, with conflicting reports supporting both immunogenic (Efimova et al., 2020; Wang et al., 2025) and non-immunogenic outcomes (Kim et al., 2022; Wiernicki et al., 2022).

To ensure comparability with prior studies in the ICD field, we focused on the most widely accepted and well-characterized DAMPs: CRT, ATP, and HMGB1 (Fucikova et al., 2020; Galluzzi et al., 2020, 2023; Kepp et al., 2014; Kroemer et al., 2013). While we acknowledge other DAMPs such as HSP70, HSP90, and ANXA1 (Hayashi et al., 2020), our selection was driven by their established role as benchmark ICD markers in the literature. This targeted approach allows for a direct and meaningful comparison with existing ICD studies. Accordingly, we have added a clarifying sentence in the manuscript (page 9) explaining this rationale.

Given the wide variability in DAMP profiles across different ICD modalities, we chose to focus on CRT, ATP, and HMGB1, which are the most established and consistently used markers of ICD (Galluzzi et al., 2020, 2023; Kepp et al., 2014; Krysko et al., 2013).

Comment 5. *"The mechanisms associated for increased anti-tumor effects have been partially elucidated. However, the authors only focus on the adjuvant effect with LPS and few DAMPs. What about the antigenicity? Which tumor-associated antigens (TAA) or tumor-specific antigen (TSA) are driving the immune response against the tumor? The authors initiated this investigation with their proteomics data. However, they could further explore major histocompatibility presentation of the tumor antigens. This could be performed in targeted manner using known TAA/TSA or rather untargeted using mass spectrometry-based immunopeptidomics. Both MHC-II and MHC-I (cross-presentation) seems relevant in this model. Additionally, ferroptosis-induced cell death might reveal relevant antigens compared to other form of cell death. The authors mentioned TSA. Is this related to specific mutations? Or non-canonical protein? What are the known TSA in this glioma model?*

The authors should consider combining immunopeptidomics with the proteomics. It will be strongly appreciated if the authors attempt to reveal the cellular immunopeptidome presented on MHC class II and MHC class I (cross-presentation) by the DC vaccines."

Answer 5. We appreciate the reviewer's comment and fully acknowledge the importance of immunopeptidome in characterizing antigen presentation. Initially, we performed untargeted, mass spectrometry-based immunopeptidome analysis on DC vaccines loaded with ferroptotic lysates (DC-GL261-RSL3), apoptotic lysates (DC-GL261-MTX, positive control: ICD), necrotic lysates (DC-GL261-FT, negative control: non-ICD), and unloaded DCs. However, we observed

considerable inter-individual variability across samples, including notable discrepancies even among unloaded DCs (**Additional Fig. 1**).

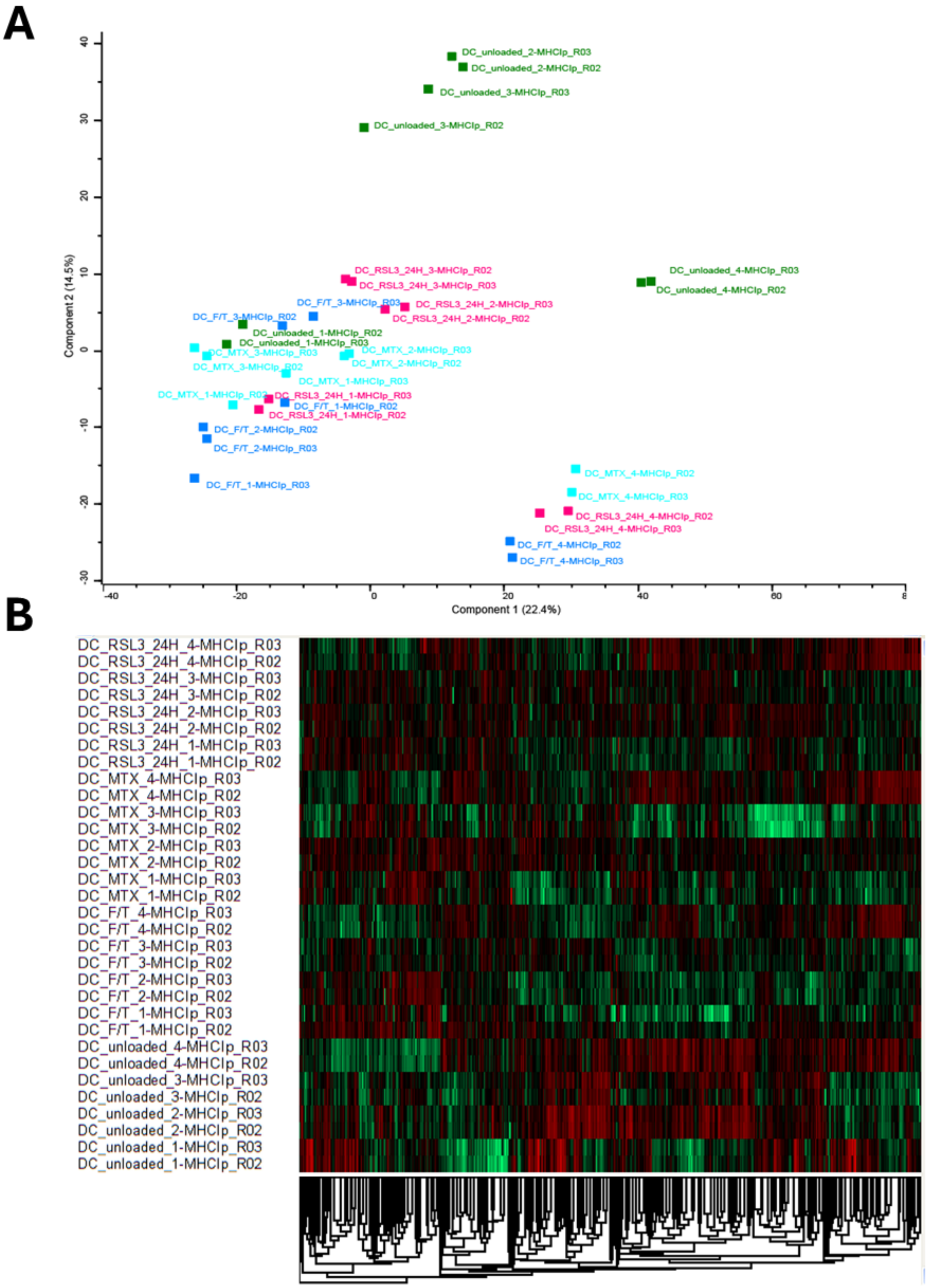

Additional Figure 1. **(A)** Principal component analysis (PCA). **(B)** Peptides differentially abundant between groups were visualized in a heatmap.

While repeating the experiment using DCs from a single mouse could have reduced variability, we believe this approach would not accurately reflect the biological heterogeneity inherent in therapeutic settings. Artificially minimizing inter-individual differences may lead to misleading

conclusions or false-positive findings. Given this variability and the substantial peptide overlap between groups, we decided not to overinterpret the immunopeptidomic data and opted against further experimental repetitions.

To address the reviewer's concern through an alternative strategy, we collaborated with MyNeo, a precision immunotherapy company specialized in neoantigen discovery and immune response prediction. MyNeo has developed proprietary bioinformatic pipelines integrating proteomics data with *in silico* MHC-binding predictions to identify immunogenic peptides with therapeutic relevance. Leveraging their expertise, we reanalyzed our proteomics dataset (**Fig. 7; Suppl. Fig. 7** and please see above the Answer 1), focusing on peptides potentially presented by MHC class I and II molecules. Furthermore, we performed *in silico* immunogenicity predictions to evaluate the likelihood of these peptides eliciting T cell responses (**Fig. 7E** and please see above the Answer 1).

Based on this comprehensive analysis, we expanded the Results, Discussion, and Materials and Methods sections to provide further data supporting the immunogenic potential of ferroptosis-derived peptides. This approach allowed us to derive more robust and clinically relevant insights, aligning with the reviewer's request to explore the antigenic landscape of ferroptosis-induced cell death. The additional data and text have been added to the *Results section* on pages 8-9. We have also added the corresponding information to the *Discussion* section on page 12 and to the *Materials and Methods* section on pages 15-17.

Minor comments

Minor Comment 1. *"What could be the advantage of the use of ferroptotic lysates compared to the DC vaccines that are currently being used in the clinic? Which types of lysates are currently being used? Some more context about this could be added to the introduction and/or the first paragraphs of the discussion."*

Answer 1. We appreciate the reviewer's comment and have now provided additional context regarding the current clinical use of DC vaccines and the potential advantages of ferroptotic lysates. Specifically, we have expanded the *Discussion* section (page 11) to highlight the following points:

1. Overcoming apoptosis resistance. Many tumors, including gliomas, develop resistance to therapies that induce apoptosis. Ferroptosis, as an alternative regulated cell death pathway, may overcome this resistance and enhance the efficacy of DC vaccines (Fulda, 2009; Hanahan & Weinberg, 2011).
2. Current clinical practice relies on non-immunogenic (*i.e.,* non-ICD) lysates. In several recent clinical trials, tumor lysates used for DC vaccination such as glioma lysates are often generated through physical disruption methods (*e.g.,* freeze-thaw cycles), which induce accidental necrosis rather than a defined immunogenic cell death pathway (Crespo-Diaz et al., 2011; Liau et al., 2023; Parney et al., 2022). It has been demonstrated that lysates prepared in this way lack immunogenicity (Aaes et al., 2016; Turubanova et al., 2019).

Therefore, in our study we also included a negative control group in which the viable glioma cells (*e.g.,* GL261 or CT2A) were subjected to several cycles of freeze-thawing, leading to accidental necrosis - *i.e.,* cell death without the induction of a specific regulated cell death pathway). We believe that including the negative control group (DC-GL261-FT) in our experiments allows for a proper benchmark against DCs loaded with ferroptotic lysates. Therefore, the DC-GL261-FT group was included in multiple experiments to enable this comparison.

In this way, the distinction between ferroptosis and accidental necrosis was demonstrated in the context of prophylactic vaccination **(Fig. 2)**, therapeutic vaccination **(Fig. 4B,C)** and the priming of cytotoxic T cell response **(Fig. 4E,F)**, as well as at the level of the immune cell infiltration **(Fig. 6, Suppl. Fig. 5)**.

The following text has been modified in the *Discussion section* on page 12:
"Notably, although the tumor cells targeted by the immune system do not undergo ferroptosis themselves, the induction of ferroptosis prior to lysate generation may represent a key step for producing efficient immunogenic lysates and subsequent DC vaccines by altering antigenicity through lipid peroxidation-induced modifications of protein and lipid structures (Catanzaro et al., 2024), enhancing antigen uptake, promoting the exposure of cryptic epitopes, and facilitating more effective cross-priming of T cells against tumor-associated antigens present in the parental tumor proteome. In line with this, ferroptosis-armed DC vaccination markedly enhanced $CD8^+$ T cell infiltration, activation, and effector memory differentiation within the glioma microenvironment, fostering robust tumor-targeting immune responses. Furthermore, our study highlights the critical role of ferroptosis-associated DAMPs in driving the efficacy of DC vaccines, reinforcing ferroptosis-based lysates as a promising platform for next-generation cancer immunotherapies."

To address this, we included a clinically relevant negative control group in which glioma cells (GL261 or CT2A) were subjected to freeze-thaw cycles (DC-GL261-FT), mimicking lysate preparation methods used in certain clinical trials (Hodi et al., 2025; Rapp et al., 2018). This allowed us to directly compare the immunogenicity of ferroptotic lysates (DC-GL261-RSL3) against necrotic lysates, establishing a more robust experimental benchmark.

Our findings indicate that DC vaccines loaded with ferroptotic lysates outperform those generated from accidentally necrotic cells in orthotopic glioma models. These results suggest that incorporating ferroptosis-based lysates into DC vaccine strategies may offer a more effective immunotherapeutic approach compared to current clinical lysate preparations.

Minor Comment 2. *"Figure 1: Based on the M&M sections, no solvent controls appeared to be used when using cell death-inducing or -inhibiting agents. Additionally, it was not mentioned in which solvent(s) the chemical compounds were dissolved. Solvents such as DMSO can already exert significant effects on cells, and can induce cell stress and even cell death when used at high final concentrations, acting as a confounder for the effect of the actual compounds. Ideally, these experiments should be repeated with proper solvent controls. Alternatively, the effect of the solvent alone should be tested."*

Answer 2. We thank the reviewer for this valuable comment. In response, we performed additional control experiments to assess the effects of DMSO and cell death inhibitors in the absence of cell death inducers. Neither DMSO nor the inhibitors alone caused significant

changes in cell viability (**Suppl. Fig. 1C,D**). These data have been included in the revised manuscript as supplementary figures.

Additionally, we have specified the solvents used for each compound in the *Materials and Methods* section for clarity on page 13.

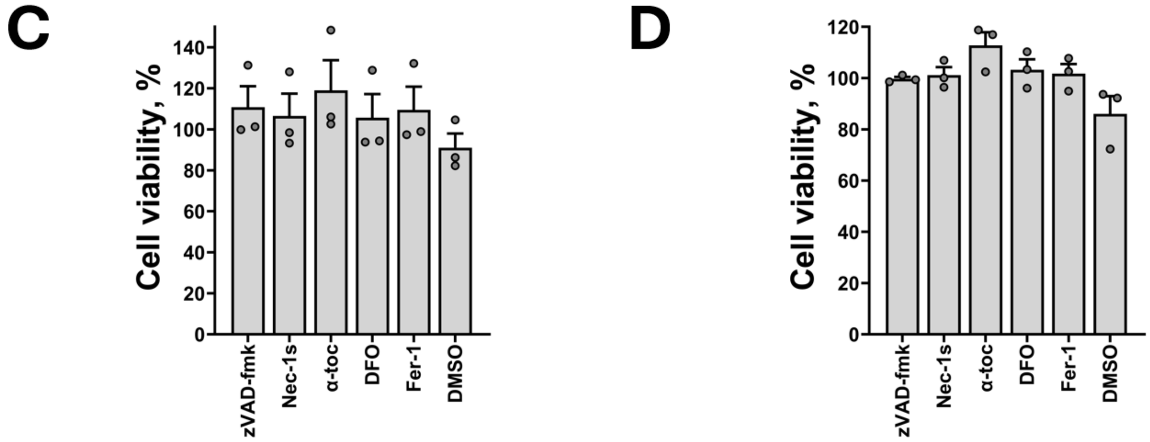

Suppl. Figure 1. (C, D) Cell death was analyzed using the MTS assay in glioma GL261 (**C**) and CT-2A (**D**) cells. The toxicity of cell death inhibitors or vehicle (DMSO) was assessed. Data are presented as mean ± SEM from three independent experiments. Statistical significance was determined using one-way ANOVA followed by Dunnett's multiple comparisons test.

Minor Comment 3. *"In Fig1: the authors tested the induction of ferroptotic cell death in two glioma cell lines. Why those two cell lines have a different response. Although this was briefly eluded in the text, it remains unclear.*
Figure 1B: The effect RSL3 on CT-2A cells appears to be dose-independent. Is there a potential explanation for this? Could the authors comment whether it would be relevant to rescue the low anti-tumor effect in CT2A cells?
May be, these cells have a reduced expression of DAMPs such as CRT or HMGB1? If yes, another proteomics investigation could be envisioned to reveal this.
Alternatively, the reduced anti-tumor effect might be driven by low expression of MHC I in CT-2A cells, could the authors comment whether rescuing low MHC-I expression with transduced overexpression be relevant?"

Answer 3. We thank the reviewer for these insightful comments. The differential sensitivity of GL261 and CT-2A cells to ferroptosis inducers likely reflects their distinct oncogenic drivers and immune phenotypes, which influence their responsiveness to cell death pathways and therapeutic interventions (Abate et al., 2006; Mikolajewicz et al., 2024; Szatmári et al., 2006). Specifically, CT-2A, which belongs to the mesenchymal glioma subtype, is characterized by elevated expression of ferroptosis suppressors, including GPX4 and ferroptosis suppressor protein 1, contributing to its innate resistance to lipid peroxidation and oxidative cell death (D'Aprile et al., 2024; Mikolajewicz et al., 2024). This likely accounts for the dose-independent response of CT-2A cells to RSL3 and SAS.

Regarding the reduced anti-tumor effect observed in CT-2A models, we agree that differences in DAMP release (*e.g.,* CRT, ATP, HMGB1) or MHC-I expression could be contributing factors. While a full proteomic comparison of DAMP expression between GL261 and CT-2A was beyond the scope of this study, future investigations could explore this aspect.

Minor Comment 4. *"Figure 1E-H: What is the effect of SAS on GSH and lipid peroxidase levels in the two cell lines?"*

Answer 4. We thank the reviewer for this comment. As shown in **Figure 1B**, CT-2A cells demonstrated strong resistance to SAS, with minimal cell death observed even at high doses.

Consequently, we did not pursue further experiments with SAS in CT-2A cells. Instead, we assessed the effect of SAS in GL261 cells. In these experiments, lipid peroxidation peaked 6 hours after treatment (**Suppl. Fig. 1E**), while GSH levels started to decline after 3 hours (**Suppl. Fig. 1G**). Importantly, the apoptosis inducer MTX did not affect lipid peroxidation or GSH levels in either GL261 or CT-2A cells (**Suppl. Fig. 1E-H**).

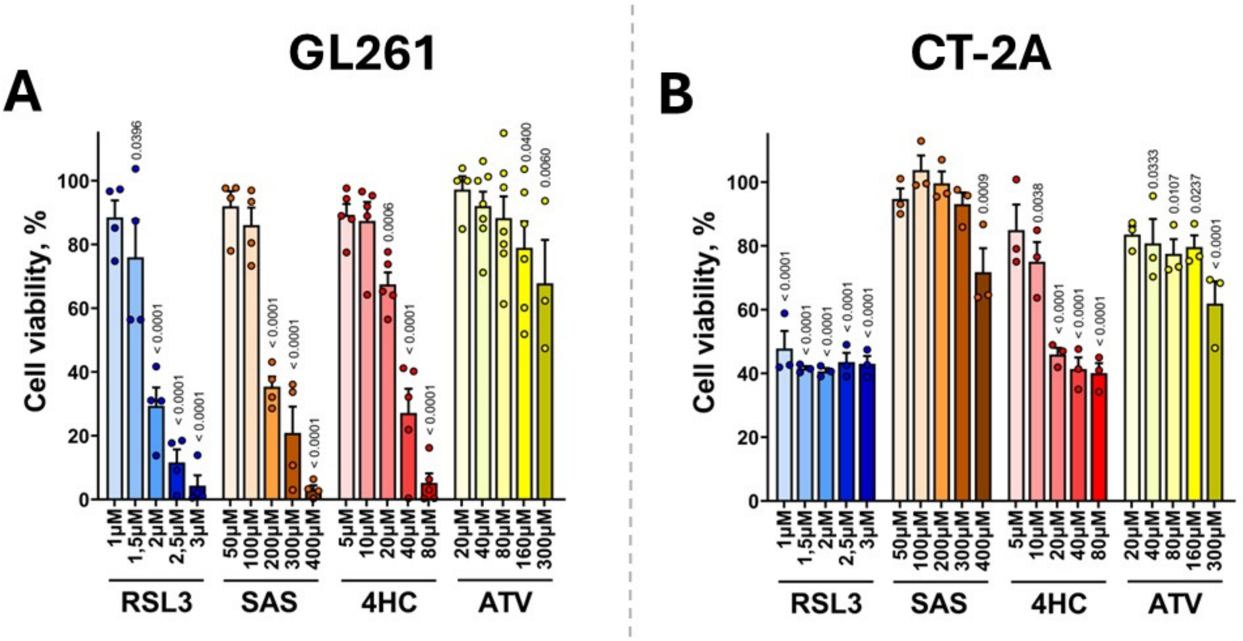

Figure 1. **(A, B)** Cell death was induced by RSL3 (1 µM – 3 µM), SAS (50 µM – 400 µM), 4HC (5 µM – 80 µM) or ATV (20 µM – 300 µM) for 24 hours and analyzed using MTS assay. RSL3, SAS, and 4HC efficiently induced cell death in GL261 cells **(A)**, but in CT-2A cells, only RSL3 and 4HC were effective **(B)**.

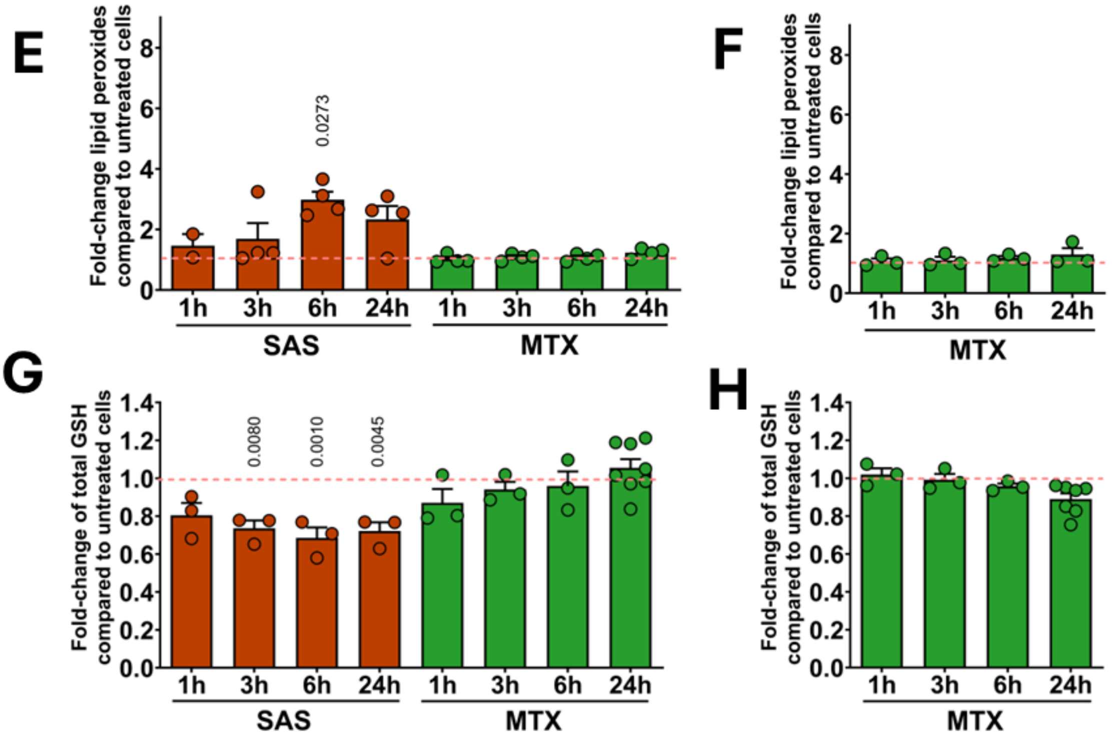

Suppl. Figure 1. **(E, G)** Lipid ROS (lipid peroxide) generation during cell death was assessed by treating GL261 cells with SAS (300 µM) or MTX (2.5 µM) for 1, 3, 6, or 24 hours (**E**), and CT-2A cells with MTX (2.5 µM) for the same timepoints (**G**), followed by BODIPY C11 staining. Data are presented as fold change relative to untreated cells.

(F, H) Glutathione (GSH) levels during cell death were assessed after treating GL261 cells with SAS (300 µM) or MTX (2.5 µM) for 1, 3, 6, or 24 hours (**F**), and CT-2A cells with MTX (2.5 µM) for the same timepoints (**H**). Data are presented as fold change relative to

untreated cells. Values are expressed as mean ± SEM from 3–7 independent experiments. Statistical significance was determined using one-way ANOVA followed by Dunnett's multiple comparisons test or the Kruskal-Wallis test with Dunn's multiple comparisons correction.

Minor Comment 5. *"Figure 2: for me, as a non-expert in in vivo studies, it is unclear why reaching the humane endpoints (or not) determines whether or not the mice were included in the analysis.*
As a side note, it would also be interesting to know whether there's statistically significant differences between the RSL3, FT and MTX groups."

Answer 5. We appreciate the reviewer's comment and have clarified this point in the figure legend. In the prophylactic vaccination experiment (**Fig. 2**), MRI scans were performed shortly before the experimental endpoint. At that time, several mice had not yet reached humane endpoints or exhibited neurological symptoms. However, due to facility constraints, it was not possible to return the mice to the animal facility after MRI; thus, all mice undergoing MRI had to be euthanized on the same day.

Three mice per group were subjected for MRI. Among them, two mice from the PBS group and two from the unloaded DC group had already reached humane endpoints and were included in the survival analysis. The remaining mice (one from each of the PBS, DC, DC-GL261-FT, DC-GL261-MTX, and DC-GL261-RSL3 groups) had not yet reached humane endpoints and were euthanized solely due to experimental logistics. Including these mice in the survival analysis would have misrepresented their cause of death as tumor progression, which is why they were excluded.

Regarding the survival differences between treatment groups, we agree this is an important point and have now included a statistical comparison of survival curves in the revised manuscript (**Fig. 2**).

Minor Comment 6. *"Fig. 2-3: The statistical evaluation of neurological deficits should not be done via multiple unpaired t-tests, as these do not correct for multiple comparisons.*
All groups should be compared in one statistical test, such as one-way ANOVA.
Were the Mantel-Cox tests done on all groups at once, or was each experimental group also separately compared to the PBS here?
Fig. 4A-C: these data should be analyzed via two-way ANOVA."

Answer 6. We have re-analyzed the neurological deficit scores and dendritic cell maturation data using two-way ANOVA, incorporating both time (days post-inoculation) and treatment group as factors. This approach corrects for multiple comparisons across groups and time points, providing a more robust statistical evaluation.

Regarding the survival analysis, we performed hypothesis-driven comparisons based on pre-specified, biologically justified expectations. In such cases, unadjusted p-values are accepted as informative (Perneger, 1998). Applying stringent corrections like Bonferroni in small-sample, hypothesis-driven preclinical studies may obscure biologically meaningful effects by inflating type II error rates (Zhu & Lu, 2015). Given the clear and consistent treatment effects observed on neurological deficits (**Figs. 2–4**), antigen presentation (**Figs. 4D–F**), and T-cell activation (**Fig. 6**), we believe our current approach to survival analysis remains appropriate and scientifically justified.

Minor Comment 7. *"The authors state the following regarding the data in Fig. 3G: "This response was observed in the ferroptotic lysate group and was not observed in control groups vaccinated with PBS, DC alone, or DC vaccines based on non-ICD lysates". The data do not fully support this statement, as there is no significant difference between the empty DC, DC-GL261-SIINFEKL-FT and DC-GL261-SIINFEKL-RSL3. Similar claims are being made in the discussion section."*

Answer 7. We acknowledge the reviewer's comment and have addressed this point by generating new experimental data. Following the Reviewer #3 comment, we have performed new experiments (**Fig. 4D-F**).

The experimental design included the use of DC vaccines loaded with glioma GL261 glioma cells subjected to F/T cycles (negative control: non-ICD, DC-GL261-OVA-FT), treated with MTX (2.5 μM, positive control: ICD, DC-GL261-OVA-MTX), as well as unloaded DCs and PBS (both negative controls). A significant increase in T cell responses was observed in the DC-GL261-OVA-RSL3 (ferroptosis) and DC-GL261-OVA-MTX (ICD, positive control) groups, but not in the negative control groups DC-GL261-OVA-FT, DC and PBS. These new data have been added in the revised version of the manuscript.

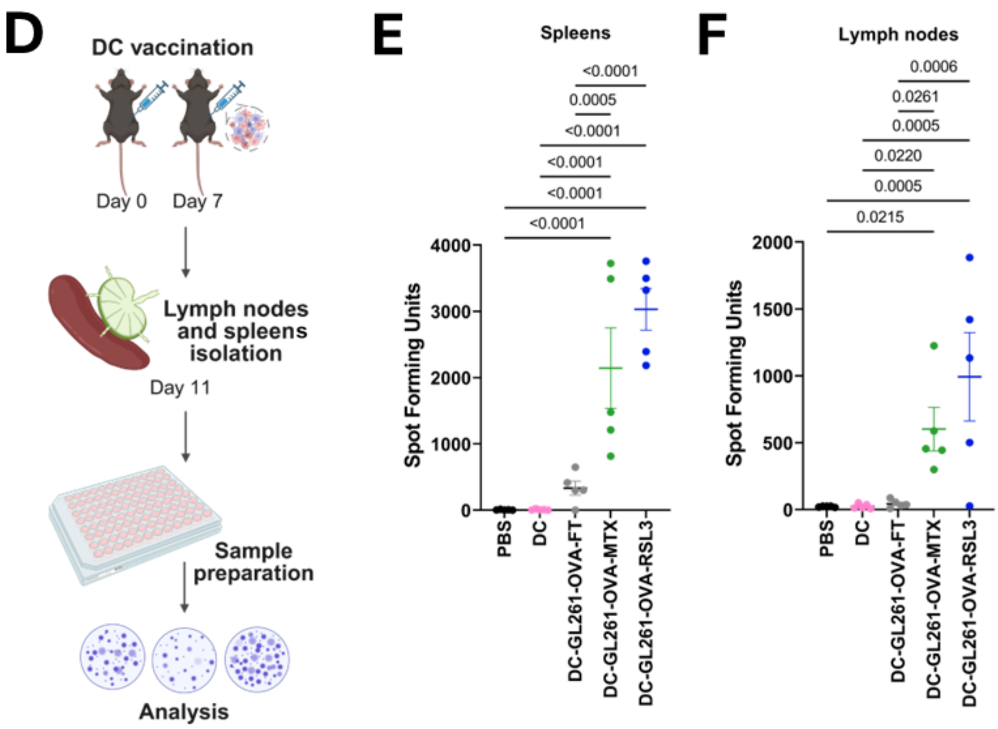

Figure 4. (D) Procedure to analyze the priming of cytotoxic T cells and IFN-γ production in mice immunized with DC vaccines on days 0 and 7. Four days after the final vaccination, the inguinal lymph nodes and splenocytes were isolated and re-stimulated *ex vivo* with the SIINFEKL peptide. **(E, F)** IFN-γ ELISpot assay of the spleens **(E)** and draining lymph nodes **(F)** from immunized mice. DC vaccines were loaded with ferroptotic GL261 lysates (DC-GL261-OVA-RSL3). Ferroptosis was induced by 2.5 μM RSL3. As controls, we used DC vaccines loaded with glioma GL261 cells subjected to F/T cycles (negative control: non-ICD, DC-GL261-OVA-FT), with MTX (2.5 μM, positive control: ICD, DC-GL261-OVA-MTX), unloaded DCs (negative control, DCs) or PBS (negative control). Each type of lysate was supplemented with 1 μM ovalbumin. The number of spot-forming units is presented as means ± SEM from data obtained from 5 mice. Statistical significance was determined using one-way ANOVA followed by Dunnett's multiple comparisons test.

Minor Comment 8. *"Did the authors investigate whether the different lysate (e.g. freeze thaw) have a lower amount of ATP, CRT, HMGB1?"*

Answer 8. We have quantified key DAMPs associated with ICD: surface-exposed calreticulin (CRT), and extracellular ATP and HMGB1 (**Fig. 8A-C**), confirming the pronounced release of these DAMPs signals during ferroptosis. Additionally, functional blocking experiments demonstrated that neutralizing CRT and ATP, but not HMGB1, diminished the immunogenicity of ferroptotic lysates. This finding is further supported by experiments using DCs derived from TLR4 knockout mice (**Fig. 5F-H, Suppl. Fig. 3C-D**), which exhibited similar anti-tumor efficacy as wild-type DCs

(**Suppl. Fig. 3E-G**). Given that TLR4 is a primary receptor for HMGB1 (Apetoh, Ghiringhelli, Tesniere, Obeid, et al., 2007), these data suggest a limited role of HMGB1 in mediating the immunogenic effects of ferroptotic lysates.

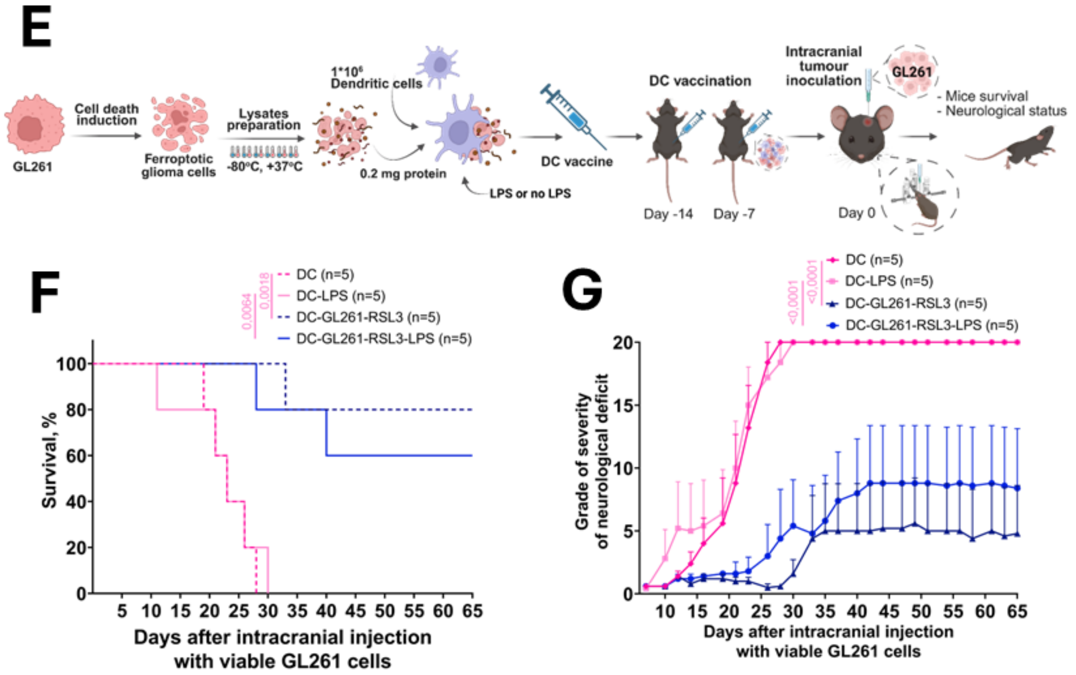

Suppl. Figure 3. **(E)** Procedure for generating ferroptosis-armed DC vaccines from wild-type (WT) mouse bone marrow-derived DCs (WT-DCs). WT-DCs were co-cultured for 90 minutes with ferroptotic GL261 lysates (RSL3, 2.5 µM, 24 h), and then further incubated with the same lysates in the presence (DC-GL261-RSL3-LPS) or absence (DC-GL261-RSL3) of 0.5 µg/mL LPS for 24 hours. WT mice received the respective DC vaccines on days –14 and –7, followed by intracranial injection of viable GL261 glioma cells on day 0. As controls, mice were treated with unloaded DCs, either LPS-stimulated (DC-LPS) or unstimulated (DC).
(F, G) Mouse survival **(F)** and neurological status **(G)** were monitored up to day 65. Survival data were analyzed using the Mantel-Cox log-rank test. Neurological status was analyzed using two-way ANOVA followed by Tukey's multiple comparisons test.

Minor Comment 9. *"To appreciate better the added value of DC-GL261-RSL3, DC-GL261-MTX should be consistently included in the whole manuscript. It is unclear why this was included in some experiments but not in other."*

Answer 9. These data have been incorporated into the manuscript, and the *Materials and Methods*, *Results,* and *Discussion* sections have been adapted accordingly.

Minor Comment 10. *"The Figure 4E-G shows two key data. (1) LPS has no added value in vivo for anti-tumor activity (Fig 4F) and (2) vaccinated mice surviving the initial tumor challenge show a prolonged immune response after second tumor challenge at day 56. This is nice data but this would have been more appreciated on wild-type DC vaccine instead of TLR4- KO DC vaccines."*

Answer 10. We have repeated the experiment using wild-type DCs, following the same vaccination schedule. As shown in **Suppl. Fig. 3E–G** (please see above the Answer for Minor Comment 8), the addition of LPS did not enhance the efficacy of ferroptosis-based DC vaccines derived from wild type mice. These findings support our conclusion that LPS does not contribute to the anti-tumor effect in this context and have been incorporated into the main conclusions of the manuscript The *Materials and Methods, Results,* and *Discussion* sections have been adapted accordingly.

Minor Comment 11. *"In figure 4F, as mentioned above, the proteomics data should be deposited in a proteomics repository such as PRIDE.*
This data deserves more investigation. The authors only looked at the proteins exclusively regulated.

What is the overlap between the conditions? Gene ontology?
If relevant (Figure 5?), the authors could show the expression level of HMGB1 and CRT with a bare plot, a s-curve showing log2 IBAQ intensity and the rank protein according to abundance.
Additionally, a schematic to describe the experimental design could be included."

Answer 11. The proteomics data have been deposited in the PRIDE repository with the following credentials:
Project accession: PXD060116
Access via token: iVY7HVrAMhq0

We have thoroughly re-analyzed the proteomic dataset, including overlap analysis and gene ontology enrichment **Fig. 7; Suppl. Fig. 7** and please see above the Answer 1)
In additional, we have performed immunogenicity prediction of peptides which could be potentially presented by MHC-I and MHC-II molecules (**Fig. 7, Suppl. Table 3,4** and please see above the Answer 1).
A schematic of the experimental design has been added (**Fig. 7A** and please see above the Answer 1) for clarity.
The *Materials and Methods* section has been significantly expanded to detail the proteomic workflows, and relevant updates have been made to the *Results* and *Discussion* sections.

Minor Comment 12. *"Suggestion: May be figure 4H should be move to a separate figure as it is not related to the investigation of LPS. Of note, figure 4H is a bit related to figure 5 where the authors investigate the antigenicity of they ferroptotic DC vaccines."*

Answer 12. The proteomics heatmap (previously **Fig. 4H**) has been moved to a new figure (**Fig. 7B**) for improved clarity and thematic consistency.
Additional proteomics-related data, including supplementary figures (**Suppl. Fig. 7)** and detailed tables (**Suppl. Table 1-4**), have been incorporated to provide a comprehensive overview of the analysis.

Minor Comment 13. *"In Fig. 5, the authors observe that CRT expression is actually much more increased with MTX than with RSL3. A similar comparison between RSL3 and MTX should be done for ATP and HMGB1.*
Furthermore, more detailed explanation/exploration is missing about how the authors link the stronger increase of CRT with MTX to the lower effectiveness of MTX lysates in vivo. Likely, CRT is only one factor driving the efficacy of the ferroptotic DC vaccines in vivo. Further analysis of the proteomics data might be helpful to reveal additional mechanisms"

Answer 13. We appreciate the reviewer's observation. Our study primarily focused on investigating the immunogenic properties of ferroptotic lysates rather than conducting a direct mechanistic comparison between RSL3-induced ferroptosis and MTX-induced apoptosis. While MTX is known to induce robust DAMP release, including CRT, ATP, and HMGB1, our data confirm higher surface CRT exposure with MTX, consistent with previous reports.
However, as noted by the reviewer, CRT expression alone does not fully explain the *superior in vivo* efficacy of ferroptotic DC vaccines. Although we did not experimentally explore additional mechanisms in this study, we hypothesize that ferroptosis-induced oxidative modifications may enhance antigenicity, contributing to more effective T-cell priming (Catanzaro et al., 2024). This potential synergy between DAMP release and antigen quality could underlie the observed differences in vaccine efficacy and warrants further investigation in future studies.

Our study aimed to explore the immunogenic properties of ferroptosis, rather than performing a head-to-head comparison between RSL3 (ferroptosis) and MTX (apoptosis). Both RSL3 and MTX represent distinct pathways of regulated cell death, each with potential relevance for cancer immunotherapy.

MTX is known to induce immunogenic apoptosis, resulting in the release of DAMPs such as CRT, ATP and HMGB1 (Galluzzi et al., 2020; Kepp et al., 2014; Liu et al., 2017). The higher surface CRT exposure observed with MTX in our experiments is consistent with these earlier findings.

However, as the reviewer notes, the presence of CRT alone does not fully predict vaccine efficacy. Despite higher CRT levels, the apoptotic-based DC vaccine shows lower in vivo survival compared to the ferroptotic-based DC vaccine (**Fig. 2–**4). This suggests that CRT is necessary but not sufficient for potent immune activation alone.

We believe this indicates a synergistic effect between adjuvanticity (DAMP release) and antigenicity. Ferroptotic cells may generate unique oxidative post-translational modifications (*e.g.,* lipid peroxidation and carbonylation) that could enhance antigen diversity or processing. This would lead to stronger T-cell priming, even if some DAMPs (*e.g.,* CRT) were less prominent.

Minor Comment 14. *"Fig 5A what is the control? The authors should repeat the experiment but including the freeze-thaw control "FT" condition (non-ICD)."*

Answer 14. Figure 5A (now **Fig. 8A**) uses viable cells as the negative control, as surface CRT exosure was measured on dying ferroptotic cells (on Sytox Green/Blue negative cells) by flow cytometry. FT-treated cells, being non-viable after repeated freeze-thaw cycles, cannot be included in this assay as they would only show intracellular CRT staining combined with Sytox positivity, indicating membrane permeabilization rather than surface CRT exposure. Therefore, viable cells are the appropriate control for this experiment. The name of the control group in the figure has been changed to "non treated" for clarity.

Minor Comment 15. *"Fig 5B shows the FACS related ot figure fig 5A. to be complete, the authors should show all the same groups for fig 5A and fig 5B."*

Answer 15. Upon review, we noticed that **Fig. 5B** (now removed) and **Suppl. Fig. 8** displayed the same dataset. To avoid redundancy and improve clarity, we have retained only **Suppl. Fig. 8**.

Minor Comment 16. *"In Figure 5, the authors hypothesize that the superior protection of the ferroptotic lysates is among others driven by ATP and CRT. However, CRT upregulation is much more pronounced in MTX-treated lysates, yet these MTX-treated lysates are less effective in vivo, when loaded onto DCs. How could these observations be explained?"*

Answer 16. We acknowledge the reviewer's observation. In our prophylactic and therapeutic vaccination experiments (**Figs. 2,3,4A–C**), MTX-based and RSL3-based (ferroptotic) DC vaccines showed comparable efficacy, and MTX was used only as a positive control throughout the study. The DAMP-blocking experiments were designed to explore the mechanisms contributing to the immunogenicity of ferroptotic lysates specifically, rather than to perform a direct comparative analysis between ferroptosis and apoptosis. We agree that additional factors beyond CRT exposure, such as antigenicity and oxidative modifications (Catanzaro et al., 2024), may play a role and warrant further investigation in future studies.

Minor Comment 17. *"Figure 5C and 5D should also include the condition with MTX to be consistent. It would be interesting to also test FT to evaluate the effect of non-ICD."*

Answer 17. We appreciate the reviewer's suggestion. Our primary focus was to investigate the immunogenic mechanisms of ferroptosis, which is why MTX was not included in Figures 5C and 5D (now **Fig. 8B and 8C**). The induction of DAMPs by MTX has been extensively documented in previous studies across multiple cancer cell lines (Li et al., 2020; Liu et al., 2017; Sukkurwala et al., 2014).

Regarding the FT condition, since these cells are fully necrotic and lack membrane integrity, they cannot actively expose DAMPs like CRT, which limits their relevance in surface exposure assays. For this reason, viable untreated cells were used as the appropriate negative control.

To maintain focus and clarity in the main manuscript, these figures have been moved to the supplementary section (**Suppl. Fig. 10**), where they continue to provide important mechanistic context.

Minor Comment 18. ***"****The experiments in figure 5E-I are very impressive with very nice data. This data is already very convincing and suggest that all components are required for the increased anti-tumor effect.*

Did the authors consider reconstituting the increased anti-tumor effect using the DC-GL261-FT supplemented with HMGB1, CRT and ATP? The data suggests that removing one of these three components is sufficient to block the increased anti-tumor effect. However, one could speculate that other DAMPs required for this effect? Thus, it seems worthwhile to investigate whether adding those three DAMPs could be sufficient for the increased anti-tumor effect.

Line 309: "The emission of these three key DAMPs may function as additional immunostimulatory adjuvants in DC vaccines." The experiments in figure 5E-I are very impressive. This data suggest that the three components are required for the increased anti-tumor effect. Did the authors consider reconstituting the increased anti-tumor effect using the DC-GL261-FT supplemented with, CRT and ATP? but also HMGB1? The data suggests that removing one components is sufficient to block the increased anti-tumor effect.

Additionally, one could speculate that other DAMPs required for this effect? Thus, it seems worthwhile to investigate whether these DAMPs are sufficient for the increased anti-tumor effect. Adding these DAMPs could be sufficient to restore the increased anti-tumor effect. If not, there might be other DAMPs required for the increased anti-tumor effect. Those unknown DAMPs might be hidden in the available proteomics data."

Answer 18. We sincerely thank the reviewer for this insightful suggestion. To explore whether supplementing FT lysates with key DAMPs could restore their anti-tumor efficacy, we performed reconstitution experiments. Specifically, FT lysates were supplemented with recombinant CRT (3 µg/10^6 cells) and ATP (1 µM), followed by a prophylactic vaccination schedule. Mice received two vaccinations before intracranial GL261 tumor challenge.

Our results showed that while the addition of CRT and ATP to FT lysates did not significantly enhance overall survival, it did delay the onset of neurological symptoms compared to FT alone (**Suppl. Fig. 10A–D** and please see above the Answer 3). This suggests a partial restoration of immunogenicity, but not to the level observed with ferroptotic lysates, indicating that other factors are likely contributing to the full anti-tumor effect.

Regarding HMGB1, as demonstrated in our blocking experiments (**Fig. 8E; Suppl. Fig. 9A**), its neutralization did not diminish the efficacy of ferroptosis-based DC vaccines. Based on this, we prioritized CRT and ATP for the reconstitution assays.

We agree with the reviewer that other, yet unidentified DAMPs or perhaps ferroptosis-specific oxidative modifications may play a crucial role, and our proteomics data provide a valuable resource for future investigation.

The *Materials and Methods, Results,* and *Discussion* sections have been updated accordingly.

Minor Comment 19. *"Did the authors consider investigating whether CD8 or CD4 T cells are expected to be most relevant for ferroptotic DC vaccines? What happens if CD8 and CD4 t cells are functionally blocked with antibody."*

Answer 19. We appreciate the reviewer's valuable suggestion. While we did not perform functional CD4/CD8 depletion experiments, we conducted a detailed immunoprofiling of the tumor microenvironment (**Fig. 6; Suppl. Fig. 5**). In mice treated with the ferroptosis-based DC vaccine, we observed a significant increase in $CD8^+$ T-cell infiltration (**Fig. 6B**), suggesting their key role in mediating the anti-tumor response.

We have included this information in the *Results* section to emphasize the involvement of $CD8^+$ T cells. Further mechanistic studies, such as CD4/CD8 depletion assays, would be of interest to dissect the precise contribution of these subsets in future work.

We performed immunoprofiling of tumour-infiltrating cells (**Fig. 6 and Suppl. Fig. 5**) and observed a significant increase in $CD8^+$ T cells in the group of mice treated with the ferroptosis-based DC vaccine (**Fig. 6B**). We have added an entirely new section entitled *"Ferroptotic DC vaccines enhance CD8+ T cell infiltration and activation in the glioma microenvironment"*, dedicated to the analysis of the tumor microenvironment and the role of ferroptosis-armed DCs in modulating the tumor microenvironment.

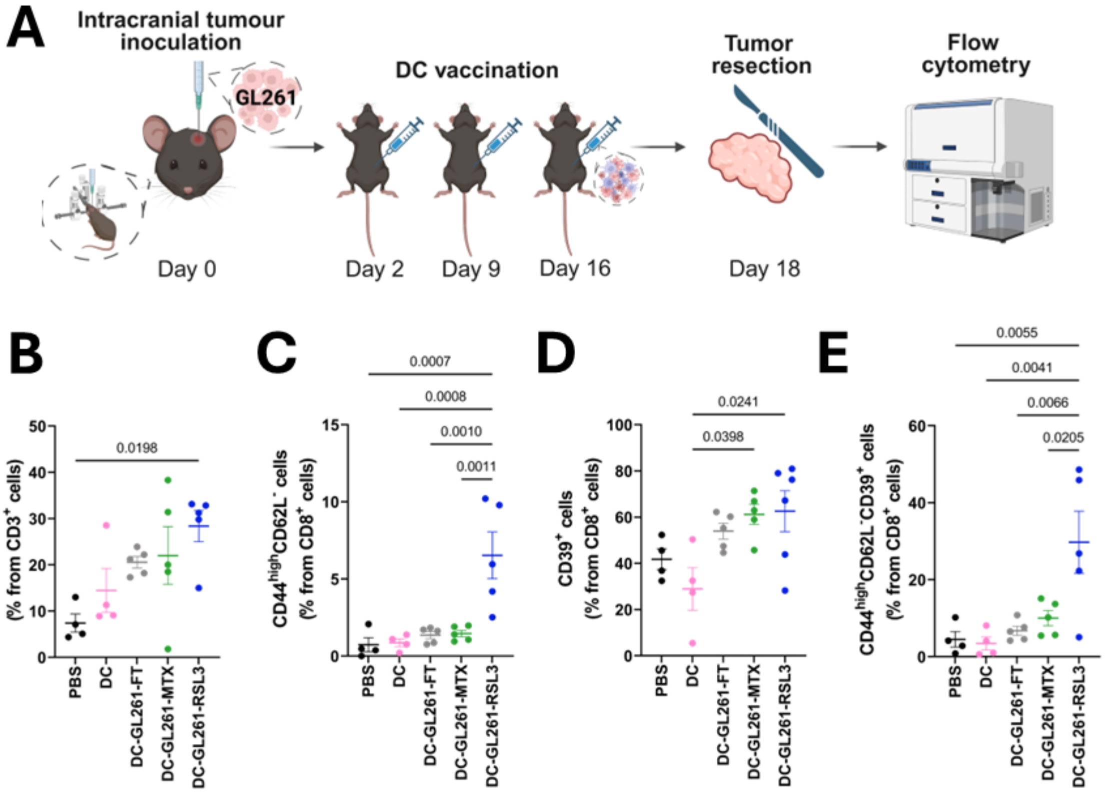

Figure 6. **(A)** Procedure to assess T-cell infiltration induced by DC vaccines in a therapeutic setting using an orthotopic intracranial mouse model. Mice were injected intracranially with viable glioma GL261 cells on day 0. Subsequently, they received the corresponding DC vaccine on days 2, 9 and 16. On day 18 after tumor inoculation, intracranial tumors were isolated, and immune cells were analyzed by multicolor flow cytometry. Control groups included mice treated with DC vaccines loaded with glioma GL261 lysate subjected to F/T cycles (negative control: non-ICD, DC-GL261-FT), DC vaccines loaded with MTX-treated lysates (2.5 μM, positive control: ICD, DC-GL261-MTX), unloaded DCs (negative control, DCs) or PBS (negative control). **(B-E)** Percentage of $CD8^+$ T cell infiltration. The ferroptosis-armed DC vaccine increased the infiltration of total $CD8^+$ T cells **(B)**, effector memory $CD8^+$ T cells ($CD44^+CD62L^-$ **C)**, $CD39^+$ exhausted $CD8^+$ T cells **(D)** and $CD39^+$ effector memory $CD8^+$ T cells **(E)**. Statistical significance was determined using one-way ANOVA followed by Dunnett multiple comparisons test.

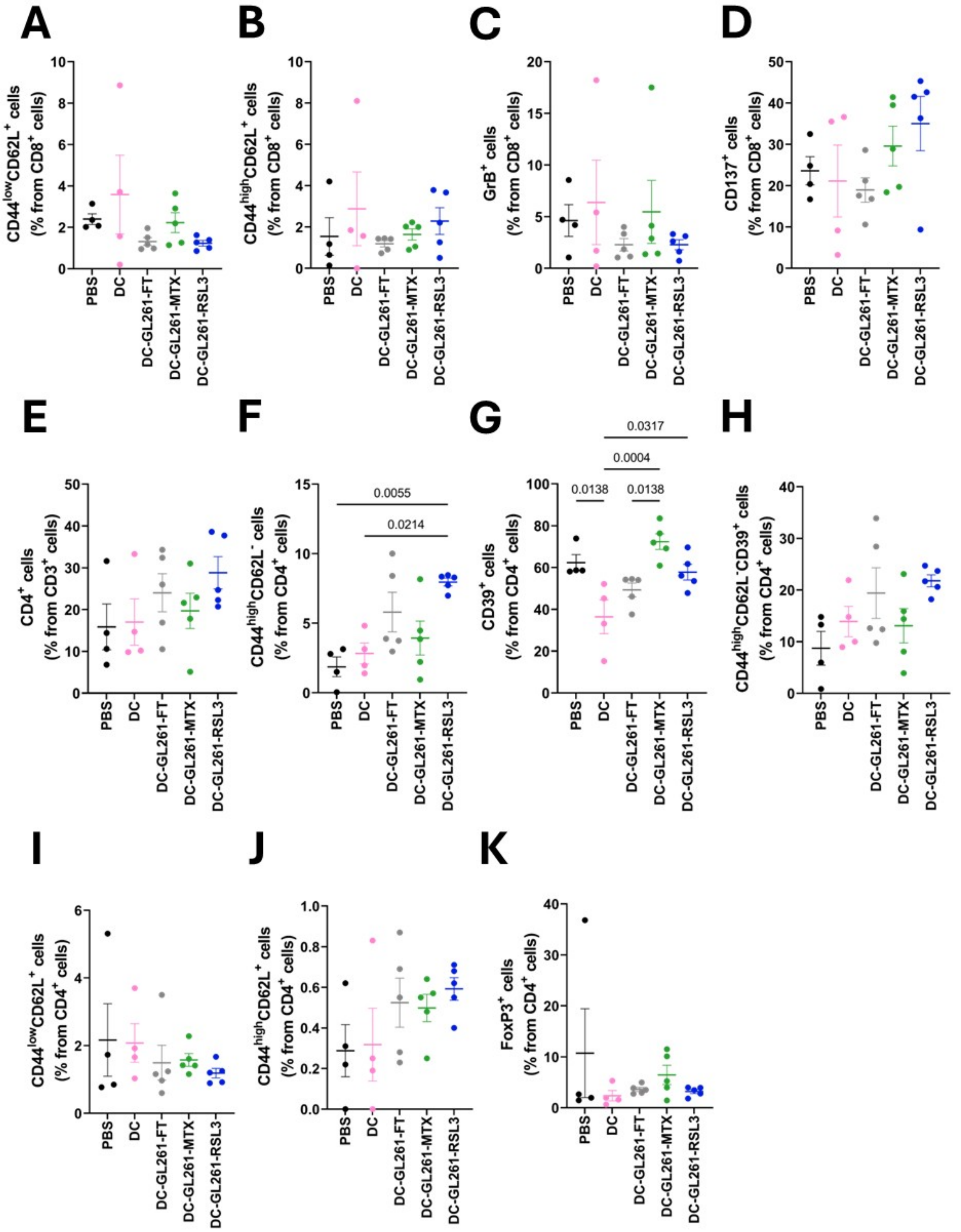

Suppl. Figure 5. **(A-D)** Percentage of $CD8^+$ T cell infiltration. The ferroptosis-armed DC vaccine did not alter the infiltration of naïve ($CD44^{low}CD62L^+$) **(A)**, central memory ($CD44^{high}CD62L^+$) **(B)**, activated (GrB^+) **(C)**, or tumor-specific ($CD137^+$) **(D)** $CD8^+$ T cells.

(E-H) Percentage of $CD4^+$ T cell infiltration **(E)**, effector memory $CD4^+$ T cells ($CD44^{low}CD62L^-$) **(F)**, $CD39^+$ $CD4^+$ T cells **(G)**, and $CD39^+$ effector memory $CD4^+$ T cells **(H)**.

(I–K) The ferroptosis-armed DC vaccine did not alter the infiltration of naïve ($CD44^{low}CD62L^+$) **(I)**, central memory ($CD44^{high}CD62L^+$) **(J)**, or regulatory ($FoxP3^+$) **(K)** $CD4^+$ T cells. Statistical significance was determined using one-way ANOVA followed by Dunnett's multiple comparisons test.

Reviewer #4

Comment 1. *"I co-reviewed this manuscript with one of the reviewers who provided the listed reports. This is part of the Nature Communications initiative to facilitate training in peer review and to provide appropriate recognition for Early Career Researchers who co-review manuscripts."*

Answer 1. We thank the reviewer for his/her contribution to the review process and for supporting the Nature Communications initiative to involve and recognize Early Career Researchers in peer review process.

Reviewer #5 (Remarks to the Author):

"This is an interesting manuscript that could have implications for dendritic cell vaccine design. The authors are subjecting the experimental glioma cell lines, GL261 and CT-2A to various death inducing agents. This results in these gliomas undergoing cell death by apoptosis, necroptosis, and ferroptosis. The resulting apoptotic gliomas are then co-cultured with dendritic cells (DCs) before transfer as DC based vaccines. It is determined that ferroptosis DC vaccination resulted in enhanced survival prophylactically and when administered immediately after tumor inoculation. Additional strategies to demonstrate the independence from LPS adjuvant but reliance on calreticulin, ATP, and potentially HMGB1 DAMPs is put forward. These are the strengths of the work."

We thank Reviewer #5 for the positive and encouraging feedback. We appreciate the recognition of the strengths of our work and the potential implications for DC vaccine design.

Comment 1. *The underpowered study in Figure 2C which does not determine if DC vaccine with RSL3 (ferroptosis) is advantageous over DC vaccine with MTX (immune cell death). This is important to know, as this is of importance for future DC vaccine based design studies.*

Answer 1. We thank the reviewer for this important comment. To address this, we expanded the number of mice per group in **Fig. 2C,D** and **Fig. 2F,G**. The updated analysis confirms that DC vaccines loaded with ferroptotic lysates are not statistically different from those loaded with MTX-treated lysates. However, ferroptotic-based DC vaccines significantly outperform controls, including DCs loaded with FT lysates, unloaded DCs, and PBS.

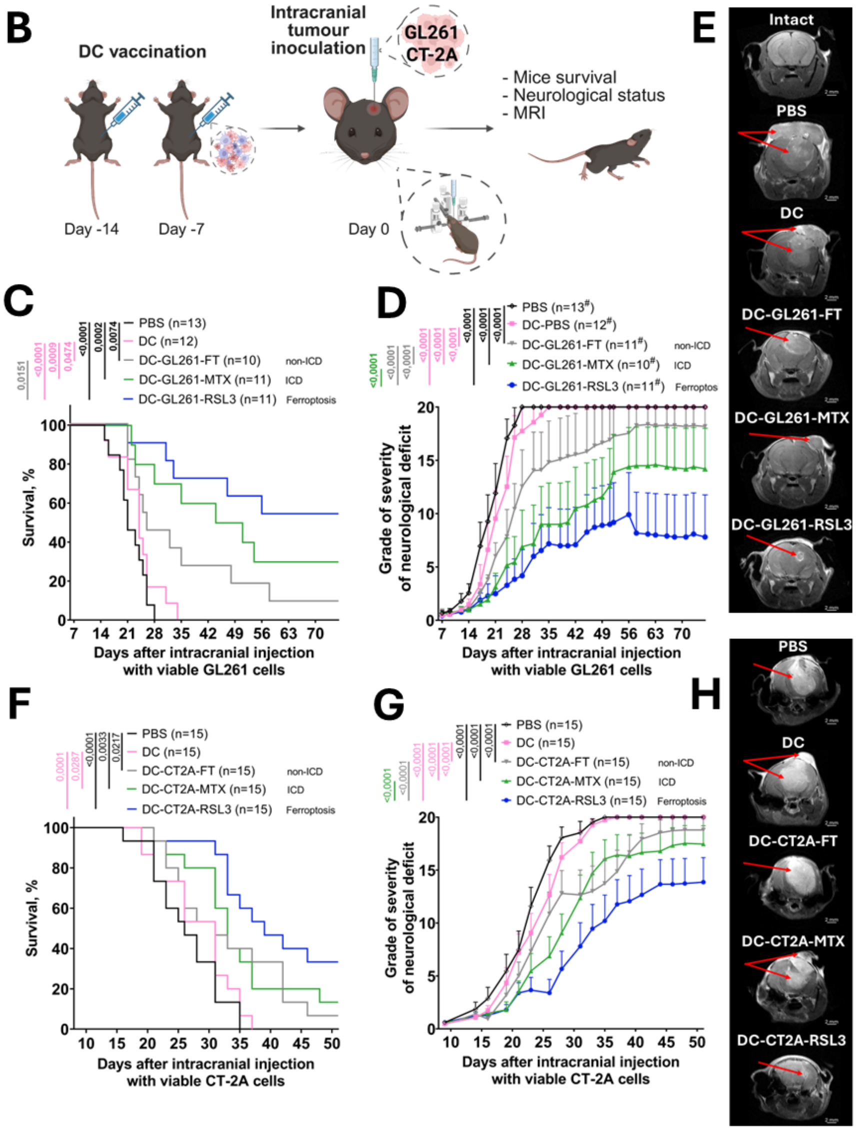

Figure 2. **(B)** Overview of the experimental setup to assess the effectiveness of DC vaccines in a prophylactic context using an orthotopic intracranial mouse model. Mice were administered $1*10^6$ DC vaccines' cells on days -14 and -7, followed by challenge with an intracranial injection of viable glioma GL261 ($2*10^4$ cells) or CT-2A ($5*10^5$ cells) cells on day 0. Survival outcomes, neurological status and MRI scans were monitored thereafter.

Prophylactic vaccination with DC-GL261-RSL3

Mouse survival **(C),** neurological status **(D)** and MRI **(E)** were assessed after injection of the ferroptosis-armed DC vaccine, followed by intracranial challenge with viable glioma GL261 cells. Mouse survival and neurological status were assessed for up to 76 days. Control groups included mice injected with DC vaccines loaded with glioma GL261 lysate subjected to F/T cycles (negative control, non-ICD, DC-GL261-FT) or treated with MTX (2.5 μM, positive control-ICD, DC-GL261-MTX), and mice injected with unloaded DCs (negative control, DCs) or with PBS (negative control). #On day 23, three mice per group underwent MRI scans. In some of these mice (2 in the PBS group and 2 in the DC group), the glioma size had already reached humane endpoints, and they were euthanized following the MRI. These mice were included in the analysis of survival and neurological status. However, other mice (1 in the PBS and DC groups and 3 in each of the DC-GL261-FT, DC-GL261-MTX, and DC-GL261-RSL3 groups) did not reach humane endpoints and were euthanized after the MRI; these mice were excluded from the analysis. **(E)** Representative T1-weighted MRI images of coronal brain sections taken on day 23, n=3 mice per group. Arrows point to tumors. Statistical analysis for mice survival was performed using Mantel-Cox logarithmic test. Statistical analysis of the neurological status of the mice was performed using a two-way ANOVA followed by Tukey's multiple comparisons correction.

Prophylactic vaccination with DC-CT2A-RSL3

Mouse survival **(F)**, neurological status **(G),** and MRI **(H)** were evaluated after injection of the ferroptosis-armed DC vaccines and intracranial challenge with viable glioma CT2A cells. Mouse survival and neurological status were monitored for up to 51 days. Control groups included mice injected with DC vaccines loaded with glioma CT-2A lysates subjected to F/T cycles (negative control, non-ICD, DC-CT2A-FT) or treated with MTX (2.5 μM, positive control-ICD, DC-CT2A-MTX), and mice injected with unloaded DCs (negative control, DCs) or PBS (negative control). **(H)** Representative T1-weighted MRI images of coronal brain sections taken on day 23, with the tumor indicated by arrows, n=4 mice per group. Statistical analysis for mice survival was determined by Mantel-Cox logarithmic test. Statistical analysis of the neurological status of the mice was performed using a two-way ANOVA followed by Tukey's multiple comparisons correction.

Comment 2. *In Figure 2E and 2H, the MRIs shown have animals in which the gliomas have grown outside the skull. It is unclear if the glioma presents this way there would be an effect on immunotherapy efficacy.*

Answer 2. We thank the reviewer for this observation. Disruption of the blood-brain barrier (BBB) is a well-documented feature of glioblastoma, both in patients and in the GL261 model (Leten et al., 2014; Pichol-Thievend et al., 2024; Wolburg et al., 2012). In our study, extracranial tumor extension was observed at late disease stages across all groups. However, only mice vaccinated with ferroptotic or apoptotic lysate-loaded DCs showed improved tumor control, suggesting that the efficacy of these vaccines is not diminished by BBB disruption or extracranial tumor growth.

Comment 3. *Figure 3A - The administration of immunotherapy at Day 2 post glioma inoculation is VERY early. This is well before there is an MRI detectable glioma in these animals.*

Answer 3. We have addressed this point by performing an additional experiment with delayed vaccination (**Fig. 4A–C**). Specifically, DC vaccination was started 7 days after intracranial inoculation of glioma GL261 cells, at a time point when tumors provably have been established (McKelvey et al., 2020). Mice treated with DC vaccines loaded with ferroptotic lysates (DC-GL261-RSL3) or with immunogenic apoptotic lysates (DC-GL261-MTX, positive control: ICD) exhibited significantly improved survival compared to negative controls (PBS, DC alone, or DC-GL261-FT: non-ICD). These data have been included in the manuscript on page 6.

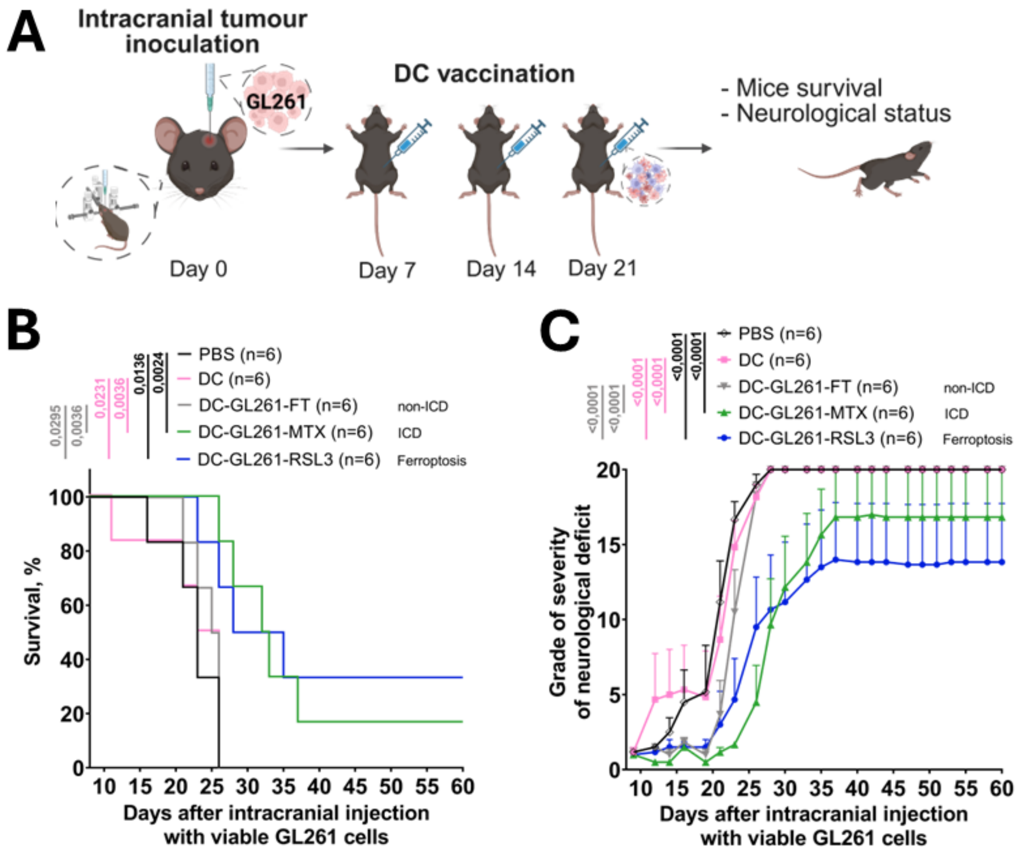

Figure 4. **(A)** Procedure to assess the effecacy of DC vaccines in a more clinically relevant therapeutic setting using an orthotopic intracranial mouse model. Mice were injected intracranially with viable glioma GL261 cells on day 0, followed by administration of the corresponding DC vaccine on days 7, 16 and 25. Control groups included mice injected with DC vaccines loaded with glioma GL261 lysate subjected to F/T cycles (negative control: non-ICD, DC-GL261-FT), DC vaccines loaded with MTX-treated lysates (2.5 µM, positive control: ICD, DC-GL261-MTX), unloaded DCs (negative control, DCs) or PBS (negative control). Mouse survival **(B)** and neurological status **(C)** were assessed following intracranial injection with viable glioma GL261 cells and subsequent therapy with ferroptosis-armed DC vaccines. Survival and neurological status were monitored for up to 60 days. Mouse survival was analyzed using the Mantel–Cox log-rank test. Statistical analysis of the neurological status of the mice was performed using a two-way ANOVA followed by Tukey's multiple comparisons correction.

Comment 4. *The studies in Figure 3 end at day 40. This is an early endpoint, as tumor recurrence is common in these models. Are these animals cured long term?*

Answer 4. We thank the reviewer for this important point. In our survival studies, experiments were typically ended once all control mice had succumbed, ensuring that surviving mice remained free of neurological symptoms for at least one additional week.

To further assess long-term immunity, we conducted a tumor rechallenge experiment (**Fig. 5F–H**), where surviving mice were re-inoculated with GL261 cells and monitored for survival, neurological status, and tumor burden via MRI. This experiment extended to 116 days and demonstrated that DC vaccines loaded with ferroptotic lysates induce robust immunological memory, providing effective protection against tumor recurrence.

Comment 5. *In Figure 3F and G, there is considerable variability in the ELISpot analysis.*
It would make sense to track SIINFEKL antigen specific CD8 T cells with peptide:MHC tetramers in the lymph nodes. Additional analysis with increased brain infiltration of CD8 T cells targeting this model antigen would be informative too.

Answer 5. We thank the reviewer for this suggestion. To address this, we performed a new ELISpot assay (**Fig. 4D–F**) using DC vaccines loaded with GL261-OVA cells subjected to FT (non-ICD, negative control), MTX (ICD, positive control), and RSL3 (ferroptosis), alongside unloaded DCs

and PBS controls. A significant increase in T-cell responses was observed in the RSL3 and MTX groups, while the FT, unloaded DC, and PBS groups showed minimal responses.
This experiment clarified the differences between DC vaccines loaded with ferroptotic lysates and non-ICD groups, highlighting the immunogenicity of ferroptotic lysates.

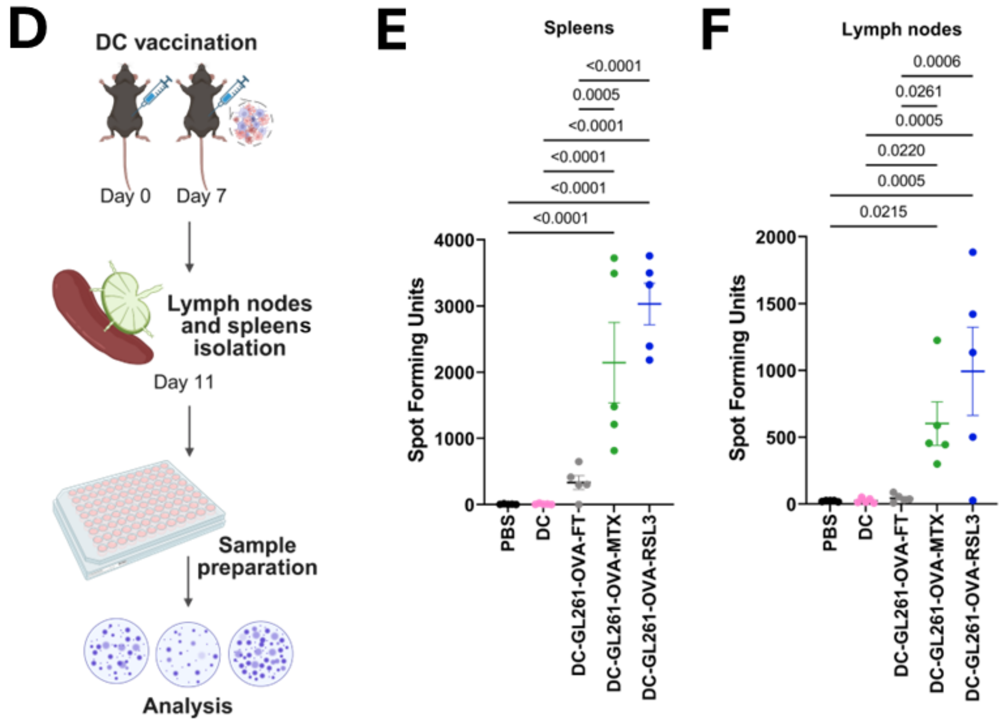

Figure 4. **(D)** Procedure to analyze the priming of cytotoxic T cells and IFN-γ production in mice immunized with DC vaccines on days 0 and 7. Four days after the final vaccination, the inguinal lymph nodes and splenocytes were isolated and re-stimulated *ex vivo* with the SIINFEKL peptide. **(E, F)** IFN-γ ELISpot assay of the spleens **(E)** and draining lymph nodes **(F)** from immunized mice. DC vaccines were loaded with ferroptotic GL261 lysates (DC-GL261-OVA-RSL3). Ferroptosis was induced by 2.5 μM RSL3. As controls, we used DC vaccines loaded with glioma GL261 cells subjected to F/T cycles (negative control: non-ICD, DC-GL261-OVA-FT), with MTX (2.5 μM, positive control: ICD, DC-GL261-OVA-MTX), unloaded DCs (negative control, DCs) or PBS (negative control). Each type of lysate was supplemented with 1 μM ovalbumin. The number of spot-forming units is presented as means ± SEM from data obtained from 5 mice. Statistical significance was determined using one-way ANOVA followed by Dunnett's multiple comparisons test.

Comment 6. *"In Figure 4 , the analysis of LPS is informative negative data. However, it appears that Figure 5F is lacking wild type mouse derived DC controls in addition to the TLR4 KO mouse DC groups.*
Figure 4G needs a control for glioma induced mortality."

Answer 6. We thank the reviewer for this observation. To address this, we conducted an additional experiment analyzing the effect of LPS on the efficacy of ferroptosis-based DC vaccines in wild-type mice (**Suppl. Fig. 3E–G**). The results confirmed that LPS did not enhance vaccine efficacy, as both wild-type and TLR4 knockout mice (**Fig. 5F-H**) showed similar levels of protection with ferroptotic DC vaccines, regardless of LPS inclusion. We have adapted the Materials and Methods and Results sections accordingly.

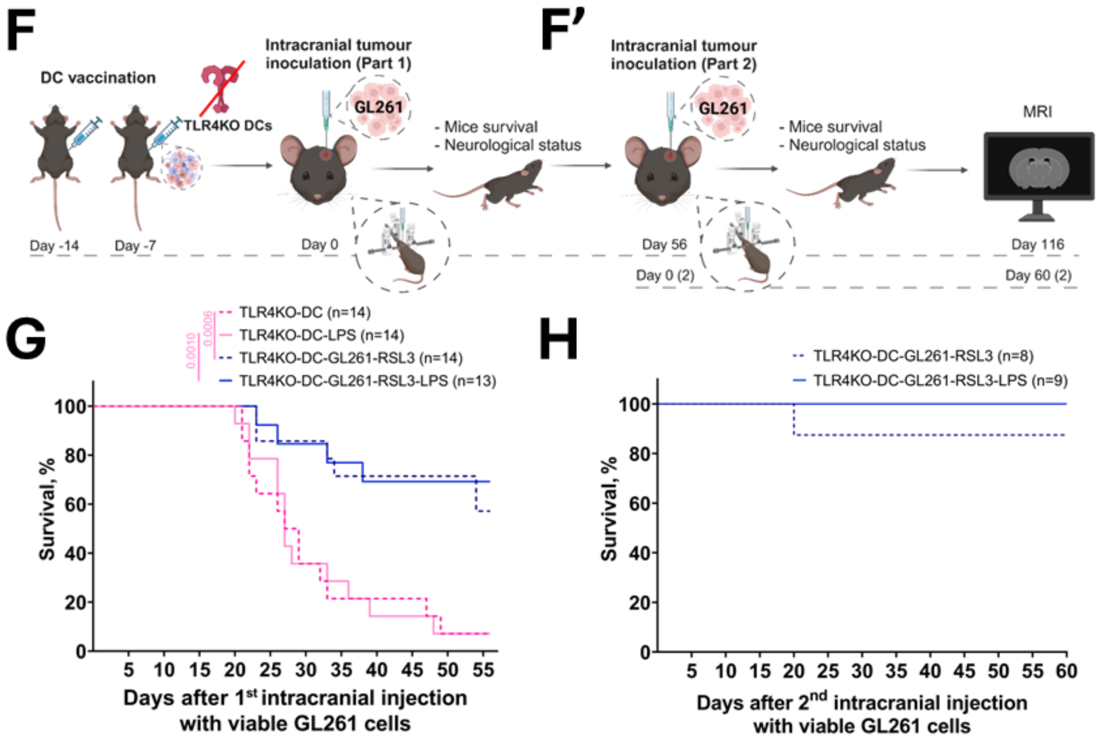

Figure 5. Part 1:

(F) Procedure for studying the effectiveness of TLR4KO-DC vaccines generated in the presence or absence of LPS (as explained in **E**) in wild type C57BL/6J mice (*i.e.,* **part 1**).

Wild type mice received ferroptosis-armed TLR4KO-DC vaccines (TLR4KO-DC-GL261-RSL3 or TLR4KO-DC-GL261-RSL3-LPS) on days -14 and -7, followed by intracranial injection of viable glioma GL261 cells on day 0 **(part 1)**. In part 1, mouse survival and neurological status were monitored up to day 56.

In part 1 **(F)**, mouse survival after injection of ferroptosis-armed TLR4KO DC vaccines (2.5 μM, 24 h, TLR4KO-DC-GL261-RSL3) followed by intracranial challenged with viable GL261 cells. Mouse survival was monitored for up to 56 days **(part 1)**. As controls, we used unloaded TLR4KO DCs, which were either treated with LPS (TLR4KO-DC-LPS) or untreated (TLR4KO-DC). Statistical analysis was performed using the Mantel-Cox logarithmic test.

Part 2:

(F') Procedure **(part 2)** for assessing the development of immunological memory in immunocompetent C57BL/6J mice that had rejected viable GL261 cells due to therapy with ferroptosis-armed TLR4KO-DCs in **(F, part 1)**. **In part 2**, the surviving mice were re-challenged on day 0 of the second intracranial glioma injection (56 days after the first challenge) with a GL261 glioma cell dose that normally induces glioma growth in naive mice (*e.g.,* see **Fig. 2C, F**; the negative control group injected with PBS). Mouse survival and neurological status were monitored for up to 116 days after the first injection (*i.e.,* 60 after the second injection). The MRI scans were performed on day 116.

(G: part 1; H: part 2) Mouse survival after therapy with ferroptosis-armed TLR4KO-DC vaccines (TLR4KO-DC-GL261-RSL3) and subsequent intracranial rechallenge with viable GL261 cells. Mouse survival was monitored for up to 60 days after the rechallenge. As controls, we used unloaded TLR4KO-DC treated with LPS (TLR4KO-DC-LPS) or left untreated (TLR4KO-DC). Results were analyzed with Mantel-Cox logarithmic rank test.

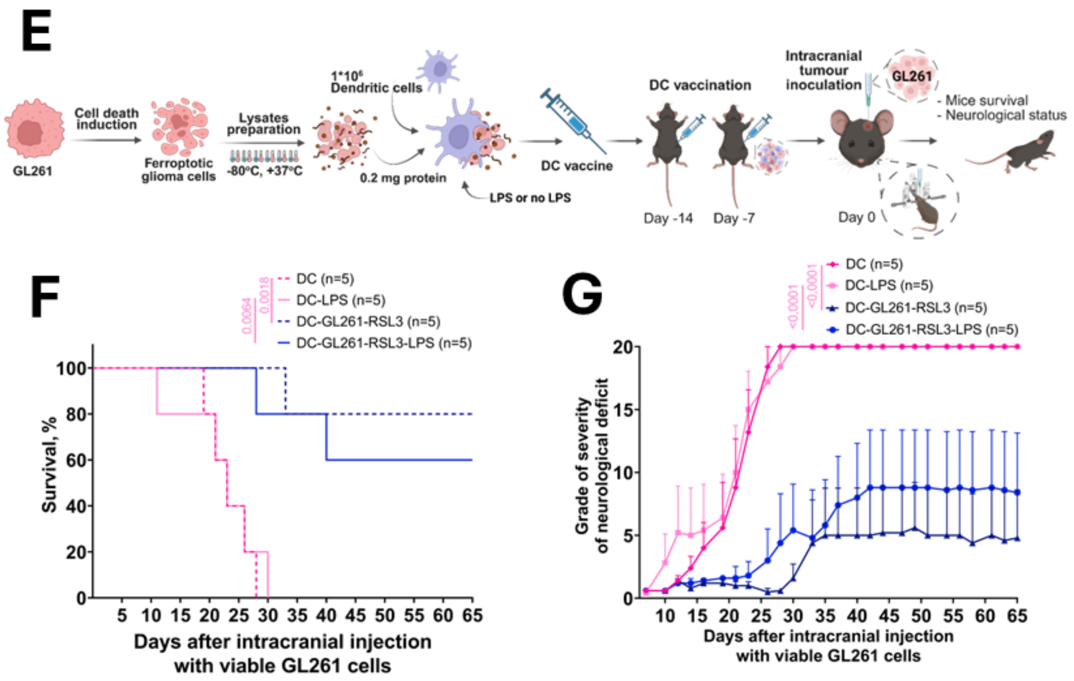

Suppl. Figure 3. **(E)** Procedure for generating ferroptosis-armed DC vaccines from wild-type (WT) mouse bone marrow-derived DCs (WT-DCs). WT-DCs were co-cultured for 90 minutes with ferroptotic GL261 lysates (RSL3, 2.5 μM, 24 h), and then further incubated with the same lysates in the presence (DC-GL261-RSL3-LPS) or absence (DC-GL261-RSL3) of 0.5 μg/mL LPS for 24 hours. WT mice received the respective DC vaccines on days –14 and –7, followed by intracranial injection of viable GL261 glioma cells on day 0. As controls, mice were treated with unloaded DCs, either LPS-stimulated (DC-LPS) or unstimulated (DC).
(F, G) Mouse survival **(F)** and neurological status **(G)** were monitored up to day 65. Survival data were analyzed using the Mantel-Cox log-rank test. Neurological status was analyzed using two-way ANOVA followed by Tukey's multiple comparisons test.

Comment 7. *"The data in Figure 5F is interpreted as HMGB1 not being required for ferroptosis DC vaccine efficacy. However, the survival curve implies this conclusion is based on an underpowered experiment."*

Answer 7. We appreciate the reviewer's concern regarding sample size. To address this, we based our sample size calculation on a previously published DC vaccination study in an orthotopic brain tumor model (Garg et al., 2016). Using G*Power 3.1.9 software (α = 0.05, power = 0.8), the analysis indicated that a minimum of 6 mice per group was required. In the HMGB1 blocking experiment, we included 7 mice, ensuring the study was appropriately powered.
The sample size was calculated with G*Power 3.1.9 software:

Analysis:	A priori: Compute required sample size		
Input:	Effect size f	=	5
	α err prob	=	0,05
	Power (1-β err prob)	=	0,8
	Numerator df	=	10
	Number of groups	=	3
Output:	Noncentrality parameter λ	=	150
	Critical F	=	8.7855247
	Denominator df	=	3
	**Total sample size**	**=**	**6**
	Actual power	=	0.8502608

Additionally, we performed a complementary experiment using TLR4-knockout DCs, as TLR4 is a primary receptor for HMGB1. Consistent with the HMGB1-blocking results, TLR4-deficient DCs retained full vaccine efficacy, showing no reduction in survival compared to wild-type DCs (**Fig.**

5G; Suppl. Fig. 9A). Both approaches independently indicate that HMGB1 signaling is not critical for the anti-tumor effect of ferroptosis-based DC vaccines in this model.

Comment 8. *"The graphical abstract in Figure 6 is more confusing than useful. The concept of ferroptosis DC vaccine is more clearly written than illustrated."*

Answer 8. We thank the reviewer for this feedback. The graphical abstract has been redesigned to improve clarity and better illustrate the concept of the ferroptosis-based DC vaccine.

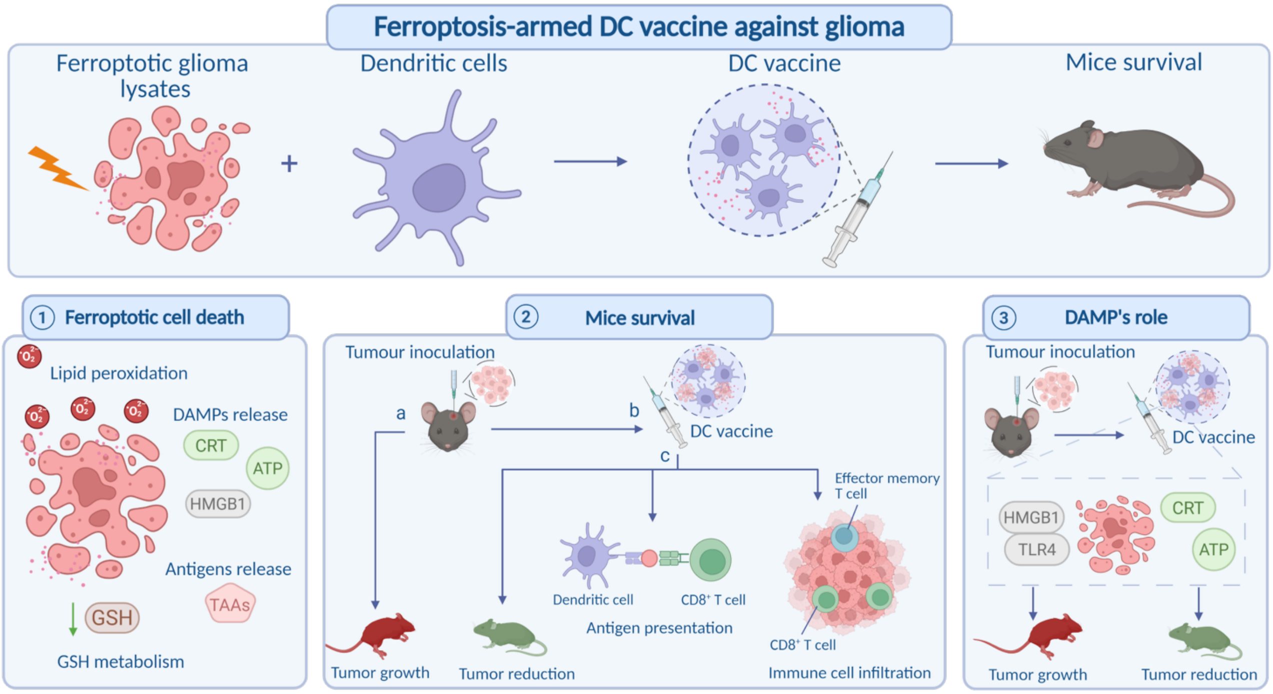

Graphical abstract. Dendritic cell (DC) vaccines primed with ferroptotic glioma lysates effectively protected mice across multiple orthotopic glioma models **(a, b)**. The immunogenicity of these ferroptotic lysates remained consistent, regardless of the glioma cell lines used, the specific ferroptosis inducers applied, or the inclusion of bacterial adjuvants like LPS. This highlights the strong intrinsic immunogenic potential of ferroptosis. DC vaccines loaded with ferroptotic lysates protect mice against glioma growth, inducing IFN-γ production, and promoting robust $CD8^+$ T cell infiltration, activation, and effector memory formation in the tumor microenvironment **(c)**. Blocking key danger signals such as calreticulin (CRT) and ATP significantly reduced the immunogenicity of ferroptotic glioma lysates and the protective effect of DC vaccines in the orthotopic glioma model. In contrast, inhibition of the high mobility group box 1 (HMGB1)–Toll-like receptor 4 (TLR4) axis did not affect their immunogenicity. Our findings highlight the therapeutic potential of utilizing ferroptotic tumor lysates derived from patient malignant biopsies as key components in personalized DC-based immunotherapies.

Reviewer #6

"In this manuscript by Savuiuk et al, the authors demonstrate that ferroptotic cell death generates an immunogenic cell lysate, potential through the generation and release of damage associated molecular patterns (DAMPs) that have the ability to mediate dendritic cell maturation on their own and without the addition of other exogenous inflammatory mediators. This is the principal finding of this work. Using ferroptotic cell lysates, the authors then attempt to demonstrate the potential utility of these in the generation of dendritic cell vaccines for the treatment of glioma. As detailed below, this line of experimental inquiry is problematic due to experimental design and technical concerns. Issues with experimental rigor and small sample sizes also lead the authors to draw conclusions that are statistically correct but otherwise likely untrue, i.e. almost certainly type II errors."

We thank the reviewer for the careful evaluation of our manuscript and for highlighting the principal findings of our work. We appreciate the constructive criticism regarding the experimental design, rigor, and statistical interpretation. These comments have been valuable in guiding our revisions and improving the overall quality and clarity of the study.

Comment 1. *" The data depicted in figure 1E is one example of small sample size and experimental variance leading to a likely type II error. Authors conclude that by six hours posttreatment of GL261 with the ferroptosis inducer RSL3, lipid peroxidation levels have normalized and are no different from control, this despite two of three samples showing lipid peroxidation levels four-fold higher than the control. Additional experimentation and larger sample size would likely establish that one of the three samples was a statistical outlier or experimental error and permit the authors to reach the opposite conclusion."*

Answer 1. We appreciate the reviewer's thoughtful comment. In response, we performed additional experiments and increased the sample size. The data were re-analyzed, and statistical evaluation has been updated accordingly (**Fig. 1E–H**), providing a more robust assessment of lipid peroxidation dynamics.

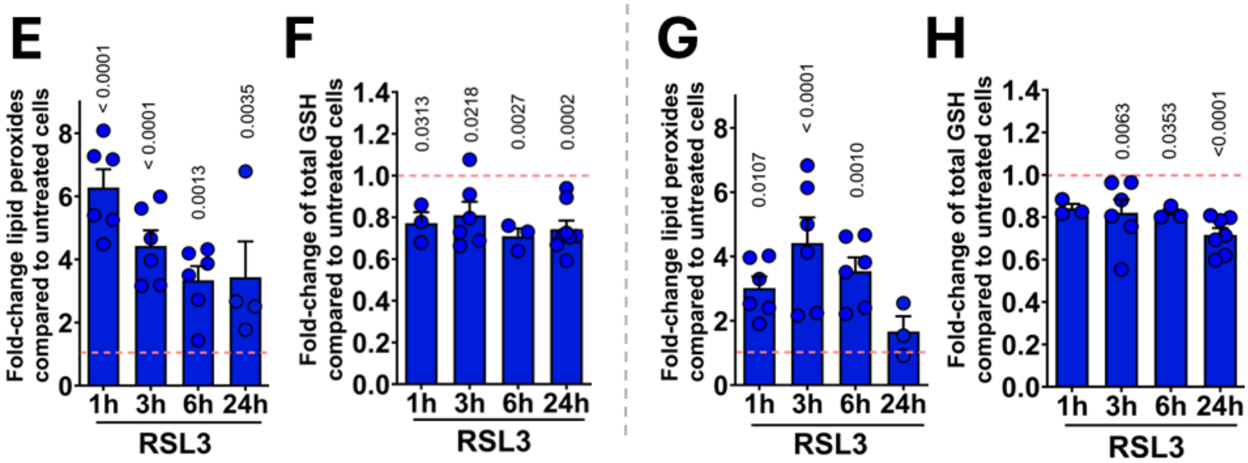

Figure 1. (E, G) Generation of lipid ROS (lipid peroxides) during ferroptosis was assessed after treating glioma cells with RSL3 (2.5 μM) for 1, 3, 6 or 24 hours and staining with BODIPY C11. Data are shown as fold change relative to untreated cells. RSL3 treatment of GL261 and CT-2A cells led to a significant increase in lipid peroxidation levels during the early stages of ferroptosis compared to untreated cells shown as dotted line. **(F, H)** Glutathione (GSH) levels were analyzed in lysates of GL261 and CT-2A cells treated for 24 hours RSL3 (2.5 μM) for 1, 3, 6 or 24 hours. RSL3 significantly reduced GSH levels in both glioma cell lines. Data are shown as fold change relative to untreated cells shown as dotted line. The values are expressed as means ± SEM, derived from 3 to 7 independent experiments. Statistical significance was determined using a one-way ANOVA followed by Dunnett multiple comparisons test or Kruskal-Wallis test with Dunn's multiple comparisons correction.

Comment 2. *"It is unclear why the authors have chosen to vaccinate mice intraperitoneally in their glioma vaccination models. Clearly some response is being targeted to the brain through this route of delivery; however, the route is exceptionally suboptimal for T-cell homing to the CNS."*

Answer 2. We acknowledge the reviewer's point regarding optimal routes for DC vaccination. While s.c. or i.d. injection is often preferred for efficient lymph node targeting, multiple studies,

including our previous work (Maes et al., 2009; Vandenberk et al., 2016; Vedunova et al., 2022), have demonstrated that intraperitoneal (i.p.) administration of DC vaccines can elicit strong systemic T-cell responses in murine glioma models. In our current study, i.p. vaccination effectively induced systemic immune activation, enhanced T-cell infiltration into the glioma microenvironment, and improved survival. These findings suggest that, despite its limitations, the i.p. route remains a feasible and effective method for inducing therapeutic anti-tumor immunity in this model.

Comment 3. *"Instead of (or in addition to) MRI imaging, characterization of in situ immune responses should be performed. At a minimum, authors should do H&E staining to identify lymphocytic infiltrates and IHC analysis for CD8+ T-cell presence although fancier (and more expensive) techniques such as spatial transcriptomics or single cell analysis would also be appropriate. Regardless, H&E/IHC are sufficient to establish that the author's vaccine approach is exerting its effects through an adaptive T-cell response."*

Answer 3. We thank the reviewer for this valuable suggestion. In line with the comment, we performed detailed immunoprofiling of the tumor microenvironment, focusing on T-cell infiltration (**Fig. 6; Suppl. Fig. 5**). Specifically, we observed a significant increase in CD8$^+$ T-cell infiltration in mice treated with the ferroptosis-based DC vaccine (**Fig. 6B**), supporting the involvement of adaptive T-cell responses in mediating the anti-tumor effect.

These findings have been highlighted in the *Results* section (page 8). While we have not yet performed in situ analyses such as H&E or IHC staining, we agree that spatial characterization of immune responses would be a valuable extension for future studies.

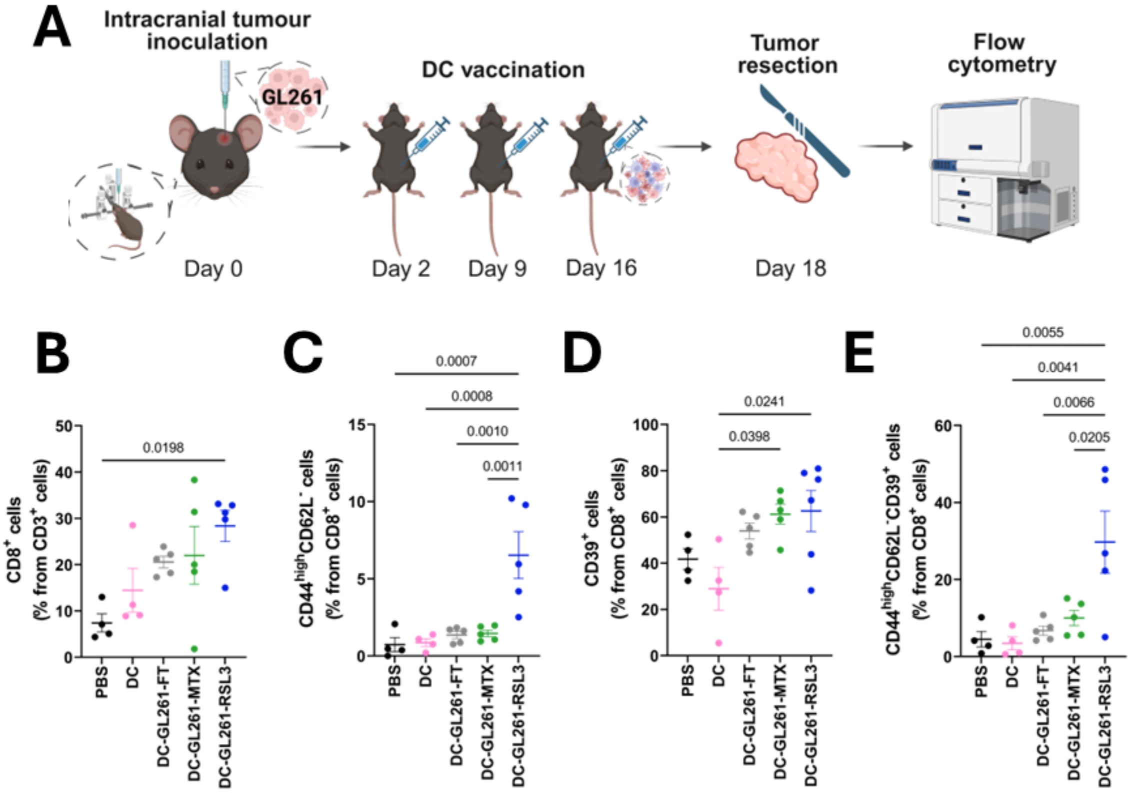

Figure 6. (A) Procedure to assess T-cell infiltration induced by DC vaccines in a therapeutic setting using an orthotopic intracranial mouse model. Mice were injected intracranially with viable glioma GL261 cells on day 0. Subsequently, they received the corresponding DC vaccine on days 2, 9 and 16. On day 18 after tumor inoculation, intracranial tumors were isolated, and immune cells were analyzed by multicolor flow cytometry. Control groups included mice treated with DC vaccines loaded with glioma GL261 lysate subjected to F/T cycles (negative control: non-ICD, DC-GL261-FT), DC vaccines loaded with MTX-treated lysates (2.5 μM, positive control: ICD, DC-GL261-MTX), unloaded DCs (negative control, DCs) or PBS (negative control). **(B-E)** Percentage of CD8$^+$ T cell infiltration. The ferroptosis-armed DC vaccine increased the infiltration of total CD8$^+$ T cells **(B)**, effector memory CD8$^+$ T cells (CD44$^+$CD62L$^-$ **C)**, CD39$^+$ exhausted CD8$^+$ T cells **(D)** and CD39$^+$ effector memory CD8$^+$ T cells **(E)**. Statistical significance was determined using one-way ANOVA followed by Dunnett multiple comparisons test.

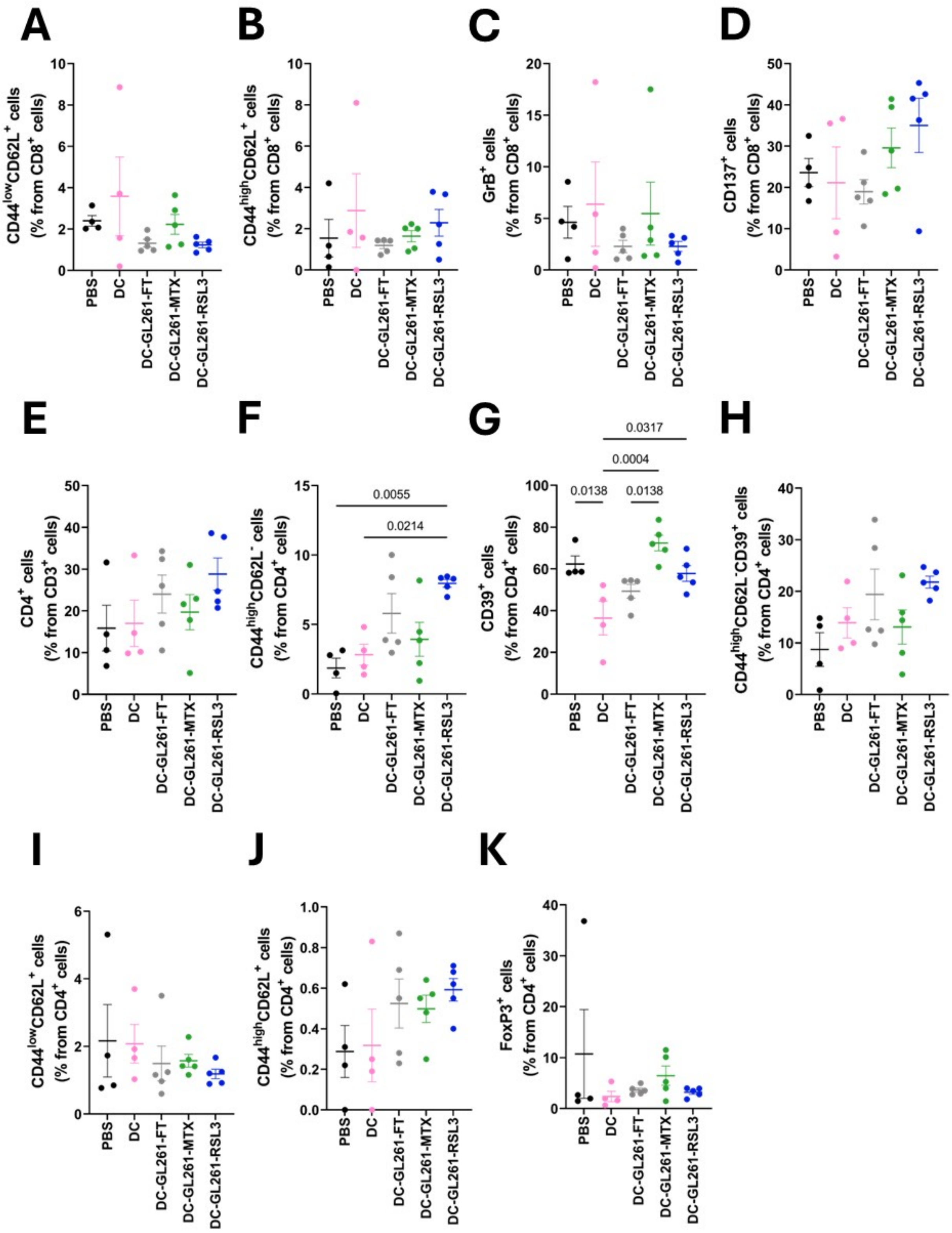

Suppl. Figure 5. (A-D) Percentage of $CD8^+$ T cell infiltration. The ferroptosis-armed DC vaccine did not alter the infiltration of naïve ($CD44^{low}CD62L^+$) **(A)**, central memory ($CD44^{high}CD62L^+$) **(B)**, activated (GrB^+) **(C)**, or tumor-specific ($CD137^+$) **(D)** $CD8^+$ T cells.

(E-H) Percentage of $CD4^+$ T cell infiltration **(E)**, effector memory $CD4^+$ T cells ($CD44^{low}CD62L^-$) **(F)**, $CD39^+$ $CD4^+$ T cells **(G)**, and $CD39^+$ effector memory $CD4^+$ T cells **(H)**.

(I–K) The ferroptosis-armed DC vaccine did not alter the infiltration of naïve ($CD44^{low}CD62L^+$) **(I),** central memory ($CD44^{high}CD62L^+$) **(J)**, or regulatory ($FoxP3^+$) **(K)** $CD4^+$ T cells. Statistical significance was determined using one-way ANOVA followed by Dunnett's multiple comparisons test.

__Comment 4.__ "How do dendritic cells loaded/matured with ferroptotic lysates compare to DC loaded with F/T lysates and matured with inflammatory cues known to induce TH1 polarizing immune responses? This is the actual question that must be answered and the actual comparisons that must be performed. The comparators in these experiments would be DC loaded with F/T glioma lysates and matured with IL-1b/TNF/IL-6 supplemented with intracellular TLR ligands like poly(I:C) (TLR3), resiquimod (TLR7/8), or CpG (TLR9) or STING agonists or RIG-I/MDA5 RLH-type agonists or type I interferons as these are all known to induce powerful TH1 polarizing responses.
Experiments should compare differences in the CD4/CD8 T-cell ratio of expanding T-cell populations as well as interferon gamma/perforin/granzyme expression levels of the expanding CD8+ cells. Are the T-cells expanded by such DC more or less likely to form durable memory populations? Are the T-cells expanded by such DC more or less likely to become exhausted?"

Answer 4. We thank the reviewer for this insightful comment.

Our findings indicate that ferroptotic lysates alone are sufficient to generate efficient DCs vaccine with strong anti-tumor properties (**Fig. 5F-H**). To further enhance maturation, we also tested the addition of LPS, a classical TLR4 agonist widely used in preclinical DC vaccine mice models (T. H. Han et al., 2009; Sabado et al., 2017).

However, *in vivo* experiments comparing DC vaccines with and without LPS (using both WT and TLR4 KO DCs revealed no added benefit in terms of anti-tumor efficacy (**Fig. 5E-H, Suppl. Fig. 3E-G**) to investigate the additive role of LPS and we didn't find any difference.

While we agree that future studies incorporating PRR agonists (*e.g.,* TLR3, TLR8/9, STING ligands) would provide valuable mechanistic insights and optimize DC maturation towards TH1 polarization, our primary aim in this study was to investigate the intrinsic immunogenicity of ferroptotic tumour cells (*i.e.,* lysates). Translating strategies involving combinatorial adjuvants and detailed T-cell profiling are important next steps but fall outside the scope of this initial mechanistic study.

Importantly, we assessed IFN-γ production by lymph node and splenic cells using ELISpot (**Fig. 4D–F**) and observed robust T-cell responses following vaccination with ferroptotic lysate-loaded DCs, further supporting their immunostimulatory potential.

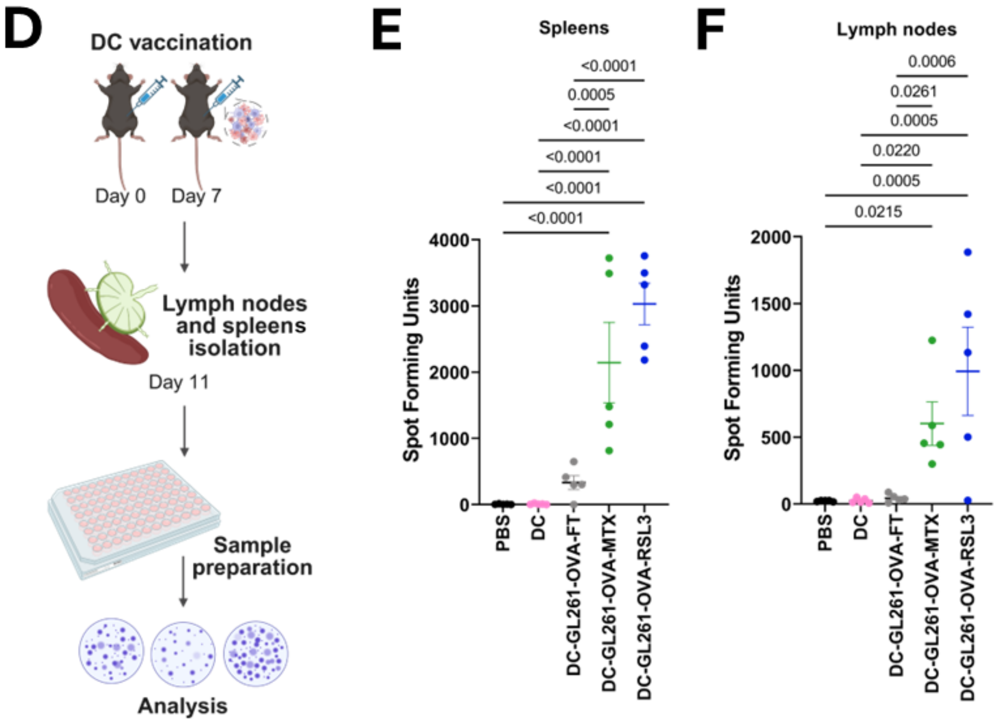

Figure 4. **(D)** Procedure to analyze the priming of cytotoxic T cells and IFN-γ production in mice immunized with DC vaccines on days 0 and 7. Four days after the final vaccination, the inguinal lymph nodes and splenocytes were isolated and re-

stimulated *ex vivo* with the SIINFEKL peptide. **(E, F)** IFN-γ ELISpot assay of the spleens **(E)** and draining lymph nodes **(F)** from immunized mice. DC vaccines were loaded with ferroptotic GL261 lysates (DC-GL261-OVA-RSL3). Ferroptosis was induced by 2.5 µM RSL3. As controls, we used DC vaccines loaded with glioma GL261 cells subjected to F/T cycles (negative control: non-ICD, DC-GL261-OVA-FT), with MTX (2.5 µM, positive control: ICD, DC-GL261-OVA-MTX), unloaded DCs (negative control, DCs) or PBS (negative control). Each type of lysate was supplemented with 1 µM ovalbumin. The number of spot-forming units is presented as means ± SEM from data obtained from 5 mice. Statistical significance was determined using one-way ANOVA followed by Dunnett's multiple comparisons test.

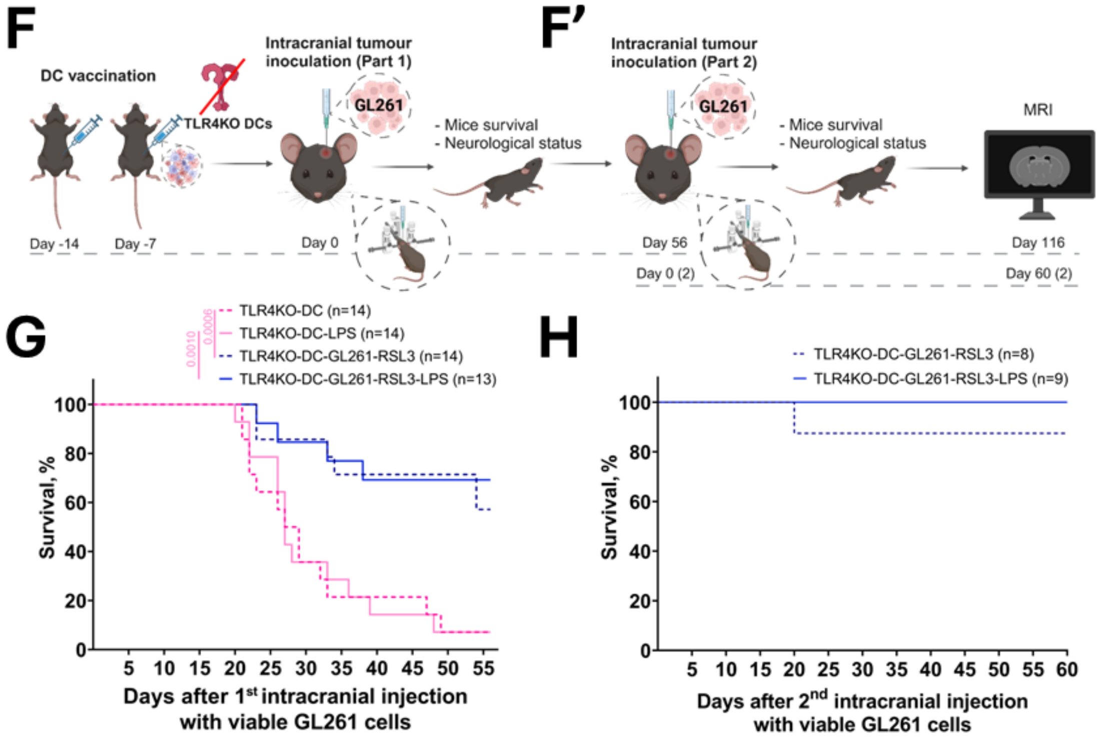

Figure 5. Part 1:

(F) Procedure for studying the effectiveness of TLR4KO-DC vaccines generated in the presence or absence of LPS (as explained in **E**) in wild type C57BL/6J mice (*i.e., **part 1***).

Wild type mice received ferroptosis-armed TLR4KO-DC vaccines (TLR4KO-DC-GL261-RSL3 or TLR4KO-DC-GL261-RSL3-LPS) on days -14 and -7, followed by intracranial injection of viable glioma GL261 cells on day 0 **(part 1)**. In part 1, mouse survival and neurological status were monitored up to day 56.

In part 1 **(F)**, mouse survival after injection of ferroptosis-armed TLR4KO DC vaccines (2.5 µM, 24 h, TLR4KO-DC-GL261-RSL3) followed by intracranial challenged with viable GL261 cells. Mouse survival was monitored for up to 56 days **(part 1)**. As controls, we used unloaded TLR4KO DCs, which were either treated with LPS (TLR4KO-DC-LPS) or untreated (TLR4KO-DC). Statistical analysis was performed using the Mantel-Cox logarithmic test.

Part 2:

(F') Procedure **(part 2)** for assessing the development of immunological memory in immunocompetent C57BL/6J mice that had rejected viable GL261 cells due to therapy with ferroptosis-armed TLR4KO-DCs in **(F, part 1)**. **In part 2**, the surviving mice were re-challenged on day 0 of the second intracranial glioma injection (56 days after the first challenge) with a GL261 glioma cell dose that normally induces glioma growth in naive mice (*e.g.,* see **Fig. 2C, F**; the negative control group injected with PBS). Mouse survival and neurological status were monitored for up to 116 days after the first injection (*i.e.,* 60 after the second injection). The MRI scans were performed on day 116.

(G: part 1; H: part 2) Mouse survival after therapy with ferroptosis-armed TLR4KO-DC vaccines (TLR4KO-DC-GL261-RSL3) and subsequent intracranial rechallenge with viable GL261 cells. Mouse survival was monitored for up to 60 days after the rechallenge. As controls, we used unloaded TLR4KO-DC treated with LPS (TLR4KO-DC-LPS) or left untreated (TLR4KO-DC). Results were analyzed with Mantel-Cox logarithmic rank test.

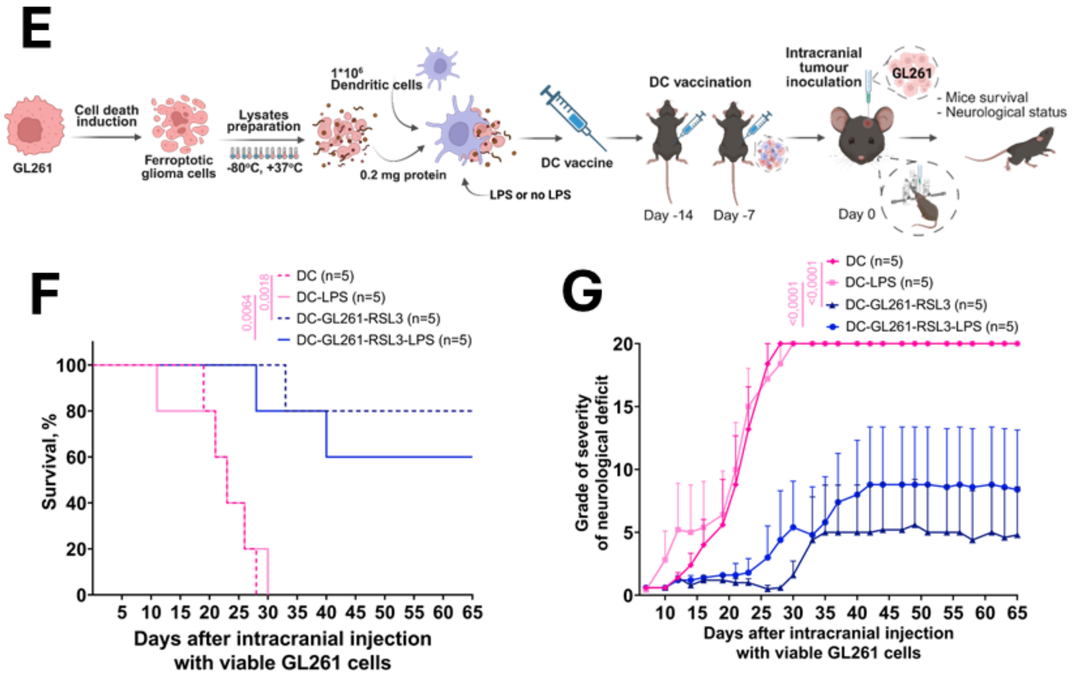

Suppl. Figure 3. **(E)** Procedure for generating ferroptosis-armed DC vaccines from wild-type (WT) mouse bone marrow-derived DCs (WT-DCs). WT-DCs were co-cultured for 90 minutes with ferroptotic GL261 lysates (RSL3, 2.5 μM, 24 h), and then further incubated with the same lysates in the presence (DC-GL261-RSL3-LPS) or absence (DC-GL261-RSL3) of 0.5 μg/mL LPS for 24 hours. WT mice received the respective DC vaccines on days –14 and –7, followed by intracranial injection of viable GL261 glioma cells on day 0. As controls, mice were treated with unloaded DCs, either LPS-stimulated (DC-LPS) or unstimulated (DC).
(F, G) Mouse survival **(F)** and neurological status **(G)** were monitored up to day 65. Survival data were analyzed using the Mantel-Cox log-rank test. Neurological status was analyzed using two-way ANOVA followed by Tukey's multiple comparisons test.

Comment 5. *"In this regard (above criticism), the authors have used the wrong control DC populations in their tumor challenge experiments. Only the DC loaded/matured with ferroptotic lysate possessed both 1) tumor antigens against which to generate adaptive responses and 2) inflammatory signals to induce DC maturation. Unloaded DC matured with LPS contained no antigens against which to generate responses and DC loaded only with F/T lysate remained immature and not generally capable of inducing adaptive immunity. Proper control should have included F/T lysate minimally adjuvanted with LPS; however, as stated above, more optimally adjuvanted with inflammatory signals known to induce TH1 polarization."*

Answer 5. We appreciate the reviewer's important point. To address this, we performed additional experiments using wild-type DCs prepared with and without LPS stimulation (**Suppl. Fig. 3E–G** and please see above Answer 4). We observed no difference in vaccine efficacy, confirming that additional LPS does not enhance the therapeutic effect of ferroptosis-based DC vaccines. These findings are consistent with our previous results using TLR4 KO DCs (**Fig. 5F–H** and please see above Answer 4), which lack the LPS receptor, further supporting that LPS signaling is not critical in this context.

Regarding the FT lysate control, we have included this group in both prophylactic (**Fig. 2**) and therapeutic (**Fig. 4**) experiments which have been adjuvanted with LPS, where it consistently showed no survival benefit compared to PBS or unloaded DCs. Based on these outcomes and considering the 3R principles, we believe additional LPS-adjuvanted FT experiments are unlikely to provide further insights.

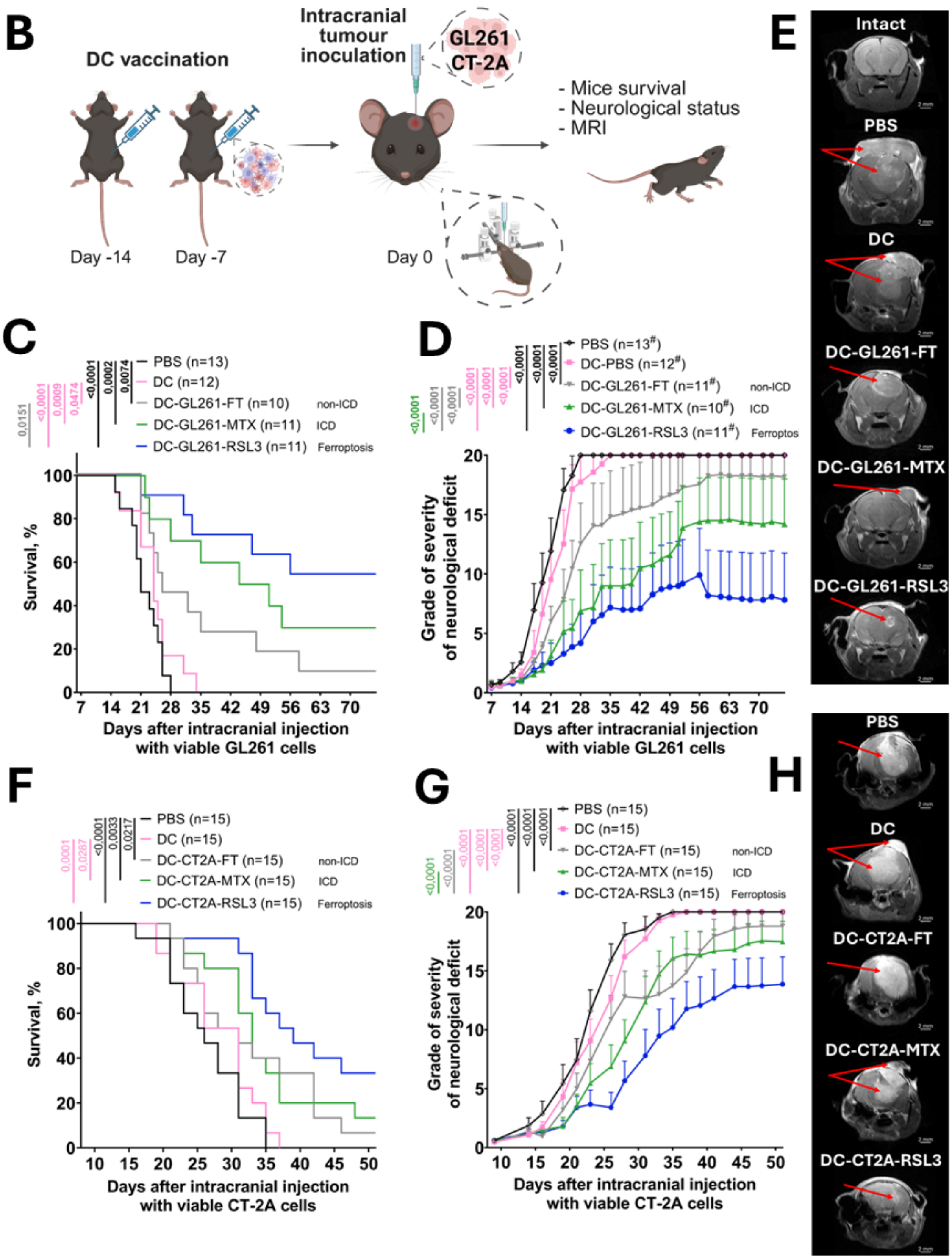

Figure 2. (B) Overview of the experimental setup to assess the effectiveness of DC vaccines in a prophylactic context using an orthotopic intracranial mouse model. Mice were administered 1*10^6 DC vaccines' cells on days -14 and -7, followed by challenge with an intracranial injection of viable glioma GL261 (2*10^4 cells) or CT-2A (5*10^5 cells) cells on day 0. Survival outcomes, neurological status and MRI scans were monitored thereafter.

Prophylactic vaccination with DC-GL261-RSL3

Mouse survival **(C),** neurological status **(D)** and MRI **(E)** were assessed after injection of the ferroptosis-armed DC vaccine, followed by intracranial challenge with viable glioma GL261 cells. Mouse survival and neurological status were assessed for up to 76 days. Control groups included mice injected with DC vaccines loaded with glioma GL261 lysate subjected to F/T cycles (negative control, non-ICD, DC-GL261-FT) or treated with MTX (2.5 µM, positive control-ICD, DC-GL261-MTX), and mice injected with unloaded DCs (negative control, DCs) or with PBS (negative control). #On day 23, three mice per group underwent MRI scans. In some of these mice (2 in the PBS group and 2 in the DC group), the glioma size had already reached humane endpoints, and they were euthanized following the MRI. These mice were included in the analysis of survival and neurological status. However, other mice (1 in the PBS and DC groups and 3 in each of the DC-GL261-FT, DC-GL261-MTX, and DC-GL261-RSL3 groups) did not reach humane endpoints and were euthanized after the MRI; these mice were excluded from the analysis. **(E)** Representative T1-weighted MRI images of coronal brain sections taken on day 23, n=3 mice per group. Arrows point to tumors. Statistical analysis

for mice survival was performed using Mantel-Cox logarithmic test. Statistical analysis of the neurological status of the mice was performed using a two-way ANOVA followed by Tukey's multiple comparisons correction.

Prophylactic vaccination with DC-CT2A-RSL3

Mouse survival **(F)**, neurological status **(G),** and MRI **(H)** were evaluated after injection of the ferroptosis-armed DC vaccines and intracranial challenge with viable glioma CT2A cells. Mouse survival and neurological status were monitored for up to 51 days. Control groups included mice injected with DC vaccines loaded with glioma CT-2A lysates subjected to F/T cycles (negative control, non-ICD, DC-CT2A-FT) or treated with MTX (2.5 μM, positive control-ICD, DC-CT2A-MTX), and mice injected with unloaded DCs (negative control, DCs) or PBS (negative control). **(H)** Representative T1-weighted MRI images of coronal brain sections taken on day 23, with the tumor indicated by arrows, n=4 mice per group. Statistical analysis for mice survival was determined by Mantel-Cox logarithmic test. Statistical analysis of the neurological status of the mice was performed using a two-way ANOVA followed by Tukey's multiple comparisons correction.

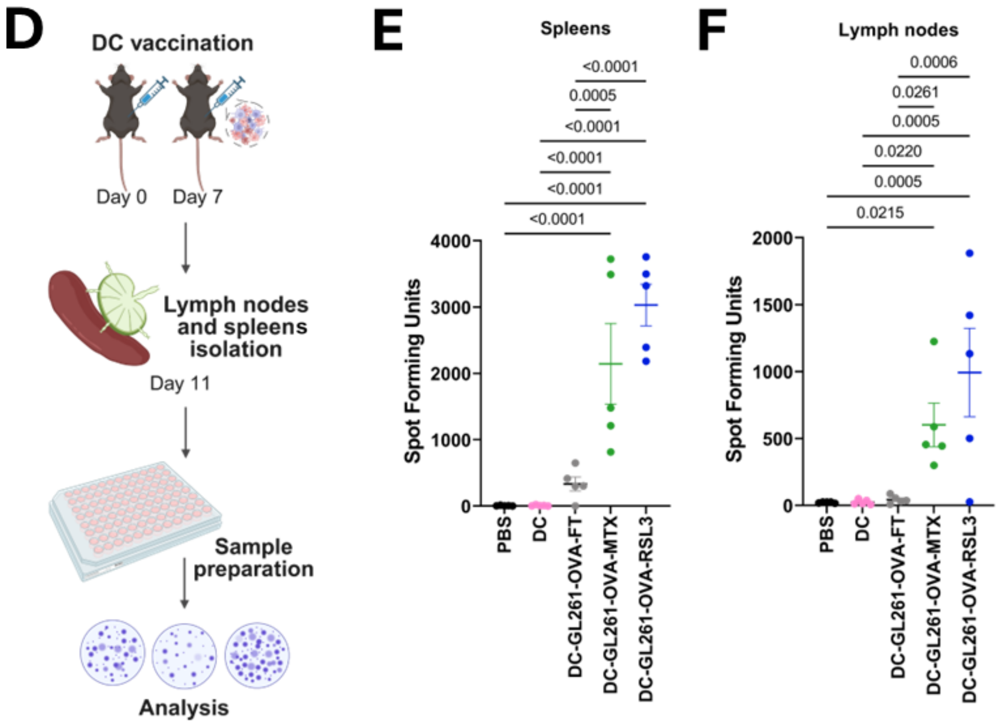

Figure 4. (D) Procedure to analyze the priming of cytotoxic T cells and IFN-γ production in mice immunized with DC vaccines on days 0 and 7. Four days after the final vaccination, the inguinal lymph nodes and splenocytes were isolated and re-stimulated *ex vivo* with the SIINFEKL peptide. **(E, F)** IFN-γ ELISpot assay of the spleens **(E)** and draining lymph nodes **(F)** from immunized mice. DC vaccines were loaded with ferroptotic GL261 lysates (DC-GL261-OVA-RSL3). Ferroptosis was induced by 2.5 μM RSL3. As controls, we used DC vaccines loaded with glioma GL261 cells subjected to F/T cycles (negative control: non-ICD, DC-GL261-OVA-FT), with MTX (2.5 μM, positive control: ICD, DC-GL261-OVA-MTX), unloaded DCs (negative control, DCs) or PBS (negative control). Each type of lysate was supplemented with 1 μM ovalbumin. The number of spot-forming units is presented as means ± SEM from data obtained from 5 mice. Statistical significance was determined using one-way ANOVA followed by Dunnett's multiple comparisons test.

Comment 6. *"Marginal results presented in figure 3G are the result of investigators administering DC intraperitoneally yet looking for reactive cells in the inguinal lymph nodes. If investigators wish to examine inguinal node resident T-cells, DC should be administered in the foot pad or hock. However, in the current model system that relies on IP injection, plentiful reactive T-cells should still be found through the analysis of splenocyte T-cell populations."*

Answer 6. In response, we repeated the experiment, isolating both lymph nodes and splenocytes for analysis. Although we maintained the intraperitoneal injection route to ensure consistency across experiments, we observed robust T-cell activation (IFN-γ ELISpot assay) upon SIINFEKL restimulation, particularly in splenocytes (**Fig. 4D–F** and please see above Answer 5), confirming a systemic immune response.

Comment 7. *“The investigators’ flow cytometry results are interesting and deserve additional follow-up. In murine DC, generally only CD86 expression is upregulated upon maturation whereas CD80 expression remains constant. Investigator’s findings that CD80 is also upregulated by ferroptotic lysates is noteworthy and should be compared to DC adjuvanted the with afforementioned TH1 polarizing ligands and cytokines. Representative flow plots should also be provided for review.”*

Answer 7. We appreciate the reviewer’s interest in our flow cytometry findings. To clarify, in vitro, ferroptotic lysates alone or combined with LPS did not induce selective DC maturation, as shown by CD80/CD86/MHC-II expression (**Fig. 5A–D**). LPS stimulation led to DC maturation across all groups, without specific enhancement by ferroptotic lysates.

However, *in vivo*, we observed a significant difference. DC vaccines loaded with FT lysates (with LPS) failed to induce a strong anti-tumor response (**Fig. 5F-H and Suppl. Fig. 3E-G**), whereas DCs loaded with ferroptotic lysates elicited potent immunity. Furthermore, LPS was dispensable for the efficacy of ferroptosis-based DC vaccines, highlighting the intrinsic immunogenicity of ferroptotic lysates. This suggests that the immunostimulatory effects of ferroptotic lysates are context-dependent (Catanzaro et al., 2024) and become evident in the *in vivo* setting.

Comment 8. *“It is not clear what point was trying to be made by blocking TLR-4 during the process of ferroprotic lysate-induced DC maturation. Immunologic danger is transduced through many dozens or possibly even hundreds of different pattern recognition receptors. Therefore the rationale of ruling out only a single PRR (TLR4) in detection of ferroptotic danger is of debatable significance.*

It would be more helpful to understand which PPR ARE responsible for detecting ferroptotic danger including HMGB1, ATP, and calreticulin since the authors demonstrate that these are important inflammatory components of ferroptotic cell lysates. For example, if the investigators wanted to rule in/rule out danger signaling through TLRs, they could utilize TRIF/MyD88 double KO DC to make the point.

In the discussion the authors cite continued immunogenicity of ferroptotic lysate even in the absence of TLR-4 as proof that there are no agonistic contaminants in their preparation. While it might be construed that there are no LPS contaminants in their preparation, an absence of all "agonistic contaminants" cannot be concluded through TLR-4 KO alone.”

Answer 8. Our rationale for blocking TLR4 was twofold. First, we detected HMGB1 derived from ferroptotic cells - a key DAMP known to promote DC maturation via TLR4 (Apetoh et al., 2007). However, detailed dissection of these pathways was beyond the scope of the current study, which aimed to establish the baseline immunogenicity of ferroptotic lysates in DC vaccination. Future studies will focus on elucidating the role of additional DAMPs (CRT, ATP) and receptors such as RAGE, P2X7, and CLEC family members.

In the revised manuscript, we have softened our conclusions to clarify that the absence of TLR4 dependence argues against LPS contamination specifically but does not exclude the presence of other immunostimulatory components within the lysate.

This clarification has been added to the *Discussion* section (page 11).

Comment 9. *“While analyzing proteomic differences between ferroptotic cell lysates and FT/MDX cell lysates , the authors note the differences in protein content and speculate that differential proteins generated through ferroptosis produce novel antigens against which T-cell responses might be made, thereby mediating better tumorcontrol/killing. Obviously this cannot possibly be true because the tumor cells have not undergone ferroptosis and therefore do not exhibit upregulated expression (or any expression) of proteins differentially expressed through induction of ferroptosis.”*

Answer 9. We thank the reviewer for this important clarification. Indeed, tumor cells in vivo are not induced to undergo ferroptosis or apoptosis and therefore do not exhibit the stress-induced

proteomic profiles observed in lysates. Our intention was not to imply that ferroptosis generates neoantigens present on untreated tumors, but rather that ferroptosis creates an immunogenic context that enhances the presentation of tumor-associated antigens (TAAs).
Specifically, ferroptosis may facilitate cross-presentation by promoting oxidative modifications (*e.g.,* carbonylation) that enhance antigen uptake, processing, or recognition by dendritic cells, thereby increasing the immunogenicity of existing self-antigens. This concept is supported by growing evidence that oxidative stress during immunogenic cell death can modulate antigenicity without altering the underlying antigen repertoire (Catanzaro et al., 2024; Radogna et al., 2019).
We have revised the *Discussion* to clarify that our findings suggest ferroptosis enhances the immunogenic potential of pre-existing TAAs, rather than generating novel tumor antigens.
This clarification has been added on page 12.

Comment 10. *"The data depicted in figure 5F is a second (important) example of a likely type II error that would likely resolve with larger sample size/additional experimental rigor."*

Answer 10. We appreciate the reviewer's concern regarding sample size. To address this, we based our sample size calculation on a previously published DC vaccination study in an orthotopic brain tumor model (Garg et al., 2016). Using G*Power 3.1.9 software (α = 0.05, power = 0.8), the analysis indicated that a minimum of 6 mice per group was required. In the HMGB1 blocking experiment, we included 7 mice, ensuring the study was appropriately powered.
The sample size was calculated with G*Power 3.1.9 software:

Analysis:	A priori: Compute required sample size		
Input:	Effect size f	=	5
	α err prob	=	0,05
	Power (1-β err prob)	=	0,8
	Numerator df	=	10
	Number of groups	=	3
Output:	Noncentrality parameter λ	=	150
	Critical F	=	8.7855247
	Denominator df	=	3
	**Total sample size**	**=**	**6**
	Actual power	=	0.8502608

Additionally, we performed a complementary experiment using TLR4-knockout DCs, as TLR4 is a primary receptor for HMGB1. Consistent with the HMGB1-blocking results, TLR4-deficient DCs retained full vaccine efficacy, showing no reduction in survival compared to wild-type DCs (**Fig. 5G; Suppl. Fig. 9A**). Both approaches independently indicate that HMGB1 signaling is not critical for the anti-tumor effect of ferroptosis-based DC vaccines in this model.

Comment 11. *"One curious claim is that blocking of either ATP or CRT is enough to completely abrogate the full inflammatory effect of ferroptotic lysate even though in each case, the other danger signal remains unblocked. Further, authors' claim that simultaneous blocking of all three inflammatory components HMGB1, ATP, and CRT results in more impaired inflammatory response than blocking of any component individually is not true. Blocking of all three imparts the same anti-inflammatory effect (full) as the individual blocking of either ATP or CRT."*

Answer 11. We thank the reviewer for this observation. Our data show that blocking either ATP or CRT alone is sufficient to significantly impair the efficacy of the ferroptosis-based DC vaccine.

Combined blockade of HMGB1, ATP, and CRT does not further reduce the immune response beyond the effect seen with ATP or CRT inhibition individually.
We have revised the manuscript to correct this overstatement. The text now describes these effects as "maximally suppressive" avoiding any implication of additive or synergistic suppression.

Comment 12. *"According to the materials and methods, BMDC in this study were generated through incubation in GM-CSF alone without concomitant presence of IL-4. Therefore this protocol likely resulted in a cell population substantially skewed toward the macrophage lineage. This potentially explains the need to administer such high numbers of cells (1 million) in the vaccination protocol. This may be rectified by application of 10 ng/ml IL-4 during BMDC differentiation."*
Answer 12. We thank the reviewer for this point. Many widely accepted murine DC vaccination studies generate BMDCs using GM-CSF alone, especially in preclinical tumor models (Han et al., 2023; Kim & Kim, 2019; Sauter et al., 2022; Wiernicki et al., 2022). Nonetheless, we agree that incorporating IL-4 and maturation cocktails (*e.g.,* TNF, PGE_2, TLR agonists) could be valuable for future studies, especially in a translational context (Gilboa, 2007; Herrera et al., 2002; Kalantari et al., 2011).
Regarding cell dose, we would like to clarify that administering 1×10^6 BMDCs per mouse is standard practice in murine vaccination protocols. Numerous tumor immunotherapy studies have used similar or even higher doses to induce robust immune responses (Masuda et al., 2019; Sakai et al., 2004; Vandenberk et al., 2016).

Minor comments

Minor comment 1. *"For the data depicted in figures 2D, 2G, 3C, and 3E, methods state that the assessor of neurologic deficits was blinded; however, this can mean a number of different things. If assessor was blinded only to which group was being assessed but was still aware that all mice being assessed (i.e. in the same cage) all belonged to one group, then bias was still likely introduced in scoring. Please state in the methods whether assessor was only blinded as to the identity of each assessment group or was truly blinded through randomized assessment ordering."*
Answer 1. We thank the reviewer for this important clarification. The assessor was blinded to the identity of each assessment group during scoring, although aware of the experimental groups involved. This information has been added to the *Materials and Methods* section on page 19.

Minor comment 2. *"It is unclear what the Y axis of figure 5A is showing. The percent of cells CRT+ Systox- cannot exceed 100% of cells, yet positive control shows a level of 700%. Therefore please accurately rename what this axis indicates. "*
Answer 2. We thank the reviewer for pointing this out. The Y-axis represents the percentage of CRT^+ cells within the viable ($Sytox^-$) population. The axis label has been corrected accordingly in the revised figure.

Minor comment 3. *"The manuscript is written extremely well, however there is a single non-English sentence construction on line 380. Replaced the word "died" with "killed"."*
Answer 3. The wording has been corrected as suggested.

References

Aaes, T. L., Kaczmarek, A., Delvaeye, T., De Craene, B., De Koker, S., Heyndrickx, L., Delrue, I., Taminau, J., Wiernicki, B., De Groote, P., Garg, A. D., Leybaert, L., Grooten, J., Bertrand, M. J. M., Agostinis, P., Berx, G., Declercq, W., Vandenabeele, P., & Krysko, D. V. (2016). Vaccination with Necroptotic Cancer Cells Induces Efficient Anti-tumor Immunity. *Cell Reports*, *15*(2), 274–287. https://doi.org/10.1016/j.celrep.2016.03.037

Abate, L. E., Mukherjee, P., & Seyfried, T. N. (2006). Gene-linked shift in ganglioside distribution influences growth and vascularity in a mouse astrocytoma. *Journal of Neurochemistry*, *98*(6), 1973–1984. https://doi.org/10.1111/j.1471-4159.2006.04097.x

Apetoh, L., Ghiringhelli, F., Tesniere, A., Criollo, A., Ortiz, C., Lidereau, R., Mariette, C., Chaput, N., Mira, J., Delaloge, S., André, F., Tursz, T., Kroemer, G., & Zitvogel, L. (2007). The interaction between HMGB1 and TLR4 dictates the outcome of anticancer chemotherapy and radiotherapy. *Immunological Reviews*, *220*(1), 47–59. https://doi.org/10.1111/j.1600-065X.2007.00573.x

Apetoh, L., Ghiringhelli, F., Tesniere, A., Obeid, M., Ortiz, C., Criollo, A., Mignot, G., Maiuri, M. C., Ullrich, E., Saulnier, P., Yang, H., Amigorena, S., Ryffel, B., Barrat, F. J., Saftig, P., Levi, F., Lidereau, R., Nogues, C., Mira, J.-P., … Zitvogel, L. (2007). Toll-like receptor 4–dependent contribution of the immune system to anticancer chemotherapy and radiotherapy. *Nature Medicine*, *13*(9), 1050–1059. https://doi.org/10.1038/nm1622

Basirjafar, P., Zandvakili, R., Masoumi, J., Zainodini, N., Taghipour, Z., Khorramdelazad, H., Yousefi, S., Tavakoli, T., Vatanparast, M., Safdel, S., Gheitasi, M., Ayoobi, F., Naseri, B., & Jafarzadeh, A. (2024). Leptin/lipopolysaccharide-treated dendritic cell vaccine improved cellular immune responses in an animal model of breast cancer. *Immunopharmacology and Immunotoxicology*, *46*(1), 73–85. https://doi.org/10.1080/08923973.2023.2253989

Casares, N., Pequignot, M. O., Tesniere, A., Ghiringhelli, F., Roux, S., Chaput, N., Schmitt, E., Hamai, A., Hervas-Stubbs, S., Obeid, M., Coutant, F., Métivier, D., Pichard, E., Aucouturier, P., Pierron, G., Garrido, C., Zitvogel, L., & Kroemer, G. (2005). Caspase-dependent immunogenicity of doxorubicin-induced tumor cell death. *Journal of Experimental Medicine*, *202*(12), 1691–1701. https://doi.org/10.1084/jem.20050915

Catanzaro, E., Demuynck, R., Naessens, F., Galluzzi, L., & Krysko, D. V. (2024). Immunogenicity of ferroptosis in cancer: a matter of context? *Trends in Cancer*, *10*(5), 407–416. https://doi.org/10.1016/j.trecan.2024.01.013

Chiva, C., Olivella, R., Borràs, E., Espadas, G., Pastor, O., Solé, A., & Sabidó, E. (2018). QCloud: A cloud-based quality control system for mass spectrometry-based proteomics laboratories. *PloS One*, *13*(1), e0189209. https://doi.org/10.1371/journal.pone.0189209

Crespo-Diaz, R., Behfar, A., Butler, G. W., Padley, D. J., Sarr, M. G., Bartunek, J., Dietz, A. B., & Terzic, A. (2011). Platelet Lysate Consisting of a Natural Repair Proteome Supports Human Mesenchymal Stem Cell Proliferation and Chromosomal Stability. *Cell Transplantation*, *20*(6), 797–812. https://doi.org/10.3727/096368910X543376

D'Aprile, S., Denaro, S., Lavoro, A., Candido, S., Giallongo, S., Torrisi, F., Salvatorelli, L., Lazzarino, G., Amorini, A. M., Lazzarino, G., Magro, G., Tibullo, D., Libra, M., Giallongo, C., Vicario, N., & Parenti, R. (2024). Glioblastoma mesenchymal subtype enhances antioxidant defence to reduce susceptibility to ferroptosis. *Scientific Reports*, *14*(1), 20770. https://doi.org/10.1038/s41598-024-72024-8

Duhen, T., Duhen, R., Montler, R., Moses, J., Moudgil, T., de Miranda, N. F., Goodall, C. P., Blair, T. C., Fox, B. A., McDermott, J. E., Chang, S.-C., Grunkemeier, G., Leidner, R., Bell, R. B., & Weinberg, A. D. (2018). Co-expression of CD39 and CD103 identifies tumor-reactive CD8 T cells in human solid tumors. *Nature Communications*, *9*(1), 2724. https://doi.org/10.1038/s41467-018-05072-0

Efimova, I., Catanzaro, E., Van der Meeren, L., Turubanova, V. D., Hammad, H., Mishchenko, T. A., Vedunova, M. V, Fimognari, C., Bachert, C., Coppieters, F., Lefever, S., Skirtach, A. G., Krysko, O., & Krysko, D. V. (2020). Vaccination with early ferroptotic cancer cells induces efficient antitumor immunity. *Journal for ImmunoTherapy of Cancer*, *8*(2), e001369. https://doi.org/10.1136/jitc-2020-001369

Fucikova, J., Kepp, O., Kasikova, L., Petroni, G., Yamazaki, T., Liu, P., Zhao, L., Spisek, R., Kroemer, G., & Galluzzi, L. (2020). Detection of immunogenic cell death and its relevance for cancer therapy. *Cell Death & Disease*, *11*(11), 1013. https://doi.org/10.1038/s41419-020-03221-2

Fulda, S. (2009). Tumor resistance to apoptosis. *International Journal of Cancer*, *124*(3), 511–515. https://doi.org/10.1002/ijc.24064

Galluzzi, L., Kepp, O., Hett, E., Kroemer, G., & Marincola, F. M. (2023). Immunogenic cell death in cancer: concept and therapeutic implications. *Journal of Translational Medicine*, *21*(1), 162. https://doi.org/10.1186/s12967-023-04017-6

Galluzzi, L., Vitale, I., Warren, S., Adjemian, S., Agostinis, P., Martinez, A. B., Chan, T. A., Coukos, G., Demaria, S., Deutsch, E., Draganov, D., Edelson, R. L., Formenti, S. C., Fucikova, J., Gabriele, L., Gaipl, U. S., Gameiro, S. R., Garg, A. D., Golden, E., ... Marincola, F. M. (2020). Consensus guidelines for the definition, detection and interpretation of immunogenic cell death. *Journal for ImmunoTherapy of Cancer*, *8*(1), e000337. https://doi.org/10.1136/jitc-2019-000337

Garg, A. D., Vandenberk, L., Koks, C., Verschuere, T., Boon, L., Van Gool, S. W., & Agostinis, P. (2016). Dendritic cell vaccines based on immunogenic cell death elicit danger signals and T cell-driven rejection of high-grade glioma. *Science Translational Medicine*, *8*(328). https://doi.org/10.1126/scitranslmed.aae0105

Gilboa, E. (2007). DC-based cancer vaccines. *Journal of Clinical Investigation*, *117*(5), 1195–1203. https://doi.org/10.1172/JCI31205

Goldszmid, R. S., Idoyaga, J., Bravo, A. I., Steinman, R., Mordoh, J., & Wainstok, R. (2003). Dendritic Cells Charged with Apoptotic Tumor Cells Induce Long-Lived Protective CD4+ and CD8+ T Cell Immunity against B16 Melanoma. *The Journal of Immunology*, *171*(11), 5940–5947. https://doi.org/10.4049/jimmunol.171.11.5940

Han, J., Bhatta, R., Liu, Y., Bo, Y., Elosegui-Artola, A., & Wang, H. (2023). Metabolic glycan labeling immobilizes dendritic cell membrane and enhances antitumor efficacy of dendritic cell vaccine. *Nature Communications*, *14*(1), 5049. https://doi.org/10.1038/s41467-023-40886-7

Han, T. H., Jin, P., Ren, J., Slezak, S., Marincola, F. M., & Stroncek, D. F. (2009). Evaluation of 3 Clinical Dendritic Cell Maturation Protocols Containing Lipopolysaccharide and Interferon-γ. *Journal of Immunotherapy*, *32*(4), 399–407. https://doi.org/10.1097/CJI.0b013e31819e1773

Hanahan, D., & Weinberg, R. A. (2011). Hallmarks of Cancer: The Next Generation. *Cell*, *144*(5), 646–674. https://doi.org/10.1016/j.cell.2011.02.013

Hayashi, K., Nikolos, F., Lee, Y. C., Jain, A., Tsouko, E., Gao, H., Kasabyan, A., Leung, H. E., Osipov, A., Jung, S. Y., Kurtova, A. V., & Chan, K. S. (2020). Tipping the immunostimulatory and inhibitory DAMP balance to harness immunogenic cell death. *Nature Communications*, *11*(1), 6299. https://doi.org/10.1038/s41467-020-19970-9

Herrera, O. B., Brett, S., & Lechler, R. I. (2002). Infection of mouse bone marrow-derived dendritic cells with recombinant adenovirus vectors leads to presentation of encoded antigen by both MHC class I and class II molecules—potential benefits in vaccine design. *Vaccine*, *21*(3–4), 231–242. https://doi.org/10.1016/S0264-410X(02)00448-6

Hodi, F. S., Giobbie-Hurder, A., Adu-Berchie, K., Ranasinghe, S., Lako, A., Severgnini, M., Thrash, E. M., Weirather, J. L., Baginska, J., Manos, M. P., Doherty, E. J., Stafford, A., Daley, H., Ritz, J., Ott, P. A., Pfaff, K. L., Rodig, S. J., Yoon, C. H., Dranoff, G., & Mooney, D. J. (2025). First-in-Human Clinical Trial of Vaccination with WDVAX, a Dendritic Cell–Activating Scaffold Incorporating Autologous Tumor Cell Lysate, in Patients with Metastatic Melanoma. *Cancer Immunology Research*, *13*(7), 978–989. https://doi.org/10.1158/2326-6066.CIR-24-0333

Isaacs, J. F., Degefu, H. N., Chen, T., Kleist, S. A., Musial, S. C., Ford, M. A., Searles, T. G., Lin, C.-C., Skorput, A. G. J., Shirai, K., Turk, M. J., Zanazzi, G. J., & Rosato, P. C. (2024). *CD39 is expressed on functional effector and tissue resident memory CD8+ T cells*. https://doi.org/10.1101/2024.03.15.585252

Kalantari, T., Kamali-Sarvestani, E., Ciric, B., Karimi, M. H., Kalantari, M., Faridar, A., Xu, H., & Rostami, A. (2011). Generation of immunogenic and tolerogenic clinical-grade dendritic cells. *Immunologic Research*, *51*(2–3), 153–160. https://doi.org/10.1007/s12026-011-8255-5

Kepp, O., Senovilla, L., Vitale, I., Vacchelli, E., Adjemian, S., Agostinis, P., Apetoh, L., Aranda, F., Barnaba, V., Bloy, N., Bracci, L., Breckpot, K., Brough, D., Buqué, A., Castro, M. G., Cirone, M., Colombo, M. I., Cremer, I., Demaria, S., … Galluzzi, L. (2014). Consensus guidelines for the detection of immunogenic cell death. *OncoImmunology*, *3*(9), e955691. https://doi.org/10.4161/21624011.2014.955691

Kim, M. K., & Kim, J. (2019). Properties of immature and mature dendritic cells: phenotype, morphology, phagocytosis, and migration. *RSC Advances*, *9*(20), 11230–11238. https://doi.org/10.1039/C9RA00818G

Kim, R., Hashimoto, A., Markosyan, N., Tyurin, V. A., Tyurina, Y. Y., Kar, G., Fu, S., Sehgal, M., Garcia-Gerique, L., Kossenkov, A., Gebregziabher, B. A., Tobias, J. W., Hicks, K., Halpin, R. A., Cvetesic, N., Deng, H., Donthireddy, L., Greenberg, A., Nam, B., … Gabrilovich, D. I. (2022). Ferroptosis of tumour neutrophils causes immune suppression in cancer. *Nature*, *612*(7939), 338–346. https://doi.org/10.1038/s41586-022-05443-0

Kokhaei, P., Choudhury, A., Mahdian, R., Lundin, J., Moshfegh, A., Österborg, A., & Mellstedt, H. (2004). Apoptotic tumor cells are superior to tumor cell lysate, and tumor cell RNA in induction of autologous T cell response in B-CLL. *Leukemia*, *18*(11), 1810–1815. https://doi.org/10.1038/sj.leu.2403517

Kroemer, G., Galassi, C., Zitvogel, L., & Galluzzi, L. (2022). Immunogenic cell stress and death. *Nature Immunology*, *23*(4), 487–500. https://doi.org/10.1038/s41590-022-01132-2

Kroemer, G., Galluzzi, L., Kepp, O., & Zitvogel, L. (2013). Immunogenic Cell Death in Cancer Therapy. *Annual Review of Immunology*, *31*(1), 51–72. https://doi.org/10.1146/annurev-immunol-032712-100008

Krysko, O., Aaes, T. L., Kagan, V. E., D'Herde, K., Bachert, C., Leybaert, L., Vandenabeele, P., & Krysko, D. V. (2017). Necroptotic cell death in anti-cancer therapy. *Immunological Reviews*, *280*(1), 207–219. https://doi.org/10.1111/imr.12583

Krysko, O., Løve Aaes, T., Bachert, C., Vandenabeele, P., & Krysko, D. V. (2013). Many faces of DAMPs in cancer therapy. *Cell Death & Disease*, *4*(5), e631–e631. https://doi.org/10.1038/cddis.2013.156

Leten, C., Struys, T., Dresselaers, T., & Himmelreich, U. (2014). In vivo and ex vivo assessment of the blood brain barrier integrity in different glioblastoma animal models. *Journal of Neuro-Oncology*, *119*(2), 297–306. https://doi.org/10.1007/s11060-014-1514-2

Li, C., Sun, H., Wei, W., Liu, Q., Wang, Y., Zhang, Y., Lian, F., Liu, F., Li, C., Ying, K., Huo, H., Qi, Z., & Li, B. (2020). Mitoxantrone triggers immunogenic prostate cancer cell death via p53-dependent PERK expression. *Cellular Oncology*, *43*(6), 1099–1116. https://doi.org/10.1007/s13402-020-00544-2

Liau, L. M., Ashkan, K., Brem, S., Campian, J. L., Trusheim, J. E., Iwamoto, F. M., Tran, D. D., Ansstas, G., Cobbs, C. S., Heth, J. A., Salacz, M. E., D'Andre, S., Aiken, R. D., Moshel, Y. A., Nam, J. Y., Pillainayagam, C. P., Wagner, S. A., Walter, K. A., Chaudhary, R., … Bosch, M. L. (2023). Association of Autologous Tumor Lysate-Loaded Dendritic Cell Vaccination With Extension of Survival Among Patients With Newly Diagnosed and Recurrent Glioblastoma. *JAMA Oncology*, *9*(1), 112. https://doi.org/10.1001/jamaoncol.2022.5370

Liu, P., Zhao, L., Loos, F., Iribarren, K., Lachkar, S., Zhou, H., Gomes-da-Silva, L. C., Chen, G., Bezu, L., Boncompain, G., Perez, F., Zitvogel, L., Kepp, O., & Kroemer, G. (2017). Identification of pharmacological agents that induce HMGB1 release. *Scientific Reports*, *7*(1), 14915. https://doi.org/10.1038/s41598-017-14848-1

Maes, W., Rosas, G. G., Verbinnen, B., Boon, L., De Vleeschouwer, S., Ceuppens, J. L., & Van Gool, S. W. (2009). DC vaccination with anti-CD25 treatment leads to long-term immunity

against experimental glioma. *Neuro-Oncology*, *11*(5), 529–542. https://doi.org/10.1215/15228517-2009-004

Maia, T. M., Staes, A., Plasman, K., Pauwels, J., Boucher, K., Argentini, A., Martens, L., Montoye, T., Gevaert, K., & Impens, F. (2020). Simple Peptide Quantification Approach for MS-Based Proteomics Quality Control. *ACS Omega*, *5*(12), 6754–6762. https://doi.org/10.1021/acsomega.0c00080

Masuda, Y., Nakayama, Y., Mukae, T., Tanaka, A., Naito, K., & Konishi, M. (2019). Maturation of dendritic cells by maitake α-glucan enhances anti-cancer effect of dendritic cell vaccination. *International Immunopharmacology*, *67*, 408–416. https://doi.org/10.1016/j.intimp.2018.12.039

McKelvey, K. J., Hudson, A. L., Prasanna Kumar, R., Wilmott, J. S., Attrill, G. H., Long, G. V., Scolyer, R. A., Clarke, S. J., Wheeler, H. R., Diakos, C. I., & Howell, V. M. (2020). Temporal and spatial modulation of the tumor and systemic immune response in the murine Gl261 glioma model. *PLOS ONE*, *15*(4), e0226444. https://doi.org/10.1371/journal.pone.0226444

Mikolajewicz, N., Tatari, N., Wei, J., Savage, N., Granda Farias, A., Dimitrov, V., Chen, D., Zador, Z., Dasgupta, K., Aguilera-Uribe, M., Xiao, Y.-X., Lee, S. Y., Mero, P., McKenna, D., Venugopal, C., Brown, K. R., Han, H., Singh, S., & Moffat, J. (2024). Functional profiling of murine glioma models highlights targetable immune evasion phenotypes. *Acta Neuropathologica*, *148*(1), 74. https://doi.org/10.1007/s00401-024-02831-w

Obeid, M., Tesniere, A., Ghiringhelli, F., Fimia, G. M., Apetoh, L., Perfettini, J.-L., Castedo, M., Mignot, G., Panaretakis, T., Casares, N., Métivier, D., Larochette, N., van Endert, P., Ciccosanti, F., Piacentini, M., Zitvogel, L., & Kroemer, G. (2007). Calreticulin exposure dictates the immunogenicity of cancer cell death. *Nature Medicine*, *13*(1), 54–61. https://doi.org/10.1038/nm1523

Palucka, K., & Banchereau, J. (2013). Dendritic-Cell-Based Therapeutic Cancer Vaccines. *Immunity*, *39*(1), 38–48. https://doi.org/10.1016/j.immuni.2013.07.004

Parney, I. F., Anderson, S. K., Gustafson, M. P., Steinmetz, S., Peterson, T. E., Kroneman, T. N., Raghunathan, A., O’Neill, B. P., Buckner, J. C., Solseth, M., & Dietz, A. B. (2022). Phase I trial of adjuvant mature autologous dendritic cell/allogeneic tumor lysate vaccines in combination with temozolomide in newly diagnosed glioblastoma. *Neuro-Oncology Advances*, *4*(1). https://doi.org/10.1093/noajnl/vdac089

Pellegatta, S., Eoli, M., Frigerio, S., Antozzi, C., Bruzzone, M. G., Cantini, G., Nava, S., Anghileri, E., Cuppini, L., Cuccarini, V., Ciusani, E., Dossena, M., Pollo, B., Mantegazza, R., Parati, E. A., & Finocchiaro, G. (2013). The natural killer cell response and tumor debulking are associated with prolonged survival in recurrent glioblastoma patients receiving dendritic cells loaded with autologous tumor lysates. *OncoImmunology*, *2*(3), e23401. https://doi.org/10.4161/onci.23401

Perneger, T. V. (1998). What’s wrong with Bonferroni adjustments. *BMJ*, *316*(7139), 1236–1238. https://doi.org/10.1136/bmj.316.7139.1236

Pfitzer, L., Lybaert, L., Bogaert, C., & Fant, B. (2022). *Improving T-cell mediated immunogenic epitope identification via machine learning: the neoIM model*. https://doi.org/10.1101/2022.06.03.494687

Pichol-Thievend, C., Anezo, O., Pettiwala, A. M., Bourmeau, G., Montagne, R., Lyne, A.-M., Guichet, P.-O., Deshors, P., Ballestín, A., Blanchard, B., Reveilles, J., Ravi, V. M., Joseph, K., Heiland, D. H., Julien, B., Leboucher, S., Besse, L., Legoix, P., Dingli, F., … Seano, G. (2024). VC-resist glioblastoma cell state: vessel co-option as a key driver of chemoradiation resistance. *Nature Communications*, *15*(1), 3602. https://doi.org/10.1038/s41467-024-47985-z

Radogna, F., Dicato, M., & Diederich, M. (2019). Natural modulators of the hallmarks of immunogenic cell death. *Biochemical Pharmacology*, *162*, 55–70. https://doi.org/10.1016/j.bcp.2018.12.016

Rapp, M., Grauer, O. M., Kamp, M., Sevens, N., Zotz, N., Sabel, M., & Sorg, R. V. (2018). A randomized controlled phase II trial of vaccination with lysate-loaded, mature dendritic cells integrated into standard radiochemotherapy of newly diagnosed glioblastoma (GlioVax): study protocol for a randomized controlled trial. *Trials*, *19*(1), 293. https://doi.org/10.1186/s13063-018-2659-7

Ritchie, M. E., Phipson, B., Wu, D., Hu, Y., Law, C. W., Shi, W., & Smyth, G. K. (2015). limma powers differential expression analyses for RNA-sequencing and microarray studies. *Nucleic Acids Research*, *43*(7), e47. https://doi.org/10.1093/nar/gkv007

Sabado, R. L., Balan, S., & Bhardwaj, N. (2017). Dendritic cell-based immunotherapy. *Cell Research*, *27*(1), 74–95. https://doi.org/10.1038/cr.2016.157

Sakai, Y., Morrison, B. J., Burke, J. D., Park, J.-M., Terabe, M., Janik, J. E., Forni, G., Berzofsky, J. A., & Morris, J. C. (2004). Vaccination by Genetically Modified Dendritic Cells Expressing a Truncated ***neu*** Oncogene Prevents Development of Breast Cancer in Transgenic Mice. *Cancer Research*, *64*(21), 8022–8028. https://doi.org/10.1158/0008-5472.CAN-03-3442

Sauter, M., Sauter, R. J., Nording, H., Olbrich, M., Emschermann, F., & Langer, H. F. (2022). Protocol to isolate and analyze mouse bone marrow derived dendritic cells (BMDC). *STAR Protocols*, *3*(3), 101664. https://doi.org/10.1016/j.xpro.2022.101664

Shao, X. M., Bhattacharya, R., Huang, J., Sivakumar, I. K. A., Tokheim, C., Zheng, L., Hirsch, D., Kaminow, B., Omdahl, A., Bonsack, M., Riemer, A. B., Velculescu, V. E., Anagnostou, V., Pagel, K. A., & Karchin, R. (2020). High-Throughput Prediction of MHC Class I and II Neoantigens with MHCnuggets. *Cancer Immunology Research*, *8*(3), 396–408. https://doi.org/10.1158/2326-6066.CIR-19-0464

Sukkurwala, A. Q., Adjemian, S., Senovilla, L., Michaud, M., Spaggiari, S., Vacchelli, E., Baracco, E. E., Galluzzi, L., Zitvogel, L., Kepp, O., & Kroemer, G. (2014). Screening of novel immunogenic cell death inducers within the NCI Mechanistic Diversity Set. *OncoImmunology*, *3*(4), e28473. https://doi.org/10.4161/onci.28473

Szatmári, T., Lumniczky, K., Désaknai, S., Trajcevski, S., Hídvégi, E. J., Hamada, H., & Sáfrány, G. (2006). Detailed characterization of the mouse glioma 261 tumor model for experimental glioblastoma therapy. *Cancer Science*, *97*(6), 546–553. https://doi.org/10.1111/j.1349-7006.2006.00208.x

Tanoue, K., Ohmura, H., Uehara, K., Ito, M., Yamaguchi, K., Tsuchihashi, K., Shinohara, Y., Lu, P., Tamura, S., Shimokawa, H., Isobe, T., Ariyama, H., Shibata, Y., Tanaka, R., Kusaba, H., Esaki, T., Mitsugi, K., Kiyozawa, D., Iwasaki, T., … Baba, E. (2024). Spatial dynamics of CD39+CD8+ exhausted T cell reveal tertiary lymphoid structures-mediated response to PD-1 blockade in esophageal cancer. *Nature Communications*, *15*(1), 9033. https://doi.org/10.1038/s41467-024-53262-w

Tesniere, A., Schlemmer, F., Boige, V., Kepp, O., Martins, I., Ghiringhelli, F., Aymeric, L., Michaud, M., Apetoh, L., Barault, L., Mendiboure, J., Pignon, J.-P., Jooste, V., van Endert, P., Ducreux, M., Zitvogel, L., Piard, F., & Kroemer, G. (2010). Immunogenic death of colon cancer cells treated with oxaliplatin. *Oncogene*, *29*(4), 482–491. https://doi.org/10.1038/onc.2009.356

Tirapu, I., Lewis, A., Kreutz, M., McLinden, H., & Diebold, S. S. (2008). Freeze-and-thaw-disrupted tumour cells impair the responsiveness of DC to TLR stimulation. *European Journal of Immunology*, *38*(10), 2740–2750. https://doi.org/10.1002/eji.200838284

Turubanova, V. D., Balalaeva, I. V., Mishchenko, T. A., Catanzaro, E., Alzeibak, R., Peskova, N. N., Efimova, I., Bachert, C., Mitroshina, E. V., Krysko, O., Vedunova, M. V., & Krysko, D. V. (2019). Immunogenic cell death induced by a new photodynamic therapy based on photosens and photodithazine. *Journal for ImmunoTherapy of Cancer*, *7*(1). https://doi.org/10.1186/s40425-019-0826-3

Vandenberk, L., Garg, A. D., Verschuere, T., Koks, C., Belmans, J., Beullens, M., Agostinis, P., De Vleeschouwer, S., & Van Gool, S. W. (2016). Irradiation of necrotic cancer cells, employed for pulsing dendritic cells (DCs), potentiates DC vaccine-induced antitumor immunity against high-grade glioma. *OncoImmunology*, *5*(2), e1083669. https://doi.org/10.1080/2162402X.2015.1083669

Vedunova, M., Turubanova, V., Vershinina, O., Savyuk, M., Efimova, I., Mishchenko, T., Raedt, R., Vral, A., Vanhove, C., Korsakova, D., Bachert, C., Coppieters, F., Agostinis, P., Garg, A. D., Ivanchenko, M., Krysko, O., & Krysko, D. V. (2022). DC vaccines loaded with glioma cells killed by photodynamic therapy induce Th17 anti-tumor immunity and provide a four-gene signature for glioma prognosis. *Cell Death & Disease*, *13*(12), 1062. https://doi.org/10.1038/s41419-022-05514-0

Wang, Z., Tang, Y., & Li, Q. (2025). A self-assembling nanoplatform for pyroptosis and ferroptosis enhanced cancer photoimmunotherapy. *Light: Science & Applications*, *14*(1), 16. https://doi.org/10.1038/s41377-024-01673-1

Wiernicki, B., Maschalidi, S., Pinney, J., Adjemian, S., Vanden Berghe, T., Ravichandran, K. S., & Vandenabeele, P. (2022). Cancer cells dying from ferroptosis impede dendritic cell-mediated anti-tumor immunity. *Nature Communications*, *13*(1), 3676. https://doi.org/10.1038/s41467-022-31218-2

Wolburg, H., Noell, S., Fallier-Becker, P., Mack, A. F., & Wolburg-Buchholz, K. (2012). The disturbed blood–brain barrier in human glioblastoma. *Molecular Aspects of Medicine*, *33*(5–6), 579–589. https://doi.org/10.1016/j.mam.2012.02.003

Zhang, X., Smits, A. H., van Tilburg, G. B., Ovaa, H., Huber, W., & Vermeulen, M. (2018). Proteome-wide identification of ubiquitin interactions using UbIA-MS. *Nature Protocols*, *13*(3), 530–550. https://doi.org/10.1038/nprot.2017.147

Zhu, H., & Lu, B. (2015). Multiple comparisons for survival data with propensity score adjustment. *Computational Statistics & Data Analysis*, *86*, 42–51. https://doi.org/10.1016/j.csda.2015.01.001

Reviewer #1

"Authors addressed all my concerns and questions. I have no additional comments and think this is a strong report."

We thank the reviewer for the positive feedback and for acknowledging the revisions made. We greatly appreciate the constructive input provided during the review process, which helped us to strengthen the manuscript.

Reviewer #2

"The authors addressed my comments and added all the necessary information regarding the proteomics data analysis. However, their revision raised some additional questions. The authors added a prediction of the MHC-presented peptides. They used the MHCnuggets tool to predict the presented peptides on MHCI and MHCII. In the supplementary Tables the authors share 18K and 14K peptides that are predicted to be presented and elicit immune response. Given the known high error rates of many of these algorithms, it seems unlikely that these predictions are correct and it is not convincing that the list of potential MHC peptides is reliable. It is also uncommon to use a single predictive tool and gain any insights. In addition, as the authors themselves indicate in their response to reviewers, the neoantigens that are elevated in the ferroptotic cells may harbor ferroptosis/redox-related modifications. Altogether, the logic of these analyses is not clear- The prediction tools are not expected to unravel the modified peptides, and these modifications are likely to be specific to ferroptosis and may markedly differ from the apoptosis-induced neoantigens. Thus the suggested similarity between these pathways is not convincing. "

We thank the reviewer for the insightful comments. We fully agree that current MHC-binding prediction algorithms have inherent limitations and cannot fully capture the complexity of antigen presentation, particularly in the context of post-translationally modified peptides that may arise during ferroptosis. Our *in silico* analysis was therefore intended as an exploratory, hypothesis-generating approach, illustrating the potential of antigen presentation prediction as a complementary read-out to the proteomic data, rather than as a definitive characterization of the immunopeptidome. We would also like to emphasize that the primary objective of this study was to investigate the adjuvanticity of ferroptotic lysates, and we clearly demonstrate that their immunogenicity is mediated by ATP release and calreticulin exposure, rather than by HMGB1.

To clarify this, we have revised the Discussion to explicitly frame these results as exploratory and to acknowledge the limitations of the predictive approach. We have also emphasized that mass spectrometry–based immunopeptidomics will ultimately be required to validate and extend these findings, while noting that this approach itself can have limited sensitivity for detecting low-abundance or modified peptides.

Line 531–536: "Given the inherent limitations of current MHC-binding prediction algorithms, our analysis should be viewed as an exploratory step. The use of MHCnuggets, which combines a low false-positive rate with a positive predictive value of approximately 60%, provides a conservative and reliable subset of predicted binders. Future studies using immunopeptidomics will be required to validate these findings and further elucidate the antigenic landscape associated with ferroptosis."

Regarding the use of a single prediction tool, we would like to point out that the algorithm employed in our analysis (MHCnuggets) has been benchmarked to achieve a positive predictive value (PPV) of approximately 60% and a very low false-positive rate. This makes it a suitable choice for a conservative exploratory analysis, providing confidence that the majority of predicted binders are true positives, even if some epitopes may have been missed.
While integrating multiple predictors can sometimes increase robustness, such an approach may also amplify shared systematic biases, as many tools rely on similar training datasets and model architectures. We therefore opted for a single, well-performing predictor to maintain internal consistency and minimize compounded bias.

Reviewer #3

"I thank the authors for the answers to my comments. I appreciate the format of the rebuttal letter very much. The data presented here is of good quality and the claims and conclusions are supported by the data. However, the link between the proteomics data and the mechanistic study is elusive. Indeed, despite the authors identified potential determinants mediating the increased immunogenic response (figure 7), they decided to focus on the canonical DAMPs (CRT, ATP and HMGB1). Investigating this DAMPs in ICD immunogenicity is very relevant, although the link with the proteomics data is not strong. Preliminary or negative data testing the newly identified proteins (or antigens) will be of great interest. Namely, the proteins indicated in figure 7E could have been tested. They could envision to test these candidates with immunoprecipitation to deplete the ICD lysate and prepare new DC vaccines. Alternatively, The authors might consider including the "Additional figure 1" in their manuscript. This immunopeptidomics data might deserve to be published in part or in full. Overall, this manuscript is of good quality and deserves to be published. "

We sincerely thank the reviewer for the positive evaluation of our manuscript and for the constructive suggestions to further explore the link between the proteomics data by performing additional mechanistic study. We fully agree that testing the newly identified proteins could provide additional insights and that the immunopeptidome data may represent an interesting resource for future work.

However, we believe that performing these additional experiments (immunoprecipitation-based depletion assays or functional testing of candidate proteins) goes beyond the scope of the present manuscript. Our primary objective was to establish ferroptotic glioma cells as a source of immunogenic antigens for dendritic cell vaccine development and to demonstrate the essential role of canonical DAMPs (CRT, ATP, HMGB1) in mediating this effect. While the proteomics dataset indeed suggests further potential determinants, we consider these findings as hypothesis-generating and will pursue them in follow-up studies.

To improve clarity, as requested by the reviewer, we have adapted **Fig. 7C-E** (now **Fig. 8C-E**) to clearly separate protein-level and peptide-level data within the same figure and we have also adjusted the order of the figurer.

Regarding 'Additional Figure 1,' we prefer not to include it in the main manuscript, as it illustrates the complexity of immunopeptidome analysis on DCs derived from different mice, which leads to considerable variation and requires further optimization and validation. Nevertheless, since all reviewer comments and responses will be available with the published manuscript, 'Additional Figure 1' will also be accessible to readers.

Comment 1. *"Figure 7: proteomics. The authors shows a heatmap in figure 7B. What statistical test was performed? Is this a one-way ANOVA? This should be indicated in legend and method section. Figure 7C-E the number of peptides presented on MHC I and MHC II is indicated in the protein volcano plots. Mixing experimental protein-level data with predicted (immuno-)peptides data is confusing. Overall, the take home message is not clear from the figure. Does this author want to highlight the high number of presented peptides in one of the conditions? The authors could consider separating the protein-level and peptide level data."*

We analyzed the differences using the limma package, which performs a moderated t-test. This is now included in the manuscript.

Lines 752–755: "Pairwise comparison testing (moderated t-test) between groups was then carried out using the limma package[76], with statistical significance for differential regulation set at an FDR cut-off value of 0.05 and a |log2(fold change)| greated than 1."

Lines 1189–1191: "**(B)** Proteins differentially abundant between groups (moderated t-test) were visualized in a heatmap following unsupervised hierarchical clustering of z-scored log2 reporter intensity values."

In addition, in the revised version of the manuscript, we modified **Fig. 7C-E (now Fig. 8C-E)** accordingly. Specifically, we removed the peptides from the volcano plots (**Fig. 8C, D**) to avoid mixing protein-level and peptide-level data.

We also updated the table to highlight the upregulated proteins that are common to both RSL3 (ferroptosis) and MTX (positive control, ICD) to improve clarity.

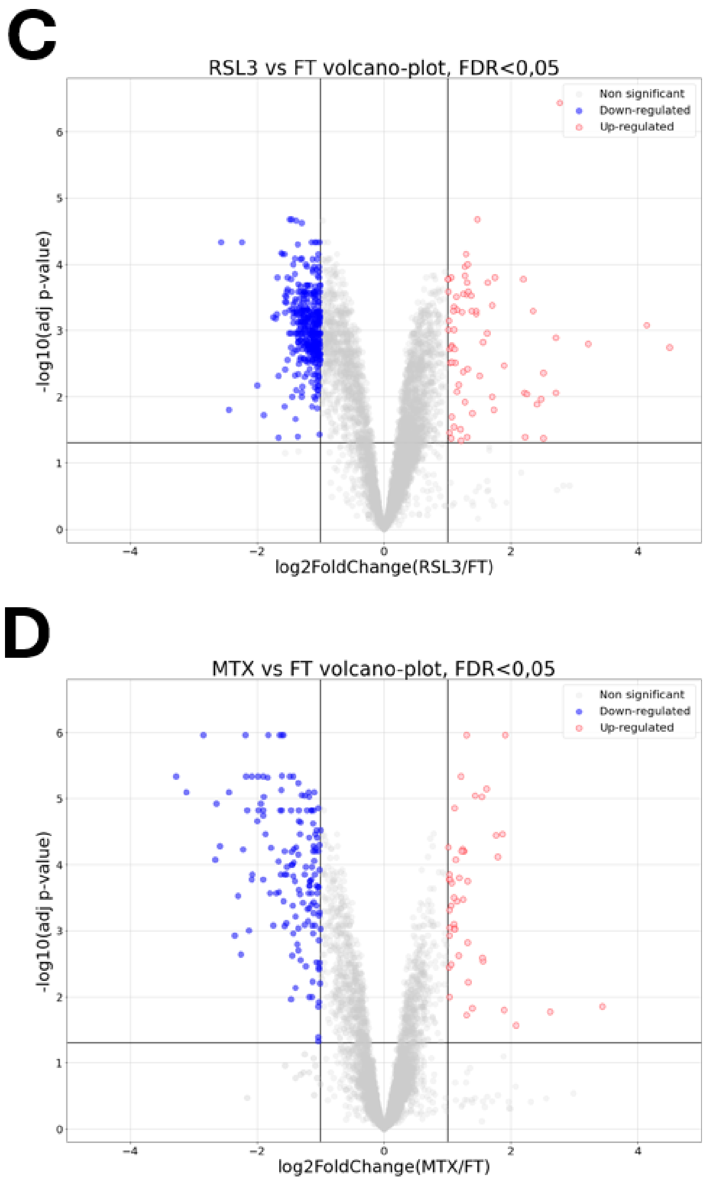

E

Up-regulated proteins which are common for RSL3 and MTX	
**MHCI**	**MHCII**
NAGPA	NAGPA
FIBG	FIBG
KIF23	KIF23
CENPE	CENPE
MMTA2	MMTA2
ANT3	ANT3
TROAP	TROAP
PRR11	PRR11
KI20A	KI20A
APOM	APOM
F13A	F13A
AURKA	AURKA
RETST	RETST
UPAR	

Peptides from these proteins which can be presented on MHCI or MHCII

**289 peptides**	**178 peptides**

Figure 8. **(C, D)** Differential protein expression analysis comparing RSL3 vs. F/T **(C)** and MTX *versus* F/T **(D)**. **(E)** Commonly upregulated proteins which contain peptides shared between RSL3- and MTX-treated lysates.

Comment 2. *"Figure 7: proteomics. The results section nicely describes the data on the prediction of the MHC-I or -II presented peptides. However, the figure is not clear. The author could also consider to include the prediction of presented peptides in another figure.*
The authors should consider re-phrasing "immunogenic peptides" to peptides presented on MHC-I or -II molecules. Indeed, most MHC-presented peptides are not immunogenic (i.e. triggering T cell activation)."

In the revised version of the manuscript, we have adapted **Fig. 7C-E (now Fig. 8C-E)** to clearly separate protein-level and peptide-level data within the same figure. We chose not to create a separate figure for the peptides, as we believe presenting them together with the corresponding protein data provides better context. However, all detailed information on peptides is available in **Supplementary Tables 3 and 4**.
In addition, we rephrased "immunogenic peptides" to "peptides presented on MHC-I or MHC-II molecules" throughout the manuscript, as suggested.

Comment 3. *Sup Figure 10: reconstitution of DAMPs in FT lysates*
"The data is impressive and add value to this manuscript by dissecting the mechanism at play. Figure 8 clearly shows the importance of those canonical DAMPs. Thus, answering a key question in the field of ICD and DC vaccination. Additionally, Sup. Figure 10 shows that individual reconstitution of the DAMPs is not sufficient to induce the immunogenic response from RSL3 lysate. Together, this data shows that while these DAMPs are essential, they are not sufficient for the immunogenic response mediated by ICD. The authors should consider adding this concept to the abstract.
The authors could consider switching figure 7 and 8 since the figure 7 test the hypothesis that ATP, CRT and HMGB1 are important for the immunogenicity of ICD while the figure 8 shows data suggesting novel molecular determinants mediating the immunogenicity of ICD."
We agree with the reviewer and we have added the following sentence to the abstract to better reflect the newly included data: "However, supplementing these DAMPs into DC vaccines loaded with non-ICD lysates did not restore efficacy to the level of the ferroptosis-based DC vaccine, suggesting a more complex mechanism beyond a purely DAMP-mediated effect"

We agree with the reviewer's suggestion and have switched Figures 7 and 8 accordingly.

Comment 4. *"I thank the authors for clarifying the aim of this study. I agree with data and results description. Figure 8E, the authors indicated a different -p-value between the text (line 394) and the figure 8E. Could the authors comment or adjust which p-value is correct. "*
Indeed, the p-values in **Fig. 8E** (**now Fig.7E**) and in the text (line 394) differ. In the text we state: **Lines 366-368:** *"Blocking HMGB1* (**Fig. 7E and Suppl. Fig. 8A**) *in ferroptotic lysates slightly reduced the effectiveness of the DC vaccines, but this difference was not statistically significant (p = 0.256)"*. Here, we compared the group with HMGB1 blockade (DC-GL261-RSL3 + anti-HMGB1 Abs, n = 7) against the control vaccine group without the addition of blocking HMGB1 Abs (DC-GL261-RSL3, n = 12). Since this p-value does not indicate a significant difference between the groups, we did not display it in the figure. Instead, the figure shows only the p-value between the negative control DC group (n = 18) and the full vaccine DC-GL261-RSL3 group (n = 12), which was p = 0.0034.

Comment 5. *"I thank the authors for generating the new data. They used in silico prediction to identify the potentially presented peptides on MHC-I and -II. This data is interesting but the presentation of this data in figure 7 is unclear. They authors should consider splitting the experimental protein-level data from the predicted (immuno-) peptide data.*
Additionally, the authors should consider using the "additional figure 1" to validate the proteomics data in figure 7. Although not perfect, this data seems relevant and could shed light on which antigens have a role in the ICD immunogenicity. Adding this data to the manuscript will also improve the value of this paper by further suggesting which antigens might be relevant for ICD immunogenicity. "
In the revised manuscript, we have adapted **Fig. 7C-E** (**now Fig. 8C-E**) to more clearly distinguish protein-level from peptide-level data within the same figure. We opted not to create a separate figure for the peptides, as presenting them alongside the corresponding protein

data provides better context. All detailed peptide information is, however, available in **Suppl. Tables 3 and 4**.

Regarding 'Additional Figure 1,' we prefer not to include it in the main manuscript, as it highlights the complexity of immunopeptidome analysis on DCs derived from different mice, which leads to considerable variation and requires further optimization and validation. However, since all reviewer comments and responses will be available with the published manuscript, 'Additional Figure 1' will also be accessible to readers.

Reviewer #4

"I co-reviewed this manuscript with one of the reviewers who provided the listed reports. This is part of the Nature Communications initiative to facilitate training in peer review and to provide appropriate recognition for Early Career Researchers who co-review manuscripts."

We thank the reviewer for the valuable comments and for supporting the Nature Communications initiative to train and recognize Early Career Researchers in peer review.

Reviewer #5

“The authors have made a concerted effort to address my prior critique. I have no further comments.”

We thank the reviewer for acknowledging our efforts and appreciate the time and attention devoted to evaluating our revised manuscript.

Reviewer #2

“The authors address my comment regarding the prediction of antigen presentation. While I still think this is a rather weak part of the manuscript, I also acknowledge that addressing this appropriately is beyond the scope of this work.”

We thank the reviewer for this comment and appreciate the constructive perspective. We agree that antigen-presentation prediction remains a complex and evolving field, and that a comprehensive analysis would extend beyond the scope of the present study. We have moved the prediction part to supplementary figures. We are grateful that the reviewer acknowledges our efforts to address the point within the limits of this work.

Reviewer #3

“The authors addressed all my concerns. I have no additional comments.”

We thank the reviewer for the thorough evaluation of our revised proposal. We appreciate the constructive feedback provided throughout the process and are pleased that all concerns have been satisfactorily addressed.

Reviewer #4

“I co-reviewed this manuscript with one of the reviewers who provided the listed reports. This is part of the Nature Communications initiative to facilitate training in peer review and to provide appropriate recognition for Early Career Researchers who co-review manuscripts.”

We fully appreciate the contribution of the Early Career Researcher who co-reviewed the manuscript as part of the Nature Communications peer-review training initiative.

Reviewer #6

“In the revised manuscript ‘Ferroptosis-armed dendritic cell vaccines for glioma immunotherapy’ (#NCOMMS-25-07518B), Mariia Saviuk and co-workers provide experimental evidence for the immunogenicity, in terms of both adjuvanticity and antigenicity, of ferroptosis and set the route for taking advantage of it in the design of DC-based vaccines to treat gliomas. The message of the article is important, novel, and within scope for Nature Communications. The manuscript is well written, precise and easy to follow. Findings are abundant, technically diverse and well presented. However, some evidence from the series of in vitro and in vivo (mouse) experimentation is not yet compelling. Hence we (my co-reviewer and I) are listing a number of concerns and making suggestions that should enhance the manuscript. Given the great future importance of these studies, every possible artifact and misinterpretation must be ruled out. In summary, we believe the article by Mariia Saviuk and colleagues represent a strong candidate for publication in Nature Communication upon revision.”

We thank the reviewers for their thorough and constructive evaluation of our manuscript. We greatly appreciate the positive assessment of the novelty, importance, and clarity of our work, as well as the acknowledgment of the breadth and technical strength of the experimental data.

We carefully considered all concerns and suggestions raised by both the reviewer and the co-reviewer. In the revised manuscript, we have addressed each point in detail and implemented the recommended clarifications and additional controls where feasible.

We believe these revisions have substantially strengthened the manuscript and improved the robustness of our conclusions.

“Major point 1: Figure 2C. These reviewers are surprised to see MTX-induced ICD protecting only the 30-35% of animals. Also, in Figure 4 and 6, MTX does not seem to be so immunogenic (low vaccination potential and low % of effector and memory T cells). This can be due to intrinsic or acquired resistance of gliomas to MTX. Verification of the induction of ICD with other ICD-inducers such as doxorubicin or oxaliplatin would be very useful and reassuring.”

We performed an additional experiment evaluating survival following therapeutic vaccination with DC vaccines generated from glioma cell lysates induced by doxorubicin or oxaliplatin. Therapeutic vaccination initiated on day 7 after intracranial tumor inoculation resulted in increased survival, with 60% survival observed for doxorubicin-based DC vaccines, 16.7% survival for mitoxantrone-based DC vaccines and 0% survival for oxaliplatin-based DC vaccines but with significant prolongation of median survival comparing to negative control groups.

Line 267-272: “Importantly, despite the positive control DC-GL261-MTX-therapeutic vaccination was not as effective as the ferroptotic one, DC vaccines loaded with other two immunogenic apoptotic lysates (DC-GL261-DOX and DC-GL261-OXA) even if in a different extent, significantly improved mouse survival compared with vaccination with PBS or unloaded DCs, confirming the reliability of our data and demonstrating that the cell death inducers are the main driver of differential preclinical outcomes (**Suppl. Fig. 3A-D**).”

However, importantly, direct comparison of apoptosis-inducing agents is beyond the primary scope of this study, although these observations may warrant further dedicated investigation.

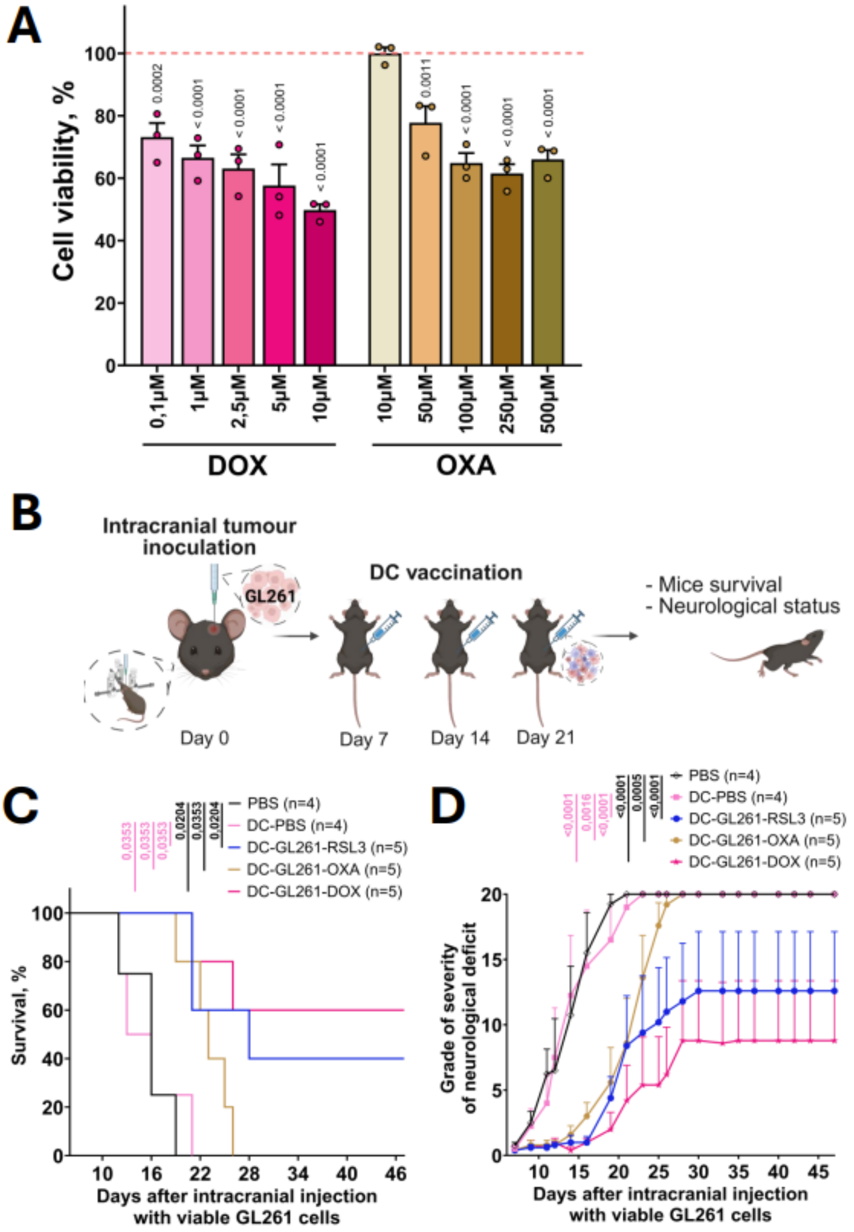

Suppl. Figure 3. **Therapeutic vaccination with DC vaccine loaded with apoptotic lysates**

(A) Cell death was induced by DOX (0,1 µM – 10 µM) or OXA (10 µM – 500 µM) on glioma GL261 cell line. Statistical significance was determined using a one-way ANOVA followed by Dunnett multiple comparisons test.

(B) Procedure to assess the efficacy of DC vaccines in a more clinically relevant therapeutic setting using an orthotopic intracranial mouse model. Mice were injected intracranially with viable glioma GL261 cells on day 0, followed by administration of the corresponding DC vaccine on days 7, 14 and 21. The lysates were prepared by inducing ferroptosis in GL261 cells with RSL3 (2.5 mM, DC-GL261-RSL3) or by inducing apoptosis in GL261 cells with OXA (100 mM, DC-GL261-OXA) or DOX (5 mM, DC-GL261-DOX). Control groups included mice injected with unloaded DCs (negative control, DCs) or PBS (negative control). Mouse survival **(C)** and neurological status **(D)** were assessed following intracranial injection with viable glioma GL261 cells and subsequent therapy with ferroptosis-armed DC vaccines. Survival and neurological status were monitored for up to 47 days. Mouse survival was analyzed using the Mantel-Cox log-rank test. Statistical analysis of the neurological status of the mice was performed using a two-way ANOVA followed by Tukey's multiple comparisons correction.

"Major point 2. Figures 3B and 4B. According to the author model, therapeutic use of DC-GL261-RSL3 provide benefit better than any other therapeutic vaccine tested. However, this strategy seems to work

better when tumors are already in progression (7 days after inoculation) than when tumors are in early development (2 days after inoculation) with survival rates of 40% at 60 days after tumor inoculation in the first case and 10% at 40 days after tumor inoculation in the second case. How do the authors explain this discrepancy?"

We observed approximately 30–40% long-term survival when vaccination was initiated on days 7, 14, and 21 after intracranial tumor inoculation, compared with approximately 10% when vaccination began on days 2, 9, and 16. Importantly, negative control groups (unloaded DCs) displayed comparable tumor progression kinetics in both experiments, with all animals succumbing at 26–27 days after inoculation, indicating similar baseline tumor growth.

Although there appears to be a numerical difference in survival between early and later vaccination schedules, this difference was not statistically significant. Therefore, we interpret the observed variation as normal experimental variability rather than evidence of a true mechanistic difference between early and delayed vaccination. The overall therapeutic trend of DC-GL261-RSL3 treatment remained consistent across experiments.

"Major point 3. Figure 5G,H. By using TLR4-/- DCs the authors are intended to outline that even if LPS can work as an adjuvant in DC maturation, it does not affect DC ability to mount a protective immune response in DC-GL261-RSL3 vaccines. However, treatment of TLR4-/- DCs with LPS is a redundant control and add less. More interesting and convincing is the experiment showed in Supplementary Figure 3E-G. In the opinion of these reviewers, it is surely better to show data in Supplementary Figure 3E-G as main data and data in Figure 5F-H as supplementary data."

We agree with the reviewer. Following the reviewer's suggestion, we moved the data from Supplementary Figure 3E-G into the main figures (now **Fig. 5E-G**) and relocated Figure 5F-H to the Supplementary section (now **Suppl. Fig. 4**).

"Major point 4. Figure 6E. Additional experiments, such as blocking of CD39, are required to verify the features of CD44highCD62L-CD39+ cells as a newly discovered tissue-resident memory T cell population."

We agree that CD39 plays an important role in regulating T-cell function and that CD39 blockade could, in principle, help further dissect the phenotype and functionality of the $CD44^{high}CD62L^{-}CD39^{+}$ population. However, we think that performing CD39-blocking experiments is beyond the scope of the current study for several reasons. First, the main focus of this work is to define the immunogenicity of ferroptosis-induced lysates and the therapeutic efficacy of the resulting DC vaccines, rather than to functionally characterize new T-cell subsets. CD39 blockade is mechanistically complex and would require a dedicated set of in vivo experiments, including multiple doses, timing regimens, and appropriate controls like CD73 co-blockade, since the CD39-CD73 axis functions as a biochemical cascade. Adding CD39-blocking experiments would substantially expand the scope, requiring new survival cohorts and mechanistic assays, which cannot be reasonably incorporated into the current framework without turning the manuscript into a separate mechanistic study.

For these reasons, we believe that CD39-blocking experiments, although scientifically valuable, would shift the manuscript away from its central objective. We addressed this aspect in the Discussion as a potential direction for future research at line 540-545.

“Major point 5: Figure 7. What is the rationale to investigate these selective alarmins? Why have the authors excluded the role of other DAMPs released during ICD such as IFNs-I (PMID: 25344738) and Ann-I (PMID: 26516201), the last being involved in DC-dying cancer cell juxtaposition?”

We fully agree that these pathways are highly relevant to ICD biology. However, we chose not to analyze them experimentally in this study. The main objective of this work is to determine whether ferroptotic lysates can induce DC maturation, generate tumor-specific T-cell responses, and provide therapeutic efficacy in intracranial glioma models. To maintain a focused and coherent narrative, we restricted our mechanistic investigation to the canonical core DAMPs (CRT exposure, ATP release, and HMGB1 secretion) because these are the minimal, broadly accepted ICD markers across diverse cell death modalities.

To acknowledge the reviewer’s point, we added a paragraph in the Discussion section addressing the potential roles of IFN-I and Annexin A1 in ferroptosis-associated immunogenicity and identifying them as important directions for future mechanistic refinement.

Line 536-539: “Indeed, although these pathways contribute to the overall immunogenicity of ferroptotic cells, future studies will be needed to clarify the contribution of additional DAMPs to ferroptosis-driven antitumor immunity, such as type I interferon and Annexin A1”

“Major point 6: Figure 7A and discussion. The authors see CRT surface exposure at a very early time point (1.5 hours) and, as they discuss, this finding is at odds with previous literature. How do they interpret these data? Could they be more accurate in the discussion?”

We agree that the detection of calreticulin (CRT) surface exposure at an early time point (1.5 h) occurs earlier than reported for other forms of immunogenic cell death, as apoptosis. In contrast, Wiernicki et al., 2022 observed a significant increase in CRT exposure approximately 4 h after ferroptosis induction. We interpret this difference as reflecting cell-type-specific death kinetics, as our previous observations (Turubanova et al., 2019) showed already that glioma GL261cells exhibit earlier CRT translocation compared with fibrosarcoma MCA205 cells after photodynamic cell death induction.

Furthermore, we do not propose that early CRT exposure is a universal feature of all ICD modalities. We now acknowledge in the Discussion that the timing of CRT externalization can vary depending on both the mode of cell death and the cellular context (see Discussion section, line 459-467).

“Major point 7: Figure 8. The authors provide data on the role of ferroptosis in molding the peptidome of cancer cells. However, as the authors admit, this is a chapter of another story. Since there is no functional data showing the immunogenicity of ferroptotic cancer cell peptides, can the authors either provide at least a list of tumor-specific and/or tumor-associated antigens among the 289-MHC-I-restricted peptides to have an idea of non-self molecules that can be able to mount an anti-tumor immune response and even better to synthetize a couple of them and test through DC-mediated T cell cross-priming (but these authors understand that antigen synthesis and testing may be out of the scope) or remove it and write a second story?”

We fully agree that functional validation of individual ferroptosis-associated peptides would represent an important and valuable line of investigation. However, we also agree

that peptide synthesis and T cell cross-priming assays would constitute a substantial and independent study, well beyond the scope of the this manuscript.

After carefully reconsidering the role of the peptidome analysis in the context of this work, we have revised both the structure and interpretation of these data. Importantly, we do not present the predicted MHC-I restricted peptides as functionally validated tumor antigens, nor do we claim direct evidence for their immunogenicity. Instead, we position this analysis as an exploratory resource that illustrates how ferroptosis may qualitatively reshape the antigenic landscape of tumor cells.

To avoid overstating the conclusions and to maintain a focused narrative, we have moved the antigen-prediction data to the Supplementary Information (**Suppl. Fig. 11C**) and have revised the main text to clearly state that these findings are hypothesis-generating. We also mentioned that at Material and Methods section, line 807-810.

We believe that retaining these data in the Supplementary section provides transparency and added value for the field, while ensuring that the main manuscript remains focused on the experimentally validated immunogenicity and therapeutic efficacy of ferroptosis-based DC vaccines.

"Major point 8: material and methods. Why do the authors exclude IL-4 to differentiate DCs from bone marrow cells?"

Many widely accepted murine DC vaccination studies generate BMDCs using GM-CSF alone in preclinical tumor models (J. Han et al., 2023; M. K. Kim & Kim, 2019; Sauter et al., 2022; Wiernicki et al., 2022). Nonetheless, we agree that incorporating IL-4 and maturation cocktails (e.g., TNF, PGE_2, TLR agonists) could be valuable for future studies, especially in a translational context (Gilboa, 2007; Herrera et al., 2002; Kalantari et al., 2011).

"Minor point 1: Figure 1A-D. Please add control group to each plot (also a dotted line as in panels E-H)."

Done

"Minor point 2: Supplementary Figure 4. Cell populations in the plots showing surface expression of CD80, CD86 and MHC-II are flattened on the right side. Please check the sample analysis and correct the plots moving the populations on the center to increase in value data."

We have increased the axes of dot plots to move the populations closer to the center.

"Minor point 3: Supplementary Figure 8D. Please show graphs with isotypes before and graphs with specific Abs after."

Done

"Minor point 4: line 270. Please correct "the synthetic the MHC" with "the synthetic MHC"."

Done

"Minor point 5: line 333. Please correct "CD44lowCD62L- effector memory cells" with "CD44highCD62L- effector memory cells"."

Done

"Minor point 6: line 368. Please add "E" to "Fig.7"."

Done

“Minor point 7: line 450. Please correct “DAMPs release” with “DAMP release”.”
Done

“Minor point 8: line 460. Please add “)” after “unregulated cell death59”.”
Done

“Minor point 9: line 552, authors use reference 46 to support the concept of lipid peroxidation-induced modifications of proteins and lipids. Reference 46 is a review and hence does not support the author sentence. Please add the reference of an original article showing that.”
We have additionally supported the reference 46 with an original research article that demonstrates lipid peroxidation induced formation of protein adducts with lipids (reference 74).

“Minor point 10: line 614. Please add “)” after “non-oxidized lipids”.”
Done

“Minor point 11: line 875. Please remove the space between IFN- and γ.”
Done

“Minor point 12: line 1102. Please correct “DCs maturation” with “DC maturation”.”
Done

“Minor point 13: legend graphical abstract. Please insert panels 1, 2 and 3. Only sub-panels a, b, c of panel 2 are described.”
We adapted the graphical abstract and the figure legend according to the comment.

Reviewer #7

“I co-reviewed this manuscript with one of the reviewers who provided the listed reports. This is part of the Nature Communications initiative to facilitate training in peer review and to provide appropriate recognition for Early Career Researchers who co-review manuscripts.”
We appreciate the involvement of the Early Career Researcher who contributed to the review as part of the Nature Communications co-review initiative.
